# Efficacy of Nilotinib in Patients With Moderately Advanced Parkinson Disease: A Randomized Clinical Trial

**DOI:** 10.1001/jamaneurol.2020.4725

**Published:** 2020-12-14

**Authors:** Tanya Simuni, Brian Fiske, Kalpana Merchant, Christopher S. Coffey, Elizabeth Klingner, Chelsea Caspell-Garcia, David-Erick Lafontant, Helen Matthews, Richard K. Wyse, Patrik Brundin, David K. Simon, Michael Schwarzschild, David Weiner, Jamie Adams, Charles Venuto, Ted M. Dawson, Liana Baker, Melissa Kostrzebski, Tina Ward, Gary Rafaloff

**Affiliations:** 1 Department of Neurology, Northwestern University Feinberg School of Medicine, Chicago, Illinois; 2 Research Programs, Michael J. Fox Foundation for Parkinson’s Research, New York, New York; 3 Department of Neurology, Northwestern University, Chicago, Illinois; 4 Department of Biostatistics, College of Public Health, University of Iowa, Iowa City; 5 The Cure Parkinson’s Trust, London, England; 6 Research and Development, The Cure Parkinson’s Trust, London, England; 7 Van Andel Institute, Center for Neurodegenerative Science, Grand Rapids, Michigan; 8 Beth Israel Deaconess Medical Center, Harvard Medical School, Boston, Massachusetts; 9 Department of Neurology, Massachusetts General Hospital, Boston, Massachusetts; 10 Consultant, Austin, Texas; 11 Department of Neurology, University of Rochester, Rochester, New York; 12 CHET CTCC, University of Rochester, Rochester, New York; 13 Department of Neurology, Johns Hopkins University School of Medicine, Baltimore, Maryland; 14 Steven’s Institute of Technology, Marlboro, New Jersey

## Abstract

**Importance:**

There is a critical need for careful and independent validation of reported symptomatic efficacy and dopaminergic biomarker changes induced by nilotinib in Parkinson disease (PD).

**Objectives:**

To assess safety and tolerability of nilotinib in participants with moderately advanced PD. Secondary and exploratory objectives were to assess its affect on PD disability, pharmacokinetics, cerebrospinal fluid (CSF) penetration, and biomarkers.

**Design, Setting, and Participants:**

This was a 6-month, multicenter, randomized parallel-group, double-blind, placebo-controlled trial. Recruitment was from November 20, 2017, to December 28, 2018, and follow-up ended on September 9, 2019. The study was conducted at 25 US sites. The study approached 173 patients, of whom 48 declined, 125 were screened, and 76 who received a stable regimen of PD medications were enrolled (39% screen failure).

**Interventions:**

Participants were randomized 1:1:1 to placebo, 150-mg nilotinib, or 300-mg nilotinib once daily orally for 6 months, followed by 2-month off-drug evaluation.

**Main Outcomes and Measures:**

The primary outcomes were safety and tolerability. The tolerability end point was defined as the ability to complete the study while receiving the assigned dose. An active arm was considered tolerable if the percentage of participants meeting the tolerability end point for that group was not significantly lower than the percentage observed in the placebo group. Secondary outcomes included change in PD disability (Movement Disorder Society Unified Parkinson’s Disease Rating Scale [MDS-UPDRS], Part II OFF/ON). Exploratory outcomes included serum and CSF pharmacokinetic profile, and CSF dopaminergic biomarkers.

**Results:**

At baseline, mean (SD) participants’ age was 64.6 (7.5) years, 52 were male (68%), mean (SD) disease duration was 9.9 years (4.7), MDS-UPDRS Part 1-3 OFF score was 66.4 (19.3), ON score was 48.4 (16.2), and Montreal Cognitive Assessment score was 27.1 (2.2). The number of participants who completed the study receiving the assigned dose were 21 (84%), 19 (76%), and 20 (77%) in the placebo, 150-mg, and 300-mg arms, respectively. Both active doses had acceptable safety profile. The most common reasons for drug suspension were asymptomatic, dose-dependent elevations of amylase, and/or lipase. Nilotinib, 150 mg and 300 mg, exhibited worse MDS-UPDRS-3 ON scores compared with placebo, achieving significance for nilotinib, 300 mg, at month 1 (*P* < .01). There was no difference in the change of MDS-UPDRS-3 OFF from baseline to 6 months between groups (*P* = .17). Cerebrospinal fluid/serum ratio of nilotinib concentration was 0.2% to 0.3%. There was no evidence of treatment-related alteration of dopamine metabolites in the CSF.

**Conclusions and Relevance:**

While we demonstrated acceptable safety and tolerability of nilotinib in our cohort, the low CSF exposure and lack of biomarkers effect combined with the efficacy data trending in the negative direction indicate that nilotinib should not be further tested in PD.

**Trial Registration:**

ClinicalTrials.gov Identifier: NCT03205488

## Key Points

**Question:**

Do the safety, tolerability, exploratory clinical outcomes, brain penetration, and biomarker profile of nilotinib in aggregate support its development for treatment of Parkinson disease (PD)?

**Findings:**

In this 6-month, multicenter, randomized placebo-controlled clinical trial of 76 participants with moderately advanced PD, nilotinib at 150-mg and 300-mg daily doses met prespecified safety and tolerability criteria. There was no evidence of symptomatic benefit of nilotinib on any measures of PD disability and there was trend toward worsening in the motor function in active treatment arms; in the cerebrospinal fluid, nilotinib level was less than 0.3% of that in the serum and failed to change dopamine metabolites levels.

**Meaning:**

While nilotinib demonstrated acceptable safety and tolerability in this cohort, the low cerebrospinal fluid exposure, lack of biomarkers effects, and efficacy data trending in the negative direction indicate that further testing of nilotinib in treatment of Parkinson disease is not warranted.

## Teaser

This randomized clinical trial assesses safety and tolerability of nilotinib in participants with moderately advanced Parkinson disease.

## Introduction

Parkinson disease (PD) is the second most common neurodegenerative disease, affecting 1% of the population older than 65 years.^1^ Slowing PD progression remains a major unmet need.^2^ Nilotinib, an approved therapy for chronic myeloid leukemia, works predominantly via BCR-Abelson tyrosine kinase (c-Abl) inhibition. It is neuroprotective in animal models of PD,^3,4,5^ leading to an interest in its development to slow PD progression. However, nilotinib has safety and tolerability liability.^6^ At the time of the study launch, the clinical experience with nilotinib in PD was limited to a small study,^7^ warranting further careful investigation. Since then, results of a phase 2 study,^8^ undertaken by the same group, were reported in 2020 where nilotinib showed acceptable safety and tolerability in moderately advanced PD and favorable changes in exploratory biomarkers of PD pathophysiology without significant effects on clinical outcomes. The primary objective of this multicenter study was to assess safety and tolerability of nilotinib in participants, with moderately advanced PD with secondary and exploratory outcomes, respectively, of effect on PD disability, pharmacokinetics, and biomarkers’ assessment.

## Methods

### Trial Design

This was a 6-month, multicenter, randomized parallel-group, double-blind, placebo-controlled trial. Detailed trial design and schedule of activities are outlined in the trial protocol in Supplement 1. Briefly, participants underwent a screening visit that included clinical assessments, safety laboratory testing, electrocardiogram (ECG), and a lumbar puncture (LP) before being randomized in 1:1:1 allocation to 150 mg or 300 mg of nilotinib or matching placebo once daily. Following randomization, in-clinic visits occurred on days 7, 14, and then monthly, with additional safety visits if necessary. Safety was monitored by standard laboratory tests and ECG at all visits. Final assessments were conducted at 6 months followed by off-drug safety assessments at months 7 and 8. Temporary study drug suspensions and rechallenges were allowed based on tolerability and/or prespecified changes in the laboratory parameters or ECG. Participants who permanently discontinued study drug were terminated from the study. A second LP was required at month 3 with an optional LP at 1 month off the study drug. Recruitment occurred between November 2017 and December 2018; the last participant completed the study in September 2019. The protocol was approved by the ethics committee at each participating site and the clinical coordination center (University of Rochester). All participants provided written informed consent. The trial was conducted in accordance with the Principles of the Declaration of Helsinki and Good Clinical Practice Guidelines. An independent data safety monitoring board reviewed blinded and unblinded data on regular basis. The authors attest to compliance with the protocol, accuracy, and completeness of the data and analyses.

### Setting and Participants

Participants were recruited from 25 US Parkinson study group sites. Eligibility criteria included a diagnosis of PD for more than 5 years using established diagnostic criteria,^9^ age 40 to 79 years, Hoehn and Yahr stage 2.5 or 3 in the medications’ on-state,^10^ and stable regimen of PD medications that had to include levodopa. Use of monoamine oxidase B (MAO-B) inhibitors was initially exclusionary but subsequently allowed to reduce screen failures, provided that a stable dose had been reached 60 days prior to enrollment. Participants were excluded if they had a diagnosis of atypical parkinsonism, clinically significant depression, history of cardiovascular conditions, liver or pancreatic disease, presence of laboratory or ECG abnormalities, presence of dementia, a Montreal Cognitive Assessment score less than 21, or any other conditions or concomitant medications associated with increased risk of use of nilotinib as per package insert.^6^

### Randomization and Interventions

Nilotinib, 150 mg, and matching placebo were provided by the drug manufacturer, Novartis in-kind. University of Rochester Clinical Materials Services Unit delivered study drug kits to the participating sites according to the randomization assignments performed by the Biostatistics Coordinating Center (University of Iowa) using random permuted blocks of sizes 3 and 6. All participants started with 1 capsule daily (150 mg or matching placebo), with dose escalated after 2 weeks to 2 capsules daily per randomization assignment.

### Outcomes

The primary outcomes were safety and tolerability of 2 doses of nilotinib vs placebo. The tolerability end point was defined as the percentage of participants who completed the study taking their assigned dose across the 3 study arms, regardless of temporary drug interruptions. A treatment arm was deemed “tolerable” if the percentage of participants meeting the tolerability end point in that arm was not significantly lower than the percentage observed in the placebo arm. For the power calculations, tolerability threshold was set at 30% less than placebo for each active arm with an assumption that the placebo group will have 90% tolerability. Safety was assessed by examining the frequency of treatment-related serious adverse events (SAEs) between groups. Adverse events were collected at every visit and rated by the investigator on severity and causality. An independent medical monitor reviewed all SAEs and made adjudications on causality, severity, and expectedness.

A key secondary objective was to conduct a futility analysis within each treatment group by comparing the observed change in the Movement Disorder Society Unified Parkinson’s Disease Rating Scale (MDS-UPDRS)^11^ Part III score in the PD medications on-state between baseline and month 6 with the one previously reported.^7^ Other secondary objectives of the study were to (1) establish the degree of symptomatic effect of nilotinib as measured by change in MDS-UPDRS Part III on-state between baseline and 1 month, 6 months, and 30 days off–study drug and (2) to explore the effect of nilotinib on progression of PD disability as measured by change in the MDS-UPDRS Part III score in the defined medications’ off-state between baseline and 6 months. Other clinical exploratory outcomes prespecified in the statistical analysis plan (Supplement 2) were changes in disability, quality of life, and functional status from baseline to 6 months.

### Biospecimen Collection for Exploratory Pharmacokinetics and Biomarker Analyses

At 3 months, predose trough and postdose maximum concentration samples of serum and CSF were collected approximately 2 hours following an in-clinic dose (reported T_max_ of approximately 2-3 hours).^12,13^ See the eMethods in Supplement 3 for details of analytical methods and results. Additional blood, serum, plasma, DNA, and CSF samples were stored for future exploratory research.

### Statistical Methods

Tolerability and safety were assessed by comparing the proportion of study participants who met the definition of tolerability or any treatment-related SAE in the placebo group vs each of the treatment groups, with a 1-sided Fisher exact test using a significance level of .05. Further comparisons examined types of AEs, SAEs, dose suspensions, and study drug discontinuations across the 3 groups. We also examined a more stringent definition of tolerability, requiring participants to complete the study while receiving their assigned treatment with no temporary dose suspensions during the study.

Linear mixed models were used to compare other secondary and exploratory objectives. The key secondary objective involved a single group hypothesis within each PD group to assess “futility” for replicating the observed difference in the prior study that observed a difference of 7.0 in the UPDRS score.^7^ Because we used the MDS-UPDRS, we included a correction factor of 1.4 as specified in Goetz et al.^14^ We conducted a futility test within each nilotinib dose group based on a null hypothesis that the observed difference was greater than a 9.8-unit reduction on the MDS-UPDRS Part III ON score (7.0 × 1.4) vs an alternative that the reduction was less than 9.8 units. For other comparisons, a global 2-*df* test was used to test for any differences among the 3 groups. If the global test was significant, stepdown pairwise comparisons were used to further explore any observed differences. Similar nonlinear mixed models were used to assess the exploratory clinical outcomes. Pharmacokinetic and biomarker results were summarized by nilotinib dose level using geometric means and 95% confidence intervals. Association between CSF nilotinib concentrations and dopamine turnover indices WAS assessed by Spearman correlation.

### Sample Size

We assumed that at least 90% of participants taking placebo would meet the study definition of tolerability and power the study to detect an absolute decrease of 30% or greater in tolerability for active treatment arms vs placebo. Hence, the sample size was chosen to provide sufficient power to compare an expected 90% vs 60% or lower tolerability rate in the placebo vs each of the treatment groups. Under these assumptions, 75 total participants provided at least 80% power at the 1-sided .05 significance level.

## Results

### Participants

**Figure 1.**
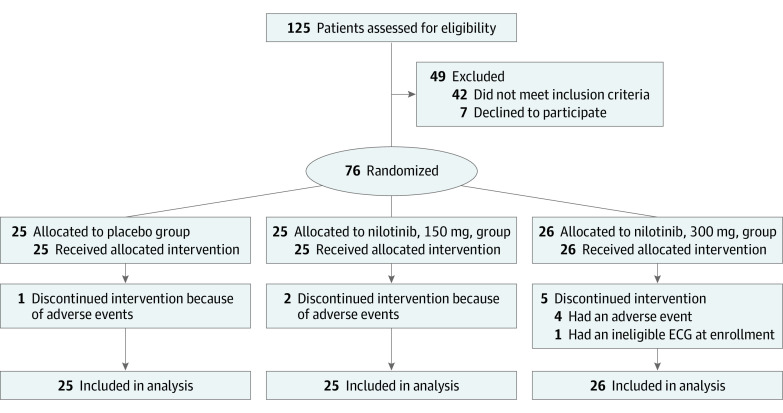
Flow of Participants in NILO-PD Study ECG indicates electrocardiogram.

Of 125 screened participants, 76 were enrolled and randomized (Figure 1). The major reasons for exclusion were comorbid conditions (n = 12), ECG abnormalities (n = 10), cardiovascular conditions (n = 7), and low Hoehn and Yahr stage (n = 7). Baseline characteristics of the enrolled cohort are detailed in Table 1. The groups were generally well balanced. Both doses of nilotinib were tolerable, with 21 (84%), 19 (76%), and 20 participants (77%) completing the study receiving the assigned dose, respectively, for the placebo and 150-mg and 300-mg nilotinib groups (*P* = .36 and *P* = .39 for nilotinib, 150 mg and 300 mg, respectively, vs placebo) (Table 2). However, there were more premature withdrawals owing to AEs in the nilotinib, 300 mg, group (Table 2). Of the 8 premature withdrawals, 1 was in the placebo arm (tremor); 2 in the nilotinib, 150 mg, arm (anxiety and increase in lipase); and 5 in the nilotinib, 300 mg, arm (increase in lipase in 2, arthritis, arrhythmia, and an abnormal ECG). The latter was present at screening but was not identified until randomization and therefore was not considered treatment related.

**Table 1.** Baseline Demographics and Disease Characteristics

Measure	Mean (SD)		
	Placebo (n = 25)	Nilotinib, 150 mg (n = 25)	Nilotinib, 300 mg (n = 26)
Age, y	65.5 (6.8)	61.2 (7.4)	66.9 (7.3)
Male, No. (%)	16 (64)	15 (60)	21 (81)
White race, No. (%)	24 (96)	23 (92)	25 (96)
Non-Hispanic ethnicity, No. (%)	24 (96)	25 (100)	25 (96)
Disease duration, y	9.4 (4.9)	8.5 (3.2)	11.7 (5.2)
Age at diagnosis, y	56.2 (6.8)	52.7 (7.6)	55.2 (9.3)
MDS-UPDRS			
Total OFF score	63.8 (21.3)	65.0 (16.0)	70.2 (20.2)
Total ON score	46.2 (17.8)	46.9 (15.1)	51.8 (15.7)
H/Y stage, No. (%)			
0-2	4 (16)	5 (20)	1 (4)
3	21 (84)	20 (80)	25 (96)
Levodopa equivalent daily dose	1066.5 (519.7)	971.8 (251.4)	1012.5 (390.5)
Class of symptomatic therapy, MAOB inhibitors, No. (%)	11 (44)	8 (32)	12 (46)
MoCA score^a^	26.9 (2.4)	27.4 (1.9)	27.0 (2.4)
Mattis Dementia Rating Scale	138.1 (5.7)	137.8 (7.8)	137.7 (6.3)
BDI II score	6.6 (4.9)	7.3 (5.4)	6.6 (4.3)
PDSS	106.6 (21.8)	115.3 (11.6)	103.2 (18.6)
Modified SE/ADL			
Off	73.2 (16.5)	77.3 (17.8)	77.0 (14.9)
On	86.8 (8.6)	92.2 (5.8)	87.0 (17.9)
PDQ-39 total score	19.0 (12.7)	18.9 (9.4)	18.4 (10.2)
EQ-5D			
Summary index	0.8 (0.1)	0.8 (0.1)	0.8 (0.2)
Health score	70.6 (25.1)	71.5 (23.3)	76.0 (15.9)

Abbreviations: BDI, Beck Depression Inventory; EQ-5D, EuroQol 5 Dimensions; H/Y, Hoehn and Yahr; MDS-UPDRS, Movement Disorder Society Unified Parkinson’ Disease Rating Scale; MAOB, monoamine oxidase type B; MoCA, Montreal Cognitive Assessment; PDQ-39, Parkinson’s Disease Questionnaire Quality of Life scale; PDSS, Parkinson Disease Sleep Scale; SE/ADL, Modified Schwab and England Activities of Daily Living.

^a^ MoCA Score is education-adjusted.

**Table 2.** Safety and Tolerability Outcomes

Variable	No. (%)		
	Placebo (n = 25)	Nilotinib, 150 mg (n = 25)	Nilotinib, 300 mg (n = 26)
Primary tolerability			
Tolerability	21 (84)	19 (76)	20 (77)
Primary safety			
Treatment-related serious adverse events	0	0	1 (4)
Other safety and tolerability			
Any adverse event	22 (88)	23 (92)	23 (88)
Any serious adverse event	2 (8)	1 (4)	1 (4)
Dose suspensions^a^	3 (12)	6 (24)	5 (19)
Study drug discontinuations^b^	1 (4)	2 (8)	5 (19)
Tolerability with no dose suspensions	20 (80)	18 (72)	17 (65)
Serious adverse events			
Arrhythmia	0	0	1 (4)
Abdominal pain	1 (4)	0	0
Gastroesophageal reflux disease	1 (4)	0	0
Suicidal ideation	0	1 (4)	0
Adverse events reported by more than 10% in any group			
Fall^c^	5 (20)	4 (16)	0
Lipase increased	4 (16)	7 (28)	6 (23)
Amylase increased	2 (8)	4 (16)	4 (15)
Nasopharyngitis	5 (20)	2 (8)	2 (8)
Fatigue	4 (16)	4 (16)	0
Nausea	0	2 (8)	4 (15)
Headache	2 (8)	1 (4)	3 (12)
Dizziness	1 (4)	4 (16)	0
Gastroesophageal reflux disease	4 (16)	0	0
Anxiety	0	3 (12)	1 (4)
Myalgia	3 (12)	0	0
Skin abrasion	3 (12)	0	0

^a^ All dose suspensions are owing to adverse events. Values are presented as number of participants experiencing the event at least once (%).

^b^ All drug discontinuations are owing to adverse events, except for one in the nilotinib, 300 mg, group, which was owing to ineligibility.

^c^ Indicates significant group differences (*P* < .05).

There were 16 drug suspensions in 14 participants, 10 of whom resumed the study drug (Table 2). The most common reasons for dose reduction or temporary suspension were increases in lipase and/or amylase, although with no significant imbalance between nilotinib groups and no associated clinical symptoms. There were more instances of AEs for elevation of lipase and amylases in the active treatment arms vs placebo, but the differences were not significant and none of these were symptomatic. Dose reduction/suspension and rechallenges were done based on the protocol prespecified laboratory and other safety parameters (see the trial protocol in Supplement 1) as advised by the clinical monitor who was blinded to the group assignment. There were no differences between groups when examined for a more stringent definition of tolerability requiring no dose suspensions (Table 2). There was no difference in the numbers of SAEs between the groups: 2 in the placebo group (gastroesophageal reflux and suicidal ideations), 1 in the 150-mg group (abdominal pain), and 1 in the 300-mg group (cardiac arrhythmia). Only 1 SAE was considered treatment related (arrhythmia in nilotinib, 300 mg, group). Adverse events presented in the order of frequency are listed in Table 2. The most common AE was falls, and interestingly, there was significantly lower incidence of falls in the 300-mg dose group (n = 0) compared with the 150-mg dose (n = 4) and placebo (n = 5) groups.

### Secondary Outcomes

**Figure 2.**
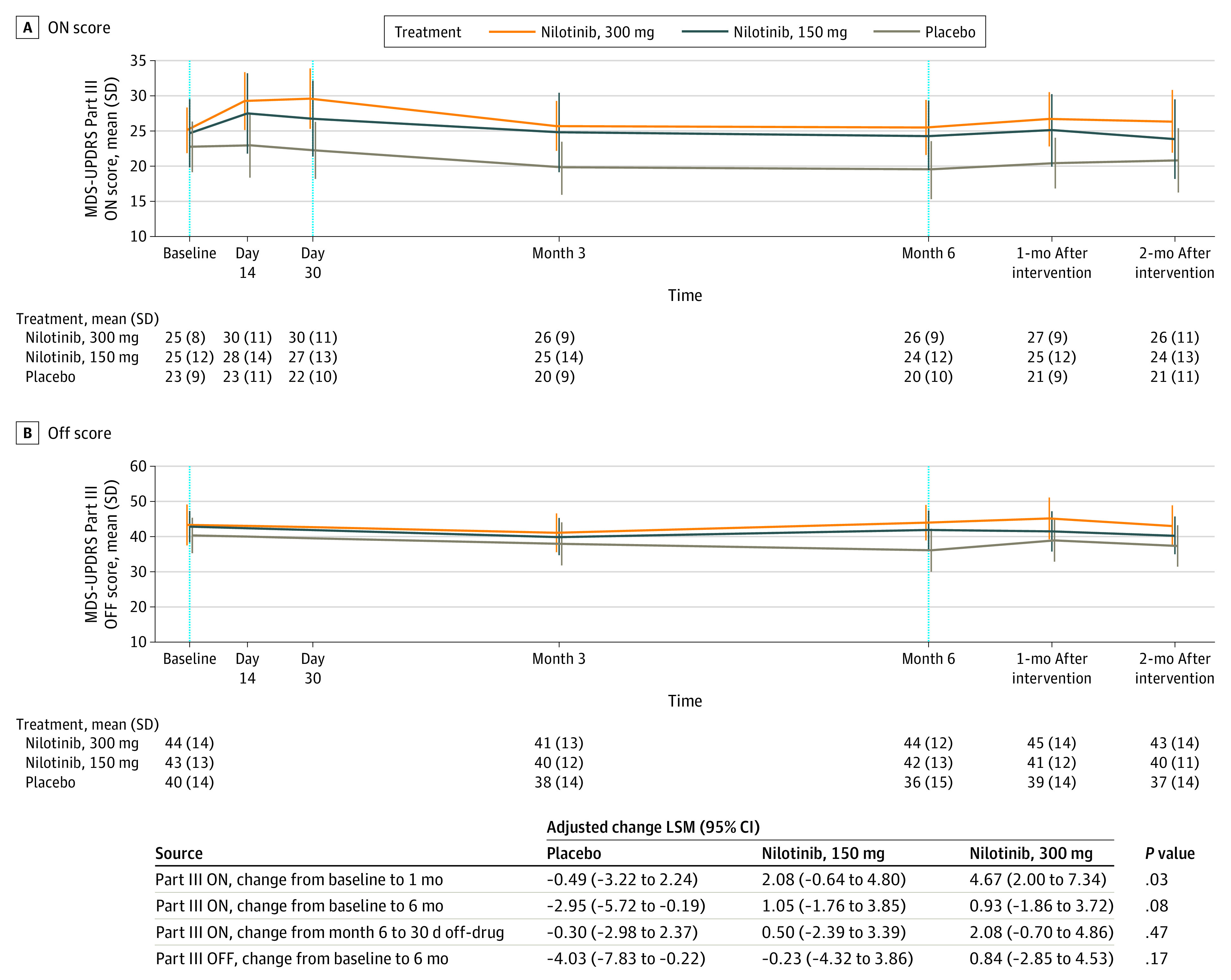
Movement Disorder Society Unified Parkinson’s Disease Rating Scale (MDS-UPDRS) Part III ON and OFF Scores Over Time

Change of MDS-UPDRS Part III in the medications on-state and off-state are presented in Figure 2. Both analyses demonstrate “futility” from the previously observed large symptomatic effect^7^ (Figure 2). The analysis demonstrated not only futility but actually a trend toward worsening in the active treatment arms. Nilotinib, 150 mg and 300 mg, exhibited worse motor scores in the medication on-state compared with placebo, achieving significance for nilotinib, 300 mg, at month 1 (Figure 2) but not at other points. There was no significant difference between the groups in the change of MDS-UPDRS Part III in the medications off-state between baseline and month 6 (Figure 2). No significant differences were observed for any other exploratory clinical outcomes (eTable 1 in Supplement 3).

### Exploratory Pharmacokinetic and Biomarker Outcomes

Nilotinib serum and CSF concentrations at month 3 are presented in eTable 2 in Supplement 3. Total serum concentrations were within the range of previously reported values.^12^ Nilotinib CSF concentrations were 0.19% and 0.26% of those in the serum for the 150-mg and 300-mg doses, respectively. The absolute concentrations observed at approximately T maximum were only 8% to 13% of the reported cellular half-maximal inhibitory concentration of 20nM (11 ng/mL) for inhibition of c-Abl by nilotinib.^13^

**Figure 3.**
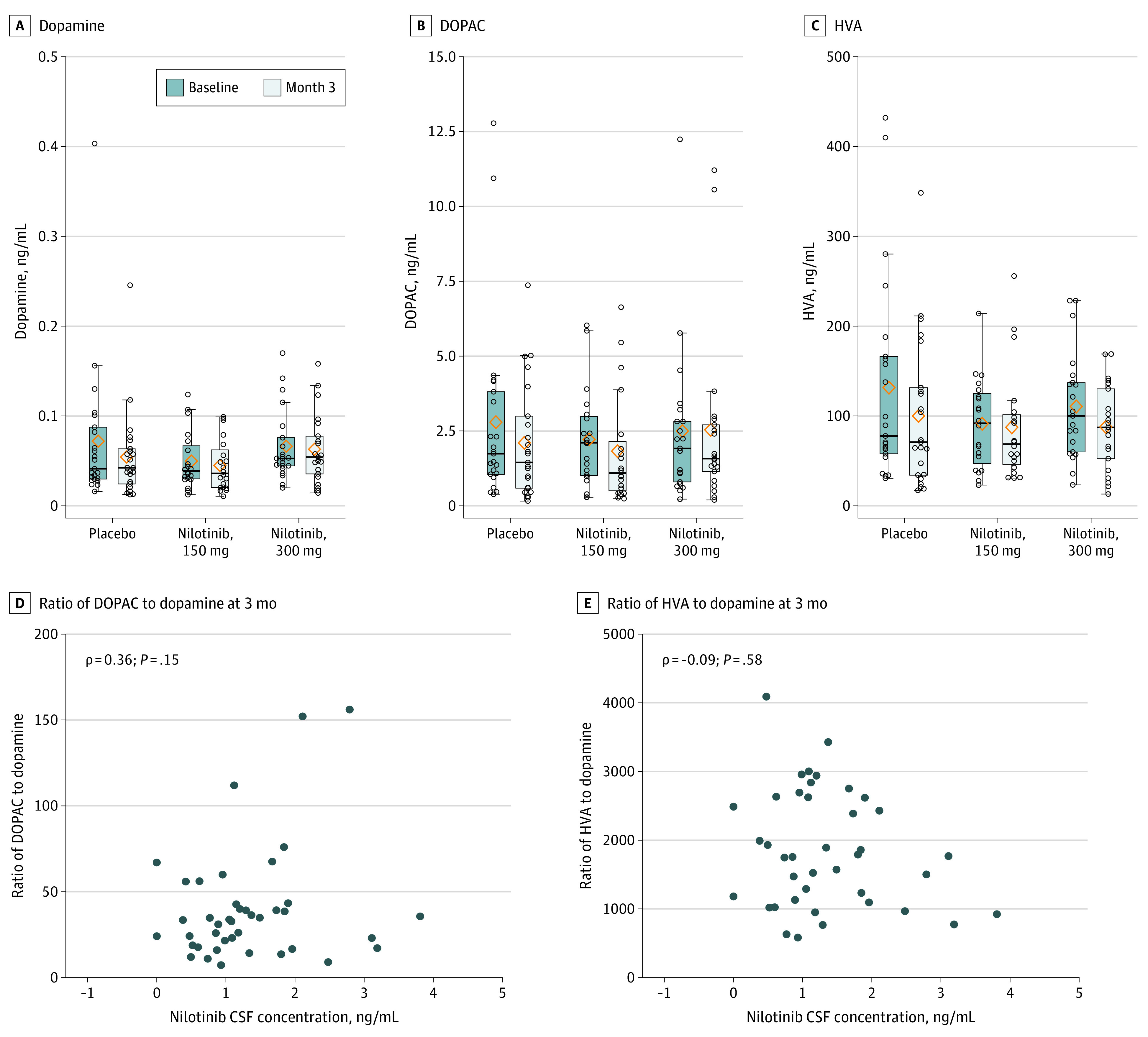
Cerebrospinal Fluid (CSF) Levels of Dopamine, Its Metabolites, and the Relationship Between Nilotinib CSF Concentrations and Dopamine Turnover Indices DOPAC indicates 3,4-dihydroxyphenylacetic acid; HVA, homovanillic acid.

Three-month nilotinib treatment did not alter CSF levels of dopamine or its metabolites measured in samples collected at approximate T maximum mean (SD) of 2 (0.5) hours after receiving a dose (Figure 3A-C). Results remained the same even after excluding participants receiving concurrent MAO-B treatment (eTable 3 in Supplement 3). Furthermore, there was no correlation between CSF nilotinib levels and levels of dopamine metabolites or their ratios with dopamine (Figure 3D and E).

## Discussion

In line with data published in 2020 by Pagan et al,^8^ our study demonstrates acceptable safety and tolerability of both tested doses of nilotinib in participants with moderately advanced PD. However, similar to the Pagan et al^8^ cohort, it has to be highlighted that these tolerability outcomes apply to participants selected based on stringent inclusion/exclusion criteria and are not generalizable to the PD population at large because the study excluded participants with comorbid conditions that increase the likelihood of nilotinib-related AEs. As such, the study had higher than usual screen failure rate of 39%, which was also seen in Pagan et al study at 25%.^8^ In addition, while not significant, there was a trend for a higher rate of drug suspensions and discontinuations in the nilotinib arms vs placebo. We did not observe a significant difference in AEs between the active and placebo groups. Nilotinib has a black box warning regarding increased risk of ventricular arrhythmia. While the only SAE of cardiac arrhythmia did occur in nilotinib 300-mg group, that event was a new-onset atrial fibrillation that occurred within 24 hours from the first study drug administration. So while classified as possibly treatment related, causality remains to be determined and might be less relevant to the mechanism of potential cardiac toxicity of the drug. We did not observe any negative effects on hematologic parameters likely owing to the fact that we excluded any participants with underlying hematologic risk factors and used lower nilotinib doses compared to ones used for leukemia. While AEs were common, most were related to asymptomatic elevation of liver and pancreatic enzymes which are expected based on nilotinib mechanism of action. Similar to Pagan et al,^8^ falls were the most common AE. However, in our study, the placebo arm had a significantly higher incidence compared with active arms, pointing to disease heterogeneity rather than drug effect.

In regard to the secondary efficacy analysis, we did not observe any positive effect of either dose of nilotinib on PD disability and as such failed to reproduce the previously reported symptomatic effect on motor and cognitive function in an open-label study.^7^ We also did not observe symptomatic benefit of either dose of nilotinib on overall PD disability (MDS-UPDRS total, OFF and ON scores, severity of motor complications [MDS-UPDRS Part IV], cognition, sleep, or quality of life). On the contrary, motor scores were generally worse for both nilotinib groups compared with placebo, achieving significance for nilotinib, 300 mg, at month 1 in the medication’s on-state. Our results regarding safety, tolerability, and lack of the symptomatic effect of nilotinib are in line with the study by Pagan et al.^8^ In aggregate, results of both studies highlight limitations of open-label studies of symptomatic effects in PD.

The major goal of this study was to inform whether the results justified further testing of nilotinib in PD. Our prespecified “go/no-go” criteria included a constellation of the safety/tolerability, which if not met would trigger an absolute no-go. With acceptable safety and tolerability, a go decision required considerations of secondary and exploratory outcomes. The assessment of the pharmacokinetic profile of nilotinib and associated biomarker changes, while exploratory, is critical for interpretation of study results, especially because at the launch of this study there were limited data on the CSF penetration of the drug.^7^ The observed serum and CSF concentrations show the CSF to serum ratio of 0.19% to 0.26%, in line with the report in a similar PD cohort^8^ as well as previous PK studies.^12^ Importantly, the CSF concentration is one-tenth of the reported cellular potency of nilotinib for c-Abl inhibition,^13^ indicating that at doses within the safe therapeutic range, there would be minimal c-Abl inhibition in the brain that was reported previously.^7^ We failed to replicate the previously reported c-Abl inhibition in the CSF^7^ using the identical assay because neither c-Abl nor its phosphorylated form were detectable in the CSF. The low brain penetration of nilotinib in this study is consistent with the CSF and brain PK profile in our dog study (eAppendix in Supplement 3), where we could not observe brain c-Abl inhibition despite somewhat higher concentrations of nilotinib in the CSF. Whether nilotinib could exert pharmacologic effects through other mechanisms remains to be seen.

As a measure of downstream and reported functional effects of nilotinib on dopaminergic neurons, we measured dopamine, dopamine metabolites, and additional monoamines in the CSF. Unlike previous reports,^7,8,15^ we did not observe a change in any biomarker, nor a correlation between CSF nilotinib exposure and dopamine, dopamine metabolites, or dopamine turnover. Because concurrent treatment with PD dopaminergic therapies, especially MAO-B inhibitors, can affect dopamine metabolites^16^ and thereby confound the results, our analysis controlled for the dose and change in the dopaminergic therapy. In addition, a subgroup analysis excluding participants on MAO-B inhibitors did not change the conclusion. This is not entirely surprising because previous reports by Pagan et al^7,8,15^ show highly variable results within the cohort as well as between studies. For example, in the open-label study,^7^ CSF homovanillic acid levels increased significantly in the 150-mg nilotinib group at 2 months but not at 6 months, whereas in the 300-mg group, homovanillic acid levels increased at 6 months only (note that 3,4-dihydroxyphenylacetic acid results were not included in the report). In contrast, the 2020 double-blind, placebo-controlled study reported significantly higher homovanillic acid levels in the CSF in the 150-mg nilotinib group but not in the 300-mg group at 12 months.^8^ Similar concerns regarding interpretation of the biomarker data were expressed in the Editorial^17^ that accompanied Pagan et al publication.

In light of the low CSF exposures and lack of effects on dopamine metabolites, we concluded that the plausibility of observing changes in proteinopathy biomarkers in the CSF, such as α-synuclein, phospho-α-synuclein, or phospho-tau, is too low to warrant additional analyses, although the biospecimens are available for future studies. In this regard, it is noteworthy that Pagan et al^8^ suggest their observations of nilotinib-induced increase in 3,4-dihydroxyphenylacetic acid levels may result from a reduction in oligomeric α-synuclein. However, they reported an increase in CSF 3,4-dihydroxyphenylacetic acid levels after a single dose of 200-mg (but not a 150-mg or 300-mg dose) nilotinib in the absence of effects on oligomeric α-synuclein levels in the same group.^15^ Regardless, their single-dose biomarker data and the congruency in the CSF exposures between the 2 studies indicate that our failure to detect dopamine metabolites changes at 3 months is a reflection of the lack of robust and reproducible effects of nilotinib on brain dopaminergic system at doses tested.

### Strengths and Limitations

The strength of our study is the multicenter design and rigorous implementation based on highly standardized protocols for clinical outcomes, PK, and biomarker data collection and analysis. The study has a number of limitations. First, it was conducted in moderately advanced PD, which is not the population typically targeted for PD disease-modifying interventions. The rationale for selecting that stage of PD was driven by the need to test for the large symptomatic effect reported previously^7^ as well as to assess safety and tolerability in a more vulnerable PD population. Although the study protocol included a provision to proceed with recruitment of a de novo PD cohort if the results supported further nilotinib development, the data in aggregate did not support a go decision. Although the doses of nilotinib tested here are in the lower range of approved doses, testing nilotinib at higher doses to increase brain exposure is not advisable owing to the safety profile of nilotinib.

## Conclusions

In conclusion, while we demonstrated acceptable safety and tolerability of nilotinib in our cohort, the low CSF exposure and lack of biomarkers effects combined with the efficacy data trending in the negative direction led us to conclude that nilotinib is not suitable for further testing in PD. These results do not refute the hypothesis that c-Abl inhibition is a potentially important therapeutic target for PD disease-modification interventions. Indeed, there are a number of novel molecules targeting c-Abl pathway in development that have a better therapeutic profile.^18^ While results of these studies are highly anticipated, ideally, next-generation studies will select participants based on the demonstration of susceptibility to the targeted mechanism of action, which will require further biomarker development.

## Notes

Supplement 1. Trial protocol

Supplement 2. Statistical analysis plan

Supplement 3. **eMethods.** Analytical methods for assessment of monoamines and their metabolites in the CSF and to determine nilotinib concentrations in the serum and CSF **eTable 1. **Changes in the exploratory outcomes over time **eTable 2. **Steady state nilotinib serum and csf concentrations at 3 mon around tmax (2 +/-0.5 hr) **eTable 3. **CSF levels of dopamine, its metabolites by treatment group excluding participants on MAO-B inhibitors **eAppendix. **Serum and CSF pharmacokinetic study in the dog

Supplement 4. Nonauthor Collaborators. Parkinson Study Group NILO-PD Investigators

Supplement 5. Data sharing statement
