# Supplementary material for: Efficacy of Nilotinib in Patients With Moderately Advanced Parkinson Disease: A Randomized Clinical Trial
Source: JAMA Neurol. 2020 Dec 14;78(3):1–9. doi: 10.1001/jamaneurol.2020.4725 (PMC7737147; doi:10.1001/jamaneurol.2020.4725)
Supplement: Supplement 1. — Trial protocol [file jamaneurol-e204725-s001.pdf]

CLINICAL STUDY PROTOCOL

Title:	A Randomized, Double-Blind, Placebo-Controlled, Phase IIa, Parallel Group, Two-Cohort Study to Define the Safety, Tolerability, Clinical and Exploratory Biological Activity of the Chronic Administration of Nilotinib in Participants with Parkinson's Disease (PD)

Acronym:	NILO-PD
IND#:	134973
Version:	4
Clinical Phase:	IIa
Sponsor:	Northwestern University Feinberg School of Medicine

Principal Investigator:	Tanya Simuni, MD<br>Professor of Neurology<br>Director, Parkinson's Disease and Movement Disorders Center<br>Northwestern University Feinberg School of Medicine<br>Department of Neurology, Abbott Hall, 11th Floor<br>710 North Lake Shore Drive<br>Chicago, Illinois 60611

Funding:	Michael J. Fox Foundation for Parkinson's Research
Supported by:	Parkinson Study Group

Clinical Coordination Center:	Clinical Trials Coordinating Center, Rochester, NY
Data Coordination Center:	Clinical Trials Coordinating Center, Rochester, NY
Biostatistics Coordination Center:	Clinical Trials Statistical & Data Management Center, Iowa City, Iowa

CONFIDENTIAL

This document is a confidential communication. The recipient agrees that no information contained herein will be published or disclosed without written approval. This document may be disclosed to appropriate institutional review boards, independent ethics committees, or duly authorized representatives of the U.S. Food & Drug Administration or other regulatory authorities as appropriate; under the condition that confidentiality is requested.

PROTOCOL APPROVAL

Amendment 1

STUDY TITLE: A Randomized, Double-Blind, Placebo-Controlled, Phase IIa, Parallel Group, Two-Cohort Study to Define the Safety, Tolerability, Clinical and Exploratory Biological Activity of the Chronic Administration of Nilotinib in Participants with Parkinson's Disease (PD)

STUDY ACRONYM: NILO-PD

3-14-2018

Tanya Simuni, MD
Principal Investigator
Date

3/14/18

Christopher S. Coffey, PhD
Biostatistician
Date

15-MAR-18

Cynthia Casaceli, MBA
Clinical Trials Coordination Center Administrative Principal Investigator
Date

INVESTIGATOR AGREEMENT

Amendment 1

STUDY ACRONYM: NILO-PD
IND# 134973
Protocol Version: 4

I have carefully read this protocol, including all appendices, and the nilotinib package insert, and agree that it contains all the necessary information for conducting the study safely.

I will conduct this study in strict accordance with this protocol and according to the current Good Clinical Practice (GCP) regulations and guidelines [21 CFR (Code of Federal Regulations) Parts 11, 50, 54 and 56 and ICH (International Conference on Harmonization of Technical Requirements for Registration of Pharmaceuticals for Human Use) Topic E6 (R2)], and local regulatory requirements. Any changes in procedure will only be made if necessary to eliminate immediate hazards and/or to protect the safety, rights or welfare of participants.

I will provide copies of the protocol and all other information relating to the pre-clinical and prior clinical experience, which were furnished to me, to all physicians and other study personnel responsible to me who participate in this study. I will discuss this information with them to assure that they are adequately informed regarding the study drug and conduct of the study.

I will ensure that the drugs supplied to me for this study will be used only for administration to participants enrolled in this study protocol and for no other purpose.

I agree to keep records on all participant information (case report forms, informed consent statements, drug shipment, drug return forms, and all other information collected during the study) in accordance with the current GCP, local and national regulations.

_______________ ____
Site Number Printed Site Name

____
Printed Site Investigator Name

____ ___________________
Site Investigator Signature Date

DOCUMENT HISTORY

Document Version	Date of Issue	Summary of Changes
1.0	05 May 2017	n/a
2.0	15 Jun 2017	• Remove renal impairment as exclusion criteria<br>• Addition of tobacco and caffine exposure questionnaire at baseline visit<br>• Treatment expectation questionnaire added to SV1<br>• Editorial updates
3.0	14 Jul 2017	• Move Diagnostic Features assessment from Baseline to Screening Visit 1<br>• Move C-SSRS from Baseline to Screening Visit 1<br>• Clarify MDS-UPDRS OFF assessment in SOA (Cohort 1 and Cohort 2)<br>• Editorial Changes
4.0	21 Feb 2018	**Section 4.2 Inclusion Criteria**<br>• Cohort 1 inclusion criteria 8a revised to allow treatment with monoamine oxidase B (MAO-B) inhibitors provided the dose has been stable for 60 days prior to baseline.<br>**Section 4.3 Exclusion Criteria**<br>• Added time frame (5yrs) for suicidal history and active suicidal ideations.<br>• Excluded participants with history uncontrolled electrolytes (hypokalemia or hypomagnesaemia) and significant lab abnormalities. Clarification added to the exclusion criteia on lab abnormalities (to align with dose reduction rules (table 2).

		• Exclusion Criteria #9 clarified to include specific parameters for exclusion of first degree AV block.<br>• History of bone marrow suppression or evidence of persistent myelosuppression added to exclusion criteria to align the protocol language with the Dose Adjustment Table.<br>• Definition of abnormal pancreatic funciton added- exclusion criteria 14.<br>• Added time frame (≤ 5 years) for history of alcohol abuse<br>• Patients treated with MAO-Bs removed from exclusion criteria.<br>**Section 4.4.1 Warnings/Precautions**<br>• Musculoskeletal Symptoms after Discontinuation added based on revised Tasigna Package Insert (12/2017).<br>**Section 5.1 Recruitment**<br>• Previous screen failures under protocol version 14Jul2017 allowed to rescreen if deemed eligbile based on Amendment 1 inclusion/exclusion criteria.<br>**Section 6. Schedule of Activities**<br>• Clarification provided - if PD medication is increased, MDS-UPDRS ON assessment is required at next study visit, even if not required per visit SOA.<br>• Section 9 moved up and combined with Section 6 to contain all protocol details related to the schedule of activities (all now contained in section 6). The changes/new language is highlighted in new section 6 of the revised protocol.<br>• Rescreening visit (section 6.2.2): +/- 7 day window added to rescreening visit.<br>• Added a 10 day window around the coagulation parameters for V03 and PW. Use of local labs allowed (STAT results)

		for coagulation parameters, to accomodate for patrticipants who are unable to return to clinic 10 days prior to visit.<br>• Table 2 – dose reduction/suspension revised: reference ranges have been added/revised.<br>**Appendix 1**<br>• December 2017 Tasigna Package Inserted<br>**Editorial Revisions Throughout**

PROTOCOL SYNOPSIS

Protocol Title	A Randomized, Double-Blind, Placebo-Controlled, Phase IIa, Parallel Group, Two-Cohort Study to Define the Safety, Tolerability, Clinical and Exploratory Biological Activity of the Chronic Administration of Nilotinib in Participants with Parkinson's Disease (PD)
Acronym/Title	NILO-PD
Clinical Phase	Phase Iia
Site Investigators	PSG Site Investigators
Study Centers	25 sites located in the US
Study Period	<u>Planned enrollment duration</u>: approximately six (6) months for each cohort.<br><u>Duration of post randomization follow up:</u><br>• Cohort 1 (moderate to advanced PD): Six (6) months<br>• Cohort 2 (early/de novo PD): Twelve (12) months
N Participants	• Cohort 1: N=75 randomized 1:1:1 to once daily dose of nilotinib or placebo (150mg: 300mg : placebo)<br>• Cohort 2: N=60 randomized 2:1 to once daily dose of nilotinib or placebo (the selected dose from cohort 1: placebo). Randomization in this cohort will not commence unless a safe & tolerable dose is determined from Cohort 1.
Study Rationale	A small number of cell and animal models suggest that nilotinib may positively affect the alpha (α) -synuclein pathology observed in PD[1-8]. A small open-label clinical study that lacked a placebo control group tested the safety and tolerability of nilotinib in PD and dementia with Lewy body (DLB) patients for the first time and explored its efficacy [9]. Although preliminary data appear promising, the study design precludes firm conclusions about safety, tolerability, optimal dose or efficacy[10]. Nilotinib is approved by the Food and Drug Administration (FDA) for certain types of leukemia but not for PD. **An opportunity exists to rigorously establish the safety, tolerability as well as optimal dose, of nilotinib to enable future definitive efficacy studies in PD patients.**
Primary Study Objective	To assess the safety and tolerability of the daily oral administration of nilotinib (150-300mg once daily) in moderate/advanced and early/de novo PD participants.
Key Secondary Objective (Moderate/Advanced Cohort)	To conduct a futility analysis within each treated group in Cohort I. This futility analysis will compare the observed change in MDS-UPDRS part III between baseline and 6 months to the observed change from the clintrials.gov NCT02281474 nilotinib study in order to determine whether we can rule out the large change previously reported – i.e., declare "futility".
Secondary Objectives	• To establish the degree of symptomatic effect of nilotinib as measured by the change in MDS-UPDRS part III between 1) baseline and Visit 2 (1 month) and 2) final visit on study drug and 30 days off study drug in cohorts 1 and 2.

	• To explore the impact of nilotinib on progression of PD disability as measured by the change in the MDS-UPDRS Part III in the defined medications OFF state in cohort 1 (moderate/advanced PD) between baseline and 6 months.<br>• To establish the impact of nilotinib on progression of PD disability as measured by the change in the MDS-UPDRS Part III score in Cohort 2 (early/de novo PD) between baseline and 12 months or time of initiation of symptomatic therapy (ST), whichever comes first.
Exploratory	**<u>Clinical Measures</u>**<br>The change in the following measures of disability, quality of life and functional status from baseline to final visit will be analyzed:<br>*Motor disability*<br>• The change in MDS-UPDRS total score. For participants on ST, MDS-UPDRS part III assessments will be collected in the defined medications OFF (approximately 12 hours post dose) and ON (based on the participant/site investigator defined best ON and/or approximately 1 hour after dose of ST) state<br>• The change in the ambulatory capacity (sum of 5 MDS-UPDRS questions:(falling, freezing, walking, gait, postural stability)<br>• Analysis of ST utilization as measured by levodopa equivalence dose<br>• Severity of motor complications as measured by MDS-UPDRS IV subscale (complications of therapy)<br><br>*Cognitive disability*<br>• The change in the cognitive function as measured by the change in Mattis Dementia Rating Scale – 2 (DRS-2)<br><br>*Sleep function*<br>• The change in sleep quality as measured by the Parkinson's Disease Sleep Scale (PDSS)<br><br>*Measures of global disability*<br>• Clinician and Participant Global Impression of Change Score (CGI)<br><br>*Measures of functional status and quality of life*<br>• The change in the MDS-UPDRS Motor and Non-Motor Experiences of Daily Living subscores<br>• The change in the modified Schwab and England scale (S&E)<br>• The change in Parkinson Disease Quality of Life Questionnaire 39 (PDQ-39)<br>• The change in European Quality of Life Scale (EQ-5D)

	Blindedness and Treatment Expectations assessments<br>• Questionnaires completed by the Site Investigator and Participant<br><br>**Pharmacology and Biomarkers**<br>Pharmacokinetics (PK) and Pharmacodynamics:<br>o To determine the serum PK of nilotinib in patients with PD.<br>o To investigate the ability of nilotinib to cross the blood brain barrier in patients at a dose(s) that is tolerable and safe as measured by dose-dependent increases in unbound nilotinib concentrations in the cerebrospinal fluid (CSF).<br>o To investigate factors that could affect the PK of nilotinib such as age, sex, PD severity, race/ethnicity.<br>o To investigate the ability of nilotinib to engage its known molecular target(s) at a dose that is tolerable and safe as measured by biomarkers of pathway inhibition in the serum and CSF (including but not limited to reduction in phospho-cAbl tyrosine kinase).<br>o To investigate the relationship between nilotinib exposures in serum and CSF with its ability to engage known molecular target(s) [measured by biomarkers of pathway inhibition in the serum and CSF (including but not limited to reduction in phosphor-cAbl).<br>To explore impact of nilotinib on:<br>o Dopaminergic system as measured by an increase in dopamine and its metabolites in CSF (e.g., homovanilic acid (HVA) level)<br>o Other biomarkers linked to potential mechanism of neuroprotection (including but not limited to CSF α -synuclein, total tau, phosphorylated -tau, etc.)<br>Store additional blood/serum/plasma/DNA/CSF samples for future research.
Study Population	**Cohort 1**: Participants with moderate/advanced PD (see inclusion and exclusion criteria)<br>**Cohort 2**: Participants with early untreated PD (see inclusion and exclusion criteria)
Study Design	This is a Phase IIa, multicenter, randomized, double-blind, placebo-controlled, parallel group study of nilotinib in participants with moderate to advanced (Cohort 1) and early/de novo (Cohort 2) PD. The study will define the safety, tolerability, and exploratory clinical and biological activity of the chronic administration of nilotinib in participants with PD.<br><br>**Cohort 1** will treat participants with moderate/advanced PD. Participants will be randomized in a 1:1:1 ratio into one of the following treatment arms:<br>• nilotinib 150mg<br>• nilotinib 300mg<br>• matching placebo

	Study duration will be approximately 8.5 months from screening to post drug evaluation; treatment period from randomization to final on treatment visit will be 6 months.<br><br>A primary outcome analysis for safety and tolerability will be conducted after 6 months of treatment. The results will determine whether either of the doses are sufficiently safe and tolerated to justify moving forward with Cohort 2.<br><br>**<u>Cohort 2</u>** will treat early/de novo PD participants. Participants will be randomized in a 2:1 ratio as follows:<br>• nilotinib – selected dose, based on safety and tolerability in Cohort 1. or<br>• matching placebo<br><br>Study duration will be approximately 14.5 months from screening to post drug evaluation; treatment period from randomization to final on study drug visit will be 12 months.<br><br>Participants in both Cohorts will be dosed orally once daily (QD) in a fasted state (no food consumed for at least 2 hours before and at least one hour after dosing).
Number of Participants	The combined two cohorts will screen approximately one hundred eighty (180) participants in order to randomize a total of approximately one hundred thirty-five (135) participants. Approximately seventy-five (75) participants will be randomized into Cohort 1 and approximately sixty (60) participants will be randomized into Cohort 2.
Main Inclusion Criteria	**Cohort 1 and 2**<br>1. Participants with idiopathic PD based on the UK Brain Bank diagnostic criteria.<br>2. Participants of any race and either gender, age 40-79 on the date the informed consent form (ICF) is signed and with the capacity to provide voluntary informed consent.<br>3. Participants able to read and understand English and the ICF and are willing to comply with all study procedures, treatment and follow-up.<br>4. Participants must be willing to undergo multiple lumbar punctures (LP) as outlined in the schedule of activities.<br>5. Participants who are taking any central nervous system acting medications (e.g., benzodiazepines, antidepressants, hypnotics) must be on a stable regimen for at least 30 days prior to the baseline visit.<br><br>**Inclusion criteria specific for Cohort 1**<br>6a. Participants with a diagnosis of PD duration > 5 years.

	7a. Participants with Hoehn & Yahr scale (H&Y) stage > 2 and < 4 in the ON state.<br>8a. Participants on a stable regimen of PD medications, that includes levodopa, for at least 30 days prior to the screening visit.<br>a. Treatment with monoamine oxidase B (MAO-B) inhibitors will be allowed provided the dose has been stable for 60 days prior to baseline.<br><br>**Inclusion criteria specific for Cohort 2**<br>6b. Participants with a diagnosis of PD duration < 3 years.<br>7b. Participants with H&Y stage ≤ 2.<br>8b. Participants who are currently NOT receiving ST (levodopa, dopamine agonists and monoamine oxidase B (MAO-B) inhibitors) and NOT projected to require ST for at least 3 months from enrollment.<br>a. Treatment with amantadine or an anticholinergic agent will be allowed provided the dose has been stable for 30 days prior to screening and will remain stable for the duration of the study.
Main Exclusion Criteria	**Cohorts 1 and 2**<br>1. Participants with a diagnosis of atypical parkinsonism.<br>2. Participants with a history of bipolar disorder or major depression, or the presence of active depression defined as a Beck Depression Inventory II (BDI-II) score >17.<br>3. Participants with a history of a suicide attempt within the last 5 years or active suicidal ideations<br>4. Participants with a history of schizophrenia or schizophrenia spectrum disorders<br>5. Participants with a history of uncontrolled hypokalemia or hypomagnesaemia, or laboratory evidence of such on screening<br>6. Participants with evidence of other significant laboratory abnormalities which in the opinion of the site investigator or clinical monitor should preclude study participation<br>7. Participants with history of cardiac arrhythmia, long QT syndrome, or a corrected QT interval (QTcF) ≥450ms at screening visit 1<br>8. Participants treated within 30 days prior to randomization, or planned use during the trial with any of the following classes of Concomitant drugs (see full lise in Appendix 2):<br>a. Class IA or III antiarrhythmic drugs<br>b. QT prolonging drugs<br>c. Strong CYP3A4 inhibitors or inducers<br>d. Anticoagulants<br>e. Proton pump inhibitors<br>9. Participants with a clinical history, or the active presence of a

	cardiovascular condition including:<br>a. Myocardial infarction, known cardiac ischemia, or angina<br>b. Cerebrovascular event (e.g. embolic stroke)<br>c. Congestive heart failure, symptomatic first degree atrioventricular (AV) block or PR interval >220msec and all second and third degree AV block, sick sinus syndrome, or other serious cardiac rhythm disturbances<br>d. Any history of Torsade de Pointes<br>e. Any other cardiovascular history that, in the opinion of the Site Investigator, will preclude study participation<br>10. Participants with a history of hepatic disease, including abnormal liver function defined as Total Bilirubin > 1.5 times upper limit of normal, Aspartate Aminotransferase (AST) and/or Alanine Aminotransferase (ALT) > 2 times the upper limit of the normal, or coagulopathy with INR > 1.4<br>11. Participants with a history of bone marrow suppression or evidence of persistent myelosuppression defined as absolute neutrophil count <1.8 X 10^9/L, significant anemia, or thrombocytopenia defined as platelet count < 100 X 10^9/L<br>12. Participants with a prior history of epilepsy or a seizure within the last 6 months<br>13. Participants with an active malignancy, or history of a neoplasm in the prior 5 years (excluding basal/squamous cell carcinoma)<br>14. Participants with a prior history of pancreatitis, total gastrectomy or evidence of abnormal pancreatic function defined as elevated amylase and/or lipase > 2 times upper limit of normal<br>15. Participants diagnosed with human immunodeficiency virus **(**HIV), clinically significant chronic hepatitis such as hepatitis B (HBV) or hepatitis C (HCV), or clinical history or signs of an active infection<br>16. Participants with a history of drug or alcohol abuse ≤ 5 years<br>17. Participants who have an active medical or psychiatric condition that in the opinion of the Site Investigator should preclude study participation<br>18. Participants with previous surgical management for Parkinson's<br>19. Participants participating in any drug or device clinical investigation concurrently or within 30 days prior to screening for this study<br>20. Participants with severe lactose and galactose intolerance<br>21. Participants with a known hypersensitivity or contraindication to study drugs (nilotinib or matching placebo) or their components.<br>22. Female participants of child-bearing potential. Female participants must be post-menopausal, post-hysterectomy, or have a documented infertility based on a known medical or surgical condition

	**Exclusion criteria specific for Cohort 1**<br>23a. Participants with a diagnosis of dementia based on the clinician's assessment, or a Montreal Cognitive Assessment (MoCA ©) score < 21 at baseline<br><br>**Exclusion criteria specific for Cohort 2**<br>23b. Participants with a MoCA score < 26 at baseline<br>24b. Participants treated within 60 days prior to randomization or expected to require treatment within 3 months from randomization with any ST (including levodopa, dopamine agonists and MAO-B inhibitors)<br>a. Treatment with amantadine or an anticholinergic agent will be allowed provided the dose has been stable for 30 days prior to screening and will remain stable for the duration of the study
Route and Dosage Form	Nilotinib versus matching placebo administered orally QD
Dosage	Nilotinib 150mg, 300mg and matching placebo
Duration of Treatment	Cohort 1 = 6 months<br>Cohort 2 = 12 months
Primary Outcome Measure(s)	To establish the safety and tolerability of nilotinib (150mg and 300mg orally QD) versus placebo in PD patients (moderate/advanced & early/de novo)<br>• Tolerability will be judged by the proportion of participants enrolled in a dosage group able to complete the study on their originally assigned dosage. Tolerability of each active arm will be compared to placebo group.<br>Safety will be primarily assessed based on a direct comparison of treatment-related serious adverse events across the treatment groups.
Sample Size Considerations	We assumed at least 90% of participants on placebo will meet definition of "tolerability" and determined required sample size to detect an absolute difference of 30% with respect to tolerability for active treatment arms versus placebo (in other words if 60% or fewer participants on an active treatment arm meet the stated definition of tolerability). Under this assumption and considering a one-sided test with alpha = 0.05, a total of 25 participants per group provides 80% power for the moderate/advanced cohort. Similarly, a sample size of 40 participants on the selected dose and 20 participants on placebo for the early/de novo second cohort provides similar power. With three groups in the moderate/advanced initial cohort, and two groups in the early/de novo second cohort, this leads to a total sample size of 135 participants.<br><br>For the major secondary objective, based on results from Table 2 in the Pagan et al[9] publication, the mean change over 6 months in the 150mg

	group was 7.0 – with a standard deviation of 12.9. Using these estimates, we computed the power of the currently proposed study to detect "futility" based on comparisons of the change within the dosage groups themselves. Converting from the UPDRS used in the Pagan et al study[9], to the MDS-UPDRS proposed for this study, we will test the following hypothesis in the 150mg group: $H_0: \delta \leq -9.8$ (7 x 1.4) vs. $H_A: \delta > -9.8$ Since this is an early phase exploratory study, we assume an alpha level of 0.10 for this one-sided hypothesis test. Based on these assumptions, if the observed reduction is half that observed in the prior study (-3.5), then the study has marginal power (52%) to declare futility. If there is no observed reduction associated with treatment, the study has 92% power to declare futility. Thus, this test of futility will have adequate power mainly if the direction of effect is in the opposite direction from that observed in the previous study (i.e., if there is no reduction, only a potentially smaller increase in MDS-UPDRS compared to placebo). Since bigger effects were observed over 6 months for the 300mg group, the study has adequate power for addressing futility in the 300mg group in a similar manner.

SCHEDULE OF ACTIVITIES - Cohort 1

**Cohort 1**	**Screening**		**Baseline**	**Titration Phase**				**Maintenance Period**			**Final visit On drug**	**Post-Drug Evaluation**		**Unsched Visit**	**Premature W/D**[b]
	**SC 1**<br>**Day -30-14d**	**SC 2**<br>**Day -14-7d**	**Visit BL**<br>**Day 0**	**SV 1**<br>**Day 7±3d**	**V01**<br>**Day 14±3d**	**V02**<br>**Day 30±3d**	**SV 2**<br>**Day 60±7d**	**V03 3mo Day 90±7d**	**SV 3**<br>**Day 120±7d**	**SV 4**<br>**Day 150± 7d**	**V04 6mo Day 180±7d**	**V05 1 mo post +30 ±7d post**	**V06 (FNL) 2 mo post +60 ±7d post**	**(U01, U02, etc.)**	**PW Visit**
**Informed Consent**	X														
**Participant Entry Numbers**	X														
**Eligibility Criteria**	X		X												
**Demography**	X														
**Socio-economic form**	X														
**Med/Neuro History**	X														
**MoCA**	X														
**Beck Depression Inventory (BDI) – II**	X														
**Vital Signs**	X		X	X	X	X	X	X	X	X	X	X	X	X	X
**Height, weight**	X							X			X	X			X
**Physical Exam**	X		X					X			X	X			X
**Neurological exam**	X										X				X
**Fundoscopy exam**	X							X				X			X
**Hoehn & Yahr Scale**	X							X			X	X	X		X
**MDS-UPDRS I-IV ON** [#]			X		X	X		X			X	X	X		X
**MDS-UPDRS III OFF**			X					X			X	X	X		X
**Modified Schwab & England scale**			X					X			X				X
**PD diagnostic features**	X										X				X
**Primary Dx form**	X										X				X
**Mattis Dementia Scale - 2**			X					X[d]			X				Xd
**PDQ-39**			X					X			X				X
**EQ-5D**			X					X			X				X
**CGI I and P**			X					X			X				X
**C-SSRS**	X							X			X		X	X	X
**PDSS**			X					X			X				X

**Cohort 1**	**Screening**		**Baseline**	**Titration Phase**				**Maintenance Period**			**Final visit On drug**	**Post-Drug Evaluation**		**Unsched Visit**	**Premature W/D**[b]
	**SC 1**<br>**Day -30-14d**	**SC 2**<br>**Day -14-7d**	**Visit BL**<br>**Day 0**	**SV 1**<br>**Day 7±3d**	**V01**<br>**Day 14±3d**	**V02**<br>**Day 30±3d**	**SV 2**<br>**Day 60±7d**	**V03 3mo**<br>**Day 90±7d**	**SV 3**<br>**Day 120±7d**	**SV 4**<br>**Day 150± 7d**	**V04 6mo**<br>**Day 180±7d**	**V05 1 mo post +30 ±7d post**	**V06 (FNL) 2 mo post +60 ±7d post**	**(U01, U02, etc.)**	**PW Visit**
**Tobacco & Caffeine Q**			X												
**Blindedness Q**											X	X			X
**Tx expectation Q**	X		X								X	X	X		X
**ECG-12 LEAD**	X		X	X		X	X	X	X	X	X	X		X	X
**CBC**	X				X	X	X	X	X	X	X	X	X	X	X
**Comp chem panel**	X				X	X	X	X	X	X	X	X	X	X	X
**Coagulation parameters**	X							X[c]			X				X[c]
**Lipid panel**	X					X	X	X	X	X	X	X	X		X
**Serum lipase and amylase**	X					X	X	X	X	X	X	X	X		
**Lumbar puncture** [a]		X						X				X*			X
**CSF PK**								2hr							
**Serum PK**					trough (1)	trough (1)	Random (1)	trough and 2hr(2)	Random (1)	Random (1)	2-5 hr (1)				Random (1)
**HIV/HBV/HCV Testing**	X														
**DNA Sample***			X												
**Pregnancy Test**	X												X		X
**Blood Biomarker panel**			X					X			X	X			X
**CSF Biomarker panel**		X						X				X			X
**Adverse Events**			X	X	X	X	X	X	X	X	X	X	X	X	X
**Concomitant Drug Therapy**	X		X	X	X	X	X	X	X	X	X	X	X	X	X
**Randomization**			X												
**Dispense Study Drug**			X					X							
**Dose titration**					X										
**Dose management log**			X	X	X	X	X	X	X	X	X				
**Check drug compliance**				X	X	X	X	X	X	X	X			X	X

[a] Initial LP will be performed 7 to 14 days prior to the Baseline visit - after coagulation parameters, other safety labs and fundus exam results have been reviewed, considered normal and participant continues to qualify for the study. Subsequent LPs can be performed within the visit window <u>prior to or after</u> the scheduled visit (except V03) - after coagulation parameters and other safety lab results from previous visit have been reviewed and are normal. Fundoscopy must be completed prior to LP. LP for Visit 3 must be conducted on the same day all other assessments.

[b]Participants permanently discontinued from study drug after suspension or intolerance must complete the Premature Withdrawal (PW) visit on return to clinic. Participants will be encouraged to complete the 30d and 60d post drug evaluation visits V05 and V06.
[c]Participants must have coagulation parameters drawn within 10 days of their V03 and/or PW Lumbar Puncture. The results must be made available to the site investigator prior to performing the lumbar puncture.
[d] Alternate form
[*] Optional
MDS-UPDRS ON required if participant increases PD medications - even if not part of SOA for visit

SCHEDULE OF ACTIVITIES - Cohort 2

**Cohort 2**	**Screening**		**Base-line**	**Titration Phase**				**Maintenance Period**							**Final visit On drug[c]**	**Sympto-matic Therapy[d]**	**Post-Drug Eval**		**Un-Sched Visit**	**Pre-mature W/D[c]**
	**SC 1**<br>**Day -30 -14d**	**SC 2**<br>**Day -14 -7d**	**BL**<br>**Day 0**	**SV1**<br>**Day 7 ±3d**	**V01**<br>**Day 14 ±3d**	**V02**<br>**Day 30 ±3d**	**SV2**<br>**Day 60 ±7d**	**V03 3Mo**<br>**Day 90 ±7d**	**SV3**<br>**Day 120 ±7d**	**SV4**<br>**Day 150 ±7d**	**V04 6Mo**<br>**Day 180 ±7d**	**SV5**<br>**Day 210 ±7d**	**SV6**<br>**Day 240 ±7d**	**V05 9Mo**<br>**Day 270 ±7d**	**V06 12Mo**<br>**Day 365 ±7d**	**As Needed**	**V07**<br>**1 mo post +30d ±7d post**	**V08 (FNL)**<br>**2mo post +60 d ±7d post**	**(U01, U02, etc.)**	**PW Visit**
**Informed Consent**	X																			
**Participant Entry Numbers**	X																			
**Eligibility Criteria**	X		X																	
**Demography**	X																			
**Socio-economics**	X																			
**Med/Neuro History**	X																			
**MoCA**	X																			
**Beck Depression Inventory (BDI)- II**	X																			
**Vital Signs**	X		X	X	X	X	X	X	X	X	X	X	X	X	X	X	X	X	X	X
**Height, weight**	X							X			X			X	X	X	X			X
**Physical exam**	X		X					X			X			X	X	X	X			X
**Neurological exam**	X														X	X				X
**Fundoscopy exam**	X							X								X[e]	X			X
**Hoehn & Yahr**	X							X			X			X	X	X	X	X		X
**MDS-UPDRS I-IV ON[a#]**								X			X			X	X		X	X		X
**MDS-UPDRS III OFF**			X		X	X		X			X			X	X	X	X	X		X
**Assess need for symptomatic therapy**	X							X			X			X	X	X	X	X		X
**Modified Schwab & England scale**			X					X			X				X	X				X
**PD diagnostic features**	X														X	X				X
**Primary Dx form**	X														X	X				X
**Mattis Dementia Scale-2**			X								X[g]				X	X[g]				X[g]
**PDQ-39**			X								X				X	X				X
**EQ-5D**			X								X				X	X				X
**CGI I and P**			X								X				X	X				X
**C-SSRS**	X							X			X					X		X	X	X
**PDSS**			X								X				X	X				X

**Cohort 2**	Screening		Base -line	Titration Phase				Maintenance Period							Final visit On drug[c]	Sympto-matic Therapy[d]	Post-Drug Eval		Un-Sched Visit	Pre-mature W/D[c]
	SC 1<br>Day -30 -14d	SC 2<br>Day -14 -7d	BL<br>Day 0	SV1<br>Day 7 ±3d	V01<br>Day 14 ±3d	V02<br>Day 30 ±3d	SV2<br>Day 60 ±7d	V03 3Mo<br>Day 90 ±7d	SV3<br>Day 120 ±7d	SV4<br>Day 150 ±7d	V04 6Mo<br>Day 180 ±7d	SV5<br>Day 210 ±7d	SV6<br>Day 240 ±7d	V05 9Mo<br>Day 270 ±7d	V06 12Mo<br>Day 365 ±7d	As Needed	V07<br>1 mo post +30d ±7d post	V08 (FNL)<br>2mo post +60 d ±7d post	(U01, U02, etc.)	PW Visit
**Tobacco & Caffeine Q**			X																	
**Blindedness Q**															X	X[hi]	X			X
**Tx expectation Q**	X		X												X	X[ij]	X	X		X
**ECG-12 LEAD**	X		X	X		X	X	X	X	X	X	X	X	X	X	X	X		X	X
**CBC**	X				X	X	X	X	X	X	X	X	X	X	X	X	X	X	X	X
**Comp chem panel**	X				X	X	X	X	X	X	X	X	X	X	X	X	X	X	X	X
**Coagulation parameters**	X							X[f]							X	X[fh]				X [C]
**Lipid panel**	X				X	X	X	X	X	X	X	X	X	X	X	X	X	X		X
**Serum Lipase and amylase**	X				X	X	X	X	X	X	X	X	X	X	X	X	X	X		X
**Lumbar puncture [b]**		X						X								LP per SOA of visit replacing	X*			X
**CSF PK**								2 hr												
**Serum PK**					trough (1)	troug h (1)	Rando m (1)	trough and 2hr(2)	Rando m (1)	Rando m (1)	2-5hr (1)	Rando m (1)	Rando m (1)	trough (1)	trough (1)	PK per SOA of visit replacing				Rando m (1)
**HIV/HBV/HCV Testing**	X																			
**DNA Sample***			X																	
**Pregnancy Test**	X															X[j]		X		X
**Blood Biomarker panel**			X					X			X				X	X[hi]	X			X
**CSF Biomarker panel**		X						X								X[e]	X			X
**Adverse Events**			X	X	X	X	X	X	X	X	X	X	X	X	X	X	X	X	X	X
**Concomitant Drug Tx**	X		X	X	X	X	X	X	X	X	X	X	X	X	X	X	X	X	X	X
**Randomization**			X																	
**Dispense Study Drug**			X					X			X			X		X[efg]				
**Dose Titration**					X															
**Dose management log**			X	X	X	X	X	X	X	X	X	X	X	X	X	X				

**Cohort 2**	**Screening**		**Base -line**	**Titration Phase**				**Maintenance Period**							**Final visit On drug[c]**	**Sympto-matic Therapy[d]**	**Post-Drug Eval**		**Un-Sched Visit**	**Pre-mature W/D[c]**
	**SC 1**<br><br>**Day -30 -14d**	**SC 2**<br><br>**Day -14 -7d**	**BL**<br><br>**Day 0**	**SV1**<br><br>**Day 7 +3d**	**V01**<br><br>**Day 14 +3d**	**V02**<br><br>**Day 30 +3d**	**SV2**<br><br>**Day 60 +7d**	**V03 3Mo**<br><br>**Day 90 +7d**	**SV3**<br><br>**Day 120 +7d**	**SV4**<br><br>**Day 150 +7d**	**V04 6Mo**<br><br>**Day 180 +7d**	**SV5**<br><br>**Day 210 +7d**	**SV6**<br><br>**Day 240 +7d**	**V05 9Mo**<br><br>**Day 270 +7d**	**V06 12Mo**<br><br>**Day 365 +7d**	**As Needed**	**V07**<br><br>**1 mo post +30d +7d post**	**V08 (FNL)**<br><br>**2mo post +60 d +7d post**	**(U01, U02, etc.)**	**PW Visit**
**Check drug compliance**				**X**	**X**	**X**	**X**	**X**	**X**	**X**	**X**	**X**	**X**	**X**	**X**	**X**			**X**	**X**

[a] MDS-UPDRS ON to be completed once the participants start symptomatic therapy during the visits where it is included in the SOA. Initiation of any PD medication will warrant OFF/ON assessment

[b] Initial LP will be performed 7 to 14 days prior to the Baseline visit - after coagulation parameters, other safety labs and fundus exam results have been reviewed, considered normal and participant continues to qualify for the study. Subsequent LPs can be performed within the visit window prior to or after the scheduled visit (except V03) - after coagulation parameters and other safety lab results from previous visit have been reviewed and are normal. Fundoscopy must be completed prior to LP. LP for Visit 3 must be conducted on the same day all other assessments.

[c] Participants permanently discontinued from study drug after suspension or intolerance must complete the Premature Withdrawal (PW) visit on return to clinic. Participants will be encouraged to complete the 30d and 60d post drug evaluation visits V07 and V08.

[d] Symptomatic Therapy (ST) visits should be performed prior to initiation of ST and ideally should replace the regularly scheduled clinic visits using the designated schedule of activities.

[e] If ST visit is replacing V03; [f] If ST visit is replacing V04; [g] If replacing V05; [h] If replacing V06; [i] If replacing V07; [j] If replacing V08

[f] Participants must have coagulation parameters drawn within 10 days of their V03 and/or PW Lumbar Puncture. The results must be made available to the site investigator prior to perfoming the lumbar

puncture.
[g] Alternate form (as applicable)
* Optional
# MDS-UPDRS ON required if participant increases PD medications - even if not part of SOA for visit.

Protocol Synopsis 7
Table 1. List of abbreviations and definition of terms 28
1. INTRODUCTION 32
1.1 Background 32
1.2 Clinical Experience 33
2. STUDY OBJECTIVES 34
2.1 Primary Objective 34
2.2 Key Secondary Objective 35
2.3 Additional Secondary Objectives 35
2.4 Exploratory Objectives 35
2.4.1 Clinical Measures 35
2.4.2 Pharmacology and biomarkers objectives 36
3. STUDY DESIGN 37
3.1 Overview 37
3.2 Discussion of Study Design 39
3.2.1 Rationale for Study 39
3.2.2 Rationale for the study design 39
3.2.3 Nilotinib 40
3.2.4 Rationale for Dosage 41
4. STUDY POPULATION 42
4.1 Participant Numbers 42
4.2 Inclusion Criteria 42
4.3 Exclusion Criteria 43
4.4 Discussion of Participant Characteristics 45
4.4.1 Warnings/Precautions 45
5. STUDY PROCEDURES 51
5.1 Recruitment 51
6. SCHEDULE OF ACTIVITIES 52
6.1 Participant Identification (ID) Number 53
6.2 Screening Visits (SC1/SC2): Cohort 1 53

6.2.1 Screening Visit 1 - SC1: Cohort 1 54
6.2.2 Rescreening 55
6.2.3 Screening Visit 2- SC2: Cohort 1 55
6.3 Baseline Visit – BL (Day 0) – Cohort 1 56
6.4 Safety Visits SV1 through SV4 – cohort 1 58
6.5 Visit 01 (Day 14 + 3d) – V01 – Cohort 1 59
Visit 02 (Day 30 + 3d) – V02 – cohort 1 59
6.6 Visit 03 (Day 90 + 7d) – V03 – cohort 1 60
6.7 Visit 04 (Day 180+7d) – V04 – Cohort 1 (Final "On Drug" Visit) 63
6.8 Visit 05 (30 Days post-treatment ± 7 d) – V05 – Cohort 1 64
Visit 06 (60 Days post-treatment ± 7d) – V06 – End of Study 64
6.9 Unscheduled Visits – Visit U01, U02, etc. – Cohort 1 66
6.10 Premature Withdrawal Visit - Visit PW – Cohort 1 67
6.11 Procedures for the participants who Discontinue the study drug for safety and intolerability reasons 70
6.12 Screening Visit 1 (Visit SC 1) – Cohort 2 (EARLY/de novo PD) 70
6.12.1 Rescreening 72
6.13 Screening Visit 2 72
6.14 Baseline Visit – BL (Day 0) – Cohort 2 73
6.15 Safety VISITS - SV 1 through Sv 6 – Cohort 2 75
6.16 Visit 01 (Day 14 + 3d) – V01 – Cohort 2 76
Visit 02 (Day 30 + 3d) – V02 – Cohort 2 76
6.17 Visit 03 (Day 90 +7d) – V03 – Cohort 2 77
6.18 Visit 04 (Day 180 ±7d) – V04 – Cohort 2 79
6.19 Visit 05 (Day 270 ± 7d) – V05 – Cohort 2 82
6.20 Visit 06 (Day 365 ± 7d) – V06 – Cohort 2 (Final "On Drug" Visit) 84
6.21 Visit 07 (30 Days post-treatment ± 7 d) – V07 – Cohort 2 86
Visit 08 (60 days post-treatment + 7d) – V08 – COHORT 2 End of Study........ 86
6.22 Unscheduled Visits – Visit U01, U02, etc. – Cohort2 88
6.23 Premature Withdrawal Visit - Visit PW – Cohort 2 89
6.24 Symptomatic therapy Visit 92

6.25 Procedures for participants who Discontinue study drug for safety and intolerability reasons 94
7. STUDY DRUG ADMINISTRATION/ASSIGNMENT 95
7.1 Study Drug 95
7.2 Labeling 95
7.3 PHARMACIST/Site Drug Handling Instructions 96
7.4 Storage 97
7.5 Accountability of Study Drug Supplies 97
7.6 participant Number Assignment/Enrollment 97
7.7 Coding/Emergency Drug Disclosure 98
7.8 Dosage of Study Drug 99
7.8.1 Rules for Study Drug Administration 101
7.9 Rules for Dose Reduction 102
7.9.1 Rules for Dose Reduction for Intolerability 102
7.9.2 Rules for Dose Reduction/ Suspension for Safety Reasons 102
7.10 Rules for Study Drug Re-challenge 104
7.10.1 Rules for Dose Re-challenge in Case of Intolerability 104
7.10.2.Rules for Dose Suspension and Re- challenge for Safety Reasons 105
7.10.3 Rules for Dose Re-challenge in Cases not Related to Safety or Tolerability 105
8. CONCOMITANT MEDICATIONS 106
8.1 Allowed Concomitant Medications 106
8.2 Cautionary and Exclusionary Medications with Nilotinib 107
9. ASSESSMENTS 109
9.1 Primary Variables 109
9.2 Documentation of Parkinson Disease 109
9.3 Safety Assessments 109
9.3.1 Clinical Variables 109
9.3.2 Medical History 110
9.3.3 Physical Examination 110
9.3.4 Neurological Examination 110
9.3.5 Vital Signs, Weight, Height 110

9.3.6 Clinical Laboratory Tests 110
9.3.7 Fundoscopy Exam 111
9.3.8 Lumbar puncture (LP) 111
9.3.9 Electrocardiogram (ECG) 112
10. CLINICAL ASSESSMENTS 112
10.1. Movement Disorders Society Unified PD Rating Scale (MDS-UPDRS) 112
10.2 Modified Schwab & England Activities of Daily Living Scale 113
10.3 Parkinson's Disease Questionnaire - 39 item version (PDQ-39) scale 114
10.4 Hoehn and Yahr Scale 114
10.5 Montreal Cognitive Assessment (MoCA) 114
10.6 Primary Diagnosis Assessment 114
10.7 PD Features 115
10.8 Beck Depression Inventory-II (BDI-II) 115
10.9 Parkinson's Disease Sleep Scale (PDSS) 115
10.10 European Quality of Life Scale (EQ-5D) 115
10.11 Blindedness Evaluation 115
10.12 C-SSRS (baseline and since last visit) 116
10.13 Mattis Dementia Rating Scale (DRS-2) 116
10.14 Clinical Global Impression scale 116
10.15 PD – Study Treatment Expectancy Questionnaire 116
10.16 Additional Assessments 117
10.16.1 Assessment of Participant Compliance 117
10.16.2 Assess need for Symptomatic Therapy (Cohort 2) 117
10.16.3 Pharmacokinetic Assessments 117
10.16.4 Biomarkers Assessments 118
11. INTERCURRENT ILLNESS 119
12. PREMATURE WITHDRAWALS/DROPOUTS 120
13. ADVERSE EVENTS 122
13.1 Adverse Event (AE) Definition 122
13.2 Serious Adverse Events (SAE) 123
13.3 Adverse Event Definitions 124

13.4 Identifying and Recording of Adverse Events 124
13.5 Adverse Event Causality Definitions 125
13.6 Adverse Event Severity Definitions 126
13.7 Reporting Serious Adverse Events 126
13.8 SERIOUS ADVERSE EVENTS EXPECTEDNESS TO THE STUDY INTERVENTION 127
13.9 Review of Adverse Events 128
13.10 Follow-Up of Unresolved Events 128
13.11 Reporting of Pregnancy 129
13.12 Reportable Events 129
14. STATISTICAL CONSIDERATIONS 130
14.1 General Design Issues 130
14.1.1 Summary of Study Design – Cohort 1 130
14.1.2 Summary of Study Design – Cohort 2 131
14.1.3 Randomization 132
14.2 Outcomes 132
14.2.1 Primary Outcome 132
14.2.2 Secondary Outcome 132
14.3 Analysis Plan – Cohort 1 133
14.3.1 Primary Hypotheses 133
14.3.2 Decision Algorithm Based on Primary Hypotheses 134
14.3.3 Key Secondary Objective 135
14.3.4 Other Secondary Objectives 136
14.3.5 Exploratory Objectives 137
14.4 Analysis Plan – Cohort 2 137
14.4.1 Primary Hypotheses 137
14.4.2 Secondary Objectives 138
14.4.3 Exploratory Objectives 139
14.5 Sample Size Justification 139
14.5.1 Sample Size – Cohort 1 139
14.5.2 Sample Size – Cohort 2 141

14.6 Impact of Missing Data 141
15. PHARMACOLOGY 142
15.1 Pharmacology Plan 142
15.2 Pharmacokinetic Analysis 142
16. REGULATORY/ETHICS 143
16.1 Compliance Statement 143
16.2 Informed Consent 143
16.3 Institutional Review Board 143
16.4 Protocol Amendments 144
16.5 Participant Confidentiality 144
17. DOCUMENTATION 145
17.1 Investigator Site File 145
17.2 Maintenance and Retention of Records 146
17.3 Quality Assurance (QA) Audits/Site Visits 146
17.4 Regulatory Inspections 147
17.5 Data Management 147
18. SITE INVESTIGATOR/SITE 148
19. CLINICAL SAFETY MONITORING 148
19.1 Clinical Monitor 148
19.2 Independent Medical Monitor 148
19.3 Study Committees 149
19.3.1 Steering Committee 149
19.3.2 Data and Safety Monitoring Board (DSMB) 149
19.4 Case Report Forms 149
19.5 Site Monitoring 150
19.6 Primary Source Documents 151
19.7 Closeout Plan 151
20. Publication of Research Findings 152
21. Data Sharing 152
22. REFERENCES 153
APPENDIX 1: Tasigna Package Insert 156

APPENDIX 2: Exclusionary and Precautionary Medications 191

APPENDIX 3: Steering Committee 198

Table 1. List of abbreviations and definition of terms

Abbreviation or specialist term	Definition
ADL	Activities of Daily Living
AE	Adverse Event
ALP	Alkaline Phosphatase
ALT	Alanine Aminotransferase
ALP	Alkaline Phosphatase
AST	Aspartate Aminotransferase
AUC	Area under the concentration-time curve
AV	Atrioventricular
BCC	Biostatistics Coordination Core
BDI-II	Beck Depression Inventory-II
BP	Blood Pressure
BUN	Blood Urea Nitrogen
CAPA	Corrective and Preventive Action Plan
CBC	Complete Blood Count
CDE	Common Data Elements
CFR	Code of Federal Regulations
CML	Chronic Myelogenous Leukemia
CMSU	Clinical Materials Services Unit
CGI	Clinical Global Impression Scale
CNS	Central Nervous System
CPK	Creatinine Phosphokinase
CRA	Clinical Research Associate
CRF	Case Report Form
CSF	Cerebrospinal Fluid

**Abbreviation or specialist term**	**Definition**
C-SSRS	Columbia Suicide Severity Rating Scale
CTCAE	Common Terminology Criteria for Adverse Events
CTCC	Clinical Trials Coordination Center
DCC	Data Coordination Core
DBP	Diastolic Blood Pressure
DLB	Dementia with Lewy Body
DRS-2	Mattis Dementia Rating Scale
DSMB	Data Safety Monitoring Board
ECG	Electrocardiogram
EDC	Electronic Data Capture
eCRF	Electronic Case Report Form
EQ-5D	European Quality of Life Scale
FDA	Food and Drug Administration
GCP	Good Clinical Practice
HBV	Hepatitis B virus
HCV	Hepatitis C virus
HIPAA	Health Insurance Portability and Accountability Act
HIV	Human Immunodeficiency Virus
HSPP	Human Subjects Protection Program
H&Y	Hoehn and Yahr Scale
HVA	Homovanilic Acid
ICD	International Classification of Diseases
ICF	Informed Consent Form
ICH	International Conference on Harmonisation
ID	Identification
IP	Investigational Product
IRB	Institutional Review Board

Abbreviation or specialist term	Definition
ITT	Intent-to-treat
LDE	Levodopa Dose Equivalence
LFT	Liver Function Tests
LMM	Linear Mixed Effects Model
LP	Lumbar Puncture
MAO-B	Monoamine Oxidase B
MDS-UPDRS	Movement Disorders Society Unified PD Rating Scale
MedDRA	Medical dictionary for Regulatory Activities
MJFF	Michael J. Fox Foundation
MRI	Magnetic Resonance Imaging
MMSE	Mini Mental Status Exams
MoCA©	Montreal Cognitive Assessment
NDA	New Drug Application
NCI	National Cancer Institute
NINDS	National Institute of Neurological Disorders and Stroke
OTC	Over the Counter
PBMC	Peripheral Blood Mononuclear Cell
PD	Parkinson's Disease
PDD	Parkinson's Disease Dementia
PDQ-39	Parkinson's Disease Questionnaire – 39 Item Version Scale
PDSS	Parkinson's Disease Sleep Scale
PI	Principal Investigator
PK	Pharmacokinetics
PP	Per Protocol
PRO	Patient Reported Outcomes
PSG	Parkinson Study Group
PT	Preferred Term
PW	Premature Withdrawal

**Abbreviation or specialist term**	**Definition**
QD	Daily
QTc	Corrected QT Interval
QTcF	Fridericia's correction for QT interval
ULN	Upper Limit of Normal
RBC	Red Blood Cell
S&E	Modified Schwab and England Scale
SAE	Serious Adverse Event
SAP	Statistical Analysis Plan
SBP	Systolic Blood Pressure
SC	Steering Committee
SCOPA-Cog	Scales for Outcomes in Parkinson's Disease-Cognition
SD	Standard Deviation
SOA	Schedule of Activities
SOC	System Order Class
SOP	Standard Operating Procedure
ST	Symptomatic Therapy
TEAE	Treatment Emergent Adverse Event
$T_{1/2}$	Terminal half-life
T_{max}	Time at which C_{max} (serum concentration) was obtained
V_{ss}	Steady state volume of distribution
V_z	Volume of distribution
WBC	White Blood Cell

1. INTRODUCTION

1.1 BACKGROUND

Parkinson's disease (PD) is the second most common neurodegenerative disease that affects 1% of the population above the age 65 [11]. The prevalence of PD will increase substantially in the next 20 years due to the aging of the population and age-related increase of the incidence of the disease [12,13]. PD is characterized by progressive motor disability that includes bradykinesia, rigidity, resting tremor and gait dysfunction. PD is associated with a spectrum of non-motor symptoms including autonomic, cognitive, mood, sleep dysfunction and sensory abnormalities which are intrinsically related to the widespread PD neuropathological process and can in part precede the onset of motor manifestations [14]. The economic burden of PD is estimated to be $23 billion annually in US and projected to increase to $50 billion by year 2040 [15]. Most of the cost is due to lost productivity and correlates with more advanced stages of the disease signifying the importance of developing treatment strategies that slow progression of accumulating disability [16].

Treatment options for PD are limited to symptomatic therapy (ST) geared towards replacement of dopamine deficiency [17]. Despite a wide armamentarium of effective ST for early PD, management of advanced disease is limited. Availability of an effective disease modifying intervention that will slow the progression of the disease will have a substantial impact on the patients' quality of life and the economic burden of disease.

As of today there is no single proven neuroprotective agent in PD [18]. Tested agents targeted various potential mechanisms of PD pathogenesis including oxidative stress (rasagiline, selegiline, Vitamin E), mitochondrial dysfunction (Coenzyme Q10, creatine), apoptotic mechanism of cell death (caspase inhibitors) and others [19-27].

The defining pathological features of PD include the progressive loss of dopaminergic neurons in the substantia nigra-pars compacta and intra-neuronal accumulation of misfolded proteins to form Lewy bodies and Lewy neurites, where α -synuclein is the major component [28,29]. Mutations in the gene encoding alpha (α) -synuclein, *SNCA*, lead to autosomal dominant PD while polymorphisms constitute risk factors for non-familial PD (reviewed by Mullin and Schapira[30]). Thus α -synuclein represents a convergent point between genetic etiology and pathology of PD and as such is an attractive target for therapeutic interventions.

Recent genetic, biochemical and pharmacological studies indicate that deficits in the cellular pathways involved in autophagy underlie inadequate clearance of misfolded α -synuclein and contribute to PD pathogenesis (reviewed by Brundin et al.; Xilouri et al.,[31,32]). A class of enzymes termed tyrosine kinases and exemplified by the non-receptor tyrosine kinase Abelson (c-Abl) are known to induce autophagy[33]. In fact, inhibitors of c-Abl have emerged as key therapies of certain tumors such as chronic myeloid leukemia (reviewed by Turcotte and Giaccia, 2010[34]) .

Recent analyses of autopsied tissues from PD patients and animal models of PD show an increase in the level of c-Abl as well as increases in the phosphorylation of its protein substrates, α-synuclein and parkin in dopaminergic neurons[1,3,7,35-37]. Interestingly, parkin is an E3 ubiquitin ligase, an enzyme critical for proteasome-mediated protein clearance. Like α -synuclein, mutations and polymorphisms in the *parkin* gene also lead to familial PD and represent risk factors of sporadic PD, respectively[38]. More importantly, inhibition of c-Abl by nilotinib, a drug used for the treatment of Chronic Myelogenous Leukemia (CML), ameliorated α -synuclein pathology and rescued dopamine neuronal loss in mouse models of PD[1,3]. Further support for a critical role of c-Abl in inducing α -synuclein-mediated PD pathogenesis was recently provided by Brahmachari et al. [39]. Using mouse genetic models they demonstrated that deletion of the gene encoding c-Abl reduced α-synuclein aggregation and neurobehavioral deficits whereas overexpression of the constitutively active c-Abl accelerated α-synuclein aggregation, neuropathology, and neurobehavioral deficits. Thus the first steps towards the development of c-Abl targeted cancer therapies for PD was undertaken and reported by Pagan et al[9].

1.2 CLINICAL EXPERIENCE

Nilotinib experience in PD population

To date, the safety and tolerability of nilotinib in PD and Dementia with Lewy Bodies (DLB) population have only been studied in a single small open label clinical study [9]. The primary objective of the study was safety and tolerability with the secondary objectives of target engagement and pharmacokinetics (PK) and exploratory clinical outcomes. The study recruited 12 participants with advanced PD, PD dementia (PDD) and Lewy body dementia who were randomized to nilotinib 150mg versus 300mg once daily in an open label fashion and were followed for 6 months.

Thirty patients were screened and twelve late stage (Hoehn and Yahr 3-5) PD, PDD or DLB participants were randomized into 150mg (N=5) or 300mg (N=7) groups and received oral daily doses of nilotinib for 24 weeks with 3 months follow up. Eighteen patients failed screening due to cardiovascular disease, infarcts, corrected QT (QTc) interval >450ms or excluded medications (i.e. warfarin). One participant was withdrawn from the study due to serious adverse events (SAEs) and another discontinued at 5 months due to increased caregiver burden. Ten participants completed the 24-week study and returned for a follow up visit (week 36). One participant did not follow up at week 36.

Clinical outcomes included Unified Parkinson's Disease Rating Scale (UPDRS) and Mini Mental Status Exams (MMSE). Motor symptoms were monitored with UPDRS. The UPDRS scores were obtained at 1-4 hours after dosing with nilotinib. An average decrease of 3.4 points and 3.6 points in UPDRS-III (motor) was observed at six months (week 24) compared to baseline with 150mg and 300mg nilotinib, respectively. These effects were reversed at 36-week

follow up visit. An average decrease of 7 points and 11.1 points in UPRDS I-IV was also observed at 6 months in the 150mg and 300mg groups, respectively. The change in UPDRS was progressive throughout the 6 months (24 weeks) trial, and was reversed in participants in the follow up visit at 36 weeks. Most participants experienced increased psychotic symptoms (hallucination, paranoia, agitation, etc.) and some dyskinesia.

Cognitive performance was monitored with MMSE throughout all visits. An average increase of 3.85 and 3.5 points in MMSE was observed at six months (24 weeks) compared to baseline with 150mg and 300mg nilotinib, respectively. The increase in MMSE scores was incremental throughout the 24-week treatment, and returned to baseline at the 36-week follow up visit. The Scales for Outcomes in Parkinson's Disease-Cognition (SCOPA-Cog) test also showed an average increase of 1.85 and 2 points at six months compared to baseline with 150mg and 300mg nilotinib, respectively.

While the investigators concluded that nilotinib at tested doses was safe and tolerable in PD population, one participant had a serious cardiac event during the study and two others had QT prolongation (both are within the spectrum of the known nilotinib related adverse events and QT prolongation is reflected in the drug black box warning). The authors reported central nervous system penetration of unbound nilotinib, with the Cerebral Spinal Fluid (CSF):plasma ratio of 12% and 5 % with 300mg and 150mg dose respectively at 2 hours post dose administration and non-detectable CSF levels at 3 hours. With regard to the secondary outcomes of target engagement, the authors reported reduction of phosphorylated Abl by 30 % with both doses of nilotinib. In addition, the authors reported significant increase in CSF dopamine metabolite, homovanilic acid (HVA) in both treatment groups and interpreted it as potentially reflection of nilotinib induced up regulation and protection of dopamine neurons. The authors also reported significant symptomatic benefit of nilotinib with improvement of motor disability and cognitive performance both of which subsided 3 months after discontinuation of treatment. Although the preliminary data appear promising, the study design precludes firm conclusions about safety, tolerability, optimal dose or efficacy. In addition, the data on the PK profile and target engagement have to be validated and replicated[10]. Nilotinib is approved by the Food and Drug Administration (FDA) for certain types of leukemia but not PD.

An opportunity exists to establish rigorously the safety, tolerability and optimal dosing of nilotinib for future efficacy studies in PD patients.

2. STUDY OBJECTIVES

2.1 PRIMARY OBJECTIVE

The primary objective of this two-cohort study is to assess the safety and tolerability of the daily oral administration of nilotinib or placebo in Participants with moderate to advanced PD (Cohort

1) and in Participants with early/de novo PD (Cohort 2). Participants in Cohort 1 will be treated with nilotinib 150mg, 300mg or matching placebo. If at least one dose is shown to be safe and tolerable in cohort 1, the study steering committee will select a dose for consideration in cohort 2. Participants in Cohort 2 will be treated with the selected dose determined in Cohort 1 or matching placebo.

2.2 KEY SECONDARY OBJECTIVE

To conduct a futility analysis within each treated group in Cohort I. This futility analysis will compare the observed change in the Movement Disorder Society Unified Parkinson's Disease Rating Scale (MDS-UPDRS) part III between baseline and 6 months to the observed change from the clintrials.gov NCT02281474 nilotinib study in order to determine whether we can rule out the large change previously reported – i.e., declare "futility".

2.3 ADDITIONAL SECONDARY OBJECTIVES

To establish the degree of symptomatic effect of nilotinib as measured by:

1) The change in MDS-UPDRS part III between baseline and Visit 2 (1 month)
2) Final visit on study drug and 30 days off study drug in cohorts 1 and 2.

To explore the impact of nilotinib on progression of PD disability as measured by the change in the MDS-UPDRS Part III score in the defined medications OFF state in Cohort 1 (moderate/advanced) between baseline and 6 months.

To establish the impact of nilotinib on progression of PD disability as measured by the change in the MDS-UPDRS Part III score in Cohort 2 (early/de novo) between baseline and 12 months or time to initiation of ST whichever comes first.

2.4 EXPLORATORY OBJECTIVES

2.4.1 CLINICAL MEASURES

The change in the following measures of disability, quality of life and functional status from baseline to final visit will be analyzed:

Motor disability

- The change in MDS-UPDRS total score. For participants on ST, MDS-UPDRS part III assessments will be collected in the defined medications OFF (approximately 12 hours post dose) and ON (based on the participant/site investigator defined best ON and/or approximately 1 hour after dose of ST) state
- The change in the ambulatory capacity (sum of 5 MDS-UPDRS questions: (falling,

freezing, walking, gait, postural stability)
- Analysis of ST utilization as measured by levodopa equivalence dose
- Severity of motor complications as measured by MDS-UPDRS IV subscale (complications of therapy)

Cognitive disability
- The change in the cognitive function as measured by the change in Mattis dementia rating scale - 2 (DRS-2)

Sleep function
- The change in sleep quality as measured by the Parkinson's disease sleep scale (PDSS)

Measures of global disability
- Clinician and Participant Global Impression of Change Score (CGI)

Measures of functional status and quality of life
- The change in the MDS-UPDRS Motor and Non-Motor Experiences of Daily Living subscores
- The change in the modified Schwab and England scale (S&E)
- The change in Parkinson Disease Quality of Life Questionnaire 39 (PDQ-39)
- The change in European Quality of Life Scale (EQ-5D)

Blindedness and Treatment Expectations assessments
- Questionnaires completed by the Site Investigator and Participant

2.4.2 PHARMACOLOGY AND BIOMARKERS OBJECTIVES

1. Pharmacokinetics and pharmacodynamics
    a. To determine the serum PK of nilotinib in patients with PD.
    b. To investigate the ability of nilotinib to cross the blood brain barrier in PD patients at a dose(s) that is tolerable and safe as measured by dose-dependent increases in unbound nilotinib levels in the CSF.
    c. To investigate factors that could affect the PK of nilotinib such as age, sex, PD severity, race/ethnicity.
    d. To investigate the ability of nilotinib to engage its known molecular target(s) at a dose that is tolerable and safe as measured by biomarkers of pathway inhibition in the serum and CSF (including but not limited to reduction in phospho-cAbl tyrosine kinase).

e. To investigate the relationship between nilotinib exposures in serum and CSF with its ability to engage known molecular target(s) (measured by biomarkers of pathway inhibition in the serum and CSF (including but not limited to reduction in phospho-cAbl)).

2. To explore impact of nilotinib on:
    a. Dopaminergic system as measured by an increase in dopamine and its metabolites in CSF (e.g., homovanilic acid (HVA) level)
    b. Other biomarkers linked to potential mechanism of neuroprotection (including but not limited to CSF α -synuclein, total tau, phospho-tau, etc.)

3. Store additional blood/serum/ plasma/DNA/CSF samples for future research

3. STUDY DESIGN

3.1 OVERVIEW

This is a Phase IIa, multicenter, randomized, double-blind, placebo-controlled, parallel group study of nilotinib in participants with moderate to advanced (Cohort 1) and early/de novo (Cohort 2) PD. The study will define the safety, tolerability, and exploratory clinical and biological activity of the chronic administration of nilotinib in participants with PD.

Participants in Cohort 1 (moderate/advanced PD) will be randomized in a 1:1:1 ratio to a once daily dose of either 150mg or 300mg of nilotinib or matching placebo. The duration of Cohort 1 study is expected to be approximately 8.5 months from screening to post drug evaluation; whereas treatment period from randomization to final "On Treatment" visit will be 6 months. The analysis for safety and tolerability will be conducted after all participants in Cohort 1 have completed 6 months of their randomized treatment assignment. These results will be analyzed to inform the dose chosen for evaluation in Cohort 2.

Cohort 2 will treat early/de novo PD participants. Participants will be randomized in a 2:1 ratio to:

- nilotinib – selected dose, based on safety and tolerability data in Cohort 1.

  or

- matching placebo

The duration of Cohort 2 study is expected to be approximately 14.5 months from screening to post drug evaluation; whereas the treatment period from randomization to final "On Treatment" visit will be 12 months.

All participants in both Cohorts will be dosed once daily (QD) in a fasted state (no food consumed for at least 2 hours before and at least one hour after dosing).

Sparse PK sampling will be conducted to assess serum and CSF concentrations of nilotinib throughout the duration of the study in both Cohorts (see section 11.3).

The study design is displayed diagrammatically below:

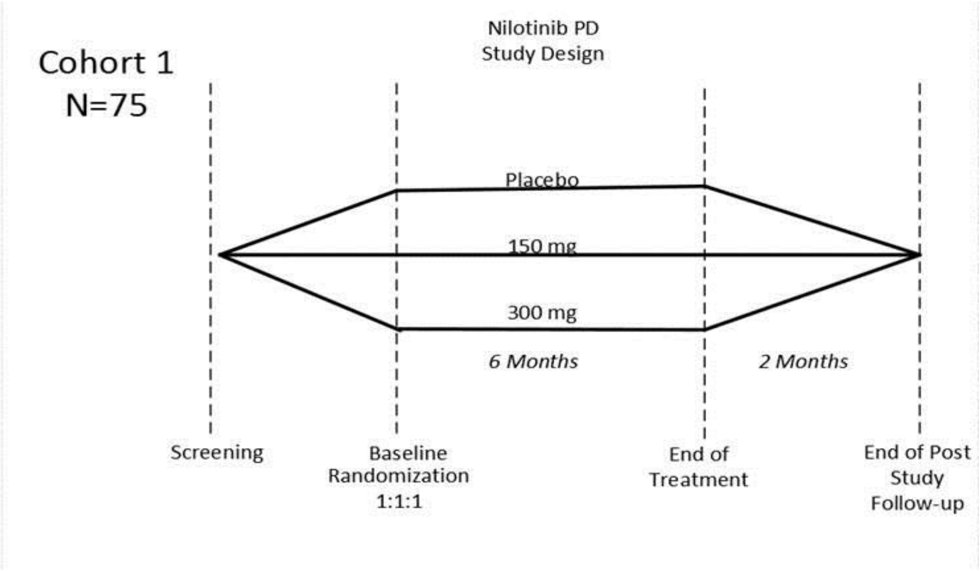

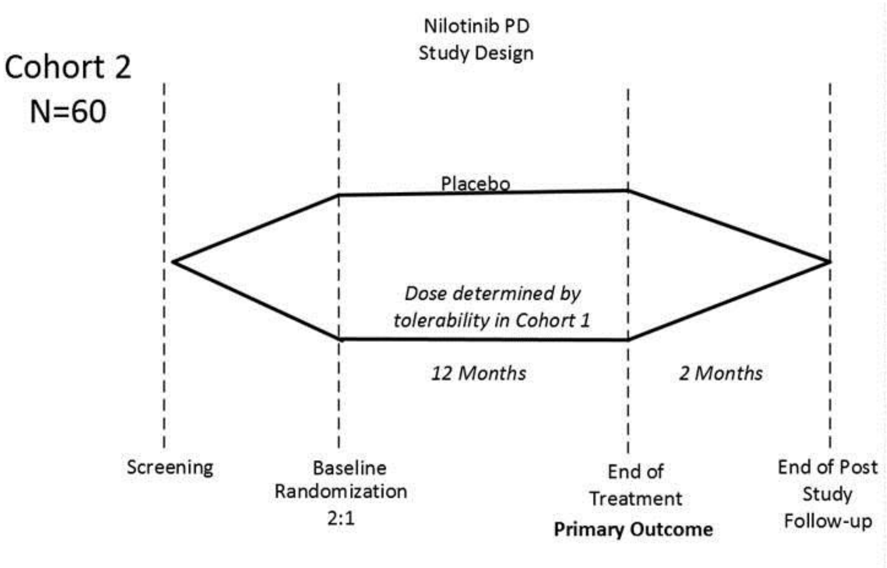

Note: Cohort 2 dose will be determined based on safety and tolerability in Cohort 1.

3.2 DISCUSSION OF STUDY DESIGN

3.2.1 RATIONALE FOR STUDY

A small number of cell and animal models suggest that nilotinib may positively affect the α -synuclein pathology observed in PD[1-3,7,31,35,36,39]. A small clinical study, that lacked a placebo control group and blinding, tested the safety and tolerability of nilotinib in PD patients for the first time and also explored its efficacy[9]. Although the preliminary data appear promising, the study design precludes firm conclusions about safety, tolerability, optimal dose or efficacy[9]. Nilotinib is approved by the FDA for certain types of leukemia but not for PD. An opportunity exists to rigorously establish the safety, tolerability as well as optimal dose of nilotinib to enable future definitive efficacy studies in PD patients.

3.2.2 RATIONALE FOR THE STUDY DESIGN

The study is planned as a two-cohort study. Based on the fact that `the data on the safety profile of the drug and prior clinical experience with nilotinib were limited to an advanced PD population with cognitive deficits, PDD a or DLB, it was felt prudent to validate and expand the data on nilotinib safety and tolerability in the moderately advanced PD population. It is expected that tolerability of nilotinib in Cohort 1 will be lower than in Cohort 2 (early/de novo PD population). Such data are important to establish, provided that nilotinib, if shown to have disease modifying benefit, will be administered long-term to PD patients across all disease stages. Another important objective of the study is to establish the magnitude of potential symptomatic effect of nilotinib and dopaminergic therapy interaction. These data will be essential for the design of future disease modifying trials.

If shown to be safe and well tolerable, future development of nilotinib will target disease modification objectives. Disease modification has historically been tested in cohorts of de novo PD population. To pursue future disease modification trials, safety and tolerability of nilotinib has to be established in the de novo PD population as well. While we do not expect that the de novo population will have a lower tolerability and safety threshold, this hypothesis must be tested. In addition, a key piece of information to be collected in Cohort 2 is the presence and degree of symptomatic effect of nilotinib, if such exists. These data will be essential for the design of the future disease modifying trials. Lastly, while the study is not powered to test efficacy of nilotinib as a putative disease modifying intervention, the data on the magnitude of the effect of nilotinib versus placebo on the rate of progression in PD disability will be collected. These data will guide the decision to pursue future clinical evaluation for that indication as well as provide essential data for the power calculations and sample size estimation for future disease modifying trials.

3.2.3 NILOTINIB

Nilotinib mechanism of action and pharmacokinetic data

Nilotinib (Tasigna®, AMN107, Novartis, Switzerland) is a kinase inhibitor approved by the FDA for the treatment of adult patients with the newly diagnosed Philadelphia chromosome positive chronic myeloid leukemia (Ph+ CML) in chronic phase (CP) and accelerated phase (AP) Ph+ CML in adult patients resistant to or intolerant to prior therapy that included imatinib. Nilotinib is available in 150mg and 200mg capsules. The recommended dose of nilotinib for CML ranges from 300 to 400mg orally twice-daily (600-800mg/day).

Please use the following link to view the package insert https://www.pharma.us.novartis.com/sites/www.pharma.us.novartis.com/files/tasigna.pdf

Nilotinib is an inhibitor of the breakpoint cluster region protein (BCR)-Abl kinase. Nilotinib binds to and stabilizes the inactive conformation of the kinase domain of Abl protein. Tasigna (nilotinib) capsules, for oral use, contain 150mg or 200mg nilotinib base, anhydrous (as hydrochloride, monohydrate) with the following inactive ingredients: colloidal silicon dioxide, crospovidone, lactose monohydrate, magnesium stearate and poloxamer 188. The capsules contain gelatin, iron oxide (red), iron oxide (yellow), iron oxide (black), and titanium dioxide. Tasigna (nilotinib) capsules should be stored at 25°C (77°F); excursions permitted between 15° to 30°C (59° to 86°F)

Pharmacokinetics of Nilotinib

The absolute bioavailability of nilotinib has not been determined. As compared to an oral drink solution (pH of 1.2 to 1.3); relative bioavailability of nilotinib capsule is approximately 50%. Peak serum concentrations of nilotinib are reached 3 hours after oral administration. Steady-state nilotinib exposure was dose-dependent with less than dose-proportional increases in systemic exposure at dose levels higher than 400mg given as once-daily dosing. Steady state exposure area under the concentration-time curve (AUC) of nilotinib with 400mg twice-daily dosing was 13% higher than with 300mg twice-daily dosing. The average steady state nilotinib trough and peak concentrations did not change over 12 months. There was no relevant increase in exposure to nilotinib when the dose was increased from 400 mg twice-daily to 600mg twice-daily.

The bioavailability of nilotinib is increased with food, thus nilotinib must not be taken with food. **No food should be consumed for at least 2 hours before and for at least 1 hour after the dose is taken.** Also avoid grapefruit products and other foods that are known to inhibit CYP3A4 *[see Cautionary and Exclusionary Medications in Appendix 2]*

The blood-to-serum ratio of nilotinib is 0.68. Serum protein binding is approximately 98% based on *in vitro* experiments.

The apparent elimination half-life estimated from the multiple dose PK studies with daily dosing was approximately 17 hours. Inter-patient variability in nilotinib AUC was 32% to 64%. Steady state conditions were achieved by Day 8. An increase in serum exposure to nilotinib between the first dose and steady state was approximately 2-fold for daily dosing and 3.8-fold for twice-daily dosing.

Main metabolic pathways identified in healthy participants are oxidation and hydroxylation. CYP3A4 is expected to be the main contributor to oxidative metabolism with CYP2C8 also making contribution. In vitro studies show nilotinib to be a competitive inhibitor of CYP3A4/5, 2C8, 2C9, 2D6, and UGT1A1. Additionally, in vitro studies demonstrate possible induction of CYP2B6, 2C8, and 2C9. Nilotinib is the main circulating component in the serum. None of the metabolites contribute significantly to the pharmacological activity of nilotinib.

After a single dose of radiolabeled nilotinib in healthy participants, more than 90% of the administered dose was eliminated within 7 days: mainly in feces (93% of the dose). Parent drug accounted for 69% of the dose. Age, body weight, gender, or ethnic origin did not significantly affect the PK of nilotinib.

3.2.4 RATIONALE FOR DOSAGE

The FDA approved dose of nilotinib for CML ranges from 300 to 400mg orally twice-daily. The study will test two doses of nilotinib 150mg and 300mg administered once daily. We have chosen to target the lowest dosing range for this study based on the concerns regarding the safety profile of the tested agent and preliminary data from Pagan et al[9] demonstrating CNS penetration at these doses which would require confirmation in the current study. For the same reasons we have chosen once daily schedule of administration.

To minimize bias on the part of participants, Site Investigators, and analysts, the study is to be conducted as a randomized, double-blind, placebo-controlled, parallel group study. To maintain the blind, study drug and placebo will be manufactured as matching capsules, packaged and provided in matching containers.

4. STUDY POPULATION

4.1 PARTICIPANT NUMBERS

This study will enroll a total of approximately one hundred eighty (180) participants with the goal of randomizing one hundred thirty-five (135) participants in two cohorts at approximately 25 sites in the United States (US). Enrollment will be conducted in two cohorts:

- Cohort 1: Approximately seventy-five (75) participants with moderate to advanced PD will be randomized 1:1:1 to a once daily dose of nilotinib or placebo (150mg; 300mg; placebo)
- Cohort 2: Approximately sixty (60) participants with early/de novo PD will be randomized to a once daily dose of nilotinib or placebo (the selected dose from Cohort 1, based on safety and tolerability). Randomization in this cohort will not commence until/if a dose is determined from Cohort 1.

Refer to Section 15 Statistical Considerations for justification of the selected sample sizes and the process for selecting the dose from cohort 1 for consideration in cohort 2.

4.2 INCLUSION CRITERIA

<u>Both Cohorts 1 and 2 will include participants who meet the following criteria</u>:

1. Participants with idiopathic PD based on the UK Brain Bank diagnostic criteria.
2. Participants of any race and either gender, age 40-79 on the date the informed consent form (ICF) is signed and with the capacity to provide voluntary informed consent.
3. Participants able to read and understand English and the ICF and are willing to comply with all study procedures, treatment and follow-up.
4. Participants must be willing to undergo multiple lumbar punctures (LP) as outlined in the schedule of activities.
5. Participants who are taking any central nervous system acting medications (e.g., benzodiazepines, antidepressants, hypnotics) must be on a stable regimen for at least 30 days prior to the baseline visit.

Inclusion criteria specific for <u>Cohort 1</u>:

6a. Participants with a diagnosis of PD duration > 5 years.
7a. Participants with H&Y stage > 2 and < 4 in the ON state.
8a. Participants on a stable regimen of PD medications, that includes levodopa, for at least 30 days prior to the screening visit.
  a. Treatment with monoamine oxidase B (MAO-B) inhibitors will be allowed provided the dose has been stable for 60 days prior to baseline.

Inclusion criteria specific for Cohort 2:

6b. Participants with a diagnosis of PD duration < 3 years.
7b. Participants with H&Y stage ≤ 2.
8b. Participants who are currently NOT receiving ST (levodopa, dopamine agonists and monoamine oxidase B (MAO-B) inhibitors) and NOT projected to require ST for at least 3 months from enrollment.
   a. Treatment with amantadine or an anticholinergic agent will be allowed provided the dose has been stable for 30 days prior to screening and will remain stable for the duration of the study.

4.3 EXCLUSION CRITERIA

Both Cohorts 1 and 2 will exclude participants who meet the following criteria:

1. Participants with a diagnosis of atypical parkinsonism.
2. Participants with a history of bipolar disorder or major depression, or the presence of active depression defined as a Beck Depression Inventory II (BDI-II) score >17.
3. Participants with a history of a suicide attempt within the last 5 years or active suicidal ideations
4. Participants with a history of schizophrenia or schizophrenia spectrum disorders
5. Participants with a history of uncontrolled hypokalemia or hypomagnesaemia, or laboratory evidence of such on screening
6. Pariticipants with evidence of other significant laboratory abnormalities which in the opinion of the site investigator or clinical monitor should preclude study participation
7. Participants with history of a cardiac arrhythmia, long QT syndrome, or a QTcF ≥450ms at screening visit 1
8. Participants treated within 30 days prior to randomization, or planned use during the trial with any of the following classes of Concomitant drugs (see full list in Appendix 2):
   a. Class IA or III antiarrhythmic drugs
   b. QT prolonging drugs
   c. Strong CYP3A4 inhibitors or inducers
   d. Anticoagulants
   e. Proton pump inhibitors
9. Participants with a clinical history, or the active presence of a cardiovascular condition including:
   a. Myocardial infarction, known cardiac ischemia, or angina
   b. Cerebrovascular event (e.g. embolic stroke)
   c. Congestive heart failure, symptomatic first degree atrioventricular (AV) block or PR interval >220msec and all second and third degree AV block, sick sinus syndrome, or other serious cardiac rhythm disturbances

d. Any history of Torsade de Pointes
e. Any other cardiovascular history that in the opinion of the Site Investigator will preclude study participation

10. Participants with a history of hepatic disease, including abnormal liver function defined Total Bilirubin > 1.5 times upper limit of normal, Aspartate Aminotransferase (AST) and/or Alanine Aminotransferase (ALT) > 2 times the upper limit of normal, or coagulopathy with INR > 4
11. Participants with a history of bone marrow suppression or evidence of persistent myelosuppression defined as absolute neutrophyl count <1.8 X 10^9/L, significant anemia, or thrombocytopenia defined as platelet count < 100 X 10^9/L
12. Participants with a prior history of epilepsy or a seizure within the last 6 months
13. Participants with an active malignancy, or history of a neoplasm in the prior 5 years (excluding basal/squamous cell carcinoma)
14. Participants with a prior history of pancreatitis, total gastrectomy or evidence of abnormal pancreatic function defined as elevated amylase and/or lipase > 2 times upper limit of normal
15. Participants diagnosed with Human Immunodeficiency Virus (HIV), clinically significant chronic hepatitis such as hepatitis B (HBV) or hepatitis C (HCV), or clinical history or signs of an active infection
16. Participants with a history of drug or alcohol abuse ≤ 5 years
17. Participants who have an active medical or psychiatric condition that in the opinion of the Site Investigator should preclude study participation
18. Participants with previous surgical management for Parkinson's disease
19. Participants participating in any drug or device clinical investigation concurrently or within 30 days prior to screening for this study
20. Participants with severe lactose and galactose intolerance
21. Participants with a known hypersensitivity or contraindication to study drugs (nilotinib or matching placebo) or their components.
22. Female participants of child-bearing potential. Female participants must be post-menopausal, post-hysterectomy, or have a documented infertility based on a known medical or surgical condition

Exclusion criteria specific for <u>Cohort 1:</u>

23a. Participants with a diagnosis of dementia based on the clinician's assessment, or a MoCA score < 21 at baseline

Exclusion criteria specific for <u>Cohort 2:</u>

23b. Participants with a MoCA score < 26 at baseline
24b. Participants treated within 60 days prior to randomization or expected to require treatment within 3 months from randomization with any ST (including levodopa, dopamine agonists and MAO-B inhibitors)

a. Treatment with amantadine or an anticholinergic agent will be allowed provided the dose has been stable for 30 days prior to screening and will remain stable for the duration of the study

4.4 DISCUSSION OF PARTICIPANT CHARACTERISTICS

The inclusion criteria were developed to select a study population that will optimally address the study specific objectives. The exclusion criteria were selected to further define the study population and to minimize putting the Participant's safety at risk. Exclusion criteria include all the warnings and precautions as guided by the nilotinib package insert Nilotinib (Tasigna®, AMN107, Novartis, Switzerland) revised 12/2017. See Appendix 1 and https://www.pharma.us.novartis.com/sites/www.pharma.us.novartis.com/files/tasigna.pdf

4.4.1 WARNINGS/PRECAUTIONS

Refer to the Tasigna package insert for the detailed discussion of the safety profile, warnings and precautions (revised 12/2017). See Appendix 1 and https://www.pharma.us.novartis.com/sites/www.pharma.us.novartis.com/files/tasigna.pdf

The following serious adverse reactions can occur with nilotinib (discussed in detail in the Package Insert)

- QT prolongation
- Myelosuppression
- Sudden Deaths
- Cardiac and Arterial Vascular Occlusive Events
- Pancreatitis and Elevated Serum Lipase
- Hepatotoxicity
- Electrolyte Abnormalities
- Hemorrhage
- Fluid Retention
- Embryo-fetal toxicity

Warnings and Precautions [from Tasigna® (nilotinib) package insert (revised 12/2017)]. See Appendix 1 and
https://www.pharma.us.novartis.com/sites/www.pharma.us.novartis.com/files/tasigna.pdf

Nilotinib WARNING: QT Prolongation and Sudden Deaths:

- Nilotinib prolongs the QT interval. Prior to nilotinib administration and periodically, monitor for hypokalemia or hypomagnesemia and correct deficiencies. Obtain electrocardiograms (ECGs) to monitor the QTc at baseline, 7 days after initiation, and periodically thereafter, and following any dose adjustments.

- Sudden deaths have been reported in patients receiving nilotinib. Do not use nilotinib in patients with hypokalemia, hypomagnesemia, or long QT syndrome.

- Avoid use of concomitant drugs known to prolong the QT interval and strong CYP3A4 inhibitors. Patient should avoid food 2 hours before and 1 hour after taking a nilotinib dose.

QT Prolongation

Nilotinib has been shown to prolong cardiac ventricular repolarization as measured by the QT interval on the surface ECG in a concentration-dependent manner. Prolongation of the QT interval can result in a type of ventricular tachycardia called torsade de pointes, which may result in syncope, seizure, and/or death. ECGs should be performed at baseline, 7 days after initiation of nilotinib, and periodically as clinically indicated and following dose adjustments *[see SOA]*.

Nilotinib should not be used in patients who have hypokalemia, hypomagnesemia or long QT syndrome. Before initiating nilotinib and periodically, test electrolyte, calcium and magnesium blood levels. Hypokalemia or hypomagnesemia must be corrected prior to initiating nilotinib and these electrolytes should be monitored periodically during therapy *[see SOA]*.

Significant prolongation of the QT interval may occur when nilotinib is inappropriately taken with food and/or strong CYP3A4 inhibitors and/or medicinal products with a known potential to prolong QT. Therefore, co-administration with food must be avoided and concomitant use with strong CYP3A4 inhibitors and/or medicinal products with a known potential to prolong QT should be avoided *[see appendix)]*. The presence of hypokalemia and hypomagnesemia may further prolong the QT interval *[see Exclusion criteria]*.

Sudden Deaths

Sudden deaths have been reported in 0.3% of patients with CML treated with nilotinib in clinical studies of 5,661 patients. The relative early occurrence of some of these deaths

relative to the initiation of nilotinib suggests the possibility that ventricular repolarization abnormalities may have contributed to their occurrence.

Cardiac and Arterial Vascular Occlusive Events

Cardiovascular events, including arterial vascular occlusive events, were reported in a randomized, clinical trial in newly diagnosed CML patients and observed in the post-marketing reports of patients receiving nilotinib therapy. With a median time on therapy of 60 months in the clinical trial, cardiovascular events, including arterial vascular occlusive events, occurred in 9.3% and 15.2% of patients in the nilotinib 300 and 400mg bid arms, respectively. These included cases of cardiovascular events including ischemic heart disease-related cardiac events (5.0% and 9.4% in the nilotinib 300mg and 400mg bid arms respectively, peripheral arterial occlusive disease (3.6% and 2.9% in the nilotinib 300mg and 400mg bid arms respectively, and ischemic cerebrovascular events (1.4% and 3.2% in the nilotinib 300mg and 400mg bid arms respectively. If acute signs or symptoms of cardiovascular events occur, advise patients to seek immediate medical attention. The cardiovascular status of patients should be evaluated and cardiovascular risk factors should be monitored and actively managed during nilotinib therapy according to standard guidelines *[see Exclusion criteria and SOA]*.

Myelosuppression

Treatment with nilotinib can cause Grade 3/4 thrombocytopenia, neutropenia and anemia. Perform complete blood counts every 2 weeks for the first 2 months and then monthly thereafter, or as clinically indicated *[see SOA]*. Myelosuppression was generally reversible and usually managed by withholding nilotinib temporarily or dose reduction.

Pancreatitis and Elevated Serum Lipase

Nilotinib can cause increases in serum lipase. Patients with a previous history of pancreatitis may be at greater risk of elevated serum lipase. *[See Exclusion criteria]*

Hepatotoxicity

Nilotinib may result in hepatotoxicity as measured by elevations in bilirubin, AST/ALT, and alkaline phosphatase. Monitor hepatic function tests monthly or as clinically indicated *[see SOA]*.

Electrolyte Abnormalities

The use of nilotinib can cause hypophosphatemia, hypokalemia, hyperkalemia, hypocalcemia, and hyponatremia. Correct electrolyte abnormalities prior to initiating nilotinib and during therapy. Monitor these electrolytes periodically during therapy *[see Exclusion criteria and SOA]*.

Hepatic Impairment

Nilotinib exposure is increased in patients with impaired hepatic function *[see Exclusion criteria and SOA]*.

Hemorrhage
In a randomized trial in patients with newly diagnosed Ph+ CML in chronic phase comparing nilotinib and imatinib, Grade 3 or 4 hemorrhage occurred in 1.1% of patients in the nilotinib 300mg bid arm, in 1.8% patients in the nilotinib 400mg bid arm, and 0.4% of patients in the imatinib arm. GI hemorrhage occurred in 2.9% and 5.1% of patients in the nilotinib 300mg bid and 400mg bid arms and in 1.4% of patients in the imatinib arm, respectively. Grade 3 or 4 events occurred in 0.7% and 1.4% of patients in the nilotinib 300mg bid and 400mg bid arms, respectively, and in no patients in the imatinib arm.

Monitoring Laboratory Tests
Complete blood counts should be performed every 2 weeks for the first 2 months and then monthly thereafter. Perform chemistry panels, including electrolytes, calcium, magnesium, liver enzymes, lipid profile, and glucose prior to therapy and periodically. ECGs should be obtained at baseline, 7 days after initiation and periodically thereafter, as well as following dose adjustments *[see Warnings and Precautions (5.2)]*. Monitor lipid profiles and glucose periodically during the first year of nilotinib therapy and at least yearly during chronic therapy. Should treatment with any HMG-CoA reductase inhibitor (a lipid lowering agent) be needed to treat lipid elevations, evaluate the potential for a drug-drug interaction before initiating therapy as certain HMG-CoA reductase inhibitors are metabolized by the CYP3A4 pathway [see Precautionary/Exclusionary Drugs Appendix 2]. Assess glucose levels before initiating treatment with nilotinib and monitor during treatment as clinically indicated. If test results warrant therapy, physician should follow their local standards of practice and treatment guidelines.

Embryo-Fetal Toxicity
There are no adequate and well controlled studies of nilotinib in pregnant women. Therefore, women of child-bearing potential will be excluded from the study *[see Exclusion criteria]*.

Fluid Retention

In the randomized trial in patients with newly diagnosed Ph+ CML in chronic phase, severe (Grade 3 or 4) fluid retention occurred in 3.9% and 2.9% of patients receiving nilotinib, 300mg bid and 400mg bid, respectively, and in 2.5% of patients receiving imatinib. Monitor patients for signs of severe fluid retention (e.g., unexpected rapid weight gain or swelling) and for symptoms of respiratory or cardiac compromise (e.g., shortness of breath) during nilotinib treatment; evaluate etiology and treat patients accordingly.

<u>Other warnings and precautions (discussed in detail in the Package Insert)</u>

Total gastrectomy

Since the exposure of nilotinib is reduced in patients with total gastrectomy, participants with gastrectomy will be excluded *[see Exclusion criteria)]*.

Lactose Intolerance

Since the capsules contain lactose, nilotinib is not recommended for patients with rare hereditary problems of galactose intolerance, severe lactase deficiency with a severe degree of intolerance to lactose-containing products, or of glucose-galactose malabsorption *[see Exclusion criteria]*.

Musculoskeletal Symptoms after Discontinuation

Patients taking nilotinib reported more frequent musculoskeletal symptoms (e.g., myalgia, pain in extremity, arthralgia, bone pain or musculoskeletal pain during the first year of discontinuation of treatment than before discontinuation.

Adverse events: Clinical trial experiences in CML population

The data below reflect exposure to nilotinib from a randomized trial in patients with newly diagnosed Ph+ CML in chronic phase treated at the recommended dose of 300mg twice-daily (n=279). The median time on treatment in the nilotinib 300mg twice-daily group was 61 months (range 0.1 to 71 months). The median actual dose intensity was 593mg/day in the nilotinib 300mg twice-daily group.

The most common (>10%) non-hematologic adverse drug reactions were rash, pruritis, headache, nausea, fatigue, alopecia, myalgia, and upper abdominal pain. Constipation, diarrhea, dry skin, muscle spasms, arthralgia, abdominal pain, peripheral edema, vomiting, and asthenia were observed less commonly (≤10% and>5%) and have been of mild to moderate severity, manageable and generally did not require dose reduction.

Increase in Fridericia's correction for QT interval (QTcF) >60 msec from baseline was observed in 1 patient (0.4%) in the 300mg twice-daily treatment group. No patient had an absolute QTcF of >500 msec while on study drug.

The most common hematologic adverse drug reactions (all grades) were myelosuppression including: thrombocytopenia (18%), neutropenia (15%) and anemia (8%). Discontinuation due to adverse reactions, regardless of relationship to study drug, was observed in 10% of patients.

In patients with CML-AP, the most commonly reported non-hematologic adverse drug reactions (≥10%) were rash, pruritus and fatigue. The common serious adverse drug reactions (≥1% and <10%) were thrombocytopenia, neutropenia, febrile neutropenia, pneumonia, leukopenia, intracranial hemorrhage, elevated lipase and pyrexia.

Sudden deaths and QT prolongation were reported. The maximum mean QTcF change from baseline at steady-state was 10 msec. Increase in QTcF >60 msec from baseline was observed in 4.1% of the patients and QTcF of >500 msec was observed in 4 patients (<1%)

Nilotinib safety profile in PD population[9]

The safety profile of nilotinib in a PD population is limited, and is based on a single, small, open label study where 12 patients were treated for 24 weeks[9]. There were three SAEs. One participant in the 300mg nilotinib group was withdrawn at week 4 and diagnosed with myocardial infarct and left bundle block. One participant in the 300mg nilotinib group was hospitalized with urinary tract infection (UTI) and one participant in the 150mg group was hospitalized with pneumonia and UTI [9].

The spectrum of adverse events (AEs) reported in this small PD population included cases of mild confusion and hallucinations as well as anxiety and agitation, not reported in CML cohorts. Non-serious AEs included transient QTc prolongation>450ms but they returned to normal QTc range in subsequent visits (one participant in 150mg and one in 300mg group). Participants in the 150mg group reported three UTIs, two cases of pneumonia, one cold virus, one mild back pain, one mild headache, one mild dysgraphia, one mild left foot drag, one mild confusion, one mild hallucination, one mild paranoia, one mild agitation, one moderate anxiety, one mild incontinence, one moderate itching and one skin irritation. Participants in the 300mg group reported one incident of blurry vision, one diarrhea, one nausea, one mild fatigue, two generalized weakness, three UTIs, one pneumonia, one weight loss, one tooth extraction, one dizziness, two mild hallucinations, one mild paranoia, one mild crying episode, one mild urinary urgency, one mild cough and one eczematous lesion. With regard to the laboratory parameters, a total of three AEs below normal range of ALT level (<100%) were observed in the 150mg group nilotinib. Two AEs above normal range (>100%), including one ALT and one bilirubin elevations observed in the 150mg group nilotinib. Two AEs with transient increase of ALT and one AST above normal range (>100%) were observed in the 300mg group nilotinib but returned to normal by end of the study.

Contraindicated Medications (see Section 8.2 and Appendix 2 for the full list of contraindicated medications and potential drug interactions)

Nilotinib is a competitive inhibitor of CYP3A4, CYP2C8, CYP2C9, CYP2D6 and UGT1A1 *in vitro*, potentially increasing the concentrations of drugs eliminated by these enzymes. *In vitro* studies also suggest that nilotinib may induce CYP2B6, CYP2C8 and CYP2C9, and decrease the concentrations of drugs which are eliminated by these enzymes.

Avoid administration of nilotinib with agents that may increase nilotinib exposure (e.g., strong CYP3A4 inhibitors) or anti-arrhythmic drugs (including, but not limited to amiodarone, disopyramide, procainamide, quinidine and sotalol) and other drugs that may prolong QT interval (including, but not limited to chloroquine, clarithromycin, haloperidol, methadone, moxifloxacin and pimozide).

Drugs that affect gastric pH

Nilotinib has pH-dependent solubility, with decreased solubility at higher pH. Drugs such as proton pump inhibitors that inhibit gastric acid secretion to elevate the gastric pH may decrease the solubility of nilotinib and reduce its bioavailability. The concomitant use of proton pump inhibitors with nilotinib is not recommended and is prohibited in this study.

In healthy participants, no significant change in nilotinib PK was observed when a single 400mg dose of nilotinib was administered 10 hours after and 2 hours before famotidine (an H2 blocker). Therefore, when the concurrent use of a H2 blocker is necessary, it may be administered approximately 10 hours before and approximately 2 hours after the dose of nilotinib.

Administration of an antacid (aluminum hydroxide/magnesium hydroxide/simethicone) to healthy participants, 2 hours before or 2 hours after a single 400mg dose of nilotinib did not alter nilotinib PK . Therefore, if necessary, an antacid may be administered approximately 2 hours before or approximately 2 hours after the dose of nilotinib.

Food effects

The bioavailability of nilotinib is increased with food; high fat meals increase C_{max} and AUC by 112% and 82%, respectively, while a light meal increases C_{max} by 33 – 55% and increase AUC by 15-29%. Thus, nilotinib must not be taken with food. No food should be consumed for at least 2 hours before and for at least 1 hour after the dose is taken. Also, avoid grapefruit products and other products that are known to inhibit CYP3A4.

5. STUDY PROCEDURES

5.1 RECRUITMENT

The study will be conducted at approximately 25 clinical sites in US. Each site should have a qualified Site Investigator and a Study Coordinator. Each site is required to have an individual Institutional Review Board (IRB) approval. All Site Investigators should be trained and qualified in the administration of the MDS-UPDRS and will be required to provide certification of training on the MDS-UPDRS within the last 2 years. Training sessions will be performed at the Investigator's meeting with special emphasis on the primary outcome measures. New sites and/or new Site Investigators identified after the Investigator's meeting will be trained individually. Enrollment will be closed as soon as the target number of participants are enrolled. Information regarding the clinical trial will be posted on multiple websites including clinicaltrials.gov and the Michael J. Fox Foundation (MJFF) website, Fox Trial Finder. A detailed recruitment plan will be provided.

Once a study site has obtained IRB approval, they can start identifying potential participants for the study. Participants should be community dwelling. All participants asked to participate in this trial will be tracked on a Confidential Participant Log, which is to be kept in a secure

location by the site study staff. This information is necessary to establish that the patient population was selected without bias. Information regarding how participants learned about the trial, referral sources will be tracked in source documents.

Only participants who meet all the inclusion and none of the exclusion criteria will be eligible to participate in the study. Reasons for ineligibility and reasons for non- participation for eligible participants will be tracked in both source documents and Electronic Case Report Form (eCRF) for all participants who have signed a consent and are screened for the study. No protocol waivers will be granted for participants unable to meet eligibility criteria. However, a one-time rescreening may be allowed on a case-by-case basis when potential for an improvement or recovery of the criteria for ineligibility is possible and the participant could become eligible for enrollment in the study. Participants may also rescreen if they were determined to be a screen failure prior to protocol Amendment 1 and are now deemed elegible based on Amendment 1 includsion/exclusion criteria.

During or before a screening visit, the Participant will be thoroughly informed about all aspects of the study, including all scheduled visits and activities, and will be able to ask questions. The participant will be requested to sign and date the informed consent form prior to undergoing any study-specific procedures. The original signed and dated informed consent form must be retained by the Site Investigator and a copy must be provided to the Participant.

Refer to the study recruitment plan for central and site-specific strategies for effective recruitment.

6. SCHEDULE OF ACTIVITIES

Throughout the study, it is highly recommended that assessments be performed in the same order as outlined in the Study Procedures section of this protocol at every visit and, if possible, at a similar time of day. Site staff that have been trained and approved to administer these assessments for this study must perform them, per delegation of authority log.

The Site Investigator is responsible for the overall conduct of the study and for conducting specific study assessments to ensure consistency in ratings and performance over the course of the study. Sub-Investigators will be allowed, provided that there is consistency in the personnel performing the ratings over the course of the study (i.e., MDS-UPDRS is performed by the same investigator at each visit throughout the study).

In this study, the Site Investigator will be responsible for the following:

- Determining Participant eligibility
- Conducting the physical (medical) and neurological examinations
- Conducting the MDS-UPDRS Part IA, Part III and Part IV

- Conducting fundoscopic exam prior to lumbar puncture
- Assessing Hoehn and Yahr stage
- Assessing Modified Schwab and England Activities of Daily Living
- Assessing Clinical Global Impression scale
- Determining need for ST (cohort 2)
- Ensuring Participant safety
- Assessing blindedness to treatment assignment and probability of PD as the accurate diagnosis, after a Participant discontinues study drug

6.1 PARTICIPANT IDENTIFICATION (ID) NUMBER

A participant ID Number will be assigned in sequential order by the site from a list provided to the site by the Clinical Trials Coordination Center (CTCC). This 4-digit number will be used to identify the participant on all study forms and safety lab specimens (as appropriate). Research specimens will be labeled per protocol (alternate labeling without the participant ID number, such as barcode labels, may be required to protect confidentiality).

Cohort 1 will enroll participants with moderate to advanced PD. After all participants in Cohort 1 complete the treatment period (throughV04), a planned interim analysis will determine whether either of the doses are sufficiently safe and tolerated to justify further study. If so, then the study steering committee will select one of the two doses for Cohort 2. Cohort 2 will enroll early/de novo PD participants. Enrollment into Cohort 2 will not begin until/if a dose is determined from Cohort 1.

Sections 6.2 through 6.11 pertain to Cohort 1. Sections 6.12 through 6.25 pertain to Cohort 2.

Schedule of activities for Cohort 1

6.2 SCREENING VISITS (SC1/SC2): COHORT 1

Participants will be assessed for study eligibility by the Site Investigator.

Prior to performing any study-specific procedures, the Site Investigator, designated Sub-Investigator or Study Coordinator will thoroughly explain to the Participant all aspects of the study, including all scheduled visits, activities and procedures, potential risks involved and that their participation is voluntary. Once the Participant has read and sign/dated the IRB-approved informed consent form, the procedures outlined below may begin. All Participants will be given a photocopy of the signed consent form.

Screening activities for this cohort will include two screening visits at the clinical site (Visit SC1 and SC2):

- Screening Visit 1 (SC1) which will take place **within 14 to 28 days prior** to the Baseline (BL) Visit.
- Screening Visit 2 (SC2) which will take place within 7 to 14 days prior to the Baseline (BL) Visit.

For the Participant to be randomized, all the inclusion criteria must be met and none of the exclusion criteria may apply. All results from the screening visit must be available and assessed before the final determination of a participant's eligibility for the study, which will be made before the baseline visit (BL).

Promptly after receipt of results from the screening activities, site staff will contact Participants to inform them either of exclusionary results that preclude their enrollment, or of their continued eligibility and to confirm their SC2 and BL visit appointment.

6.2.1 SCREENING VISIT 1 - SC1: COHORT 1

The following procedures and evaluations are to be performed at the **Screening Visit (SC1):**

Note: Assessments should be completed in the following order as closely as possible to ensure that the neurological tests are performed at approximately the same time (mornings) on each testing day:

- Obtain signed, written informed consent and permission to use Protected Health Information
- Assign Participant Entry ID
- Assign Unique Participant ID
- Determine eligibility through an assessment of inclusion/exclusion criteria
- Obtain demographic data and socio-economic information (highest level of education attained, marital status, handedness)
- Obtain vital signs, including height, weight and orthostatic vital signs (blood pressure, heart rate after participant has been seated for 1-3 minutes). Orthostatic vital signs are serial measurements of blood pressure and pulse that will be taken in supine position and standing position after 2 minutes
- Obtain medical & neurological history: include diagnosis and history of PD with dates
- Administer Beck Depression Inventory-II (BDI-II)
- Administer Montreal Cognitive Assessment (MoCA)
- Obtain list of concomitant medications; record:

  - Prescription and Over the Counter (OTC) medications
  - Vitamins and herbal supplements
  - Alcohol, tobacco and marijuana
- Perform physical examination (by Site Investigator)
- Perform neurological examination (by Site Investigator)
- Perform fundoscopy exam (by Site Investigator)
- Administer Hoehn and Yahr Scale
- Complete primary diagnosis form
- Perform 12-lead electrocardiogram (ECG)
- Complete Treatment Expectation Questionnaire
- Determine PD (diagnostic) features
- Administer Columbia Suicide Severity Rating Scale (C-SSRS) - **'Baseline'** version
- Collect blood for safety lab tests: Complete Blood Count (CBC), comprehensive chemistry panel, lipid panel, serum lipase and amylase
- Collect blood for coagulation parameters
- Collect blood for pregnancy test (for all women unless they are surgically sterile)
- Collect blood for HIV/HBV/HCV testing

The SC 1 visit will be approximately 4 hours in duration.

6.2.2 RESCREENING

Rescreening will be permitted for the following reasons:

1. At the discretion of the Clinical Monitor 30 days (+/- 7days) of Screening Visit 1 for laboratory and other reversible variables.

2. If a participant screen fails prior to protocol Amendment 1 and is determined to be eligible based on Amendment 1 inclusion/exclusion criteria. This visit may occur any time after the intial Screening Visit 1.

6.2.3 SCREENING VISIT 2- SC2: COHORT 1

- Participant will return to clinic between **<u>7 and 14 days prior to the Baseline Visit</u>** for an initial lumbar puncture. The procedure can be performed once coagulation parameters, other safety labs from Screening Visit 1 and fundus exam results have been reviewed, are determined to be within normal limits and participant continues to qualify for the study. Perform lumbar puncture to collect CSF for biomarker panel.

- CSF sample will be processed by the site's local lab facility (unless the lab is not able to process the CSF within 4 hours) to evaluate protein and glucose levels (routine chemistry) and cell count;
- CSF samples will be processed by specialty lab for biomarker panel

The SC2 visit will be approximately 45 minutes in duration

6.3 BASELINE VISIT – BL (DAY 0) – COHORT 1

Participant will return to clinic for BL assessments, randomization and first dose of study drug. Participants should be instructed to NOT take their PD medications at home on the morning of the study visit and bring their PD medication to clinic.

Participant should have abstained from PD medication(s) for approximately 12 hours prior to visit. Participants should be examined in the medications OFF and ON state. PD medications should be taken after completion of MDS-UPDRS OFF exam.

MDS- UPDRS Part III assessment should be completed in the defined medications OFF state (approximately 12 hours after the last dose of ST) **and** in medications ON state (based on the participant/ Site Investigator defined BEST ON and/or approximately 1 hour after dose of ST). MDS-UPDRS Parts I and II are to be completed in the ON state. Part IV is historical information.

Assessments should be completed in the order presented below as closely as possible to ensure that the neurological tests are performed at approximately the same time (mornings) on each testing day:

- Review of inclusion/exclusion criteria to ensure Participant remains eligible
- Administer Movement Disorders Society Unified Parkinson's Disease Rating Scale (MDS-UPDRS) Part III OFF (approximately 12 hours after the last dose of ST)
- Allow the Participant to take their regular dose of PD medications
- Review concomitant medication use since last visit. Record changes and additions as follows:
    - Prescription and OTC medications
    - Vitamins and herbal supplements
    - Alcohol, tobacco and marijuana
- Inquire and record information regarding adverse events (AEs)
- Obtain vital signs, including orthostatic vital signs (blood pressure, heart rate after participant has been seated for 1-3 minutes)

- Administer MDS-UPDRS Parts I-III in ON state (based on the participant/ Site Investigator defined BEST ON and/or approximately 1 hour after dose of ST).
- Administer MDS-UPDRS Part IV (historical information)
- Perform physical examination (by Site Investigator)
- Perform 12-lead electrocardiogram (ECG)
- Administer Modified Schwab & England Activities of Daily Living
- Perform Clinical Global Impression of severity (CGI) (Site Investigator and Participant)
- Administer Mattis Dementia Scale
- Administer Parkinson Disease Quality of Life Questionnaire (PDQ-39) (self- administered by Participant)
- Administer European Quality of Life Scale (EQ-5D) (self-administered by Participant)
- Administer Parkinson Disease Sleep Scale (PDSS)
- Complete Caffeine and Tobacco Exposure questionnaires
- Complete Treatment Expectation Questionnaire
- Collect blood for biomarker panel

- Randomize participant: If the participant continues to meet all eligibility requirements per the Site Investigator's final assessment, the participant may be randomized to treatment. Upon successful completion of randomization, the EDC system will generate the Participant's 5-digit enrollment/randomization number, which will be used to identify the Participant's appropriate drug kit.

  Under no circumstances may study drug be pulled from storage and given to a Participant unless that drug kit has been assigned to that Participant via the enrollment process.

  The coordinator will record the Participant's initials and Participant ID Number on the drug kit when it is taken from storage.
- Dispense one (1) bottle A and one (1) bottle B from the kit assigned to the Participant, which is a 3-month supply of study drug. Observe participant for approximately 30 minutes after taking first dose of study drug.
- Record initial dose on the Dose Management Log
- Educate Participant regarding dosing restrictions.
- **Provide instructions regarding study drug covering:**
  - Storage requirements
  - Importance of any specific Participant instructions
- **Study drug should be taken at the same time every day in the morning unless otherwise instructed. Study drug should be taken on empty stomach. No food should be consumed for at least 2 hours before and for at least 1 hour after the dose is taken.**

- Always take capsules with a glass of water or unsweetened, non-alcoholic beverage;
- Participant should avoid grapefruit, including beverages or any supplements containing grapefruit for the duration of the study.
- Ensure if participant takes antacids, they are not taken sooner than 2 hours before taking study drug and not for at least 2 hours afterwards
- Return of <u>all</u> study drug bottles at each visit (whether empty, partially used, or unused)
- When applicable, importance of not taking any study medication on designated study visit days until after assessments or blood draw.

- Stress to Participant the importance of calling site to report any AEs or for any study questions

BL visit will be approximately 4-6 hours in duration

6.4 SAFETY VISITS SV1 THROUGH SV4 – COHORT 1

Safety Visit 1 (Day 7 ±3d) – SV1
Safety Visit 2 ((Day 60 ± 7d) – SV2
Safety Visit 3 ((Day 120 ± 7d) – SV3
Safety Visit 4 ((Day 150 ± 7d) – SV4

Additional assessments for individual visits are listed below.
Note: Blood collection parameters vary for each Safety visit.

- Review concomitant medication use since last visit. Record changes and additions as follows:
  - Prescription and OTC medications
  - PD Medications – if dose increased, MDS-UPDRS ON required
  - Vitamins and herbal supplements
  - Alcohol, tobacco and marijuana
- Inquire and record information regarding adverse events (AEs)
- Ensure dose management log is maintained appropriately; clarify issues with participant during visit; re-train Participant if necessary.
- Complete study drug accountability and compliance check on all returned study drug bottles (empty, partially used or unused); clarify issues with Participant during visit; re-train participant if necessary.
- Obtain vital signs, including orthostatic vital signs (blood pressure, heart rate after participant has been seated for 1-3 minutes)
- Perform 12-lead electrocardiogram (ECG)

Additional assessments
Safety Visit 2 (Day 60 ± 7d)
Safety Visit 3 (Day 120 ± 7d)
Safety Visit 4 (Day 150 ± 7d)

- Collect blood for sparse PK analysis – **post-dose random sample** to be collected at any time relative to last dose of study drug- **SV2, SV3 and SV4**
- Collect blood for safety lab tests: CBC, comprehensive chemistry panel, lipid panel, serum lipase and amylase. - **SV2, SV3 and SV4**

The safety visits will be approximately 1 hour each in duration

6.5 VISIT 01 (DAY 14 ± 3D) – V01 – COHORT 1
VISIT 02 (DAY 30 ± 3D) – V02 – COHORT 1

Participant will return to clinic abstaining from taking study drug for 24 ± 2 hours in anticipation of sparse PK blood draw. Participant can take their regular dose of PD medication(s) the morning of their visit. Participant must bring both PD medication(s) and study drug to the visit.

Assessments should be completed in the order presented below as closely as possible to ensure that the neurological tests are performed at approximately the same time (mornings) on each testing day:

- Collect blood for safety lab tests: CBC, comprehensive chemistry panel
- Collect blood for sparse PK analysis – pre-dose trough sample to be collected 24 ± 2 hours post last dose of study drug.
- Allow the participant to take their study drug
- Review concomitant medication use since last visit. Record changes and additions as follows:
    - Prescription and OTC medications
    - Vitamins and herbal supplements
    - Alcohol, tobacco and marijuana
- Inquire and record information regarding adverse events (AEs)
- Obtain vital signs, including orthostatic vital signs (blood pressure, heart rate after participant has been seated for 1-3 minutes)

- Ensure dose management log is maintained appropriately; clarify issues with participant during visit; re-train Participant if necessary.
- Complete study drug accountability and compliance check on all returned study drug bottles (empty, partially used or unused); clarify issues with participant during visit; re-train participant if necessary.
- Administer MDS-UPDRS Parts I-III in ON state (based on the participant/site investigator defined best ON and/or approximately 1 hour after dose of ST)
- Administer MDS-UPDRS Part IV (historical information)

Additional assessment **– V1 only**
Visit 01 (Day 14 ± 3d)

- Determine dose titration

Additional assessment **– V2 only**
Visit 02 (Day 30 ± 3d)

- Collect blood for safety lab tests: lipid panel, serum lipase and amylase
- Perform 12-lead electrocardiogram (ECG)

Visits 01 and 02 will be approximately 2 hours in duration.

6.6 VISIT 03 (DAY 90 + 7D) – V03 – COHORT 1

Participant should be instructed to NOT take their PD medication(s) or their study drug at home on the morning of Visit 3 and bring their PD medication(s) and study drug to clinic.

Participant will return to clinic, abstaining from study drug for 24 ± 2 hours in anticipation of sparse PK blood draw.

Participant should bring study drug to the visit as CSF and corresponding serum PK will be collected 2 ± 0.5 hours post observed dose. Lumbar puncture must be conducted on the same day as the rest of the assessments for Visit 3. Coagulation parameters, collected within 10 days prior to the study visit, must be reviewed by the site investigator prior to performing the LP.

Participants should be examined in the medications OFF and ON state. PD medications should be taken after completion of MDS-UPDRS OFF exam. MDS-UPDRS Part III assessment should be completed in the defined medications OFF state (approximately 12 hours after the last dose of

ST) **and** in medications ON state (based on the participant/ Site Investigator defined BEST ON and/or approximately 1 hour after dose of ST). MDS-UPDRS Parts I and II are to be completed in the ON state. Part IV is historical information.

Assessments should be completed in the following order as closely as possible to ensure that the neurological tests are performed at approximately the same time (mornings) on each testing day:

- Administer MDS-UPDRS Part III OFF (approximately 12 hours after the last dose of ST).
- Collect blood for coagulation parameters for a stat read if the participant did not have their blood drawn and coagulation parameters tested in the last 10 days.
- Collect blood for safety lab tests: CBC, comprehensive chemistry panel, lipid panel, serum lipase and amylase.
- Collect blood for biomarker panel
- Collect blood for sparse PK analysis at 2 time points:
    - Sample 1: **pre-dose trough sample must be collected 24 ± 2 hours post last dose** of study drug.
    - Sample 2: **must be collected 2 ± 0.5 hours post observed dose** at the time of the collection of the CSF sample
- Allow the participant to take their study drug and regular dose of PD medications
- Review concomitant medication use since last visit. Record changes and additions as follows:
    - Prescription and OTC medications
    - Vitamins and herbal supplements
    - Alcohol, tobacco and marijuana
- Inquire and record information regarding adverse events (AEs)
- Perform physical examination (by Site Investigator)
- Perform fundoscopy exam (to be performed by the PI prior to the LP)
- Perform Clinical Global Impression of Change (CGI) (Site Investigator and participant)
- Administer Hoehn and Yahr Scale
- Administer Modified Schwab & England Activities of Daily Living
- Administer Mattis Dementia Scale
- Administer Parkinson Disease Quality of Life Questionnaire (PDQ-39) (self-administered by participant)
- Administer European Quality of Life Scale (EQ-5D) (self-administered by participant)
- Administer Columbia Suicide Severity Rating Scale (C-SSRS) – 'Since Last Visit' version
- Administer Parkinson Disease Sleep Scale (PDSS)
- Obtain vital signs, including height, weight and orthostatic vital signs (blood pressure, heart rate after participant has been seated for 1-3 minutes)

- Perform 12-lead electrocardiogram (ECG)
- Administer MDS-UPDRS Parts I-III in ON state (based on the participant/ Site Investigator defined BEST ON and/or approximately 1 hour after dose of ST)
- Administer MDS-UPDRS Part IV (historical information)
- Perform lumbar puncture to collect spinal fluid samples (**note**: all samples collected on same day):
  - PK sample must be collected **2 ± 0.5 hours post observed dose** of study drug - note: a corresponding blood sample must also be collected.
  - Biomarker sample
    - LP may be performed only after results of the coagulation lab parameters (collected at SV2 visit) and fundoscopic exam (performed by PI) have been reviewed and documented
    - CSF sample will be processed by the site's local lab facility (unless the lab is not able to process the CSF within 4 hours) to evaluate protein and glucose levels (routine chemistry) and cell count;
    - CSF samples will be process by specialty lab for biomarker panel. Refer to NILO-PD Lab Manual for specific instructions.
    - LP must be performed on the same day as the rest of the assessments for Visit 3.
- Complete IP accountability and compliance check on all returned IP bottles (empty, partially used, or unused)
- Ensure dose management log is maintained appropriately; clarify issues with participant during visit; re-train Participant if necessary.
- Dispense one (1) bottle A and one (1) bottle B from the original kit assigned to the participant, which is a 3-month supply of study drug. **Under no circumstances may study drug be pulled from storage and given to a participant unless that drug kit has been assigned to that participant via the enrollment process.**

The coordinator will record the participant's initials and participant ID Number on the drug kit when it is taken from storage.

V03 will be approximately 2 hours in duration.

6.7 VISIT 04 (DAY 180±7D) – V04 – COHORT 1 (FINAL "ON DRUG" VISIT)

This is the Participant's final visit on study drug, therefore all unused, empty and partially used bottles should be returned for final accountability at this visit.

Participant should be instructed to NOT take their PD medication(s) at home on the morning of Visit 4 and bring their PD medication(s) to clinic. Additionally, Participant should adjust the time in which they take their study drug to ensure a 2 to 5 hour period prior to sparse PK blood draw.

Participant must abstain from taking PD medication(s) for approximately 12 hours prior to their clinic visit. Participant should be examined in the medications OFF and ON state. PD medication(s) should be taken after completion of MDS-UPDRS OFF exam.

MDS-UPDRS Part III assessment should be completed in the defined medications OFF state (approximately 12 hours after the last dose of ST) **and** in medications ON state (based on the participant/ Site Investigator defined BEST ON and/or approximately 1 hour after dose of ST). MDS-UPDRS Parts I and II are to be completed in the ON state. Part IV is historical information.

Assessments should be completed in the following order as closely as possible to ensure that the neurological tests are performed at approximately the same time (mornings) on each testing day:

- Allow Participant to take their study drug, if they haven't previously taken their daily dose, ensuring a 2 to 5 hour period prior to PK blood collection,
- Administer MDS-UPDRS Part III OFF (approximately 12 hours after the last dose of ST)
- Allow Participant to take their regular dose of PD medications
- Review concomitant medication use since last visit. Record changes and additions as follows:
    - Prescription and OTC medications
    - Vitamins and herbal supplements
    - Alcohol, tobacco and marijuana
- Inquire and record information regarding adverse events (AEs)
- Perform physical examination by Site Investigator
- Perform neurological examination by Site Investigator
- Administer Clinical Global Impression of Change (CGI) (Site Investigator and Participant)
- Determine PD diagnostic features
- Complete primary diagnosis form
- Administer MDS-UPDRS Parts I-III in ON state (based on the participant/ Site Investigator defined BEST ON and/or approximately 1 hour after dose of ST)
- Administer MDS-UPDRS Part IV (historical information)

- Administer Mattis Dementia Scale
- Administer Parkinson Disease Quality of Life Questionnaire (PDQ-39) (self-administered by participant)
- Administer European Quality of Life Scale (EQ-5D) (self-administered by participant)
- Administer Columbia Suicide Severity Rating Scale (C-SSRS) – 'Since Last Visit' version
- Administer Parkinson Disease Sleep Scale (PDSS)
- Administer Blindedness questionnaires (Site Investigator, Coordinator and participant)
- Administer Treatment expectation questionnaire (self-administered by participant)
- Collect blood for PK;
    - **Sample must be collected 2-5 hours post-dose of study drug**
- Collect blood for safety lab tests: CBC, comprehensive chemistry panel, lipid panel, serum lipase and amylase
- Collect blood for coagulation parameters
- Collect blood for biomarker samples
- Administer Modified Schwab & England Activities of Daily Living
- Administer Hoehn and Yahr Scale
- Obtain vital signs, <u>including height, weight</u> and orthostatic vital signs (blood pressure, heart rate after participant has been seated for 1-3 minutes)
- Perform a 12-lead electrocardiogram (ECG)
- **Complete a <u>final</u> study drug accountability and compliance check on all returned study drug bottles (empty, partially used or unused)**
- Ensure dose management log is maintained appropriately; clarify issues with participant during visit.

V04 will be approximately 3 hours in duration.

6.8 VISIT 05 (30 DAYS POST-TREATMENT ± 7 D) – V05 – COHORT 1
VISIT 06 (60 DAYS POST-TREATMENT ± 7D) – V06 – END OF STUDY

Participant should be instructed to NOT take their PD medication(s) at home on the morning of Visit 4 and bring their PD medication(s) to clinic.

Participant will return to clinic abstaining from PD medications for approximately 12 hours. <u>Participants should be examined in the PD medications OFF and ON state.</u> PD medication should be taken after completion of MDS-UPDRS OFF exam.

MDS-UPDRS Part III assessment should be completed in the defined medications OFF state (approximately 12 hours after the last dose of ST) **and** in medications ON state (based on the participant/ Site Investigator defined BEST ON and/or approximately 1 hour after dose of ST). MDS-UPDRS Parts I and II are to be completed in the ON state. Part IV is historical information.

Assessments should be completed in the following order as closely as possible to ensure that the neurological tests are performed at approximately the same time (mornings) on each testing day:

- MDS-UPDRS Part III OFF (approximately 12 hours after the last dose of ST)
- Allow the participant to take their regular dose of PD medications

- Review concomitant medication use since last visit. Record changes and additions as follows:
    - Prescription and OTC medications
    - Vitamins and herbal supplements
    - Alcohol, tobacco and marijuana
- Inquire and record information regarding adverse events (AEs)
- Administer Hoehn and Yahr Scale
- Administer MDS-UPDRS Parts I-III in ON state (based on the participant/ Site Investigator defined BEST ON and/or approximately 1 hour after dose of ST)
- Administer MDS-UPDRS Part IV (historical information)
- Administer Treatment expectation questionnaire (participant)
- Obtain vital signs and orthostatic vital signs (blood pressure, heart rate after participant has been seated for 1-3 minutes)
- Collect blood for safety lab tests: CBC, comprehensive chemistry panel, lipid panel serum lipase and amylase

Additional assessments

Visit 05 (30 days post-treatment ± 7 d)

- Perform physical examination (by Site Investigator)
- Height and weight
- Perform a 12-lead electrocardiogram (ECG)
- Administer Blindedness questionnaires (Site Investigator, Coordinator and participant)
- Perform fundoscopy exam (to be performed by the PI prior to the LP)

- Perform lumbar puncture. (Lumbar puncture for this visit is strongly encouraged but not required)

    - LP may be performed only after results of the coagulation lab parameters (collected at V04 visit) and fundoscopic exam (performed by PI) have been reviewed and documented
    - CSF sample will be processed by the site's local lab facility (unless the lab is not able to process the CSF within 4 hours) to evaluate protein and glucose levels (routine chemistry) and cell count;
    - CSF samples will be process by specialty lab for biomarker panel;
    - LP may be performed on a separate day within the visit window, if necessary
- Collect blood for biomarker panel

V05 will be approximately 1.5 hours in duration.

Additional assessments

Visit 06 (60 Days post-treatment ± 7d)

- Columbia Suicide Severity Rating Scale (C-SSRS) - 'Since Last Visit' Version – only
- Collect blood for pregnancy test (for all women unless surgically sterile)

V06 will be approximately 2.5 hours in duration.

6.9 UNSCHEDULED VISITS – VISIT U01, U02, ETC. – COHORT 1

An unscheduled visit may be performed at any time during the study at the participant's request or as deemed necessary by the Site Investigator. The date and reason for the unscheduled visit or telephone contact will be recorded in the source documentation. Reasons for unscheduled visits may include, but are not limited to, a potential severe side effect, worsening of symptoms, multiple missed doses of medication.

Assessments should be completed in the following order as closely as possible to ensure that the neurological tests are performed at approximately the same time (mornings) on each testing day:

- Review concomitant medication use since last visit. Record changes and additions as follows:
    - Prescription and OTC medications
    - PD Medications – if dose increased, MDS-UPDRS ON required
    - Vitamins and herbal supplements
    - Alcohol, tobacco and marijuana
- Inquire and record information regarding adverse events (AEs)

- Administer Columbia Suicide Severity Rating Scale (C-SSRS) – 'Since Last Visit' version
- Obtain vital signs, including orthostatic vital signs (blood pressure, heart rate after participant has been seated for 1-3 minutes)
- Perform 12-lead electrocardiogram (ECG)
- Collect blood for safety lab tests: CBC, comprehensive chemistry panel
- Complete study drug accountability and compliance check on all returned study drug bottles (empty, partially used or unused)
- Ensure dose management log is maintained appropriately; clarify issues with participant during visit; re-train Participant if necessary.

An unscheduled visit may vary in duration.

6.10 PREMATURE WITHDRAWAL VISIT - VISIT PW – COHORT 1

Participants have the right to withdraw from the study at any time without prejudice. The Site Investigator may withdraw study drug from a participant in the study in the event of intercurrent illness, adverse events, other reasons concerning the health or well-being of the participant, or in the case of lack of cooperation, non-compliance, protocol violation or other administrative reasons. Premature withdrawal will be implemented in the case of emergency disclosure of drug treatment.

In the event of premature withdrawal from the study, the Premature Withdrawal (PW) Visit procedures and evaluations should be completed whether or not the withdrawal is determined at a regularly scheduled study visit or at an unscheduled visit. In instances where the participant refuses or cannot to return for a PW visit, it is acceptable to attempt to obtain data by telephone and arrange for return of study drug. It is important to collect the final date and time of day the last dose of study drug was taken.

Reasons for withdrawal of the Participant prior to completion of the study must be stated in the eCRF and in the site source documentation for all study participants who were enrolled in the study. The Participant will be encouraged to remain in the study and return for the 1 month and 2 month post drug evaluations (V05 and V06) as a safety follow up.

Adverse events will be followed for 30 days after a Participant's last dose of study drug. AEs, abnormal laboratory results and abnormal ECG values will be followed to resolution or stabilization, whichever occurs first, but no new AEs will be recorded.

The CTCC must be informed within 24 hours of all study Participants who prematurely withdraw from the study.

Participant should be instructed to bring both study drug and PD medications to the PW visit. It is important to collect the final date and time of day the last dose of study drug was taken.

Participants should be instructed to NOT take their PD medications at home on the morning of their PW visit and to bring their PD medication(s) the study visit. Participant must return to clinic abstaining from PD medications for approximately 12 hours. Participants should be examined in the medications OFF and ON state. Medication should be taken after completion of MDS-UPDRS OFF exam.

Lumbar puncture must be conducted on the same day as the rest of the PW visit assessments. Coagulation parameters, collected within 10 days prior to the study visit, must be reviewed by the site investigator prior to performing the LP.

MDS- UPDRS Part III assessment should be completed in the defined medications OFF state (approximately 12 hours after the last dose of ST) **and** in medications ON state (based on the participant/ Site Investigator defined BEST ON and/or approximately 1 hour after dose of ST). MDS-UPDRS Parts I and II are to be completed in the ON state. Part IV is historical information.

Assessments should be completed in the following order as closely as possible to ensure that the neurological tests are performed at approximately the same time (mornings) on each testing day:

- MDS-UPDRS Parts III OFF (approximately 12 hours after the last dose of ST)
- Allow the participant to take their PD medications
- Administer MDS-UPDRS Parts I-III in ON state (based on the participant/ Site Investigator defined BEST ON and/or approximately 1 hour after dose of ST)
- Administer MDS-UPDRS Part IV (historical information)
- Collect blood for PK only if the participants remained on the study drug or discontinued within the last 3 days;
    - If study drug was taken within past 3 days, collection of one **random sample** at any time relative to post-dose at each visit.
    - The date and time of day of the last dose of the study drug has to be documented in the eCRF
- Collect blood for pregnancy test (for all women unless surgically sterile)
- Collect blood for safety lab tests: CBC, comprehensive chemistry panel and lipid panel.
- Collect blood for coagulation parameters (results must be reviewed and documented prior to performing lumbar puncture)
- Collect blood for biomarker samples

- Review concomitant medication use since last visit. Record changes and additions as follows:
    - Prescription and OTC medications
    - Vitamins and herbal supplements
    - Alcohol, tobacco and marijuana
- Inquire and record information regarding adverse events (AEs)
- Perform physical examination by Site Investigator
- Perform neurological examination by Site Investigator
- Perform fundoscopy exam (to be performed by the PI prior to the LP)
- Administer Clinical Global Impression of Change (CGI) (Site Investigator and participant) –
- Determine PD diagnostic features
- Complete primary diagnosis form
- Administer Mattis Dementia Scale
- Administer Parkinson Disease Quality of Life Questionnaire (PDQ-39) (self-administered by participant)
- Administer Columbia Suicide Severity Rating Scale (C-SSRS) – 'Since Last Visit' version-only
- Administer European Quality of Life Scale (EQ-5D) (self-administered by participant)
- Administer Parkinson Disease Sleep Scale (PDSS)
- Administer Blindedness questionnaires (Site Investigator, Coordinator and participant)
- Administer Treatment expectation questionnaire (self-administered by participant)
- Administer Modified Schwab & England Activities of Daily Living
- Administer Hoehn and Yahr Scale
- Obtain vital signs, <u>including height, weight</u> and orthostatic vital signs (blood pressure, heart rate after participant has been seated for 1-3 minutes)
- Perform a 12-lead electrocardiogram (ECG)
- Perform lumbar puncture. (Lumbar puncture for this visit is strongly encouraged but not required)
    - LP may be performed only after results of the coagulation lab parameters and fundoscopic exam have been reviewed and documented
    - CSF sample will be processed by the site's local lab facility (unless the lab is not able to process the CSF within 4 hours) to evaluate protein and glucose levels (routine chemistry) and cell count;
    - CSF samples will be process by specialty lab for biomarker panel;
    - LP may be performed on a separate day within the visit window, if necessary

- **Complete a final study drug accountability and compliance check on all returned study drug bottles (empty, partially used or unused.)**
- Ensure dose management log is maintained appropriately; clarify issues with participant during visit.

PW will be approximately 3 hours in duration.

6.11 PROCEDURES FOR THE PARTICIPANTS WHO DISCONTINUE THE STUDY DRUG FOR SAFETY AND INTOLERABILITY REASONS

If a participant needs to discontinue study drug for safety reasons, the site should complete a Premature Withdrawal visit (section 6.10) either while still taking the study drug or as soon as possible after drug discontinuation. AEs will be followed for 30 days after a Participant's last dose of study drug. AEs, abnormal laboratory results and abnormal ECG values will be followed to resolution or stabilization, whichever occurs first, but no new AEs will be recorded. The participant will be encouraged to return for the 30 and 60 days post drug evaluations (V05 and V06) as per schedule of activities.

In case the study drug has been discontinued for safety laboratory values or ECG abnormalities reasons, ECG and abnormal laboratories values must be repeated in 2 weeks and at 30 days.

<u>Schedule of activities for Cohort 2</u>

NOTE: Cohort 2 will not start enrollment until/when a safe and tolerable dose of study drug is determined from Cohort 1 and study materials for Cohort 2 are available for distribution to sites.

6.12 SCREENING VISIT 1 (VISIT SC 1) – COHORT 2 (EARLY/DE NOVO PD)

Participants will be assessed for study eligibility by the Site Investigator.

Prior to performing any study-specific procedures, the Site Investigator or designated sub-investigator or study coordinator will thoroughly explain to the Participant all aspects of the study, including all scheduled visits, activities and procedures, potential risks involved and that their participation is voluntary. Once the Participant has read and sign/dated the IRB-approved informed consent form, the procedures outlined below may begin. All participants will be given a photocopy of the signed consent form.

Screening activities for this cohort will include one screening visit at the clinical site (Visit SC), which will take place **<u>within 28 to 14 days prior</u>** to the Baseline (BL) Visit.

For the Participant to be randomized, all the inclusion criteria must be met and none of the exclusion criteria may apply. All results from the screening visit must be available and assessed before the final determination of a participant's eligibility for the study, which will be made before the baseline visit (BL).

Promptly after receipt of results from the screening activities, site staff will contact participants to inform them either of exclusionary results that preclude their enrollment, or of their continued eligibility and to confirm their BL visit appointment.

The following procedures and evaluations are to be performed at the **Screening Visit (SC1):**

Note: Assessments should be completed in the following order as closely as possible to ensure that the neurological tests are performed at approximately the same time (mornings) on each testing day:

- Obtain signed, written informed consent and permission to use Protected Health Information
- Assign participant Entry ID
- Assign Unique participant ID
- Determine eligibility through an assessment of inclusion/exclusion criteria
- Obtain demographic data and socio-economic information (highest level of education attained, marital status, handedness)
- Administer Beck Depression Inventory-II (BDI-II)
- Administer Montreal Cognitive Assessment (MoCA©)
- Obtain vital signs, including height, weight and orthostatic vital signs (blood pressure, heart rate after participant has been seated for 1-3 minutes)
- Obtain medical & neurological history: include diagnosis and history of PD with dates
- Obtain list of concomitant medications; record:
    - Prescription and OTC medications
    - Vitamins and herbal supplements
    - Alcohol, tobacco and marijuana
- Perform physical examination (by Site Investigator)
- Perform neurological examination (by Site Investigator)
- Perform fundoscopy exam (by Site Investigator)
- Administer Hoehn and Yahr Scale
- Assess need for ST
- Complete primary diagnosis form
- Determine PD (diagnostic) features
- Administer Columbia Suicide Severity Rating Scale (C-SSRS) -'Baseline' version

- Perform 12-lead electrocardiogram (ECG)
- Complete Treatment Expectation Questionnaire

- Collect blood for safety lab tests: CBC, comprehensive chemistry panel, lipid panel, serum lipase and amylase
- Collect blood for coagulation parameters
- Collect blood for pregnancy test (for all women unless they are at least two years postmenopausal, or surgically sterile)
- Collect blood for HIV, HBV, HCV testing

The SC 1 visit will be approximately 2 to 3 hours in duration.

6.12.1 RESCREENING

Rescreening will be permitted at the discretion of the Clinical Monitor within 30 days (+/- 7 days) of the original screening visit for the laboratory and other reversible variables. Only out of range parameters will have to be repeated.

Participants may also rescreen if they were determined to be a screen failure prior to protocol Amendment 1 and are now deemed elegible based on Amendment 1 includsion/exclusion criteria.

6.13 SCREENING VISIT 2

Participant will return to clinic between 7 to 14 days prior to the Baseline Visit for an initial lumbar puncture. The procedure can be performed once coagulation parameters, other safety labs from Screening Visit 1 and fundus exam results have been reviewed and are normal and participant continues to qualify for the study.

- Perform lumbar puncture to collect CSF for biomarker panel.
    - CSF sample will be processed by the site's local lab facility (unless the lab is not able to process the CSF within 4 hours) to evaluate protein and glucose levels (routine chemistry) and cell count;
    - CSF samples will be process by specialty lab for biomarker panel

The SC 2 visit will be approximately 45 minutes in duration

6.14 BASELINE VISIT – BL (DAY 0) – COHORT 2

Participant will return to clinic for BL assessments, randomization and first dose of study drug. Assessments should be performed in the order presented below if possible.

Assessments should be completed in the order presented below as closely as possible to ensure that the neurological tests are performed at approximately the same time (mornings) on each testing day:

- Review of inclusion/exclusion criteria to ensure participant remains eligible
- Review concomitant medication use since last visit. Record changes and additions as follows:
    - Prescription and OTC medications
    - Vitamins and herbal supplements
    - Alcohol, tobacco and marijuana
- Inquire and record information regarding adverse events (AEs)
- Obtain vital signs and orthostatic vital signs (blood pressure, heart rate after participant has been seated for 1-3 minutes)
- Perform physical examination (by Site Investigator)
- Perform 12-lead electrocardiogram (ECG)
- Administer Modified Schwab & England Activities of Daily Living
- Perform Clinical Global Impression of Severity(CGI) (Site Investigator and participant)
- Administer Mattis Dementia Scale
- Administer Parkinson Disease Quality of Life Questionnaire (PDQ-39) (self- administered by participant)
- Administer European Quality of Life Scale (EQ-5D) (self-administered by participant)
- Administer Parkinson Disease Sleep Scale (PDSS)
- Complete Treatment Expectation Questionnaire
- Complete Environmental Questionnaire
- Administer MDS-UPDRS Parts I-III
- Collect blood for biomarker panel
- Randomize participant: If the participant continues to meet all eligibility requirements per the Site Investigator's final assessment, the participant will be randomized to treatment via the web-based enrollment module (see Section 6.1 for information regarding enrollment procedures). Upon successful completion of randomization, the EDC system will generate

the participant's 4-digit enrollment/randomization number, which will be used to identify the participant's appropriate drug kit.

Under no circumstances may study drug be pulled from storage and given to a participant unless that drug kit has been assigned to that participant via the enrollment process.

The coordinator will record the participant's initials and participant ID Number on the drug kit when it is taken from storage.

- Dispense a 3-month supply of study drug as outlined in Section 7.8. Observe participant for approximately 30 minutes after taking first dose of study drug.
- Record initial dose on the Dose Management Log
- Educate Participant regarding dosing restrictions.

- **Provide instructions regarding study drug covering:**
    - Storage requirements
    - Importance of any specific patient instructions,
    - **Study drug should be taken at the same time every day in the morning unless otherwise instructed. Participants should avoid eating food for at least 2 hours before the dose is taken and avoid eating food for at least 1 hour after the dose is taken.**
    - Always take capsules with a glass of water or unsweetened, non-alcoholic beverage;
    - Participant should avoid grapefruit, including beverages or any supplements containing grapefruit for the duration of the study. Ensure if participant takesH2 blockers, they are not taken sooner than 10 hours before taking study drug and not for at least 2 hours afterwards.
    - Ensure if participant takes an antacid, they are not taking these medications for about 2 hours before the study drug is taken or for at least 2 hours after study drug is taken.
    - Return of <u>all</u> study drug bottles at each visit (whether empty, partially used, or unused)
    - When applicable, importance of not taking any study medication on designated study visit days until after assessments or blood draw.
- Stress to participant the importance of calling site to report any AEs or for any study questions

BL visit will be approximately 2-4 hours in duration (timing dependent on when lumbar puncture is performed).

6.15 SAFETY VISITS - SV 1 THROUGH SV 6 – COHORT 2

Safety Visit 1 (Day 7 ± 3d) – SV1
Safety Visit 2 (Day 60 ± 7d) – SV2
Safety Visit 3 (Day 120 ± 7d) – SV3
Safety Visit 4 (Day 150 ± 7d) – SV4
Safety Visit 5 (Day 210 ± 7d) - SV5
Safety Visit 6 (Day 240 ± 7d) – SV6

Additional assessments for individual visit are listed below.

- Review concomitant medication use since last visit. Record changes and additions as follows:
    - Prescription and OTC medications
    - PD Medications – if dose increased, MDS-UPDRS ON required – **SV03, SV04, SV05, SV06**
    - Vitamins and herbal supplements
    - Alcohol, tobacco and marijuana
- Inquire and record information regarding adverse events (AEs)
- Ensure dose management log is maintained appropriately; clarify issues with participant during visit; re-train participant if necessary.
- Complete study drug accountability and compliance check on all returned study drug bottles (empty, partially or unused); clarify issues with participant during visit; re-train participant if necessary.
- Vital signs and orthostatic vital signs (blood pressure, heart rate after participant has been seated for 1-3 minutes)
- Perform 12-lead electrocardiogram (ECG)

Additional assessments
Safety Visit 2 (Day 60 ± 7d)
Safety Visit 3 (Day 120 ± 7d)
Safety Visit 4 (Day 150 ± 7d)
Safety Visit 5 (Day 210 ± 7d)
Safety Visit 6 (Day 240 ± 7d)

- Collect blood for safety lab tests: CBC, comprehensive chemistry panel, lipid panel, serum lipase and amylase
- Collect blood for sparse PK analysis – **post-dose random sample** to be collected at any time relative to last dose of study drug.

The safety visits will be approximately 1 hour each in duration

6.16 VISIT 01 (DAY 14 ± 3D) – V01 – COHORT 2
VISIT 02 (DAY 30 ± 3D) – V02 – COHORT 2

Participant will return to clinic abstaining from taking study drug for 24 ± 2 hours in anticipation of the sparse PK blood draw. Participant must bring study drug to the visit.

Assessments should be completed in the order presented below as closely as possible to ensure that the neurological tests are performed at approximately the same time (mornings) on each testing day:

- Collect blood for safety lab tests: CBC, comprehensive chemistry panel, lipid panel, serum lipase and amylase
- Collect blood for sparse PK analysis – pre-dose trough sample to be collected 24 ± 2 hours post last dose of study drug.
- Allow the participant to take their study drug
- Review concomitant medication use since last visit. Record changes and additions as follows:
    - Prescription and OTC medications
    - Vitamins and herbal supplements
    - Alcohol, tobacco and marijuana
- Inquire and record information regarding adverse events (AEs)
- Obtain vital signs, and orthostatic vital signs (blood pressure, heart rate after participant has been seated for 1-3 minutes)
- Determine dose titration (**Visit 1 only** – if applicable)
- Maintain dose management log
- Complete study drug accountability and compliance check on all returned study drug bottles (empty, partially or unused); clarify issues with participant during visit; re-train participant if necessary.
- Administer MDS-UPDRS Parts I-III

Additional assessments

Visit 02 (Day 30 ± 3d)

- Perform 12-lead electrocardiogram (ECG)

Visits 01 and 02 will be approximately 2 hours in duration.

6.17 VISIT 03 (DAY 90 ±7D) – V03 – COHORT 2

Participant will return to clinic, abstaining from study drug for 24 ± 2 hours in anticipation of sparse PK blood draw.

Participant should bring study drug to the visit as CSF and corresponding serum PK will be collected 2 ± 0.5 hours post observed dose. Lumbar puncture must be conducted on the same day as the rest of the assessments for Visit 3. Coagulation parameters, collected within 10 days prior to the study visit, must be reviewed by the site investigator prior to performing the LP.

The Site Investigator should assess the participants need for ST at the beginning of the visit. If ST is required, V03 will be abandoned and replaced by the ST visit. The Site Investigator and Coordinator should refer to the ST visit description in section 6.24 and follow the ST visit SOA (NOTE: expected assessments for the visit being replaced will be provided in section 6.24 as well as the ST visit SOA).

Once ST is initiated, participants should be examined in the medications OFF and ON state. Participants should be instructed to NOT take PD medications prior to the study visit. Participants must bring both PD medication(s) and study drug to the visit. PD Medication should be taken **after** completion of MDS-UPDRS Part III OFF exam.

Once ST is initiated, MDS- UPDRS Part III assessment should be completed in the defined medications OFF state (approximately 12 hours after the last dose of ST) **and** in medications ON state (based on the participant/ Site Investigator defined BEST ON and/or approximately 1 hour after dose of ST). MDS-UPDRS Parts I and II are to be completed in the ON state. Part IV is historical information.

Initiation of ST is a reportable event and CTCC must be notified.

Assessments should be performed in the order presented below, if possible:

Participant must bring study drug to visit. Assessments should be performed in the order presented below, if possible:

- Administer MDS-UPDRS
    - If ST has been initiated:
        - Administer MDS-UPDRS Part III OFF (approximately 12 hours after the

last dose of ST)

- Allow the participant to take their PD medications
- Administer MDS-UPDRS Parts I-III in ON state (based on the participant/ Site Investigator defined BEST ON and/or approximately 1 hour after dose of ST)
- Administer MDS-UPDRS Part IV (historical information) - if applicable

- If no therapy has been initiated at this time:
  - Administer MDS-UPDRS Parts I-III
- Assess Need for ST
- If participant requires initiation of ST, an ST visit should be initiated (see SOA).
- **Assessments specifically required at this visit <u>must be completed</u> as well as assessments required for the ST visit (see SOA)**

- Collect blood for sparse PK analysis at 2 time points:
  - Sample 1: pre-dose trough sample **must be collected 24 + 2 hours post last dose** of study drug.
  - Sample 2: **must be collected 2 + 0.5 hours post observed dose** at the time of the collection of the CSF sample
  - LP must be conducted on the same day as the rest of the assessments for Visit 3.
- Review concomitant medication use since last visit. Record changes and additions as follows:
  - Prescription and OTC medications
  - Vitamins and herbal supplements
  - Alcohol, tobacco and marijuana
- Inquire and record information regarding adverse events (AEs)
- Perform physical examination (by Site Investigator)
- Perform fundoscopy exam (to be performed by the Site Investigator prior to the LP)
- Administer Hoehn and Yahr Scale
- Administer Modified Schwab & England Activities of Daily Living
- Administer Columbia Suicide Severity Rating Scale (C-SSRS) – 'Since Last Visit' version
- Obtain Vital signs, <u>including height, weight</u> and orthostatic vital signs (blood pressure, heart rate after participant has been seated for 1-3 minutes)
- Perform 12-lead electrocardiogram (ECG)
- Collect blood for safety lab tests: CBC, comprehensive chemistry panel, lipid panel, serum lipase and amylase

- Collect blood for biomarker panel
- Collect blood for coagulation parameters for a stat read if the participant did not have their blood drawn and coagulation parameters tested in the last10 days.
- Perform lumbar puncture to collect spinal fluid samples:
  - PK sample must be collected **2 ± 0.5 hours post observed dose** of study drug - note: a corresponding blood sample must also will also be collected at the same time.
  - CSF Biomarker sample
    - LP may be performed only after results of the coagulation lab parameters (collected at SV02 visit) and fundoscopic exam have been reviewed and documented
    - CSF samples will be process by specialty lab for biomarker panel
    - CSF sample will be processed by the site's local lab facility (unless the lab is not able to process the CSF within 4 hours) to evaluate protein and glucose levels (routine chemistry) and cell count;
- LP must be conducted on the same day as the rest of the assessments for Visit 3.
- Complete study drug accountability and compliance check on all returned study drug bottles (empty, partially used or unused)
- Ensure dose management log is maintained appropriately; clarify issues with participant during visit; re-train Participant if necessary.
- Dispense a 3-month resupply of study drug as outlined in Section 7.8.

  Under no circumstances may study drug be pulled from storage and given to a participant unless that drug kit has been assigned to that participant via the enrollment process.

  The coordinator will record the participant's initials and participant ID Number on the drug kit when it is taken from storage.

V03 will be approximately 4 hours in duration.

6.18 VISIT 04 (DAY 180 ±7D) – V04 – COHORT 2

Participants should be instructed to adjust the time they take their study medication so that PK blood collect may occur 2 to 5 hours post-dose.

The Site Investigator should assess the Participants need for ST at the beginning of the visit. If ST is required, V04 will be abandoned and replaced by the ST visit. The Site Investigator and Coordinator should refer to the ST visit description in section 6.24 and follow the ST visit SOA (NOTE: expected assessments for the visit being replaced will be provided in section 6.24 as well as the ST visit SOA).

If ST has already been initiated, Participant should be instructed to NOT take their PD medication(s) at home on the morning of V04 and bring their PD medication(s) to clinic. Participant must abstain from taking PD medication(s) for approximately 12 hours prior to their clinic visit. Participant must bring both PD medication(s) and study drug to visit.

<u>Once ST has been initiated, Participants should be examined in the medications OFF and ON state.</u> MDS- UPDRS Part III assessment should be completed in the defined medications OFF state (approximately 12 hours after the last dose of ST). PD Medication should be taken **<u>after</u>** completion of MDS-UPDRS Part III OFF exam.

In medications ON state (based on the participant/ Site Investigator defined BEST ON and/or approximately 1 hour after dose of ST). MDS-UPDRS Parts I and II are to be completed in the ON state. Part IV is historical information.

Assessments should be completed in the following order as closely as possible to ensure that the neurological tests are performed at approximately the same time (mornings) on each testing day:

- Administer MDS-UPDRS
  - If ST has been initiated:
    - Administer MDS-UPDRS Part III OFF (approximately 12 hours after the last dose of ST)
    - Allow the participant to take their PD medications
    - Administer MDS-UPDRS Parts I-IV in ON state (based on the participant/ Site Investigator defined BEST ON and/or approximately 1 hour after dose of ST)
    - Administer MDS-UPDRS Part IV (historical information) - if applicable

  - If no ST has been initiated at this time:
    - Administer MDS-UPDRS Parts I-III
    - Assess Need for ST
    - If participant requires initiation of ST, an ST visit should be initiated (see SOA).
    - **Assessments specifically required at this visit <u>must be completed</u> as well as assessments required for the ST visit (see SOA)**
- Review concomitant medication use since last visit. Record changes and additions as follows:
  - Prescription and OTC medications
  - Vitamins and herbal supplements
  - Alcohol, tobacco and marijuana

- Inquire and record information regarding adverse events (AEs)
- Perform physical examination (by Site Investigator)
- Perform Clinical Global Impression of Change (CGI) (Site Investigator and participant)
- Administer Hoehn and Yahr Scale
- Administer Modified Schwab & England Activities of Daily Living
- Administer Mattis Dementia Scale
- Administer Parkinson Disease Quality of Life Questionnaire (PDQ-39) (self-administered by participant)
- Administer European Quality of Life Scale (EQ-5D) (self-administered by participant)
- Administer Columbia Suicide Severity Rating Scale (C-SSRS) – 'Since Last Visit' version
- Administer Parkinson Disease Sleep Scale (PDSS)
- Obtain vital signs, including height, weight and orthostatic vital signs (blood pressure, heart rate after participant has been seated for 1-3 minutes)
- Perform 12-lead electrocardiogram (ECG)
- Collect blood for safety lab tests: CBC, comprehensive chemistry panel, including lipid panel, serum lipase and amylase
- Collect blood for biomarker panel
- Collect blood for sparse PK analysis:
    - **Sample must be collected 2 – 5 hours post dose of study drug.**
- Dispense a 3-month resupply of study drug as outlined in Section 7.8

  Under no circumstances may study drug be pulled from storage and given to a participant unless that drug kit has been assigned to that participant via the enrollment process.

  The coordinator will record the participant's initials and participant ID Number on the drug kit when it is taken from storage.
- Ensure dose management log is maintained appropriately; clarify issues with participant during visit; re-train Participant if necessary.
- Complete IP accountability and compliance check on all returned IP bottles (empty, partially used or unused)

V04 will be approximately 2 - 3 hours in duration.

6.19 VISIT 05 (DAY 270 ± 7D) – V05 – COHORT 2

Participant should return to clinic, abstaining from study drug for 24 ± 2 hours in anticipation of sparse PK blood draw; participant should bring study drug and PD medication (if applicable) to visit.

The Site Investigator should assess the participants need for ST at the beginning of the visit. If ST is required, V05 will be abandoned and replaced by the ST visit. The Site Investigator and Coordinator should refer to the ST visit description in section 6.24 and follow the ST visit SOA (NOTE: expected assessments for the visit being replaced will be provided in section 6.24 as well as the ST visit SOA).

If ST has already been initiated, Participant should be instructed to NOT take their PD medication(s) at home on the morning of V04 and bring their PD medication(s) to clinic. Participant must abstain from taking PD medication(s) for approximately 12 hours prior to their clinic visit. Participant must bring both PD medication(s) and study drug to visit.

Once ST has been initiated, Participants should be examined in the medications OFF and ON state. MDS- UPDRS Part III assessment should be completed in the defined medications OFF state (approximately 12 hours after the last dose of ST). PD Medication should be taken **after** completion of MDS-UPDRS Part III OFF exam.

In medications ON state (based on the participant/ Site Investigator defined BEST ON and/or approximately 1 hour after dose of ST). MDS-UPDRS Parts I and II are to be completed in the ON state. Part IV is historical information.

Assessments should be completed in the following order as closely as possible to ensure that the neurological tests are performed at approximately the same time (mornings) on each testing day:

- Administer MDS-UPDRS
  - If ST has been initiated:
    - Administer MDS-UPDRS Part III OFF (approximately 12 hours after the last dose of ST)
    - Allow the participant to take their PD medications
    - Administer MDS-UPDRS Parts I-IV in ON state (based on the participant/ Site Investigator defined BEST ON and/or approximately 1 hour after dose of ST)
    - Administer MDS-UPDRS Part IV (historical information) - if applicable

- If no therapy has been initiated at this time:
    - Administer MDS-UPDRS Parts I-III
    - Assess Need for ST
    - If participant requires initiation of ST, an ST visit should be initiated (see SOA).
    - **Assessments specifically required at this visit <u>must be completed</u> as well as assessments required for the ST visit (see SOA)**
- Collect blood for safety lab tests: CBC, comprehensive chemistry panel, lipid panel, serum lipase and amylase
- Collect blood for sparse PK analysis – pre-dose trough sample to be collected 24 + 2 hours post last dose of study drug.
- Allow the participant to take their study drug
- Review concomitant medication use since last visit. Record changes and additions as follows:
    - Prescription and OTC medications
    - Vitamins and herbal supplements
    - Alcohol, tobacco and marijuana
- Inquire and record information regarding adverse events (AEs)
- Perform physical exam (by Site Investigator)
- Administer Hoehn and Yahr Scale
- Assess need for ST
- Obtain vital signs, including height, weight and orthostatic vital signs (blood pressure, heart rate after participant has been seated for 1-3 minutes)
- Perform 12-lead electrocardiogram (ECG)
- Ensure dose management log is maintained appropriately; clarify issues with participant during visit; re-train Participant if necessary.
- Dispense a 3-month resupply of study drug as outlined in Section 7.8. **Under no circumstances may study drug be pulled from storage and given to a participant unless that drug kit has been assigned to that participant via the enrollment process. The coordinator will record the participant's initials and participant ID Number on the drug kit when it is taken from storage.**
- Complete study drug accountability and compliance check on all returned study drug bottles (empty, partially used or unused); clarify issues with participant during visit; re-train participant if necessary.

Visits 05 will be approximately 2 hours in duration.

6.20 VISIT 06 (DAY 365 ± 7D) – V06 – COHORT 2 (FINAL "ON DRUG" VISIT)

Participant should return to clinic, abstaining from study drug for 24 ± 2 hours in anticipation of sparse PK blood draw. Participant should bring study drug and PD medication (if applicable) to visit.

The Site Investigator should assess the participants need for ST at the beginning of the visit. If ST is required, V06 will be abandoned and replaced by the ST visit. The Site Investigator and Coordinator should refer to the ST visit description in section 6.24 and follow the ST visit SOA (NOTE: expected assessments for the visit being replaced will be provided in section 6.24 as well as the ST visit SOA).

If ST has already been initiated, Participant should be instructed to NOT take their PD medication(s) at home on the morning of V06 and bring their PD medication(s) to clinic. Participant must abstain from taking PD medication(s) for approximately 12 hours prior to their clinic visit. Participant must bring both PD medication(s) and study drug to visit.

Once ST has been initiated, Participants should be examined in the medications OFF and ON state. MDS- UPDRS Part III assessment should be completed in the defined medications OFF state (approximately 12 hours after the last dose of ST). PD Medication should be taken **after** completion of MDS-UPDRS Part III OFF exam.

In medications ON state (based on the participant/ Site Investigator defined BEST ON and/or approximately 1 hour after dose of ST). MDS-UPDRS Parts I and II are to be completed in the ON state. Part IV is historical information.

Assessments should be completed in the following order as closely as possible to ensure that the neurological tests are performed at approximately the same time (mornings) on each testing day:

- Administer MDS-UPDRS
  - If ST has been initiated:
    - Administer MDS-UPDRS Part III OFF (approximately 12 hours after the last dose of ST)
    - Allow the participant to take their PD medications
    - Administer MDS-UPDRS Parts I-IV in ON state (based on the participant/ Site Investigator defined BEST ON and/or approximately 1 hour after dose of ST)
    - Administer MDS-UPDRS Part IV (historical information) - if applicable
  - If no therapy has been initiated at this time:

    - Administer MDS-UPDRS Parts I-III
    - Assess Need for ST
    - If participant requires initiation of ST, an ST visit should be initiated (see SOA); if no need for ST, continue with visit schedule.
    - Assessments specifically required at this visit <u>must be completed</u> as well as assessments required for the ST visit (see SOA)
- Collect blood for PK;
    - Pre-dose trough sample to be collected 24 + 2 hours post last dose of study drug.
- Collect blood for safety lab tests: CBC, comprehensive chemistry panel including lipid panel, serum lipase and amylase
- Collect blood for coagulation parameters
- Collect blood for biomarker samples
- Allow the participant to take their study drug and PD medications (if applicable)
- Review concomitant medication use since last visit. Record changes and additions as follows:
    - Prescription and OTC medications
    - Vitamins and herbal supplements
    - Alcohol, tobacco and marijuana
- Inquire and record information regarding adverse events (AEs)
- Perform physical examination by Site Investigator
- Perform neurological examination by Site Investigator
- Administer Clinical Global Impression of Change (CGI) (Site Investigator and participant)
- Determine PD diagnostic features
- Complete primary diagnosis form
- Administer Mattis Dementia Scale
- Administer Parkinson Disease Quality of Life Questionnaire (PDQ-39) (self-administered by participant)
- Administer European Quality of Life Scale (EQ-5D) (self-administered by participant)
- Administer Parkinson Disease Sleep Scale (PDSS)
- Administer Blindedness questionnaires (Site Investigator, Coordinator and participant)
- Administer Treatment expectation questionnaire (self-administered by participant)
- Administer Modified Schwab & England Activities of Daily Living
- Administer Hoehn and Yahr Scale
- Obtain vital signs, including height, weight and orthostatic vital signs (blood pressure, heart rate after participant has been seated for 1-3 minutes
- Perform a 12-lead electrocardiogram (ECG)

- **Complete a final study drug accountability and compliance check on all returned study drug bottles (empty, partially used or unused)**
- Ensure dose management log is maintained appropriately; clarify issues with participant during visit; re-train Participant if necessary.

V06 will be approximately 2 hours in duration.

6.21 VISIT 07 (30 DAYS POST-TREATMENT ± 7 D) – V07 – COHORT 2
VISIT 08 (60 DAYS POST-TREATMENT ± 7D) – V08 – COHORT 2 END OF STUDY

The Site Investigator should assess the participants need for ST at the beginning of the visit. If ST is required, V07/V08 will be abandoned and replaced by the ST visit. The Site Investigator and Coordinator should refer to the ST visit description in section 6.24 and follow the ST visit SOA (NOTE: expected assessments for the visit being replaced will be provided in section 6.24 as well as the ST visit SOA).

Once ST has been initiated, Participants should be examined in the medications OFF and ON state. MDS- UPDRS Part III assessment should be completed in the defined medications OFF state (approximately 12 hours after the last dose of ST). PD Medication should be taken **after** completion of MDS-UPDRS Part III OFF exam.

In medications ON state (based on the participant/ Site Investigator defined BEST ON and/or approximately 1 hour after dose of ST). MDS-UPDRS Parts I and II are to be completed in the ON state. Part IV is historical information.

Assessments should be completed in the following order as closely as possible to ensure that the neurological tests are performed at approximately the same time (mornings) on each testing day:

- Administer MDS-UPDRS
  - If ST has been initiated:
    - Administer MDS-UPDRS Part III OFF (approximately 12 hours after the last dose of ST)
    - Allow the participant to take their PD medications
    - Administer MDS-UPDRS Parts I-IV in ON state (based on the participant/ Site Investigator defined BEST ON and/or approximately 1 hour after dose of ST)
    - Administer MDS-UPDRS Part IV (historical information) - if applicable
  - If no ST has been initiated at this time:
    - Administer MDS-UPDRS Parts I-III
    - Assess Need for ST
    - If participant requires initiation of ST, an ST visit should be initiated (see

SOA).

- **Assessments specifically required at this visit <u>must be completed</u> as well as assessments required for the ST visit (see SOA)**

- Review concomitant medication use since last visit. Record changes and additions as follows:
  - Prescription and OTC medications
  - Vitamins and herbal supplements
  - Alcohol, tobacco and marijuana
- Inquire and record information regarding adverse events (AEs)
- Administer Hoehn and Yahr Scale
- Administer Treatment expectation questionnaire (participant)
- Obtain vital signs and orthostatic vital signs (blood pressure, heart rate after participant has been seated for 1-3 minutes)
- Collect blood for safety lab tests: CBC, comprehensive chemistry panel, lipid panel, serum lipase and amylase

<u>Additional assessments</u>

Visit 07 (30 days post-treatment ± 7 d)

- Perform physical examination (by Site Investigator)
- Height and weight
- Perform fundoscopy exam (to be performed by the Site Investigator prior to the LP)
- Perform a 12-lead electrocardiogram (ECG)
- Administer Blindedness questionnaires (Site Investigator, Coordinator and participant)
- Perform lumbar puncture (can be performed on a separate day within the visit window if necessary). Lumbar puncture for this visit is strongly encouraged but not required.
  CSF Biomarker panel:
  - LP may be performed only after results of the coagulation lab parameters (collected at V06 visit) and fundoscopic exam (performed by Site Investigator) have been reviewed and documented
  - CSF sample will be processed by the site's local lab facility (unless the lab is not able to process the CSF within 4 hours) to evaluate protein and glucose levels (routine chemistry) and cell count;
  - CSF samples will be process by specialty lab for biomarker panel;
  - LP may be performed on a separate day within the visit window, if necessary
- Collect blood for biomarker panel
- **Complete IP accountability and compliance check on all returned IP bottles (empty, partially used or unused), if any were not returned in previous visits.**

V07 will be approximately 2 hours in duration.

Additional assessments

Visit 08 (60 days post-treatment ± 7d)

- Columbia Suicide Severity Rating Scale (C-SSRS) - 'Since Last Visit' Version – only
- Collect blood for pregnancy test (for all women unless they are surgically sterile)

V08 will be approximately 1.5 hours in duration.

6.22 UNSCHEDULED VISITS – VISIT U01, U02, ETC. – COHORT2

An unscheduled visit may be performed at any time during the study at the participant's request or as deemed necessary by the Site Investigator. The date and reason for the unscheduled visit or telephone contact will be recorded in the source documentation. Reasons for unscheduled visits may include, but are not limited to, a potential severe side effect, worsening of symptoms, multiple missed doses of medication.

Assessments should be completed in the following order as closely as possible to ensure that the neurological tests are performed at approximately the same time (mornings) on each testing day:

- Review concomitant medication use since last visit. Record changes and additions as follows:
    - Prescription and OTC medications
    - PD Medications – if dose increased, MDS-UPDRS ON required
    - Vitamins and herbal supplements
    - Alcohol, tobacco and marijuana
- Inquire and record information regarding adverse events (AEs)
- Administer Columbia Suicide Severity Rating Scale (C-SSRS) – 'Since Last Visit' version
- Obtain vital signs and orthostatic vital signs (blood pressure, heart rate after participant has been seated for 1-3 minutes)
- Perform 12-lead electrocardiogram (ECG)
- Collect blood for safety lab tests: CBC, comprehensive chemistry panel, lipid panel.
- Complete study drug accountability and compliance check on all returned study drug bottles (empty, partially or unused).

- Ensure dose management log is maintained appropriately; clarify issues with participant during visit; re-train Participant if necessary.

Unscheduled visit durations may vary

6.23 PREMATURE WITHDRAWAL VISIT - VISIT PW – COHORT 2

Participants have the right to withdraw from the study at any time without prejudice. The Site Investigator may withdraw study drug from a participant in the study in the event of intercurrent illness, adverse events, other reasons concerning the health or well-being of the participant, or in the case of lack of cooperation, non-compliance, protocol violation or other administrative reasons. Premature withdrawal will be implemented in the case of emergency disclosure of drug treatment.

In the event of premature withdrawal from the study, the Premature Withdrawal (PW) Visit procedures and evaluations should be completed whether or not the withdrawal is determined at a regularly scheduled study visit or at an unscheduled visit. In instances where the participant refuses or cannot to return for a PW visit, it is acceptable to attempt to obtain primary outcome data by telephone and arrange for return of study drug.

If a participant will return to the study site for the PW visit and undergo LP, coagulation parameters must be reviewed by the site investigator prior to performing the LP. The sample may be collected within 10 days of the PW visit.

Reasons for withdrawal of the participant prior to completion of the study must be stated in the eCRF and in the site source documentation for all study participants who were enrolled in the study. The participant will be encouraged to remain in the study and return for the 1 month and 2 month post drug evaluations (V07 and V08) as a safety follow up.

Adverse events will be followed for 30 days after a Participant's last dose of study drug. AEs, abnormal laboratory results and abnormal ECG values will be followed to resolution or stabilization, whichever occurs first, but no new AEs will be recorded.

In case the study drug has been discontinued for safety laboratory values or ECG abnormalities reasons, ECG and abnormal laboratories must be repeated in 2 weeks and at 30 days.

The CTCC must be informed within 24 hours of all subjects who Prematurely Withdrawal from study for any reason.

Participant will return to clinic, abstaining from both PD medications (if applicable) for 12 hours; the participant should bring both PD medications and study drug to visit.

Once ST is initiated, participants should be examined in the medications OFF and ON state. Participants should be instructed to NOT take PD medications at home on the morning of the study visit.

Once ST is initiated, MDS- UPDRS Part III assessment should be completed in the defined medications OFF state (approximately 12 hours after the last dose of ST). PD Medication should be taken **after** completion of MDS-UPDRS Part III OFF exam.

In medications ON state (based on the participant/ Site Investigator defined BEST ON and/or approximately 1 hour after dose of ST). MDS-UPDRS Parts I and II are to be completed in the ON state. Part IV is historical information.

Assessments should be completed in the following order as closely as possible to ensure that the neurological tests are performed at approximately the same time (mornings) on each testing day:

- Administer MDS-UPDRS
  - If ST has been initiated:
    - Administer MDS-UPDRS Part III OFF (approximately 12 hours after the last dose of ST)
    - Allow the participant to take their PD medications
    - Administer MDS-UPDRS Parts I-IV in ON state (based on the participant/ Site Investigator defined BEST ON and/or approximately 1 hour after dose of ST)
    - Administer MDS-UPDRS Part IV (historical information) - if applicable
  - If no ST has been initiated at this time:
    - Administer MDS-UPDRS Parts I-III
    - Assess Need for ST
    - If participant requires initiation of ST, an ST visit should be initiated (see SOA).

Assessments specifically required at this visit must be completed as well as assessments required for the ST visit (see SOA)

- Collect blood for PK only if the participants remained on the study drug or discontinued within last 3 days;
  - If study drug taken within past 3 days, collection of one **random sample** at any time relative to post-dose at each visit.
  - The time of the last dose of the study drug has to be documented in the eCRF
- Collect blood for pregnancy test (for all women unless surgically sterile)

- Collect blood for safety lab tests: CBC, comprehensive chemistry panel, lipid panel, serum lipase and amylase
- Collect blood for biomarker samples
- Review concomitant medication use since last visit. Record changes and additions as follows:
  - Prescription and OTC medications
  - Vitamins and herbal supplements
  - Alcohol, tobacco and marijuana
- Inquire and record information regarding adverse events (AEs)
- Perform physical examination by Site Investigator
- Perform neurological examination by Site Investigator
- Perform fundoscopy exam (to be performed by the PI prior to the LP)
- Administer Clinical Global Impression of Change (CGI) (Site Investigator and participant)
- Determine PD diagnostic features
- Complete primary diagnosis form
- Administer Mattis Dementia Scale
- Administer Parkinson Disease Quality of Life Questionnaire (PDQ-39) (self-administered by participant)
- Administer European Quality of Life Scale (EQ-5D) (self-administered by participant)
- Administer Columbia Suicide Severity Rating Scale (C-SSRS) - 'Since Last Visit' Version – only
- Administer Parkinson Disease Sleep Scale (PDSS)
- Administer Blindedness questionnaires (Site Investigator, Coordinator and participant)
- Administer Treatment expectation questionnaire (self-administered by participant)
- Administer Modified Schwab & England Activities of Daily Living
- Administer Hoehn and Yahr Scale
- Obtain vital signs, including height, weight and orthostatic vital signs (blood pressure, heart rate after participant has been seated for 1-3 minutes
- Perform a 12-lead electrocardiogram (ECG)
- Perform lumbar puncture. (Lumbar puncture for this visit is strongly encouraged but not required)
  - LP may be performed only after results of the coagulation lab parameters and fundoscopic exam have been reviewed and documented
  - CSF sample will be processed by the site's local lab facility (unless the lab is not able to process the CSF within 4 hours) to evaluate protein and glucose levels (routine chemistry) and cell count;

  - CSF samples will be process by specialty lab for biomarker panel;
  - LP may be performed on a separate day within the visit window, if necessary
- **Complete a final study drug accountability and compliance check on all returned study drug bottles (empty, partially used or unused)**
- Ensure dose management log is maintained appropriately; clarify issues with participant during visit; re-train Participant if necessary.

PW will be approximately 3 hours in duration.

6.24 SYMPTOMATIC THERAPY VISIT

If the Site Investigator determines that a participant needs PD ST, every attempt should be made to conduct an ST visit prior to initiation of ST. An ST visit should only be conducted if the subject has **NOT** yet started ST. Initiation of **ANY** PD medications (amantadine, anticholinergics, dopamine agonists or levodopa compounds) **OR** increase in the dose of medications allowed at baseline (amantadine and/or anticholinergics) is considered initiation of ST.

If the need for ST is determined by the Site Investigator **during** a regularly scheduled study visit or safety visit (per SOA), the ST visit should be initiated. Additionally, assessments required at specified visits (e.g., PK assessment, lumbar puncture, coagulation parameters) must be completed as well as assessments required for the ST visit.

If the need for ST is determined **between** scheduled study visits and the subject has NOT yet started taking PD medication(s), the ST visit should be initiated in place of the regularly scheduled visit (see SOA). **Note:** subjects will not be required to return for an additional visit to perform ST visit; ST will replace the next scheduled study visit, including safety visits.

Participants who initiate ST will continue on study drug and will continue in the study until the Final Visit.

Initiation of ST is a reportable event and CTCC must be notified.

Assessments should be performed in the order presented below, if possible:

- Administer MDS-UPDRS
  - Administer MDS-UPDRS Parts I-III
  - Assess Need for ST
  - If participant requires initiation of ST, an ST visit should be initiated (see SOA).

Assessments specifically required at this scheduled visit must be completed as well as assessments required for the ST visit (see SOA)

- Collect blood for PK – per SOA of visit replacing
- Collect blood for safety lab tests: CBC, comprehensive chemistry panel including lipid panel, serum lipase and amylase
- Collect blood for coagulation parameters ***– if replacing V06***
- Collect blood for pregnancy test (for all women unless surgically sterile)***- if replacing V06 or V08***
- Collect blood for biomarker samples – ***if replacing V06 or V07***
- Perform lumbar puncture ***if replacing V03 or V07*** (can be performed on a separate day within the visit window if necessary). Lumbar puncture for this visit is strongly encouraged but not required.
- Perform fundoscopy exam (to be performed by the Site Investigator prior to the LP)
- Lumbar Puncture for CSF Biomarker panel: ***if replacing V03 or V07***
    - LP may be performed only after results of the coagulation lab parameters and fundoscopic exam (performed by Site Investigator) have been reviewed and documented
    - CSF sample will be processed by the site's local lab facility (unless the lab is not able to process the CSF within 4 hours) to evaluate protein and glucose levels (routine chemistry) and cell count;
    - CSF samples will be process by specialty lab for biomarker panel;
    - LP may be performed on a separate day within the visit window, if necessary
- Review concomitant medication use since last visit. Record changes and additions as follows:
    - Prescription and OTC medications
    - Vitamins and herbal supplements
    - Alcohol, tobacco and marijuana
- Inquire and record information regarding adverse events (AEs)
- Administer Columbia Suicide Severity Rating Scale (C-SSRS) – 'Since Last Visit' version
- Perform physical examination by Site Investigator
- Perform neurological examination by Site Investigator
- Administer Clinical Global Impression of Change (CGI) (Site Investigator and participant)
- Assess need for ST
- Determine PD diagnostic features
- Complete primary diagnosis form
- Administer Mattis Dementia Scale
- Administer Parkinson Disease Quality of Life Questionnaire (PDQ-39) (self-administered by participant)

- Administer European Quality of Life Scale (EQ-5D) (self-administered by participant)
- Administer Parkinson Disease Sleep Scale (PDSS)
- Administer Blindedness questionnaires (Site Investigator, Coordinator and participant)- ***if replacing V06 or V07***
- Administer Treatment expectation questionnaire (self-administered by participant - ***if replacing V05, V07 or V08***
- Administer Modified Schwab & England Activities of Daily Living
- Administer Hoehn and Yahr Scale
- Obtain vital signs, including height, weight and orthostatic vital signs (blood pressure, heart rate after participant has been seated for 1-3 minutes
- Perform a 12-lead electrocardiogram (ECG)
- Complete a final study drug accountability and compliance check on all returned study drug bottles (empty, partially used or unused)
- Dispense a 3-month resupply of study drug as outlined in Section 7.8. – if replacing ***V03, V04 or V05***

Under no circumstances may study drug be pulled from storage and given to a participant unless that drug kit has been assigned to that participant via the enrollment process.

The coordinator will record the participant's initials and participant ID Number on the drug kit when it is taken from storage.

6.25 PROCEDURES FOR PARTICIPANTS WHO DISCONTINUE STUDY DRUG FOR SAFETY AND INTOLERABILITY REASONS

If a participant discontinues study drug for safety reasons, the participant should complete the Premature Withdrawal visit (section 6.23) either while the participant is still taking the study drug or as soon as possible after the drug discontinuation. Adverse events will be followed for 30 days after a Participant's last dose of study drug. AEs, abnormal laboratory results and abnormal ECG values will be followed to resolution or stabilization, whichever occurs first, but no new AEs will be recorded. The Participants will be encouraged to return for the 30 and 60 days post drug evaluations (V07 and V08) as per schedule of activities.

.

7. STUDY DRUG ADMINISTRATION/ASSIGNMENT

This section will describe how the study drug will appear and how it will be administered. Additional details about study drug, supply, labeling, and distribution are provided in the NILO-PD Operations Manual.

7.1 STUDY DRUG

The study will use nilotinib 150mg oral capsules and matching placebo.
Study drug will be supplied as identical appearing capsules (hard gelatin, size 1) for oral administration.

- **Active study drug** capsules will contain: **150mg nilotinib** and the following inactive ingredients: colloidal silicon dioxide, crospovidone, lactose monohydrate, magnesium stearate and poloxamer 188.
- **Placebo** will contain the inactive ingredients (colloidal silicon dioxide, crospovidone, lactose monohydrate, magnesium stearate and poloxamer 188) used in the active study drug.

The Active and Placebo study drug will be provided by Novartis Pharmaceuticals Corporation in 120-count, appropriately sized white high-density polyethylene (HDPE) bottles with a standard lid closure.

The CMSU will be responsible for the following:

- Generation of the clinical labels
- Labeling of the bottles and kits
- Configuration of the bottles into 4-bottle kits that will support 6 months of dosing
- Distribution of kits to the sites

7.2 LABELING

At a minimum, the following information will be included on each six (6) month kit box and each bottle:

- Name and address of distribution center
- Study number/Acronym
- Enrollment ID (randomization number)
- Description of the product (dosage form, strength, quantity per bottle)
- Route of administration

- Directions for use
- Storage conditions
- Space for information to be completed by Site Investigator/designee:
  - Name and telephone number of Site Investigator
  - Dispensing date
  - Participant number
- Statement "Caution: New Drug – Limited by United States law to investigational use"
- Statement: "Keep out of reach of children"
- Labeling will be in English text.

Participants enrolled into Cohort 1 will require one 6-month kit and Participants enrolled into Cohort 2 will require two 6-month kits.

7.3 PHARMACIST/SITE DRUG HANDLING INSTRUCTIONS

- Upon receipt of a study drug shipment from the CMSU, the pharmacist or study coordinator should perform a complete inventory by comparing packing list to check the contents and condition of the shipment.
- Discrepancies or damaged kits must be reported to CMSU for immediate replacement.
- The pharmacist/study coordinator performing the inventory the signs, dates and returns a copy of the Packing List/Acknowledgement of Receipt to CMSU **within 48 hours of receipt**.
- File the original Packing List/Acknowledgement of Receipt in the site's regulatory binder.
- Ensure drug is properly secured (locked area) and **stored at 25°C (77°F); excursions permitted between 15° to 30°C (59° to 86°F).**

Cohort 1

Study drug will be supplied in participant-specific kits that each contains four (4) bottles. Each four (4) bottle kit is intended to support six months of dosing and contains two (2) bottle A's and two (2) bottles B's.

Cohort 2

300mg Option

Study drug will be supplied in participant-specific kits that each contains four (4) bottles. Each four (4) bottle kit is intended to support six months of dosing.

<u>150mg Option</u>

Study drug will be supplied in participant-specific kits that each contains two (2) bottles. Each two (2) bottle kit is intended to support six months of dosing.

7.4 STORAGE

All study drug must be kept in a secure, safe area where temperature is controlled and monitored under recommended **storage conditions as stated on the labeling (at 25°C (77°F); excursions permitted between 15° to 30°C (59° to 86°F) with access limited to persons directly involved in the study.**

7.5 ACCOUNTABILITY OF STUDY DRUG SUPPLIES

In accordance with local regulatory requirements, the Site Investigator or designated site staff must document the amount of investigational product dispensed and/or administered to study participants, the amount received from the CMSU, and the amount destroyed upon completion of the study. Sites are expected to arrange for destruction any unused study drug at their site according to their institution's policies. Only if local environmental regulations prohibit the destruction of study drug at the site level will the CMSU accept a return of unused study drug from the site.

The Site Investigator is responsible for keeping current and accurate records of study drug received, dispensed and its disposition. The study drug must be stored under the appropriate conditions (described in Section 7.4) during the course of the study, the Site Investigator/designee must maintain an accurate inventory of all study drug dispensed to or returned by the Participant. The reason for any unreturned study drug must be documented.

The Site Investigator is responsible for ensuring product accountability records are maintained throughout the course of the study. The inventory will include details of the study drug and dispensed to participants, batch, and ID numbers. All unused capsules and bottles must be kept until reconciliation of delivery records with accountability logs by the study monitor.

7.6 PARTICIPANT NUMBER ASSIGNMENT/ENROLLMENT

The Biostatistics Coordination Center (BCC) will generate the randomization code that will identify the Enrollment ID/randomization numbers that the CMSU will include on the labels of the bottles/kits that will be supplied to sites.

- A5-digit Enrollment ID Number will be assigned at the BL (randomization) visit to confirm enrollment and proper receipt of the randomized study drug assignment. The Enrollment ID Number will be correspondingly labeled on the respective study drug container

- The treatment for each participant will be assigned by a randomized code. A blocked randomization scheme will be used to ensure approximately even distribution of participants in treatment groups.

- As the participant qualifies for the randomized phase of the study, the Site Investigator or Study Coordinator completes the RANDOM process in the Internet accessible Electronic EDC system, which will assign to that participant an Enrollment ID (Randomization Kit) Number. These numbers are assigned in a randomized order, rather than sequentially.

- The randomization algorithm and participant enrollment process will be implemented through the EDC system using authenticated, password-protected accounts for each study site. The EDC system will check for participant eligibility based on inclusion/exclusion page entries.

- Once a participant has been allocated an Enrollment ID Number, this number cannot be assigned to another participant.

7.7 CODING/EMERGENCY DRUG DISCLOSURE

The Site Investigator or Site Pharmacist (if applicable) will be provided with a sealed code envelope for each Enrollment ID (Randomized Kit Number) for all kits received from the CMSU. With each supply of Cohort #1 kits and Cohort #2 baseline kits will be tamper evident envelopes that are labeled with a randomization number and contain a scratch-off (similar to a lottery ticket) treatment disclosure associated with a particular randomization number. These envelopes contain the individual study treatment assignments associated with each kit. All sealed code envelopes will be collected at the closeout monitoring visit (either on site or remotely) at the end of the study and returned to CMSU will be inspected to ensure that they have not been opened.

Sealed code envelopes should only be opened in emergency situations for reasons of participant safety. Whenever possible, the Site Investigator should consult with the Clinical Monitor or another member of the study team prior to opening the sealed code envelope and "breaking the blind". When the sealed code envelope for a Participant has been opened, the reason must be fully documented detailing the purpose, date and personnel involved in the source document and entered on the case report form. The participant will be withdrawn from further exposure to study drug.

The disclosure envelope and contents should be sent to an unblinded designee at CMSU within 48 hours of the disclosure. Assigned drug treatment must not be revealed to any other individual associated with the study unless disclosure to him/her is critical to the care of the participant.

7.8 DOSAGE OF STUDY DRUG

Cohort 1

Participants will be randomized in a 1:1:1 ratio into one of the following treatment arms:

- nilotinib 150mg
- nilotinib 300mg
- placebo

Rules for drug initiations and titration

The double-blind dosing for the three treatment arms in Cohort 1 will be achieved using a two-bottle type (Bottle A and Bottle B) approach. During the two-week dose titration period, participants will take one capsule daily from Bottle A. After two weeks of titration, participants will continue to take one capsule daily from Bottle A and add one capsule daily from Bottle B. The table below summarizes the contents of Bottles A and B for the 150mg, 300mg and Placebo treatment arms.

Treatment Arm	Bottle A	Bottle B
Nilotinib 150mg	Nilotinib 150mg Capsules	Matching Placebo Capsules
Nilotinib 300mg	Nilotinib 150mg Capsules	Nilotinib 150mg Capsules
Placebo	Matching Placebo Capsules	Matching Placebo Capsules

At their baseline visit, participants will be dispensed one (1) bottle A and one (1) bottle B. Participants will initiate study drug at a dosage of 150mg daily or matching placebo (1 capsule once daily from bottle A) and titrate to either 150mg daily, 300mg daily or matching placebo (2 capsules once daily) after the first 2 weeks. To maintain the blind of the study, participants will take one (1) capsule from bottle A and one (1) capsule from bottle B once daily.

Participants must remain on one capsule daily dosage level a minimum of 14 days prior to upward titration to 2 capsules once daily. **Dose up-titration can be delayed at the discretion of the Site Investigator by 2 weeks but has to occur no later than V02 (1 month). If the participant is unable to up-titrate the dose, this will be considered intolerability and they will remain on one capsule daily for the duration of the study.**

At their 3-month visit, participants will return their bottles A and B from the baseline visit and will be dispensed an additional one (1) bottle A and one (1) bottle B. Participants will continue to take one (1) capsule from each bottle once daily for the remainder of the study.

Cohort 2

Participants will be randomized in a 2:1 ratio into one of the following treatment arms:

- Selected dose from Cohort 1 based on safety and tolerability (150mg or 300mg)
- Placebo

150mg Dose Option

The double-blind dosing for the two treatment arms in Cohort 2 will be achieved using a single bottle type approach. If 150mg is selected as the dose for Cohort 2, there will be no titration period. Participants will take one capsule daily for the duration of the study. Study drug will be supplied in participant-specific kits that each contains two (2) bottles. Each two (2) bottle kit is intended to support six months of dosing. Participants will be dispensed one (1) bottle at the baseline visit and will take one (1) capsule daily.

The table below summarizes the bottle contents for the 150mg and Placebo treatment arms.

Treatment Arm	Bottle
Nilotinib 150mg	Nilotinib 150mg Capsules
Placebo	Matching Placebo Capsules

Participants will be dispensed one bottle for three months of dosing at randomization and at Months 3, 6 and 9. At their 3, 6 and 9-month visits, participants will return their bottle from their previous visit and will be dispensed an additional one (1) bottle. Participants will continue taking one capsule daily for the remainder of the study. The 60 participants will each require 4 bottles of study drug.

300mg Dose Option

Double-blind dosing for the two treatment arms in Cohort 2 will be achieved using a single bottle type approach. During the two-week dose titration period, participants will take one capsule daily. After two weeks of titration, participants will begin taking two capsules daily.

Dose up-titration can be delayed at the discretion of the Site Investigator by up to 2 weeks and must occur no later than V02 (month 1). **If the participant is unable to up-titrate prior to V02, this will be considered intolerability and the participant will remain at one capsule daily for the duration of the study.**

Study drug will be supplied in participant-specific kits that each contain four (4) bottles. Each four (4) bottle kit is intended to support six months of dosing. Participants will be dispensed two (2) bottles at the baseline visit and will take one (1) capsule daily for the first two (2) weeks. After two (2) weeks participants will increase their dose to two (2) capsules daily.

The table below summarizes the bottle contents for the 300mg and Placebo treatment arms.

Treatment Arm	Bottle
Nilotinib 300mg	Nilotinib 150mg Capsules
Placebo	Matching Placebo Capsules

At their 3, 6 and 9-month visits, participants will return their bottles from their previous visit and will be dispensed an additional two (2) bottles. Participants will continue taking two (2) capsules daily for the remainder of the study.

7.8.1 RULES FOR STUDY DRUG ADMINISTRATION

- Study drug must be taken on an empty stomach.
  - Participants should avoid eating food for at least 2 hours before the dose is taken, and avoid eating food for at least 1 hour after the dose is taken.
- Participants should swallow study drug capsules whole with water.
- Participants should not drink grapefruit juice, eat grapefruit, or take supplements containing grapefruit extract at any time during treatment.
- Participants should take study medication at a regularly scheduled time daily in the morning (except when scheduled for specific study visits).
  - If a participant is late by 12 hours in taking their daily dose, it is considered a missed dose and should be instructed to take their next dose at their regularly scheduled time.
  - Participants should not take 2 doses at the same time to make up for a missed dose. Participants should never take a double dose in the event of a missed dose.
- Participants who take medication to block the production of stomach acid (H2 blocker, see Appendix 2), should avoid taking these medications for about 10 hours before study drug is taken or for at least 2 hours after study drug taken.
- Participants who take antacids containing aluminum hydroxide, magnesium hydroxide or simethicone to reduce the amount of acid in their stomach, should avoid taking these medications for about 2 hours before the study drug is taken or for at least 2 hours after study drug is taken.

7.9 RULES FOR DOSE REDUCTION

7.9.1 RULES FOR DOSE REDUCTION FOR INTOLERABILITY

Dosage reductions can occur at any time during the study.

All study drug reductions must be called into the CTCC.

In case of intolerability at two capsules daily dosage reduction to one capsule daily should be attempted prior to discontinuing study drug. Instructions for dosage reduction may be given either over the phone after review of any adverse events, or at the corresponding in-person visit (either regularly scheduled or unscheduled visit depending on the timing of the dosage reduction). See below for dosage reduction directions.

Cohort 1

Following the 2 weeks of titration, dose reduction will be accomplished by eliminating the capsule taken daily from **bottle A**. Participants will reduce their dosage in a blinded fashion by 1 capsule (equivalent to 150mg of nilotinib or placebo). Participants unable to tolerate one capsule from Bottle B daily will be taken off the study drug and will not be allowed to continue in the study.

Cohort 2

150mg Option –study drug will be suspended (see rules for dose re challenge below, Section 7.10).

300mg Option – Following two weeks of titration, dose reduction is accomplished by reducing quantity of capsules taken daily from two (2) to one (1).

If a participant is not able to tolerate the lowest dose of study drug, one capsule daily (150mg or placebo), the participant will be taken off the study drug and followed until resolution of any adverse events. The participant will be encouraged to complete the 30 and 60 day post drug evaluations as per schedule of activities. See Section 7.10 for the specific visits instructions.

7.9.2 RULES FOR DOSE REDUCTION/ SUSPENSION FOR SAFETY REASONS

Dosage reductions can occur at any time during the study. The following safety variables will trigger dose reduction/ suspension

Table 2. Dose adjustment for safety reasons

**Dose adjustment for QT prolongation**	
ECG with QTc > 480 msec	<u>Symptomatic (Cardiac Event, Syncope)</u><br>Terminate nilotinib dosing, treat accordingly, and obtain cardiology referral.<br><br><u>Asymptomatic</u><br>Withhold nilotinib<br>Perform an analysis of serum potassium and magnesium and if abnormal, correct with supplements to within normal limits.<br>Review concomitant medication usage.<br>Repeat ECG in one week.<br>Re-challenge allowed if QTcF returns to < 480 msec and to within 20 msec of baseline<br>Terminate dosing if QTcF returns to > 480 msec after re-challenge
**Dose adjustment for myelosuppression**	
Absolute Neutrophil count < 1.2 X 10^9/L<br><br>Platelet count < 100 X 10^9/L<br><br>Hemoglobin : 2 units drop in hemoglobin compared to baseline	<u>Symptomatic (Infection, Bleeding)</u><br>Terminate nilotinib dosing, treat accordingly, and obtain hematology referral.<br><u>Asymptomatic</u><br>Withhold nilotinib and monitor blood counts every 2 weeks until normalization<br>Re-challenge allowed if prior abnormalities fully resolve. Increase hematological monitoring to every 2 weeks.<br>Terminate dosing if the participant develops any recurrent evidence of myelosuppression
**Dose adjustment for selected non – hematologic laboratory abnormalities**	
Elevated serum lipase or amylase > 2 X the ULN<br><br>Elevated bilirubin ≥ 1.5 X ULN<br><br>Elevated ALT/AST> 2X the ULN	<u>Symptomatic (signs and symptoms of pancreatitis, hepatitis)</u><br>Terminate nilotinib dosing, initiate work-up and treat accordingly, and obtain gastroenterology referral.<br><br><u>Asymptomatic</u><br>Withhold nilotinib and repeat serum analyses.

	Re-challenge if serum lipase, amylase, bilirubin, or liver function tests (LFTs) normalize.
Magnesium ≤ 1.5<br><br>Potassium ≤ 3.5<br><br>Any out of range clinically significant electrolyte abnormalities	<u>Symptomatic (cardiac or other adverse events)</u><br>Terminate nilotinib dosing, treat accordingly, and obtain internal medicine referral.<br><br><u>Asymptomatic</u><br>Correct electrolytes abnormalities, and monitor electrolytes every 2 weeks until normalization (if clinically significant).<br><br>Terminate dosing if the participant develops recurrent evidence of hypokalemia, hypomagnesemia or clinically significant electrolyte abnormalities despite appropriate supplementation.
**Dosage adjustment for new cardiac and vascular occlusive events**	
New cardiovascular event	Terminate nilotinib dosing, initiate work-up and treat accordingly, and obtain cardiology referral.

See Section 6.11 (Cohort 1) and 9.25 (Cohort 2) for the instructions on the schedule of activities for the participants who discontinue the study drug for safety or tolerability reasons.

7.10 RULES FOR STUDY DRUG RE-CHALLENGE

All attempts at re-titration of study drug must be called into the CTCC.

7.10.1 RULES FOR DOSE RE-CHALLENGE IN CASE OF INTOLERABILITY

Following dosage reductions for <u>tolerability</u> reasons, participants may be re- challenged <u>once</u> during the study <u>titration</u> phase at the discretion of the Site Investigator.

Participants may resume titration to two capsules daily dosage at the discretion of the Site Investigator, provided the intolerability issues have resolved. Participants that are unable to tolerate the dose increase will be reduced to one capsule daily and enter the maintenance phase of the study. Participants unable to tolerate one capsule daily will be taken off the study drug and

will follow procedures outlined for study termination for intolerability (See Section 6.10-Premature Withdrawal).

No re-challenges will be allowed after Safety Visit 02 (SV02), Day 60. Visit 03, Day 90 will be the formal beginning of the maintenance phase of the study. If intolerability symptoms do not resolve by SV02, and participant is not able to tolerate at least one capsule daily, the participant will not be allowed to continue in the study and a premature withdrawal visit should be conducted. The participant will be required to return to the study site for the 30 and 60 day safety follow-up visits (for Cohort 1 = Visit 05 and Visit 06; for Cohort 2 = Visit 07 and Visit 08).

7.10.2. RULES FOR DOSE SUSPENSION AND RE- CHALLENGE FOR SAFETY REASONS

Dosage suspension for safety reasons can occur at any time during the study. Re-challenge will be allowed when abnormalities have resolved fully. Please, refer to the Table 2 (above)

See instructions for dose re challenges if dose reduction / suspension were triggered by safety reasons (Table 2 above).

7.10.3 RULES FOR DOSE RE-CHALLENGE IN CASES NOT RELATED TO SAFETY OR TOLERABILITY

If study drug is temporarily suspended for occurrences other than study drug related safety or tolerability issues, study drug may be resumed by the Site Investigator. Drug suspensions lasting more than 7 days require re-titration. If drug suspension is less than 7 days, the participant can restart study drug at prior dosing level. This occurrence must be carefully documented on the Dose Management Log.

For drug suspensions lasting more than 7 days, participants need to be re-titrated:

Cohort 1: Participants will take one capsule once daily (150mg or matching placebo- Bottle A) and increase to 2 capsules once daily (one capsule from Bottle A and one capsule from Bottle B) in 7 days.

Cohort 2: If dose will be 150mg: Participants will resume one capsule once daily dose (either 150mg or matching placebo).
If dose will be 300mg: Participants will take one capsule once daily (150mg or matching placebo) and increase the dose to 2 capsules once daily in 7 days.

Participants will not be allowed to remain in the study if study drug is permanently discontinued. Once the decision to permanently discontinue study drug is made, a premature withdrawal visit should be conducted. The participant will be encouraged return to the study site for the 30 and

60 day safety follow-up visits (for Cohort 1 = Visit 05 and Visit 06; for Cohort 2 = Visit 07 and Visit 08).

All study drug suspensions must be called into the CTCC.

8. CONCOMITANT MEDICATIONS

8.1 ALLOWED CONCOMITANT MEDICATIONS

Cohort 1- Stable regimen of PD medications, that includes levodopa, will be allowed. The regimen needs to be stable for at least 30 days prior to the screening visit and for the duration of the study if feasible. Dose adjustment will be allowed at the discretion of the Site Investigator. If PD medications are to be increased, participants should be seen at regularly scheduled visit and MDS-UPDRS ON has to be completed even if not part of the SOA for that visit. Reduction of the dose of PD medications for side effects or intolerability can be done at any point and can be done either in person or over the phone. The Site Investigator will document any new medications or changes in medication at each study visit in source documents and on the Concomitant Medication Log in the eCRF.

Cohort 2-Use of amantadine or anticholinergics will be allowed. The dosage needs to be stable for 30 days prior to baseline visit and for the duration of the study if feasible. Initiation or an increase in dosage of these medications or the initiation of any new ST during the study will result in the participant reaching milestone of time for ST and all assessments as described in the ST visit will need to be completed.

Any change in the dosage of these medications will require CTCC notification.

It is anticipated that around 50% of participants recruited in Cohort 2, will require initiation of ST during the study. As per inclusion criteria, Participants are not expected to start ST prior to Visit 3 (3 months). Subsequent to Visit 3, ST may be initiated at any time at the discretion of the Site Investigator and /or treating physician. The Site Investigator will document any new medications or changes in medication at each study visit in the source documents and on the Concomitant Medication Log in the eCRF.

Time to initiation of ST (for Cohort 2) or change in the dose of ST for Cohort 1 will be analyzed. ST dose will be calculated in levodopa dose equivalence (LDE)[40].

A stable regimen of central nervous system acting medications (benzodiazepines, antidepressants, hypnotics) is allowed. The dosage needs to be stable for 30 days prior to the baseline visit. The dosage can be adjusted during the study at the discretion of the Site Investigator. Central nervous system acting medications can be initiated during the study, if needed, at the discretion of the Site Investigator.

8.2 CAUTIONARY AND EXCLUSIONARY MEDICATIONS WITH NILOTINIB

The general categories of exclusionary medications include: QT prolonging drugs, strong CYP3A4/5 inhibitors or inducers, anticoagulants and proton pump inhibitors. Detailed lists of exclusionary and precautionary medications are available in Appendix 2 and may be updated as new information is generated. Study site staff will be informed if any updates to the list(s) are made.

Drugs that May Prolong the QT Interval

Nilotinib may prolong the QT interval. The administration of nilotinib with agents that prolong the QT interval such as anti-arrhythmic medicines should be avoided. See Appendix 2 for a list of drugs that can prolong the QT interval (see also the precautionary list of drugs with possible or conditional risk of Torsades de Pointes- Appendix 2).

Concomitant Strong CYP3A4 Inhibitors and Inducers

See Appendix 2 for a list of exclusionary medications: Strong CYP3A4 Inhibitors

Nilotinib is predominantly metabolized by CYP3A4 and partially by CYP2C8. Therefore, concomitant use of strong CYP3A4 inhibitors is prohibited to avoid undesirable increases in exposure to nilotinib. For example, in a drug-drug interaction study of healthy individuals receiving the CYP3A4 inhibitor, ketoconazole (400mg once daily), the systemic exposure of nilotinib was increased ~ 3-fold. Products containing grapefruit should also be avoided, as previous studies have reported increases in nilotinib Cmax and AUC by 60% and 29%, respectively (Yin et al. 2010[45]).

Concomitant use of strong inducers of CYP3A4 will also not be allowed, to avoid reducing nilotinib exposures to a clinically relevant extent. In a drug-drug interaction study of rifampicin, a potent CYP3A4 inducer, nilotinib AUC decreased by approximately 80%. Should treatment with any potent CYP3A4 inhibitors or inducers be required, therapy with nilotinib should be interrupted.

Nilotinib is a competitive inhibitor of CYP3A4, 2C8, 2C9, 2D6, and UGT1A1 in vitro, potentially increasing the concentrations of drugs eliminated by these enzymes. In vitro studies also suggest that nilotinib may induce CYP2B6, 2C8, and 2C9, and decrease the concentration of drugs which are eliminated by these enzymes. In human studies, nilotinib is considered a moderate inhibitor of CYP3A4. It has been shown to increase the systemic exposure of oral midazolam (a CYP3A4 substrate) 2.6-fold. As a result, the systemic exposure of drugs metabolized by CYP3A4 (e.g., certain HMG-CoA reductase inhibitors) may be increased when co-administered with nilotinib. Dose adjustment may be necessary for drugs that are CYP3A4 substrates, especially those that have narrow therapeutic indices when co-administered with nilotinib. Therefore, appropriate monitoring is needed for potential adverse effects related to

concomitant medications that are substrates of CYP3A4 and the other CYP enzymes mentioned above with the initiation of nilotinib. Also, precaution with the initiation of a new medication during the course of study should be taken if that agent is a substrate of any of the CYP enzymes mentioned above.

Nilotinib inhibits the human drug efflux transporter, P-glycoprotein (P-gp). If nilotinib is administered with drugs that are substrates of P-gp, increased concentrations of the substrate drug are likely, and cautions should be exercised. Nilotinib is also a substrate of P-gp, and if it is administered with drugs that inhibit P-gp, increased concentrations of nilotinib are likely. Thus, caution should be exercised with concomitant use of P-gp inhibitors.

See Appendix 2 for a list of exclusionary medications: Strong CYP3A4 Inducers.

Drugs that Affect Gastric pH

See Appendix 2 for a list of exclusionary medications: Proton Pump Inhibitors.

Nilotinib has pH-dependent solubility, with decreased solubility at higher pH. Drugs such as proton pump inhibitors that inhibit gastric acid secretion to elevate the gastric pH may decrease the solubility of nilotinib and reduce its bioavailability. In healthy participants, coadministration of a single 400mg dose of nilotinib with multiple doses of esomeprazole (a proton pump inhibitor) at 40mg daily decreased the nilotinib AUC by 34%. Increasing the dose of nilotinib when coadministered with such agents is not likely to compensate for the loss of exposure. Since proton pump inhibitors affect pH of the upper GI tract for an extended period, separation of doses may not eliminate the interaction. The concomitant use of proton pump inhibitors with nilotinib is not recommended.

Antacid that contains aluminum hydroxide, magnesium hydroxide, or simethicone are allowed. These medicines should be taken about 2 hours before or about 2 hours after they take study drug.

In healthy participants, no significant change in nilotinib PK was observed when a single 400mg dose of nilotinib was administered 10 hours after and 2 hours before famotidine (an H2 blocker). Therefore, when the concurrent use of an H2 blocker is necessary, it may be administered approximately 10 hours before and approximately 2 hours after the dose of nilotinib. Administration of an antacid (aluminum hydroxide/magnesium hydroxide/simethicone) to healthy participants, 2 hours before or 2 hours after a single 400mg dose of nilotinib did not alter nilotinib PK. Therefore, if necessary, an antacid may be administered approximately 2 hours before or approximately 2 hours after the dose of nilotinib.

9. ASSESSMENTS

The timing of assessments described below will be per the protocol Schedule of Activities.

9.1 PRIMARY VARIABLES

Tolerability: will be defined based on ability to complete the study on the assigned dose. Tolerability will be judged by the proportion of participants enrolled in a dosage group able to complete the study on their originally assigned dosage. Tolerability of each active arm will be compared to group.

Safety: Safety will be primarily assessed based on a direct comparison of treatment-related serious adverse events across the two treatment groups. Additional safety assessments will involve further comparisons of adverse events, overall serious adverse events, and safety laboratory findings across the two groups. Summary tables will be tabulated and provided for review to the Independent Medical Monitor (IMM) and Data Safety Monitoring Board (DSMB) on an agreed upon time frequency.).

Efficacy: will be assessed by the change of the MDS-UPDRS Part III over time. All participants in cohort 1 and participants in cohort 2 who have started ST will have an assessment of the motor exam (Part III) in a practically defined OFF state (approximately 12 hours after the last dose of ST) and ON state (based on the participant/ Site Investigator defined BEST ON, approximately 1 hour after dose of ST). A number of other exploratory efficacy outcome measures will be collected as per SOA

9.2 DOCUMENTATION OF PARKINSON DISEASE

The diagnosis of idiopathic PD should be documented based on the UK Brain Bank diagnostic criteria[41]. The participants are expected to have at least two out of three cardinal manifestations of PD. If tremor is not present, participants must have unilateral onset and persistent asymmetry of the symptoms.

9.3 SAFETY ASSESSMENTS

9.3.1 CLINICAL VARIABLES

In addition to the data from assessments listed below, participants will provide information on their demographics, past medical history, including PD, socio-economic history, smoking, alcohol and caffeine usage, as well as concomitant medication usage and compliance with study procedures.

9.3.2 MEDICAL HISTORY

Medical history (by body system with current-status) and detailed PD diagnosis and history will be obtained prior to randomization. Clinical evaluations at each subsequent in-person visit after randomization will review any changes over baseline and will involve clinical assessments including vital signs (blood pressure, heart rate, respiratory rate, temperature), weight, electrocardiogram, laboratory abnormalities, and adverse events.

9.3.3 PHYSICAL EXAMINATION

Physical examinations during the study will include assessment of skin, head, neck, lymphatic, eyes, ears, nose and throat, abdomen, and respiratory, cardiovascular/peripheral vascular and musculoskeletal systems.

9.3.4 NEUROLOGICAL EXAMINATION

A comprehensive neurological examination, including assessment of tremor and coordination, will be performed by the Site Investigator, who will conduct open and directed questioning to the participant followed by physical examination.

9.3.5 VITAL SIGNS, WEIGHT, HEIGHT

Vital signs will be assessed at every visit and will include blood pressure, pulse rate, respiratory rate and temperature. Blood pressure (BP) and pulse rate should be taken after participant has been seated for 1-3 minutes. Orthostatic vital signs are serial measurements of blood pressure and pulse that will be taken in supine position and standing position after 2 minutes. At Screening, if BP is "uncontrolled" (Systolic Blood pressure (SBP) greater than 180mmHg or Diastolic Blood Pressure (DBP) greater than 98mmHg) on the first reading, this can be repeated up to 2 more times to obtain a reading that is "controlled". If the first reading is "controlled" (SBP less than 180mmHg or DBP less than 98mmHg) then no need for further measurements. Measurement of height will be taken at Screening only and weight will be measured at all visits.

9.3.6 CLINICAL LABORATORY TESTS

Clinical laboratory tests will be performed by the laboratory(ies) specified in Section 4 of the Form FDA 1572 for the study and their reference ranges will be used. If a clinical laboratory not listed on the Form FDA 1572 is used, their reference ranges must be provided to the CTCC.

All samples for laboratory analysis must be collected, prepared, labeled, and shipped according to the laboratory's requirements as outlined in detail in the laboratory manual.

- Complete blood count (CBC) with differential (hematocrit, hemoglobin, platelet count, RBC indices, Total RBC, Total WBC, and WBC & differential)
- Comprehensive (metabolic) chemistry panel bicarbonate, blood urea nitrogen (BUN), chloride, creatinine, glucose, potassium, sodium glucose, calcium, albumin, total protein, electrolytes, alkaline phosphatase (ALP), alanine amino transferase (ALT), aspartate amino transferase (AST), bilirubin
- Specialty blood chemistry- Lipid panel (Lipids comprise cholesterol, High-Density Lipoprotein, Low-Density Lipoprotein and triglycerides), serum lipase and amylase.
- Serum pregnancy test for all women unless surgically sterile.
- All participants will have safety laboratory tests at the designated visits outlined in the protocol. These samples will be analyzed at a central laboratory. All samples for laboratory analysis must be collected, prepared, labeled, and shipped according to the laboratory's requirements. There will be provisions for reflex testing in the event of an abnormal result requiring immediate retesting.
- Coagulation Parameters- will be collected prior to performing Lumbar puncture. Results of the coagulation parameters should be available to the site Investigator prior to performing Lumbar puncture as per section 6.0

9.3.7 FUNDOSCOPY EXAM

Fundoscopy will be performed by the Site Investigator to examine the disk margins prior to every lumbar puncture. If disk margins are not clearly visualized the Site Investigator can refer the participant to ophthalmology for the dilated eye exam

9.3.8 LUMBAR PUNCTURE (LP)

The LP will be performed by the Site Investigator or another qualified clinician appointed by the Site Investigator.

A LP for the collection of approximately 15-20 mL of CSF will be conducted for all participants per the visit schedule unless there is evidence of clinically significant coagulopathy or thrombocytopenia that would interfere with the safe conduct of the procedure. It is expected that all participants will undergo an LP prior to randomization.

The baseline LP should be scheduled as indicated in the SOA within 7 to 14 days prior to randomization. Safety laboratory results have to be available prior to LP. The fundoscopic exam has to be documented. In case the clinician fails to perform routine LP, LP procedure can be scheduled under fluoroscopy – once that has been approved by the CTCC.

The first 1-2 mL of CSF will be discarded; the subsequent 2-4 mL will be processed at the site's local laboratory facility (unless the laboratory is not able to process the CSF within 4 hours) to conduct standard analyses on cell count, including red blood cell counts. The remaining CSF will be processed and shipped to a central laboratory for biomarker and PK assessments (PK only at visit 3). Please refer to the laboratory manual provided by the central laboratory.

The CSF samples will be sent to a central repository to be stored indefinitely for research purposes. The CSF samples will be made available to researchers to conduct analyses related to PD and other disorders.
Participants will be closely monitored during the procedure and following the procedure. Further details can be found in the Operations Manual for this study.

9.3.9 ELECTROCARDIOGRAM (ECG)

Electrocardiogram (ECG) (12-lead) will be performed at designated visits per the Schedule of Activities and transmitted electronically to the central ECG laboratory which will determine the RR, PR, QRS and QTc (QTcF) intervals and provide a report back to the site. The ECGs will be recorded after the participant has been in the supine position for at least 5 minutes and will be read centrally by qualified cardiologist(s). The ECG report must be reviewed by the Site Investigator and any new or worsening clinically significant abnormalities will be followed up with the participant and reported in the CRF as an AE.

10. CLINICAL ASSESSMENTS

10.1. MOVEMENT DISORDERS SOCIETY UNIFIED PD RATING SCALE (MDS-UPDRS)

The MDS-UPDRS will be conducted per the Schedule of Activities. The MDS-UPDRS was designed by movement disorders experts to address weaknesses of the original UPDRS (e.g., by adding questions on constipation and sialorrhea) while preserving its overall format. The MDS-UPDRS has four parts:

- **Part I (non-motor experiences of daily living)**, comprising
  - Part IA concerning behaviors that are assessed by the Site Investigator with all pertinent information from patients and caregivers
  - Part IB that is completed by the patient with or without the aid of the caregiver, but independently of the Site Investigator.

- **Part II (motor experiences of daily living)**, designed to be a self-administered questionnaire like Part IB, but similarly can be reviewed by the Site Investigator to ensure completeness and clarity.

- **Part III (motor examination)** has instructions for the rater to give or demonstrate to the patient; it is completed by the clinician rater. **Once the participant initiates ST** MDS-UPDRS part III will be collected in the defined medications OFF (approximately 12 hours after the last dose of ST) state at designated visits and cohorts and both OFF and ON (based on the participant/ Site Investigator defined BEST ON and/or approximately 1 hour after dose of ST) states for designated visit and cohorts per the schedule of activities.
- **Part IV (motor complications)** with instructions for the rater and also instructions to be read to the patient. This part integrates patient-derived information with the rater's clinical observations and judgments and is completed by the rater. **Complete Part IV only if ST has been initiated.**

Participants will self-administer Parts IB and II, but will review responses for accuracy and clarity with the Site Investigator or Coordinator. Parts IA, III and IV must be conducted by the Site Investigator. Parts I, II, and III will be conducted at study visits as indicated on the Schedule of Activities. **For participants who have started ST**, Part IV will be conducted at visits where MDS-UPDRS Parts I-III are conducted/collected. Use of MDS-UPDRS is responsive to core instrument recommendations for the Quality of Life subdomain of the National Institute of Neurological Disorders and Stroke (NINDS) Common Data Elements (CDEs) for PD, and to FDA guidance encouraging use of patient-reported outcomes (PROs) as a substantial portion of the responses are patient-reported. **Ideally, the same Site Investigator should assess all participants on parts IA and III of the MDS-UPDRS at all study visits.**

10.2 MODIFIED SCHWAB & ENGLAND ACTIVITIES OF DAILY LIVING SCALE

The Modified Schwab & England scale is a Site Investigator and participant assessment of the participant's level of independence. The participant will be scored on a percentage scale reflective of his/her ability to perform acts of daily living. Printed scores with associated descriptors range from 100% to 0% in increments of 10%, where 100% is "participant has full ability and is completely independent; essentially normal" and 0% is "vegetative functions such as swallowing, bladder and bowel functions are not functioning; bedridden". Scores should be coded in increments of 5, (i.e. 095, 090, 085). This assessment will be completed jointly by the participant and Site Investigator.

10.3 PARKINSON'S DISEASE QUESTIONNAIRE - 39 ITEM VERSION (PDQ-39) SCALE

The PDQ-39 is a disease-specific quality of life questionnaire containing 39 questions organized over eight domains: mobility (10 items), activities of daily living (6 items), emotional well-being (6 items), stigma (4 items), social support (3 items), cognition (4 items), communication (3 items), and bodily discomfort (3 items). The PDQ-39 is the most widely used health related-QoL instrument in PD, and is considered to have generally good psychometric properties and content validity. Use of PDQ-39 is responsive to core instrument recommendations for the Quality of Life subdomain of the NINDS CDEs for PD, and to FDA guidance encouraging use of patient reported outcomes (PROs). This assessment will be collected from participants.

10.4 HOEHN AND YAHR SCALE

The Hoehn and Yahr is a commonly used scale for describing how the symptoms of PD progress. The scale allocates stages from 0 to 5 to indicate the relative level of disability. This scale is included within the MDS-UPDRS and will be completed for all subjects.

- Stage zero: No symptoms.
- Stage one: Symptoms on one side of the body only.
- Stage two: Symptoms on both sides of the body. No impairment of balance.
- Stage three: Balance impairment. Mild to moderate disease. Physically independent.
- Stage four: Severe disability, but still able to walk or stand unassisted.
- Stage five: Wheelchair-bound or bedridden unless assisted.

10.5 MONTREAL COGNITIVE ASSESSMENT (MOCA)

In early PD, when cognitive deficits occur, they are subtle and mild and the participants usually perform in the normal range of the widely used Mini-Mental State Exam (MMSE). The Montreal Cognitive Assessment is a rapid screening instrument like the MMSE, but was developed to be more sensitive to patients presenting with mild cognitive complaints. It is designated an NINDS CDE for PD. Compared to the MMSE the MoCA may be more sensitive to mild cognitive deficits in PD.[188] The MoCA assesses short term and working memory, visual-spatial abilities, executive function, attention, concentration, language and orientation. The total score ranges from 0 to 30 (highest function).

10.6 PRIMARY DIAGNOSIS ASSESSMENT

The Primary Diagnosis form captures, in the Site Investigator's opinion, a current percentile probability the participant has idiopathic Parkinson disease as well as meets current diagnostic criteria for the study based on available information. Ranges include: 90-100%; 50-89%, 10-49% and 0-9%. In addition, the Site Investigator selects the most likely primary diagnosis

from a listing that includes idiopathic PD, many other neurological disorders, and the option of no neurological disorder nor other neurological disorder, or other neurological disorder.

10.7 PD FEATURES

The PD Features assessment captures participant reported first PD symptom onset and actual or estimated date of PD diagnosis and symptoms that were present at the time of diagnosis, if known.

10.8 BECK DEPRESSION INVENTORY-II (BDI-II)

The Beck Depression Inventory-II (BDI-II). The BDI-II is a 21-item self-reporting inventory covering cognitive, behavioral and somatic aspects of depression. Each item consists of four statements ordered for severity and the participant selects the statement that best describes how he or she felt over the past week. Total scores range from 0 to 63; higher scores indicate higher severity.

10.9 PARKINSON'S DISEASE SLEEP SCALE (PDSS)

The change in sleep function will be assessed via the Parkinson's disease sleep scale (PDSS). The PDSS consists of 15 questions that the person with Parkinson's rates by marking along a line that represents a continuum from worst (0) to best (10). The clinician or scorer then measures the marks and assigns values to them. The PDSS asks about movement during the night, awkward positions or difficulty in changing positions, nocturia, nightmares, and other factors that interrupt sleep. The PDSS is primarily useful for physicians in trying to assess the need for nighttime anti-parkinson's medications.

10.10 EUROPEAN QUALITY OF LIFE SCALE (EQ-5D)

The EQ-5D is a measure of self-reported health outcomes that is applicable to a wide range of health conditions and treatments. It consists of two parts: a descriptive system (Part I) and a visual analogue scale (Part II). Part I of the scale consists of 5 single-item dimensions including: mobility, self-care, usual activities, pain/discomfort, and anxiety/depression. Each dimension has a 3-point response scale designed to indicate the level of the problem. Part II uses a vertical graduated VAS (thermometer) to measure health status, ranging from worst imaginable health state to best imaginable health state. The extended version of the EQ-5D includes a valuation task which is used only for valuation studies. There is also an optional set of demographic questions (EuroQol Group; McDowell & Newell, 1996; Bowling, 2005).

10.11 BLINDEDNESS EVALUATION

Per the schedule of activities, the Site Investigator, Coordinator, and participant will complete a blindedness evaluation in which each is asked to give his/her independent impression of the

participant's treatment assignment and the primary and secondary reasons for this opinion. Participants' responses will not be available to the Site Investigator or Coordinator when they make their assessments.

10.12 C-SSRS (BASELINE AND SINCE LAST VISIT)

The Columbia Suicide Severity Rating Scale (C-SSRS) is an FDA endorsed questionnaire to screen for suicidality in trials of central nervous system (CNS) active compounds.[42,43] The C-SSRS is an interview by trained study personnel that should be done at Screening and during the study as outlined in the Schedule of Activities. The form called "Baseline" provided at Screening collects the history of suicide and a form called "Since the Last Visit" collects data at subsequent visits.

10.13 MATTIS DEMENTIA RATING SCALE (DRS-2)

The Mattis dementia rating scale is a psychometric Instrument designed to assess the extent and nature of dementia. The scale consists of content that covers: attention, initiation/perseveration, construction, conceptualization, and memory (Monsch et al., 1995). The scale has been used extensively in studies assessing cognitive function pertaining to neurodegenerative disorders like Alzheimer's disease (Monsch et al., 1995) and PD (Brown et al., 1999). The DRS -2 is responsive to change over time in early and more advanced PD and has been validated as a screening test to detect dementia in PD (Llebaria, Mov dis 2008). DRS-2 alternate forms will be used at V03 and PW (as applicable) during cohort 1 and at V03, ST and PW (as applicable) to avoid practice bias.

10.14 CLINICAL GLOBAL IMPRESSION SCALE

The CGI is a 3-item observer-rated scale that measures illness severity (CGIS), global improvement or change (CGIC), and therapeutic response. Each component of the CGI is rated separately; the instrument does not yield a global score.

The CGIS is rated on a 7-point scale, with the severity of illness scale using a range of responses from 1 (normal) through to 7 (amongst the most severely ill patients). CGIC scores range from1 (very much improved) through to 7 (very much worse). Treatment response ratings should take account of both therapeutic efficacy and treatment-related AEs and range from 0 (marked improvement and no side-effects), and 4 (unchanged or worse and side-effects outweigh the therapeutic effects).

10.15 PD – STUDY TREATMENT EXPECTANCY QUESTIONNAIRE

A self-administered questionnaire assessing expectations of study drug effect will be collected at the selected visits as specified in the SOA

10.16 ADDITIONAL ASSESSMENTS

10.16.1 ASSESSMENT OF PARTICIPANT COMPLIANCE

At each study visit, the Site Investigator and/or Study Coordinator will assess the participant's compliance with the study requirements. This will include checks of protocol compliance, including use of study drug and concomitant medications, if applicable in order to assess the reliability of participant-generated data. **(If applicable, note that participants who fail to comply with the study requirements may be withdrawn from the study.)**

10.16.2 ASSESS NEED FOR SYMPTOMATIC THERAPY (COHORT 2)

Beginning at Visit 3, the Site Investigator will assess the participant's need for ST. A questionnaire will be used to facilitate the Site Investigator's decision. This decision will be based on PD disability posing a threat to the participant's current occupational status, current abilities (potential capacities) related to occupational matters, to handle routine personal finances and domestic responsibilities, and activities of daily living.

10.16.3 PHARMACOKINETIC ASSESSMENTS

PK samples will be collected to determine the serum PK of nilotinib in patients with PD.

PK analysis will use **serum** and CSF sample from Visit 3.

- To investigate the ability of nilotinib to cross the blood brain barrier in PD patients at a dose(s) that is tolerable and safe as measured by dose-dependent increases in unbound nilotinib levels in the CSF
- To investigate the relationship between nilotinib exposures in serum and CSF with its ability to engage known molecular target(s) (measured by biomarkers of pathway inhibition in the serum and CSF (e.g., reduction in phosphor-cAbl)).
- To investigate factors that could affect the PK of nilotinib such as age, sex, PD severity, race/ethnicity.

Serum and CSF PK samples will be collected at the specified visits as outlined in the Schedule of activities and summarized below

- **<u>Cohort 1</u>**
  - Serum PK samples will be collected
  - <u>Visit 1 (Day 14):</u> Collection of a **pre-dose trough** sample (24 $\pm$ 2 hours since last dose).
  - <u>Visit 2 (Day 30):</u> Collection of a **pre-dose trough** sample (24 $\pm$ 2 hours since last dose).

  - Visit 3 (Day 90): Collection of a **pre-dose trough** sample (24 ± 2 hours since last dose). A cerebrospinal fluid (CSF) sample will be collected via lumbar puncture **2 ± 0.5 hours post-observed dose**, and a corresponding serum sample will also be collected.
  - Visit 4 (Day 180): Collection of a sample **2 – 5 hours post-dose**.
  - Safety Visits 2, 3, 4: Collection of one **random sample** at any time relative to post-dose at each visit.
  - Pre-Mature Withdrawal Visits: If study drug taken within past 3 days, collection of one **random sample** at any time relative to post-dose at each visit.

- **Cohort 2**
  - Visit 1 (Day 14): Collection of a **pre-dose trough** sample (24 ± 2 hours since last dose).
  - Visit 2 (Day 30): Collection of a **pre-dose trough** sample (24 ± 2 hours since last dose).
  - Visit 3 (Day 90): Collection of a **pre-dose trough** sample (24 ± 2 hours since last dose). A cerebrospinal fluid (CSF) sample will be collected via lumbar puncture **2 ± 0.5 hours post-observed dose**, and a corresponding serum sample will also be collected.
  - Visit 4 (Day 180): Collection of a sample **2 – 5 hours post-dose**.
  - Visit 5 (Month 9): Collection of a **pre-dose trough** sample (24 ± 2 hours since last dose).
  - Visit 6 (Month 12): Collection of a **pre-dose trough** sample (24 ± 2 hours since last dose).
  - Safety Visits 2, 3, 4, 5, 6: Collection of one **random sample** at any time relative to post-dose at each visit.
  - Pre-Mature Withdrawal Visits: If study drug taken within past 3 days, collection of one **random sample** at any time relative to post-dose at each visit.

PK samples will be collected and shipped according to the laboratory's requirements. (Refer to instructions in the laboratory's manual.)

10.16.4 BIOMARKERS ASSESSMENTS

Refer to the laboratory manual for the detailed description of the biomarkers panel.

Blood, serum, plasma and spinal fluid samples collection aimed to explore the ability of nilotinib to engage its known and exploratory molecular target(s) by biomarkers of pathway inhibition in the serum and CSF. In addition, Peripheral blood mononuclear cell (PBMC) will be collected. . In addition, the biomarkers panel will explore impact of nilotinib on dopaminergic system as measured by the levels of dopamine and its metabolites in CSF and collect data on other biomarkers linked to potential mechanism of neuroprotection (including but not limited to CSF alpha-synuclein, total tau, phospho-tau, etc.). Please, refer to the biomarkers analysis plan for the specific details on the data collection and to the labs operational manual for the instructions for the samples collection.

The samples will be collected, at the visits indicated:

- <u>Blood for DNA</u> [collected at BL]
- <u>Blood for biomarkers</u> [see SOA for the schedule of samples collection]

*If the participant permanently discontinues study drug for any reason, before the final visit, the blood sample should be taken at an unscheduled study visit on the day participant plans to discontinue the study drug as outlined in the SOA for the Premature Withdrawal visit (Section 6.10- Cohort 1; Section 6.23- Cohort 2).

<u>DNA Sample Collection</u>
A blood sample will be collected from each participant at the Baseline visit. These samples will be used to extract DNA for storage and shared for future unspecified research. Participants will be given the option of refusing to have this sample collected or may request that their sample be destroyed at any time, without affecting their participation in the study. One of the rationales for the DNA sample collection is to assess if there is correlation of response to nilotinib with a particular genetic profile.

11. INTERCURRENT ILLNESS

In the event of an intercurrent illness, it is at the Site Investigator's discretion to determine if the participant may continue in the study with study drug treatment. The clinical course of the intercurrent illness will be followed to its appropriate conclusion and full notation made in the appropriate source documentation. All intercurrent illnesses must be recorded in the eCRF as adverse events.

12. PREMATURE WITHDRAWALS/DROPOUTS

Participants will be advised in the written informed consent forms that they have the right to withdraw from the study at any time without prejudice, and may be withdrawn at the Site Investigator's/Principal Investigator's discretion at any time. Reason for withdrawal from study will be documented in the source documents and captured in the eCRF as appropriate.

If a participant who has started study drug terminates the study prematurely, every effort should be made to obtain final laboratory tests and evaluations of clinical status, especially those pertaining to the study's primary outcome measure. Reasonable effort should be made to contact any participant lost to follow up during the course of the study in order to complete study related assessments and retrieve any outstanding data, drugs or clinical supplies. Following unsuccessful telephone contact, an effort to contact the participant by mail using a method that provides proof of receipt should be attempted. A Participant may be deemed lost to follow up if all efforts have failed after one month. Such efforts should be documented in the source documents. Participants withdrawn from the study will not be replaced. Such efforts should be documented in the source documents.

A Participant may withdraw or be withdrawn from the study for the following reasons:

- Adverse Event
  - Refer to Section 14

- Administrative
  - Withdrawal of consent
  - Request of Study Sponsor or Steering Committee (SC)
  - Request of primary care physician
  - Participant non-compliance with protocol
  - Failure to meet entry criteria
  - Pregnancy
  - Protocol deviation
  - Participant deemed lost to follow up/failure to return
  - Early termination of study
  - Other

All patients who prematurely and permanently discontinue study medication prior to (Visit 04) in Cohort 1, or (Visit 06) in Cohort 2 will be asked to complete Premature Withdrawal visit and Post Drug Evaluation Visits V05/V06 for Cohort 1 and V07/V08 for Cohort 2, if possible.

Participant early discontinuation of study drug and premature withdrawal (including participants who are lost to follow up) should be reported to the CTCC Project Manager within 24 hours of the site's knowledge of the event.

The Screening/Demographic form must be completed for all study participants who sign informed consent. This includes participants who completed the study or withdrew/were withdrawn from study treatment or were screened and signed a consent form but did not start treatment.

The End of Study/Conclusion of Study Participation form should be completed for all participants who have been randomized. If a participant withdraws due to an adverse event, the site must ensure that the event is captured on the eCRF Adverse Event form.

List of criteria for permanent treatment discontinuation

The following may be justifiable reasons for the Site Investigator to discontinue a participant from treatment.

Participant specific criteria:

- Participant experiences two similar SAEs or one life-threatening SAE (assessed as related by the Independent Medical Monitor and/or site investigator)
- Participant becomes pregnant

Trial specific criteria:

- Any AEs, per Site Investigator judgment, that may jeopardize the participant's safety
- Participant develops any ECG, laboratory or clinical events as outlined in **Table 2, Section 7.9.2**
- Any unblinding of the study treatment by the Site Investigator
- Any use of prohibited concomitant treatment (see Appendix 2)
- At participant's request, i.e., withdrawal of the consent for treatment

Any abnormal laboratory or ECG value will be rechecked by the Clinical Monitor and the central reader, respectively, for confirmation before making a decision of permanent discontinuation of the study drug for the concerned participant.

If a participant decides to discontinue participation in the study, he/she should be contacted by the Site Investigator in order to obtain information about the reason(s) for discontinuation and collection of any potential AEs.

If possible, and after the permanent discontinuation of study drug treatment, the participant will be assessed using the Premature Withdrawal Visit.

All cases of permanent treatment discontinuation should be recorded by the Site Investigator in the source documents and appropriate pages of the eCRF when considered as confirmed.

Participants who permanently discontinue treatment will be withdrawn from the study. Please refer to section 6.10 (cohort 1) and section 6.23 (cohort 2) for specific instructions for premature withdrawal.

Circumstances for permanent discontinuation and premature withdrawal must be clearly documented in source documents.

13. ADVERSE EVENTS

Refer to the Safety Management Plan for a detailed description of adverse events assessment.

13.1 ADVERSE EVENT (AE) DEFINITION

Adverse event means any untoward medical occurrence associated with the use of a drug in humans, whether or not considered drug related.

An adverse event can be any unfavorable and unintended sign (e.g., an abnormal laboratory finding), symptom or disease temporally associated with the use of a drug, and does not imply any judgment about causality. An adverse event can arise with any use of the drug (e.g., off-label use, use in combination with another drug) and with any route of administration, formulation, or dose, including an overdose.

Some examples of adverse events are:

- A change, excluding minor fluctuations, in the nature, severity, frequency, or duration of a pre-existing condition.
- A deterioration in the participant's condition due to the participant's primary disease or a pre-existing condition.
- Development of an intercurrent illness during the study.
- Development of symptoms which may or may not be related to the use of a concomitant medication or study drug.
- Appearance of abnormal laboratory results or significant shifts from baseline, but still within the reference ranges, which the Investigator considers clinically important.

When possible, AEs should be reported as diagnoses rather than individual signs and symptoms or procedures. The AE description must include start and stop dates (when available), intensity, causality and outcome, as well as any actions taken.

13.2 SERIOUS ADVERSE EVENTS (SAE)

An SAE is an AE or suspected adverse reaction occurring at any dose of the investigational product (including placebo) and is considered "serious" if, in the view of either the investigator or IMM, it results in any of the following outcomes:

- Death
  - Report if you suspect that the death was an outcome of the adverse event, and include the date if known;
- Life-threatening adverse event
  - Report if suspected that the patient was at substantial risk of dying at the time of the adverse event, or use or continued use of the device or other medical product might have resulted in the death of the patient.
  - This does not include an adverse event that, had it occurred in a more severe form, might have caused death;
- Inpatient hospitalization (initial or prolonged)
  - Report if admission to the hospital or prolongation of hospitalization was a result of the adverse event.
  - Emergency room visits that do not result in admission to the hospital should be evaluated for one of the other serious outcomes (e.g., life-threatening; required intervention to prevent permanent impairment or damage; other serious medically important event).
  - This does not include hospitalization for:
    - Elective or pre-planned treatment for a pre-existing condition which has not worsened since signing the informed consent
    - Social reasons and/or respite care in the absence of any deterioration of the participant's general condition.
    - Complications that occur during hospitalization are AEs. If a complication prolongs hospitalization or fulfills any other serious criteria, the event is serious.
- Disability or Permanent Damage
  - Report if the adverse event resulted in a substantial disruption of a person's ability to conduct normal life functions, i.e., the adverse event resulted in a significant, persistent or permanent change, impairment, damage or disruption in the patient's body function/structure, physical activities and/or quality of life;
- Congenital Anomaly Birth Defect

    - Report if you suspect that exposure to a medical product prior to conception or during pregnancy may have resulted in an adverse outcome in the child.
- Other Serious (Important Medical Events)
    - Report when the event does not fit the other outcomes, but the event may jeopardize the participant and may require medical or surgical intervention (treatment) to prevent one of the other outcomes. Examples include allergic bronchospasm (a serious problem with breathing) requiring treatment in an emergency room, serious blood dyscrasias (blood disorders) or seizures/convulsions that do not result in hospitalization. The development of drug dependence or drug abuse would also be examples of important medical events.
    - This category also includes any event the Site Investigator or the Clinical Monitor judges to be serious or which would suggest a significant hazard, contraindication, side effect or precaution. It can also involve the withdrawal of a participant from a study due to abnormal lab values, excluding screening labs.

Reports of serious adverse events, as defined above, require immediate notification (within 24 hours of the site's awareness) to the CTCC Project Manager or Clinical Monitor whether or not the Investigator believes that the experience is related to study drug or expected.

Novartis Drug Safety & Epidemiology will also be notified if any of the events above occur. Reporting will occur according to the Safety Management Plan.

13.3 ADVERSE EVENT DEFINITIONS

1. Adverse events that present after a participant signs the informed consent but prior to the initiation of study drug are considered non-treatment emergent adverse events and will be coded separately.

2. Treatment Emergent AES (TEAE): Adverse events that present, or worsen in intensity or frequency, following the first dose of study drug will be categorized as treatment emergent adverse events.

13.4 IDENTIFYING AND RECORDING OF ADVERSE EVENTS

At each visit, the site study staff will assess adverse events by recording all voluntary complaints of the participant and by assessment of clinical and laboratory features. At each study visit, the occurrence of AEs should be sought by non-leading questioning of the participant and caregiver during the study and may also be identified when the participant and/or caregiver spontaneously volunteered them. Open-ended, non-leading questioning of the participant is the preferred method to detect AEs.

Suitable non-leading questions include:
"How are you feeling?"
"How have you been doing since your last evaluation?"
"Have you taken any new medicines since your last evaluation? If so, why?"

Collection of AEs will begin immediately following signing of the ICF through the final study visit. All AEs will be recorded into the adverse events eCRF in EDC within 5 business days. AEs should be recorded by diagnosis (if known) rather than individual signs and symptoms. Each individual event should be recorded in the eCRF. The Site Investigator will monitor each participant closely and record all observed or volunteered AEs. Adverse findings detected at the Screening visit (e.g., abnormalities on clinical laboratory testing, ECGs, physical/neuro examination) will be recorded as Medical History and adverse events occurring after the Screening visit but before starting study treatment will be recorded on the AE log and considered non-treatment emergent. Participants will be contacted by the site to follow up on any outstanding AE at the end of the study.

AEs will be followed:

- until resolution;
- stabilization;
- for 30 days after the participant's last dose of study drug;

Whichever occurs first.

13.5 ADVERSE EVENT CAUSALITY DEFINITIONS

For each adverse event, the causality (i.e., their relationship to the study treatment) must be assessed by the Investigator and recorded on the Adverse Event Log according to the classifications in the table below. Ambiguous cases should be considered as having a reasonable possibility of a causal relationship unless further evidence becomes available to refute this.

TERM	DEFINITION	CLARIFICATION
Unrelated	No possible relationship	The temporal relationship between drug exposure and the adverse event onset/course is unreasonable or incompatible, or a causal relationship to study drug is implausible.
Unlikely	Not reasonably related, although a causal relationship cannot be ruled out	While the temporal relationship between drug exposure and the adverse event onset/course does not preclude causality, there is a clear alternate cause that is more likely to have caused the adverse event than the study drug.
Possible	Causal relationship is uncertain	The temporal relationship between drug exposure and the adverse event onset/course is reasonable or unknown, dechallenge or

TERM	DEFINITION	CLARIFICATION
		rechallenge information is either unknown or equivocal, and while other potential causes may not exist, a causal relationship to the study drug does not appear probable.
Probable	High degree of certainty for causal relationship	The temporal relationship between drug exposure and the adverse event onset/course is reasonable. There is a clinically compatible response to dechallenge (rechallenge is not required), and other causes have been eliminated or are unlikely.
Definite	Causal relationship is certain	The temporal relationship between drug exposure and the adverse event onset/course is reasonable, there is a clinically compatible response to dechallenge, other causes have been eliminated, and the event must be definitive pharmacologically or phenomenologically, using a satisfactory rechallenge procedure if necessary.

AE Causality Table adapted from the National Cancer Institute (NCI) Common Terminology Criteria for Adverse Events (CTCAE), v. 4.03.

13.6 ADVERSE EVENT SEVERITY DEFINITIONS

The Site Investigator will make an assessment of severity (intensity) for each AE reported during the study. The assessment will be based on the Site Investigator's clinical judgement. The intensity of each AE recorded in the CRF should be assigned to one of the following categories:

- **Mild**: An event that causes no limitations of usual activities
- **Moderate**: An event that causes some limitation of usual activities
- **Severe**: An event that prevents carrying out usual activities

13.7 REPORTING SERIOUS ADVERSE EVENTS

- Collection of SAEs will begin immediately following signing of the ICF through the final study visit. The Site Investigator will monitor each participant closely and record all observed or volunteered SAEs. Serious adverse events occurring after signing the informed consent form but before starting study treatment will be considered non-treatment emergent. The Site Investigator should record all serious adverse events that occur during the study period on the Adverse Event Log and in the appropriate source documents.

- **The Site Investigator and/or Coordinator should notify the CTCC Project Manager by phone or email (PM) within 24 hours of his/her becoming aware of the occurrence of a serious adverse event. The PM will in turn notify the Clinical Monitor (CM) and Independent Medical Monitor (IMM). Novartis Drug Safety & Epidemiology will also be notified as outlined in Safety Management Plan.**

- The following information should be supplied if available at the time of the initial report: study identifier (acronym or short title), site number, participant number, date of onset of event, event description, criteria for a serious event that were met, hospital admission information (if applicable), and the Site Investigator's current opinion of the causality/relationship between the event and the study drug.

- Upon completion of the initial report, the CTCC PM will enter the appropriate participant information into the EDC Incident Protocol.

- Within 24 hours of the initial report, the Site Coordinator will complete the MedWatch 3500A form and submit to the CTCC, following procedures outlined in the operations manual. The MedWatch 3500Aform must be completed for all Serious Adverse Events regardless of causality or expectedness. Additional supplementary information (e.g., hospital discharge summary with patient identifiers obliterated) should be obtained and forwarded to the CTCC Project Manager.

- The Site Investigator determines causality (as defined in Section 14.5) of the adverse event for the purposes of SAE reporting. The Independent Medical Monitor (IMM) will make the final adjudication on causality.

- The IMM will review SAE reports to determine causality, severity and expectedness as outlined in the Safety Management Plan.

- IMM, with the assistance of the CTCC PM is responsible for identifying events requiring SAE reporting that are "Unexpected" and classified as "Suspected Adverse Reactions" (SUSARs) in an expedited manner.

- The Site Investigator will comply with his/her local IRB regulations regarding the reporting of AEs/SAEs.

13.8 SERIOUS ADVERSE EVENTS EXPECTEDNESS TO THE STUDY INTERVENTION

Expectedness will be determined for each SAE reported during the study. The assessment will be based on the IMM clinical judgement. The judgement will be based on the known spectrum of adverse events reported with the investigational drug nilotinib, as reported in the package

insert (Tasigna®, AMN107, Novartis, Switzerland) revised 12/2017. https://www.pharma.us.novartis.com/sites/www.pharma.us.novartis.com/files/tasigna.pdf

and limited data from the small study in PD population (see Section 1.1)

Each SAE should be assigned to one of the following categories:

- Expected
- Unexpected

13.9 REVIEW OF ADVERSE EVENTS

- Adverse events will be presented in tabular form and given to the IMM and the DSMB on a periodic basis as agreed upon or as requested. Local Site Investigators are also required to fulfill all reporting requirements of their local institutions.

  The DCC/BCC will prepare aggregate reports of all adverse events (serious/not serious, expected/unexpected and relationship to study drug) for the IMM and the DSMB on an agreed upon basis or as requested. In addition, all adverse events will be coded using the MedDRA system. A report detailing protocol compliance will also be available from the DCC/BCC for DSMB and/or site review monthly or as requested. The research team will then evaluate whether the protocol or informed consent document requires revision based on the reports.

13.10 FOLLOW-UP OF UNRESOLVED EVENTS

- SAEs that remain ongoing past the participant's last protocol-specified visit will be evaluated by the Site Investigator.
  SAEs will be followed:
  - until resolution;
  - stabilization;
  - for 30 days after the Participant's last dose of study drug;

Whichever occurs first.

The Site Investigator and CM will assist the IMM, as appropriate, by providing further information on the event especially if the event has not resolved or stabilized at the time of completion of the initial report. This may involve contacting other clinicians responsible for the participant's care, with participant's authorization for release of information, to obtain information on diagnoses, investigations performed and treatment given.

13.11 REPORTING OF PREGNANCY

- This study excludes women of child bearing potential. While unlikely, the study site must report all pregnancies in study participants or female partners of male participants to the CTCC PM within 24 hours from notification.
- Any participant becoming pregnant during the study will be taken off study drug immediately and will have premature withdrawal visit.
- All reported pregnancies must be followed to conclusion and outcome reported to the CTCC PM on the *Pregnancy Outcome Form.* If an SAE occurs during pregnancy (e.g., spontaneous abortion), if the offspring is born with a congenital anomaly or there is a neonatal death, the site has to inform the CTCC PM within 24 hours from notification.
- Pregnancies in Female Partners of Male Participants:
  - Male participants will be instructed through the Informed Consent Form to immediately inform the Site Investigator if their partner becomes pregnant during the study or within 12 weeks after the last dose of study drug. The Site Investigator must notify the CTCC within 24 hours after learning of the pregnancy. Attempts should be made to collect and report details of the course and outcome of any pregnancy in the partner of a male participant exposed to study treatment. A Site Investigator who is contacted by the male participant or his pregnant partner may provide information on the risks of the pregnancy and the possible effects on the fetus, to support an informed decision in cooperation with the treating physician and/or obstetrician.

13.12 REPORTABLE EVENTS

The following occurences will be considered reportable events and must be reported to the CTCC within 24 hours of the event, or the Site Investigator's knowledge of the event. The CTCC PM and Principal Investigator will notify FDA, Novartis and other appropriate entities as necessary as outlined in the Safety Management Plan:

- Temporary suspension of Study Drug;
- Study Drug reduction/rechallenge;
- Participant withdrawal;
- Early discontinuation of Study Drug;
- Serious adverse event (SAE);
- Death
- Emergency treatment disclosure;
- Overdosage, defined as is the accidental or intentional use of the drug in an amount higher than the dose being studied even if it did not result in AE;
- Pregnancy (including female partner of male participant);

- All reports of misuse and abuse of Nilotinib
- Other medication errors and uses outside of what is foreseen in the protocol (irrespective if a clinical event has occurred)

14. STATISTICAL CONSIDERATIONS

A brief description of the statistical analysis plan is provided below. More details will be included in a formal statistical analysis plan, which will be developed by Clinical Trials Statistical Management Center at the University of Iowa in collaboration with the protocol PI, CTCC and NILO-PD Steering Committee (SC)

Due to the exploratory nature of this study, we will not attempt to make any type of adjustment for multiple comparisons. Correspondingly, unless specified differently, all hypotheses will be assessed at that 0.05 significance level.

14.1 GENERAL DESIGN ISSUES

14.1.1 SUMMARY OF STUDY DESIGN – COHORT 1

Nilotinib is approved by the FDA for certain types of leukemia. A small number of cell and animal models suggest that nilotinib may positively affect the α-synuclein pathology observed in PD[1-3,7,31,36]. A small clinical study that lacked a placebo control group and blinding tested the safety/tolerability of nilotinib in PD patients for the first time, and also explored its efficacy[9] Although the preliminary data appear promising, the study design used in these prior studies precludes making firm conclusions about safety, tolerability, optimal dose, or efficacy[10]. Hence, an opportunity exists to rigorously establish the safety, tolerability, and optimal dose of nilotinib to enable and inform the conduct of future definitive efficacy studies in PD participants.

To address these issues, we will conduct a multi-center, prospective, randomized, controlled, double-blinded phase IIa study intended to evaluate the safety, tolerability, and preliminary efficacy of nilotinib in participants with moderate to advanced PD. The first stage of the study will enroll approximately 75 participants, randomized in a 1:1:1 manner to receive a once daily dose of either nilotinib (150mg), nilotinib (300mg), or placebo. All Participants will be treated for 6 months. The primary objective of the first stage of this study is to determine the most tolerated dose. This objective will be accomplished by comparing the percentage of participants who complete the study on their assigned dose across the three arms. Although the decision will be primarily based on tolerability, we will also compare the safety profiles across the three groups and utilize this safety information in making a final decision. At the end of the first stage, we will make one of three decisions:

- If the 300mg dose is tolerable, and has an acceptable safety profile, this dose will be chosen for inclusion in the second cohort.
- If the 300mg dose is not tolerable, or if the 300mg dose is tolerable but has an unacceptable safety profile, then we will consider the 150mg dose. If the 150mg dose is

tolerable, and has an acceptable safety profile, this dose will be chosen for inclusion in the second cohort.

- If neither dose is tolerable, or if all tolerable doses have unacceptable safety profiles, we will conclude that no dose appears suitably safe and tolerable for inclusion in the second cohort. Under this scenario, the trial should be stopped without proceeding to a second cohort.

A key secondary objective of the first cohort will be to conduct a single group futility hypothesis in each PD group comparing the observed change in MDS-UPDRS part III between baseline and month 6 in order to determine whether the study can rule out the large change previously reported in the prior clinical study[9] . Other secondary objectives will involve examining the degree of symptomatic effect of nilotinib as measured by the change in MDS-UPDRS part III shortly after initiation of study drug (between baseline and 1 month), and after discontinuation of study drug [between month 6 (end of treatment)and month 7 (1 month after treatment)]. For all participants, assessment of the potential symptomatic effect will be done using the MDS-UPDRS part III collected in the ON (based on the participant/site investigator defined best ON and/or approximately 1 hour after dose of ST) state.

14.1.2 SUMMARY OF STUDY DESIGN – COHORT 2

The second cohort will involve a multi-center, prospective, randomized, controlled, double-blind phase IIa study intended to evaluate the safety, tolerability, and preliminary efficacy of nilotinib in participants with early/de novo PD. Based on the algorithm described in section 14.1.1 above, the dose most tolerated and safe from cohort 1 will be selected for inclusion in cohort 2. The second stage of the study will enroll approximately 60 participants, randomized in a 2:1 manner to receive a once daily dose of either nilotinib (at the dose chosen from the findings in cohort 1) or placebo. All participants will be treated for 12 months. The primary objective of the second stage is to determine whether the chosen dose of nilotinib appears to be sufficiently safe and tolerable to support further study in larger studies to further assess efficacy. A secondary objective will involve examining the degree of symptomatic effect of nilotinib as measured by the change in MDS-UPDRS part III shortly after initiation of study drug (between baseline and 1 month), and after discontinuation of study drug (between month 12 (end of treatment) and month 13 (1 month after treatment). For the participants treated with ST, assessment of the potential symptomatic effect will be done in the PD medications ON (based on the participant/site investigator defined best ON and/or approximately 1 hour after dose of ST) state.

14.1.3 RANDOMIZATION

Refer to Section 7.6 for details.

The randomization process will be similar for both Cohort 1 and 2. Participants will be assigned a Participant ID number at the time they sign/date the informed consent form. The treatment for each participant will be assigned by a randomized code. A blocked randomization scheme will be used to ensure approximately even distribution of participants in treatment groups. As the participant qualifies for the randomized phase of the study, the Site Investigator or Study Coordinator completes the randomization process in the EDC system, which will assign to that participant a unique Enrollment ID (Randomization Kit) Number. These numbers are assigned in a randomized order, rather than sequentially. The randomization algorithm and participant enrollment process will be implemented through the EDC system using authenticated, password-protected accounts for each study site. The EDC system will check for participant eligibility based on inclusion/exclusion page entries

14.2 OUTCOMES

14.2.1 PRIMARY OUTCOME

The specific primary aims of both stages of this study are to determine whether nilotinib is sufficiently safe and tolerable to warrant future study in a phase III efficacy trial.

Tolerability: Tolerability will be defined based on the ability to complete the study (6 months for cohort 1; 12 months for cohort 2) on the assigned dose. Any Participant who has to be removed from study drug, or fails to complete the study (for any reason) will be deemed not to have tolerated their assigned medication. Tolerability will be assessed by comparing the percentage of Participants enrolled in each dosage group who are able to complete the study on their originally assigned dosage group across all groups within each cohort.

Safety: Safety will be primarily assessed by examining the frequency of treatment- related serious adverse events across all groups within each cohort.

14.2.2 SECONDARY OUTCOME

A key secondary efficacy outcome for each cohort is to compare the MDS-UPDRS part III over time.

All participants in Cohort 1 and participants in cohort 2 who have started PD medications will have an assessment of the motor exam (Part III) in a practically defined OFF state (approximately 12 hours after the last dose of ST) and ON state (at least one-hour post dose).

14.3 ANALYSIS PLAN – COHORT 1

14.3.1 PRIMARY HYPOTHESES

Primary Tolerability Hypotheses: ***To assess whether either 150mg or 300mg once daily dosing of nilotinib is sufficiently tolerable, compared to placebo***

Below, we introduce some key notation that we use to describe the analysis plan for the proposed trial:

- Let p_{150} represent the true (unknown) percentage of participants treated with nilotinib 150mg who will tolerate the assigned medication
- Let p_{300} represent the true (unknown) percentage of participants treated with nilotinib 300mg who will tolerate the assigned medication
- Let p_{p} represent the true (unknown) percentage of participants treated with placebo who will tolerate the assigned medication

The primary tolerability hypotheses being tested in this trial are that participants treated with either dose of nilotinib will have similar tolerability to participants treated with placebo. The primary hypotheses will compare the overall tolerability rates for each nilotinib arm vs. placebo participants. Using the notation above, the two one-sided hypothesis tests below will be assessed:

$$H_{01}: p_{150} = p_P \quad vs. \quad H_{A1}: p_{150} < p_P$$

AND

$$H_{02}: p_{300} = p_P \quad vs. \quad H_{A2}: p_{300} < p_P.$$

Therefore, rejecting the null hypothesis suggests that the particular dose group of nilotinib shows significantly less tolerability relevant to the placebo group. If we do not reject the null hypothesis, this would provide justification for proceeding with further study of that drug (pending the results of the safety assessment). Each hypothesis will be assessed via a logistic regression model, adjusted for center (stratification variable). Due to randomization, it is unlikely that important covariates will be imbalanced in this study. However, given the small sample size, this cannot be dismissed. We will assess for important baseline imbalances, and if any imbalances exist, the logistic regression model will also adjust for the relevant covariates with important imbalances present.

Primary Safety Hypothesis: ***To assess whether there are clinically important increases in treatment related adverse events for the nilotinib-treated vs. placebo participants.***

The set of primary safety hypotheses will involve separate comparisons of the safety profiles for each of the nilotinib groups vs. placebo. As described elsewhere, general assessments of safety will occur throughout the trial in conjunction with reviews by the IMM and DSMB. This specific

primary hypothesis will be assessed in two ways. First, the percentage of participants who experience a treatment-related SAE, overall and by body system, will be compared across the two groups using standard chi-square tests. Then, the rates of treatment-related SAE's across the two groups will be compared using a Poisson regression model. In order to provide an overall summary of the safety of each nilotinib dose, additional safety assessments will involve further comparisons of adverse events, serious adverse events, and safety laboratory findings across the two groups. All of this information will be synthesized and reviewed by the Sponsor and steering committee at the end of each cohort. Notably, this summary information will be critical for making the dosing decision for cohort 2 (or deciding not to proceed with cohort 2) at the conclusion of follow-up for cohort 1.

14.3.2 DECISION ALGORITHM BASED ON PRIMARY HYPOTHESES

At the conclusion of these analyses, a decision rule will be invoked to make a determination regarding the "best" dose to move forward with in the second cohort of the study. This decision will be based on a combination of the results from the tolerability and safety hypotheses. In general, the decision algorithm will proceed as follows:

- Did the 300mg nilotinib group show suitable tolerability?
    - If 'Yes', then did the 300mg nilotinib group show suitable safety?
        - If 'Yes', then **recommend taking the 300mg nilotinib dose to the second cohort**
        - If 'No' then did the 150mg nilotinib group show suitable tolerability?
            - If 'Yes', then did the 150mg nilotinib group show suitable safety?
                - If 'Yes', then **recommend taking the 150mg nilotinib dose to the second cohort**
                - If 'No' then **recommend stopping the study and concluding that neither dose is sufficiently safe and tolerable to justify further exploration**
            - If 'No' then **recommend stopping the study and concluding that neither dose is sufficiently safe and tolerable to justify further exploration**
    - If 'No', then did the 150mg nilotinib group show suitable tolerability?
        - If 'Yes', then did the 150mg nilotinib group show suitable safety?
            - If 'Yes' then **recommend taking the 150mg nilotinib dose to the second cohort**

- If 'No' then **recommend stopping the study and concluding that neither dose is sufficiently safe and tolerable to justify further exploration**

- If 'No' then **recommend stopping the study and concluding that neither dose is sufficiently safe and tolerable to justify further exploration**

14.3.3 KEY SECONDARY OBJECTIVE

A key secondary objective of this study is to conduct a single group hypothesis within each PD group to assess "futility" for replicating the large difference observed in a previously published study[9] . This will be based on a single group hypothesis test comparing the observed change in the MDS-UPDRS part III scores collected ON (based on the participant/site investigator defined best ON and/or approximately 1 hour after dose of ST) between baseline and 6 months to the observed change reported from the Pagan et al[9] study in order to determine whether we can rule out the large change previously reported. Based on the results from the Pagan et al[9] publication, which used the UPDRS:

- Mean change over 6 months in the 150mg group = 7.0 reduction (std dev = 12.9)
- Mean change over 6 months in the 300mg group = 10.8 reduction (std dev = 7.8)

For consistency, we will test a similar hypothesis within each group using the smallest observed reduction. However, since we will be using the MDS-UPDRS, we include a correction factor of 1.4 as specified in Goetz et al[44]. In other words, the 'futility' test will be based on the following set of hypotheses:

$$H_0: \delta \leq -9.8 \text{ (7 x 1.4) vs. } H_A: \delta > -9.8$$

where δ represents the change from baseline to 6 months in the corresponding group being tested. Since this is an early phase exploratory study, and we want to ensure adequate power for ruling out the large effects previously observed, we assume an alpha level of 0.10 for each one-sided hypothesis of interest.

To assess this hypothesis, the following linear mixed effects model (LMM) will be used. Model parameters and variance components will be estimated using restricted maximum likelihood. Several potential covariance structures (unrestricted, independent, compound symmetry, autoregressive 1) will be considered, and the model with the lowest AIC value will be selected. The following model will be used:

$$\mu_{ij} = \beta_0 + \beta_1 X_1 + \beta_2 X_2 + \beta_3 X_3 + \beta_4 X_4 + \beta_5 X_5 + \beta_6 X_6 + e_{ij}$$

where

- μ_{ij} is the difference between the observed MDS-UPDRS part III score for the jth visit (day 14, day 30, month 3, month 6) and the MDS-UPDRS part III score observed at baseline for i^{th} participant

- X_1 represents the baseline MDS-UPDRS part III score for the ith participant
- X_2 is an indicator term representing the center for the ith participant
- X_3 = 1 the record is from a day 14 visit, and 0 otherwise
- X_4 = 1 the record is from a day 30 visit, and 0 otherwise
- X_5 = 1 the record is from a 3 month visit, and 0 otherwise
- X_6 = 1 the record is from a 6 month visit, and 0 otherwise
- e_{ij} is random error for i^{th} participant from j^{th} visit

The desired test of interest can be obtained by testing the null hypothesis of the following contrast:

$$H_0: \beta_6 \leq -9.8 \text{ vs. } H_A: \beta_6 > -9.8$$

Rejecting the null hypothesis implies that a future study is unlikely to observe the large effects reported in the Pagan et al (2016) study. However, that would not rule out potentially meaningful effects on a smaller scale. Furthermore, note that failure to reject the null hypothesis does not imply that a significant difference has been observed. If the null hypothesis is not rejected, or if the hypothesis is rejected but the observed differences seem potentially meaningful, further study would be warranted.

14.3.4 OTHER SECONDARY OBJECTIVES

The study will also assess additional secondary objectives comparing the change in MDS-UPDRS scores over time between groups in order to establish both the potential degree of symptomatic effect of nilotinib and the impact of nilotinib on the progression of PD disability. These analyses will be examined using a model similar to that described in section 15.3.3, with additional terms added to represent the treatment group, post-treatment visits, and potential interaction terms. The interaction terms will be assessed initially. If significant, then the specific terms will be extracted for each comparison:

- To assess the impact of nilotinib on the progression of PD disability, we will examine the change in MDS-UPDRS from baseline to 6 months (evaluated for both the "OFF" and "ON" states
- To assess the degree of symptomatic effect, we will compare both:
    - Change in MDS-UPDRS from baseline to 1 month (MDS-UPDRS ON state)
- Change in MDS-UPDRS from 6 months (end of treatment) to 7 months (1 month post treatment) (MDS-UPDRS ON state)

If no significant interaction is observed, then it will be assumed that the effect of nilotinib is consistent over time and a single result will be provided.

14.3.5 EXPLORATORY OBJECTIVES

A number of additional exploratory objectives will be assessed in order to further examine the impact of nilotinib on disability, quality of life, and functional status. These will include, but are not limited to:

- Motor Disability
  - Change in MDS-UPDRS Total Score (Performed for ON and OFF scores)
  - Change in Ambulatory Capacity (sum of 5 MDS-UPDRS questions – falling, freezing, walking, gait, postural stability)
  - Analysis of ST utilization as measured by levodopa equivalence dose
  - Severity of motor complications as measured by MDS-UPDRS part IV subscale (complications of therapy)
- Cognitive Disability – Change in cognitive function as measured by the change in DRS-2 (Mattis dementia rating scale)
- Sleep Function – Change in sleep quality as measured by the Parkinson's disease sleep scale (PDSS)
- Measures of Global Disability – Clinician and patient global impression of change score (CGI)
- Measures of Functional Status and Quality of Life
  - Change in the MDS-UPDRS Motor and Non-Motor Experiences of Daily Living subscores
  - Change in the modified Schwab and England scale
  - Change in Parkinson Disease Quality of Life Questionnaire 39 (PDQ-39)
  - Change in European Quality of Life Scale (EQ-5D)

14.4 ANALYSIS PLAN – COHORT 2

14.4.1 PRIMARY HYPOTHESES

Primary Tolerability Hypotheses: ***To assess whether the dose selected from cohort 1 (either 150mg or 300mg once daily dosing of nilotinib) is sufficiently tolerable, compared to placebo***

The primary tolerability hypothesis for cohort 2 will be tested in a similar manner to that described above for cohort 1, with the exception that only a single test will be performed:

$$H_{01}: p_{selected} = p_P \quad vs. \quad H_{A1}: p_{selected} < p_P\ ,$$

where p_{selected} represents the true (unknown) percentage of participants treated with the nilotinib dose selected in cohort 1 who will tolerate the assigned medication. The hypothesis will be assessed via a logistic regression model, adjusted for center (stratification variable). Due to randomization, it is unlikely that important covariates will be imbalanced in this study. However, given the small sample size, this cannot be dismissed. We will assess for important baseline

imbalances, and if any imbalances exist, the logistic regression model will also adjust for the relevant covariates with important imbalances present.

Primary Safety Hypothesis: ***To assess whether there are clinically important increases in adverse experiences for the nilotinib-treated vs. placebo participants.***

The primary safety hypothesis will involve a comparison of the safety profiles for the group of participants treated with the chosen dose of nilotinib vs. placebo. This will be assessed in the same manner described above for cohort 1.

14.4.2 SECONDARY OBJECTIVES

The second cohort will also assess secondary objectives comparing the change in MDS-UPDRS scores over time between groups in order to establish both the potential degree of symptomatic effect of nilotinib and the impact of nilotinib on the progression of PD disability. These analyses will be examined using a model similar to that described in section 15.3.3, with additional terms added to represent the treatment group, additional visits due to extended follow-up time in the second cohort, post-treatment visits, and potential interaction terms. As with the first cohort, the interaction terms will be assessed initially. If significant, then the specific terms will be extracted for each comparison:

- To assess the impact of nilotinib on the progression of PD disability, we will examine the change in MDS-UPDRS from baseline to 12 months (evaluated for both the "OFF" and "ON" states
- To assess the degree of symptomatic effect, we will compare both:
    - Change in MDS-UPDRS from baseline to 1 month (MDS-UPDRS ON state)
- Change in MDS-UPDRS from 12 months (end of treatment) to 13 months (1 month post treatment) (MDS-UPDRS ON state)

If no significant interaction is observed, then it will be assumed that the effect of nilotinib is consistent over time and a single result will be provided.

Unlike the first cohort, the second cohort involves an early/de novo cohort of PD participants. It is expected that no participants will be on ST initially (as per inclusion/ exclusion criteria). However, it is expected that many will need to begin ST over the course of the 12 month follow-up period. Hence, we will perform this analysis three separate ways:

- Using ON scores for those on ST, combined with scores for those not yet having started ST, over the entire 12 month period
- Using OFF scores for those on ST, combined with scores for those not yet having started ST, over the entire 12 month period
- Using only MDS-UPDRS part III scores prior to initiation of ST for each participant. Note that this analysis will ignore all information collected post initiation of ST for each participant, and will thus involve much less data than the other two comparisons

14.4.3 EXPLORATORY OBJECTIVES

A number of additional exploratory objectives will be assessed in order to further examine the impact of nilotinib on disability, quality of life, and functional status. These will include, but are not limited to:

- Motor Disability
    - Change in MDS-UPDRS Total Score (Performed for ON and OFF scores)
    - Change in Ambulatory Capacity (sum of 5 MDS-UPDRS questions – falling, freezing, walking, gait, postural stability)
    - Analysis of ST utilization as measured by levodopa equivalence dose
    - Severity of motor complications as measured by MDS-UPDRS part IV subscale (complications of therapy)
- Cognitive Disability – Change in cognitive function as measured by the change in Mattis dementia rating scale (DRS-2)
- Sleep Function – Change in sleep quality as measured by the Parkinson's disease sleep scale (PDSS)
- Measures of Global Disability – Clinician and patient global impression of change score (CGI)
- Measures of Functional Status and Quality of Life
    - Change in the MDS-UPDRS Motor and Non-Motor Experiences of Daily Living subscores
    - Change in the modified Schwab and England scale
    - Change in Parkinson Disease Quality of Life Questionnaire 39 (PDQ-39)
    - Change in European Quality of Life Scale (EQ-5D)
    - Explore impact of nilotinib on:
        - Dopaminergic system as measured by an increase in dopamine and its metabolites in CSF (e.g., HVA level)
        - Other biomarkers linked to potential mechanism of neuroprotection (e.g., CSF alpha-synuclein, total tau, phospho-tau, cytokines, neurofilament, Neuron Specific Enolase/NSE, etc.)

14.5 SAMPLE SIZE JUSTIFICATION

14.5.1 SAMPLE SIZE – COHORT 1

For the purposes of computing the required sample size, we assumed that at least 90% of participants on placebo will meet the study definition of "tolerability" (completing the study on the assigned dose). Furthermore, it was determined that an absolute decrease of 30% or greater would provide sufficient tolerability concerns that would not warrant further study of

that dose. Hence, the sample size was chosen to provide sufficient power to compare an expected 90% tolerability rate in the placebo group versus a 60% or lower tolerability rate in each of the treatment groups. Under these assumptions, and considering a one-sided test with an alpha of 0.05, a total of 25 participants per group provides 80% power for the moderate/advanced cohort.

For the major secondary objective, we utilized preliminary estimates based on results from Table 2 in the Pagan et al (2016) study. Using these estimates, we computed the power of the study proposed for cohort 1 to detect "futility" based on comparisons of the change within the dosage groups themselves. Converting from the UPDRS used in the Pagan et al (2016) study to the MDS-UPDRS proposed for this study, the test is set up in the manner described in section 15.3.3. Furthermore, we believe that since the conversion factor maintains the relationships between the mean and standard deviation, although the expected values might differ the power calculations provided below give a valid estimate of the power for the proposed study. Specifically, we assume an alpha level of 0.10 for the one-sided futility hypothesis, and assume a standard deviation of 12.9. The table below shows the power, computed across a range of assumed values for the true change from baseline in the nilotinib treated participants. The table below demonstrates the benefits of using the futility hypothesis (with differences shown in both UPDRS and converted to MDS-UPDRS units). When the true change in MDS-UPDRS part III over the 6 month treatment period involves an increase in scores (implying progression of disease), the test will declare "futility" with high probability. Likewise, when the true change in MDS-UPDRS part III is equal to or greater than the observed change in the Pagan et al (2016) study, the study has a very low change of incorrectly declaring futility. If the observed change in the MDS-UPDRS part III is half that observed in the prior study (a reduction of 3.5), then the study has marginal power (52%) to declare futility. If there is no observed reduction associated with treatment, the study has 92% power to declare futility. Given the sample size limitations of this study, we feel that this provides a reasonable change of addressing the main futility question of interest regarding whether the large observed effect in the prior study can be refuted. The test of futility will have adequate power mainly if the direction of effect is in the opposite direction from that observed in the previous study (i.e., if there is no reduction, only a potentially smaller increase in MDS-UPDRS over time). These sample size calculations were based on the observed change for the 150mg group in the Pagan et al study[9]. Since bigger effects were observed over 6 months for the 300mg group, the study has adequate power for addressing futility in the 300mg group in a similar manner.

**True Change in UPDRS**	**-7.0**	**-3.5**	**0**	**+3.5**
**True Change in MDS-UPDRS**	**-9.8**	**-4.9**	**0**	**+4.9**
Pr(Futility)	10%	52%	92%	99%

14.5.2 SAMPLE SIZE – COHORT 2

For the purposes of computing the required sample size for cohort 2, we make similar assumptions to those stated above for cohort 1. Using these assumptions, a sample size of 40 participants on the selected dose from cohort 1 and 20 participants on placebo for the early/de novo cohort provides 80% power.

14.6 IMPACT OF MISSING DATA

The primary analysis will follow the intent-to-treat (ITT) paradigm. All enrolled participants must be included in the primary ITT analysis, and will be analyzed in the treatment group to which they were initially randomized. As such, it will be critically important to minimize the occurrence of missing data. Obviously, the optimal strategy for dealing with missing data is to make every effort to obtain complete data during the conduct of the study. Our team of data managers and protocol coordinators will work diligently and use a variety of methods in order to minimize the percentage of missing data in this trial. Nevertheless, there is likely to be a small percentage of missing data. For the primary analysis, we will take a conservative approach and assume that any participant not completing the study for any reason did not tolerate study therapy. In order to further assess the potential dependence of the results of the primary analysis to these missing values, a series of sensitivity analyses will be conducted. We will attempt to collect as much detailed information as possible for participants who are lost to follow-up or terminate the study early due to any reason. Any participants who terminate treatment or the study early due to tolerability concerns (AEs) will always be counted as not tolerating the dose. For participants that do not provide complete data, and for which there is no clear tolerability concern, we will analyze the data using the following strategies:

- Using Only Observed Data (No Imputation)
- Worst-case scenario: Assume all missing participants in the treatment group did not tolerate the dose and all missing participants in the placebo group did tolerate the dose
- Best-case scenario: Assume all missing participants in the rituximab group did tolerate the dose and all missing participants in the placebo group did not tolerate the dose.

The results of these analyses will provide important information regarding the sensitivity of the findings to the missing data, and will be critical to the Steering Committee in assessing the full value of the study results.

15. PHARMACOLOGY

15.1 PHARMACOLOGY PLAN

- Pharmacology Objectives
    - Primary Pharmacology Objective
        - To determine the serum PK of nilotinib in patients with Parkinson's disease.
    - Secondary Pharmacology Objectives
        - To investigate the ability of nilotinib to cross the blood brain barrier in Parkinson's disease patients at a dose(s) that is tolerable and safe as measured by dose-dependent increases in unbound nilotinib levels in the CSF.
    - Exploratory Pharmacology Objectives
        - To investigate the relationship between nilotinib exposures in serum and CSF with its ability to engage known molecular target(s) (measured by biomarkers of pathway inhibition in the blood and CSF (e.g., reduction in phosphor-cAbl).
        - To investigate factors that could affect the PK of nilotinib such as age, sex, PD severity, race/ethnicity.

15.2 PHARMACOKINETIC ANALYSIS

- Nilotinib serum and CSF concentrations will be assayed at a laboratory specified in the Laboratory Operational Manual
- For nilotinib serum PK, a nonlinear mixed-effects population PK model will be developed from the serum concentration-time data collected from study participants. The analysis will focus on characterizing the mean population-level PK parameters and variability of nilotinib in PD patients (e.g. apparent oral clearance, volume of distribution, etc.). Participant-level covariates will be incorporated into the population PK model to determine their effects on the PK parameters. Individual Bayesian estimates of nilotinib exposure (e.g. apparent oral clearance, Area Under the Concentration-Time curve, etc.) will be generated from the final population PK model to evaluate their association with pharmacodynamics markers.
- Nilotinib CSF concentrations will be summarized by geometric means (with coefficient of variations) and median values (with interquartile ranges) in aggregate, and compared by dose level.

16. REGULATORY/ETHICS

16.1 COMPLIANCE STATEMENT

This study will be conducted in accordance with the Good Clinical Practice (GCP) guidelines promulgated by the International Conference on Harmonization (ICH) and FDA, and any applicable national and local regulations including FDA regulations under 21 CFR Parts 11, 50, 54, 56, 312 and 314.

All procedures not described in this protocol will be performed according to the study Operations Manual unless otherwise stated. Laboratory tests/evaluations described in this protocol will be conducted in accordance with quality laboratory standards as described in the central laboratory manual, unless otherwise stated.

16.2 INFORMED CONSENT

This study will be conducted in accordance with the provisions of 21 Code of Federal Regulations (CFR) Part 50. Study Sponsor and CTCC must be given an opportunity to review the consent form prior to site IRB submission and before it is used in the study.

In accordance with relevant regulations, an informed consent agreement explaining the procedures and requirements of the study, together with any potential hazards/risks must be read and/or explained to each participant. Each participant (or participant's legally authorized representative, if applicable) will sign such an informed consent form or give verbal consent.

In this consent, the study must be adequately described as well as all procedures participants will need to do, how their confidentiality will be maintained, and explicitly ask for their consent to participate.

The participant must be assured of the freedom to withdraw from participation in the study at any time.

The consent process for each participant who signs informed consent will be documented in the participant's source documents (e.g., research file, research progress note) and should include the title of the study, that the consent was discussed with an opportunity for questions and answers, how the participant demonstrated comprehension, that the consent was signed prior to the first study procedure, and that the participant received a signed copy of the consent.

16.3 INSTITUTIONAL REVIEW BOARD

The Study Sponsor and CTCC will supply all necessary information to the Site Investigator for submission of the protocol and consent form to the IRB for review and approval. The Site Investigator agrees to provide the IRB all appropriate material. The trial will not begin until the

Site Investigator has obtained appropriate IRB approval. A copy of the approval letter listing all documents and versions approved, including the approved consent form, must be provided to the CTCC.

The Site Investigator will request a composition of IRB members reviewing the protocol and informed consent from the IRB. Appropriate reports on the progress of this study by the Site Investigator will be made to the IRB and the CTCC in accordance with institutional and government regulations. The Site Investigator is also responsible for annual renewal requests. The CTCC will notify the site when the IRB may be notified of study completion. It is the Site Investigator's responsibility to notify the IRB when the study ends. This includes study discontinuation, whether it is permanent or temporary. A copy of the site IRB acknowledgement of study completion must be provided to the CTCC.

The Site Investigator will discuss any proposed protocol changes with the CTCC Project Manager and no modifications will be made without prior written approval by CTCC and Study Sponsor (if applicable), except where clinical judgment requires an immediate change for reasons of participant welfare. The IRB will be informed of any amendments to the protocol or consent form, and approval, where and when appropriate, will be obtained before implementation.

16.4 PROTOCOL AMENDMENTS

Changes to the protocol should only be made via an approved protocol amendment. Protocol amendments must be approved by the Sponsor, the NILO-PD Steering Committee and each respective site's IRB prior to implementation, except when necessary to eliminate hazards and/or to protect the safety, rights or welfare of participants. (See Investigator's Agreement.)

16.5 PARTICIPANT CONFIDENTIALITY

The Site Investigator must assure that the privacy of participants, including their personal identity and personal medical information, will be maintained at all times. U.S. sites have additional privacy obligations to study participants under the Health Insurance Portability and Accountability Act (HIPAA). Participants will be identified by subject ID numbers on case report forms and other documents submitted to the Sponsor, SC and the CTCC.

After a participant signs an informed consent, it is required that the Site Investigator permit the study monitor, independent auditor or regulatory agency personnel to review the signed informed consent(s) and that portion of the participant's medical record that is directly related to the study. This shall include all study relevant documentation including participant medical history to verify eligibility, laboratory test result reports, admission/discharge summaries for hospital admissions occurring while the participant is in the study, and autopsy reports for deaths occurring during the study (when available).

The Participant's Authorization allows the Sponsor and CTCC to receive and review the Participants' protected health information that may be re-disclosed to any authorized representative of the Sponsor, CTCC or central laboratory facility for review of participant medical records in the context of the study.

17. DOCUMENTATION

17.1INVESTIGATOR SITE FILE

As part of the Trial Master File, the Site Investigator should have the following study documents accessible to the Monitor during the study:

1. Signed Form FDA 1572
2. *Curriculum vitae* for Site Investigator and staff listed on Form FDA 1572 signed and dated within 2 years of initiation of involvement in study
3. The signed IRB form/letter stating IRB approval of protocol, consent forms (including version), and advertisement notices, documentation of the IRB composition, and all IRB correspondence including notification/approval of protocol amendments, notification of serious adverse events to the IRB per local reporting requirements, and IRB notification of study termination
4. IRB approved consent form (sample) and advertisement materials
5. Signed protocol investigator agreement (and amendments, where applicable)
6. Signed and dated participant consent forms
7. Copies of the completed source document worksheets
8. Delegation Log with names, signatures, initials, and functional role of all persons completing protocol assessments, providing back-up to the Site Investigator and Coordinator, if applicable, as well as staff entering data to the EDC system. Training documents for delegated roles should be on file as well.
9. Copies of laboratory reports/printouts (*if applicable*)
10. Any source data/records not kept with the participant's hospital/medical records
11. Drug Accountability Log
12. Laboratory accreditation and relevant laboratory reference ranges (*if applicable)*
13. Signed and dated receipt of supplies
14. Record of all monitoring visits made to study sites by assigned study personnel
15. Copies of study correspondence to and from site personnel, Study Sponsor, CTCC, Clinical Trials Statistical Management Center at the University of Iowa and study vendors.
16. Record of any Corrective and Preventive Action Plans (CAPA) as required
17. Package Insert
18. Certificate for Human Subject Protection Program (HSPP) & Good Clinical Practive (GCP) training for each individual named on the Delegation Log and Form FDA 1572 who have direct participant contact

19. Copy of professional licensure/registration, as applicable, for each individual named on the Delegation Log, who has direct participant contact ensuring licensure is in the state in which the study will be conducted
20. A Note to File indicating the assessments that will be considered source documents, if applicable
21. Any other documentation as required by the Regulatory Authorities (e.g., Conflict-of-Interest/Financial Disclosure)

The Site Investigator must also retain all printouts/reports of tests/procedures, as specified in the protocol, for each participant. This documentation, together with the participant's hospital/medical records, is the participant's source data for the study.

17.2 MAINTENANCE AND RETENTION OF RECORDS

It is the responsibility of the Site Investigator to maintain a comprehensive and centralized filing system of all relevant documentation. At the end of the study, Site Investigators will be instructed to retain all study records required by the federal regulations in a secure and safe facility with limited access.

Regulations require retention for:

- A period of at least two years after notification from the Sponsor that a U.S. New Drug Application (NDA) has been approved for the indication that was investigated.
- Or if no NDA is filed or approved for such indication, a period of at least two years after the investigation is completed or discontinued and the FDA has been notified by the Sponsor.

The Site Investigator will be instructed to consult with CTCC before disposal of any study records and to notify CTCC of any change in the location, disposition, or custody of the study files.

17.3 QUALITY ASSURANCE (QA) AUDITS/SITE VISITS

During the course of the study, and after it has been completed, it is likely that one or more study site visits will be undertaken by authorized representatives of the CTCC.

The purpose of the audit is to determine whether or not the study is being, or has been, conducted and monitored in compliance with the protocol as well as recognized GCP guidelines and regulations. These audits will also increase the likelihood that the study data and all other study documentation can withstand a subsequent regulatory authority inspection.

If such audits are to occur, they will be arranged for a reasonable and agreed upon time.

17.4 REGULATORY INSPECTIONS

This study may be inspected by regulatory agencies, such as FDA. These inspections may take place at any time during or after the study and are based on the local regulations as well as ICH guidelines.

17.5 DATA MANAGEMENT

Utilizing Electronic Data Capture (EDC). An Internet accessible EDC system for data management will be utilized for this study. This system is protected by 128-bit server certificates and utilizes authenticated, password-protected accounts for each site. The EDC system is designed to ensure timeliness and accuracy of data as well as the prompt reporting of data from the study on an ongoing basis to the study principal and co-investigators. The system is compliant with relevant FDA regulatory requirements per 21 CFR Part 11.

The BCC will be responsible for design of the randomization scheme, creation of analytical databases, and the statistical analysis plan. Data management staff at the CTCC will be responsible for all data collection procedures.

Utilizing Electronic Data Capture (EDC): Data review, coding and query processing will be done through interaction with the CTCC, site personnel and the Study Monitor. Queries will be generated in real-time as the data is entered. Once data are submitted to the EDC system, it is immediately stored in the central study database located at the CTCC and are accessible for review by data management staff. Changes to the data will be fully captured in an electronic audit trail. As data recorded by sites in eCRFs are received, narrative text of adverse events and concomitant medications will be periodically coded using established coding mechanisms.

Utilizing Electronic Data Capture (EDC): The cycle of electronic data entry, review, query identification/resolution, and correction occurs over the course of the study period until all participants have completed the study and corresponding data has been entered into the EDC.

Data will be securely transferred to the BCC . Once the BCC and CTCC, in conjunction with the Sponsor and the Principal Investigator, agree that all queries have been adequately resolved and the database has been deemed "clean", the database will be officially signed off and deemed locked. All permissions to make changes (append, delete, modify or update) the database are removed at this time.

Site personnel, Sponsor, SC and CTCC staff will remain blinded as to treatment assignments until after database lock and treatment unblinding at the conclusion of each cohort (cohort 1 and

cohort 2). A designated statistician at the CTCC will be unblinded to treatment assignment for the purpose of confirming the balancing the randomization across sites.

18. SITE INVESTIGATOR/SITE

This study will be conducted under the supervision and direction of the Investigator(s) listed in Section 1 of the Form FDA 1572. Sub-investigators (if required and/or permitted by the Sponsor) are listed in Section 6 of the Form FDA 1572. The study will be conducted at the address (es) listed in Section 3 of the Form FDA 1572.

The Site Investigator must not conduct the study at any sites other than the one(s) stated on the Form FDA 1572.

The protocol, informed consent form, and advertisement notices will be approved by the IRB listed in Section 5 of the Form FDA 1572.

Each Site Investigator is responsible for providing copies of the protocol and all other information relating to the preclinical and prior clinical experience, which were furnished to him/her, to all physicians and other study personnel responsible to them who participate in this study. The Site Investigator will discuss this information with them to assure that they are adequately informed regarding the study drug and conduct of the study. The Site Investigator must assure that all study staff members are qualified by education, experience and training to perform their specific responsibilities.

19. CLINICAL SAFETY MONITORING

Refer to the Safety Management Plan for a detailed description of safety management

19.1 CLINICAL MONITOR

The Clinical Monitor (CM) is responsible for frequent review of blinded safety data including adverse events, serious adverse events, laboratory and EKG data. Additionally, the CM is responsible for assisting sites in responding appropriately to safety concerns. If the CM encounters significant safety concerns, they will discuss the severity, importance and relevance of the safety concern with the PI, the Site Investigator and the PM and work with all parties to determine appropriate action. Refer to the safety management plan for additional details on the Clinical Monitor responsibilities. All aspects of the study will be monitored by Authorized individuals in compliance with Good Clinical Practice (GCP) and applicable regulations.

19.2 INDEPENDENT MEDICAL MONITOR

The Independent Medical Monitor (IMM) will review all serious adverse events on occurrence and reporting by site personnel and classify the events according to the probability that the events are related to treatment, severity, and expectedness. The IMM may determine that the

Serious Adverse Event is serious, related and unexpected and requires expedited reporting to the FDA. The IMM may communicate with the CM and/or the Principal Investigator for clarification but should code events independent from CM and or Site Investigator. Consensus between the Site Investigator and the IMM is not required. In case of disagreement between Site Investigator and IMM, IMM adjudication prevails for the reporting purposes. The IMM will alert the CTCC PM and Principal Investigator of any concerns related to SAEs that should be forwarded to the entire DSMB for review via email. The DSMB may suggest changes to the protocol or consent form to the Principal Investigator as a consequence of adverse events.

IMM will also review the aggregate reports of all adverse events (serious/not serious, expected/unexpected and relationship to study drug) prepared by the DCC/BCC on an agreed upon basis or as requested. The events will be presented in tabular form and given to the IMM and the DSMB on a periodic basis as agreed upon or as requested. IMM will receive the reports by pseudo-blinded group allocation (e.g., A, B, C) unless full unbinding is necessary. IMM may participate in the closed DSMB sessions if requested by the DSMB Chair. Refer to the safety management plan for additional details on the IMM responsibilities.

19.3 STUDY COMMITTEES

19.3.1 STEERING COMMITTEE

The Steering Committee (SC) is composed of the Principal Investigator, Chief Biostatistician, Director of the Coordinating Centers or designees, Clinical Monitor(s), participant representative and independent investigator members of the Nilotinib Study Group with expertise in Parkinson's disease. The SC is responsible, along with the Sponsor, for the design of the study protocol and analysis plan, and oversees the clinical trial from conception to analysis and publication.

19.3.2 DATA AND SAFETY MONITORING BOARD (DSMB)

An independent DSMB will be appointed and will be responsible for periodic review of the trial conduct and progress and safety data (e.g., surveillance laboratory results, adverse events) throughout the trial. The frequency and format of the DSMB meetings, reports, and guidelines for interim analysis will be established and documented prior to study participant enrollment.

19.4 CASE REPORT FORMS

Utilizing Electronic Data Capture (EDC). Sites will enter participant information and data into an electronic case report form (eCRF) in the EDC application. The eCRFs are used to record study data and are an integral part of the study and subsequent reports. Therefore, the eCRFs must be completed for each participant screened or enrolled according to the participant's source data on a per-visit basis. Authorized study personnel will each be granted access to the electronic data capture tool via provision of a unique password-protected user-ID that will limit access to enter and view data specifically for participants enrolled at their site.

Data should be entered into the EDC system within 5 business days of a participant's visit.

Electronic Signatures:
An electronic signature from the Site Investigator is required on the following eCRFs:

- Signature Form
- Adverse Event Form
- Adverse Event Follow-up Log

It is the Site Investigator's responsibility to ensure that entries are proper and complete. During entry of data, error checks will be performed by the EDC that will immediately flag problematic data (i.e., missing, out of range, inconsistent) allowing for sites to correct the data at that time. Error checks will be implemented in the EDC based upon specifications defined in the data management plan.

The data entered from the eCRFs will be securely transmitted to a central database stored on a secure server located at the CTCC. Upon completion of a participant's visit or the study, sites have the option to print the completed eCRFs depicting the data that were entered.

At the conclusion of the study, the Data Management Team will provided by a PDF (portable document format) file on electronic media depicting eCRFs to each site. The PDF file should be printed for each participant participating in the study and filed in the participant's binder.

19.5 SITE MONITORING

To ensure compliance with GCPs and other applicable regulatory requirements, the Study Monitor or representative is responsible for monitoring that sites conduct the study according to the protocol, standard operating procedures, and other written instructions and regulatory guidelines. Please refer to the Site Monitoring Plan for detailed outline of the procedures.

Monitoring visits by a Study Monitor will be arranged in advance, at a mutually-acceptable time, with site personnel. The site personnel must allow sufficient time for the Study Monitor to review CRFs and relevant source documents and queries. The Site Coordinator and/or Site Investigator(s) should be available to answer questions or resolve data clarifications.

Remote monitoring will conducted at various intervals throughout the study and/or at study closeout, in which the site may be asked to supply source documentation to the Study Monitor or Project Manager.

19.6 PRIMARY SOURCE DOCUMENTS

The Site Investigator must maintain primary source documents supporting significant data for each participant in the participant's medical notes. These documents, which are considered 'source data', should include the following documentation:

- Demographic information
- Evidence supporting the diagnosis/condition for which the participant is being studied
- General information supporting the participant's consent to participate in the study
- General history and physical findings
- Hospitalization or Emergency Room records (if applicable)
- Each study visit by date, including any relevant findings/notes by the Site Investigator(s), occurrence (or lack) of adverse events, and changes in medication usage including the date the study drug commenced and completed
- Any additional visits during the study
- Any relevant telephone conversations with the participant regarding the study or possible adverse events, and attempts to reach participants by telephone or mail
- Original, signed informed consent forms for study participation

The Site Investigator must also retain all participant-specific printouts/reports of tests/procedures performed as a requirement of the study (e.g., laboratory and ECG reports). Laboratory reports from the central laboratory will be signed and dated by the Site Investigator following review and documentation of clinical significance (or not) of any abnormal findings. The reports will be filed with the participant's source documents. This documentation, together with the participant's hospital/site medical records, is the participant's 'source data' for the study. During monitoring visits, the Study Monitor will need to validate data in the eCRFs against these source data.

The CTCC will assist the sites in developing supplemental source document forms.

19.7 CLOSEOUT PLAN

Once all participants have completed all study visits and prior to database lock, study monitor(s) may conduct an on-site or remote monitoring closeout visit to ensure:

- all relevant study data has been retrieved
- all data queries have been resolved
- any protocol deviations are documented appropriately
- all major and significant protocol deviations have reported to the site's IRB
- study drug and clinical supplies have been/will be properly destroyed per instructions provided by the CMSU.

- unblinding documents/envelope have been retrieved and sent to the appropriate designated individual
- Site Investigator has copies of all study-related data/information on file.

Upon completion of the study protocol requirements, any remaining biospecimens shall be owned by the Michael J. Fox Foundation (MJFF) (the study funder) and transferred to a biorepository of its choosing. All study participants will be informed that their samples collected may be made available for sharing to the scientific community. MJFF shall be entitled to release biospecimens for any scientific research purpose related to advancing scientific understanding, cures, and therapies for Parkinson's disease and its complications, subject to applicable law, and as allowed by the signed informed consent forms. Any biospecimens will be released in compliance with all applicable laws, including those pertaining to patient privacy and IRB approval

20. PUBLICATION OF RESEARCH FINDINGS

Publication of the results of this trial will be governed by the policies and procedures developed by the NILO-PD Steering Committee and in accordance with the International Committee of Medical Journal Editors (ICJME) Uniform Requirements for Manuscripts Submitted to Biomedical Journals (http://www.icmje.org/urm_main.html). Any presentation, abstract, or manuscript will be made available for review by the Michael J. Fox Foundation, Novartis and the NILO-PD Steering Committee Members prior to submission. Study publication and authorship will be governed by the NILO-PD Steering Committee and Parkinson's Study Group (PSG) authorship policy.

21. DATA SHARING

MJFF is a public charity and research conducted with funds from MJFF must be conducted in the public interest. MJFF-funded studies require data sharing to advance scientific discovery. MJFF may make study data, such data not protected by patient privacy or other law, accessible to the scientific community through a secure medium for research purposes after the completion of the study. The data will contain no personal identifying information about study participants and all study participants will be informed that their collected de-identified data will be made available for sharing. The study data collected will be coded and may be used for future unspecified research purposes, for example to develop future studies of PD and related disorders. The research information collected will be labeled with a unique identification code. By using this code, the participant's identity will not be disclosed to any researchers using this data in the future. Data collected during the NILO-PD study (de identified) may also be shared with Novartis and its authorized agents.

22. REFERENCES

1. Hebron ML, Lonskaya I, Moussa CE. Nilotinib reverses loss of dopamine neurons and improves motor behavior via autophagic degradation of alpha-synuclein in Parkinson's disease models. *Hum Mol Genet.* 2013;22(16):3315-3328.
2. Hebron ML, Lonskaya I, Olopade P, Selby ST, Pagan F, Moussa CE. Tyrosine Kinase Inhibition Regulates Early Systemic Immune Changes and Modulates the Neuroimmune Response in alpha-Synucleinopathy. *J Clin Cell Immunol.* 2014;5:259.
3. Karuppagounder SS, Brahmachari S, Lee Y, Dawson VL, Dawson TM, Ko HS. The c-Abl inhibitor, nilotinib, protects dopaminergic neurons in a preclinical animal model of Parkinson's disease. *Sci Rep.* 2014;4:4874.
4. Lonskaya I, Hebron M, Chen W, Schachter J, Moussa C. Tau deletion impairs intracellular beta-amyloid-42 clearance and leads to more extracellular plaque deposition in gene transfer models. *Mol Neurodegener.* 2014;9:46.
5. Lonskaya I, Hebron ML, Desforges NM, Franjie A, Moussa CE. Tyrosine kinase inhibition increases functional parkin-Beclin-1 interaction and enhances amyloid clearance and cognitive performance. *EMBO Mol Med.* 2013;5(8):1247-1262.
6. Lonskaya I, Hebron ML, Desforges NM, Schachter JB, Moussa CE. Nilotinib-induced autophagic changes increase endogenous parkin level and ubiquitination, leading to amyloid clearance. *J Mol Med (Berl).* 2014;92(4):373-386.
7. Mahul-Mellier AL, Fauvet B, Gysbers A, et al. c-Abl phosphorylates alpha-synuclein and regulates its degradation: implication for alpha-synuclein clearance and contribution to the pathogenesis of Parkinson's disease. *Hum Mol Genet.* 2014;23(11):2858-2879.
8. Wenqiang C, Lonskaya I, Hebron ML, et al. Parkin-mediated reduction of nuclear and soluble TDP-43 reverses behavioral decline in symptomatic mice. *Hum Mol Genet.* 2014;23(18):4960-4969.
9. Pagan F, Hebron M, Valadez EH, et al. Nilotinib Effects in Parkinson's disease and Dementia with Lewy bodies. *J Parkinsons Dis.* 2016.
10. Wyse RK, Brundin P, Sherer TB. Nilotinib - Differentiating the Hope from the Hype. *J Parkinsons Dis.* 2016.
11. de Lau LM, Breteler MM. Epidemiology of Parkinson's disease. *Lancet neurology.* 2006;5(6):525-535.
12. Dorsey ER, Constantinescu R, Thompson JP, et al. Projected number of people with Parkinson disease in the most populous nations, 2005 through 2030. *Neurology.* 2007;68(5):384-386.
13. Twelves D, Perkins KS, Counsell C. Systematic review of incidence studies of Parkinson's disease. *Mov Disord.* 2003;18(1):19-31.
14. Braak H, Ghebremedhin E, Rub U, Bratzke H, Del Tredici K. Stages in the development of Parkinson's disease-related pathology. *Cell and tissue research.* 2004;318(1):121-134.
15. Huse DM, Schulman K, Orsini L, Castelli-Haley J, Kennedy S, Lenhart G. Burden of illness in Parkinson's disease. *Mov Disord.* 2005;20(11):1449-1454.

16. Dodel RC, Singer M, Kohne-Volland R, et al. The economic impact of Parkinson's disease. An estimation based on a 3-month prospective analysis. *PharmacoEconomics.* 1998;14(3):299-312.
17. Olanow CW, Watts RL, Koller WC. An algorithm (decision tree) for the management of Parkinson's disease (2001): treatment guidelines. *Neurology.* 2001;56(11 Suppl 5):S1-S88.
18. Suchowersky O, Gronseth G, Perlmutter J, Reich S, Zesiewicz T, Weiner WJ. Practice Parameter: neuroprotective strategies and alternative therapies for Parkinson disease (an evidence-based review): report of the Quality Standards Subcommittee of the American Academy of Neurology. *Neurology.* 2006;66(7):976-982.
19. Effects of tocopherol and deprenyl on the progression of disability in early Parkinson's disease. The Parkinson Study Group. *N Engl J Med.* 1993;328(3):176-183.
20. Jankovic J, Hunter C. A double-blind, placebo-controlled and longitudinal study of riluzole in early Parkinson's disease. *Parkinsonism & related disorders.* 2002;8(4):271-276.
21. Dopamine transporter brain imaging to assess the effects of pramipexole vs levodopa on Parkinson disease progression. *Jama.* 2002;287(13):1653-1661.
22. A controlled, randomized, delayed-start study of rasagiline in early Parkinson disease. *Arch Neurol.* 2004;61(4):561-566.
23. Fahn S, Oakes D, Shoulson I, et al. Levodopa and the progression of Parkinson's disease. *N Engl J Med.* 2004;351(24):2498-2508.
24. Whone AL, Watts RL, Stoessl AJ, et al. Slower progression of Parkinson's disease with ropinirole versus levodopa: The REAL-PET study. *Ann Neurol.* 2003;54(1):93-101.
25. Shults CW, Oakes D, Kieburtz K, et al. Effects of coenzyme Q10 in early Parkinson disease: evidence of slowing of the functional decline. *Arch Neurol.* 2002;59(10):1541-1550.
26. A randomized, double-blind, futility clinical trial of creatine and minocycline in early Parkinson disease. *Neurology.* 2006;66(5):664-671.
27. A randomized clinical trial of coenzyme Q10 and GPI-1485 in early Parkinson disease. *Neurology.* 2007;68(1):20-28.
28. Spillantini MG, Crowther RA, Jakes R, Hasegawa M, Goedert M. alpha-Synuclein in filamentous inclusions of Lewy bodies from Parkinson's disease and dementia with lewy bodies. *Proc Natl Acad Sci U S A.* 1998;95(11):6469-6473.
29. Spillantini MG, Schmidt ML, Lee VM, Trojanowski JQ, Jakes R, Goedert M. Alpha-synuclein in Lewy bodies. *Nature.* 1997;388(6645):839-840.
30. Mullin S, Schapira A. The genetics of Parkinson's disease. *Br Med Bull.* 2015;114(1):39-52.
31. Brundin P, Atkin G, Lamberts JT. Basic science breaks through: New therapeutic advances in Parkinson's disease. *Mov Disord.* 2015;30(11):1521-1527.
32. Xilouri M, Brekk OR, Stefanis L. Autophagy and Alpha-Synuclein: Relevance to Parkinson's Disease and Related Synucleopathies. *Mov Disord.* 2016;31(2):178-192.
33. Roskoski R, Jr. A historical overview of protein kinases and their targeted small molecule inhibitors. *Pharmacol Res.* 2015;100:1-23.
34. Turcotte S, Giaccia AJ. Targeting cancer cells through autophagy for anticancer therapy. *Curr Opin Cell Biol.* 2010;22(2):246-251.

35. Imam SZ, Trickler W, Kimura S, et al. Neuroprotective efficacy of a new brain-penetrating C-Abl inhibitor in a murine Parkinson's disease model. *PLoS One.* 2013;8(5):e65129.
36. Ko HS, Lee Y, Shin JH, et al. Phosphorylation by the c-Abl protein tyrosine kinase inhibits parkin's ubiquitination and protective function. *Proc Natl Acad Sci U S A.* 2010;107(38):16691-16696.
37. Tanabe A, Yamamura Y, Kasahara J, Morigaki R, Kaji R, Goto S. A novel tyrosine kinase inhibitor AMN107 (nilotinib) normalizes striatal motor behaviors in a mouse model of Parkinson's disease. *Front Cell Neurosci.* 2014;8:50.
38. Shin JH, Ko HS, Kang H, et al. PARIS (ZNF746) repression of PGC-1alpha contributes to neurodegeneration in Parkinson's disease. *Cell.* 2011;144(5):689-702.
39. Brahmachari S, Ge P, Lee SH, et al. Activation of tyrosine kinase c-Abl contributes to alpha-synuclein-induced neurodegeneration. *J Clin Invest.* 2016;126(8):2970-2988.
40. Tomlinson CL, Stowe R, Patel S, Rick C, Gray R, Clarke CE. Systematic review of levodopa dose equivalency reporting in Parkinson's disease. *Mov Disord.* 2010;25(15):2649-2653.
41. Hughes AJ, Daniel SE, Lees AJ. Improved accuracy of clinical diagnosis of Lewy body Parkinson's disease. *Neurology.* 2001;57(8):1497-1499.
42. The Columbia Suicide Severity Rating Scale.
43. Guidance for Industry Suicidality: Prospective Assessment of Occurrence in Clinical Trials, DRAFT GUIDANCE. 2010.
44. Goetz CG, Stebbins GT, Tilley BC. Calibration of unified Parkinson's disease rating scale scores to Movement Disorder Society-unified Parkinson's disease rating scale scores. *Movement disorders : official journal of the Movement Disorder Society.* 2012;27(10):1239-1242.

45. Yin OQ, Gallagher N, Li A, Zhou W, Harrell R, Schran H. Effect of grapefruit juice on the pharmacokinetics of nilotinib in healthy participants. J Clin *Pharmacol*. 2010; 50: 188-94.

APPENDIX 1: TASIGNA PACKAGE INSERT

HIGHLIGHTS OF PRESCRIBING INFORMATION
These highlights do not include all the information needed to use TASIGNA safely and effectively. See full prescribing information for TASIGNA.

TASIGNA® (nilotinib) capsules, for oral use
Initial U.S. Approval: 2007

WARNING: QT PROLONGATION AND SUDDEN DEATHS

See full prescribing information for complete boxed warning.

- **Tasigna prolongs the QT interval. Prior to Tasigna administration and periodically, monitor for hypokalemia or hypomagnesemia and correct deficiencies (5.2). Obtain ECGs to monitor the QTc at baseline, seven days after initiation, and periodically thereafter, and following any dose adjustments (5.2, 5.3, 5.7, 5.15).**
- **Sudden deaths have been reported in patients receiving nilotinib (5.3). Do not administer Tasigna to patients with hypokalemia, hypomagnesemia, or long QT syndrome (4, 5.2).**
- **Avoid use of concomitant drugs known to prolong the QT interval and strong CYP3A4 inhibitors (5.8).**
- **Avoid food 2 hours before and 1 hour after taking the dose (5.9).**

--------------------------RECENT MAJOR CHANGES----------------------------

Dosage and Administration, Discontinuation of treatment after a sustained molecular response (MR 4.5) on Tasigna (2.2)	12/2017
Dosage and Administration, Re-initiation of treatment in patients who lose molecular response after discontinuation of therapy with Tasigna (2.3)	12/2017
Warnings and Precautions, Embryo-fetal toxicity (5.17)	12/2017
Warnings and Precautions, Monitoring of BCR-ABL transcript levels (5.18)	12/2017

----------------------------INDICATIONS AND USAGE--------------------------

Tasigna is a kinase inhibitor indicated for:

The treatment of newly diagnosed adult patients with Philadelphia chromosome positive chronic myeloid leukemia (Ph+ CML) in chronic phase. (1.1)

The treatment of chronic phase (CP) and accelerated phase (AP) Ph+ CML in adult patients resistant to or intolerant to prior therapy that included imatinib. (1.2)

--------------------------DOSAGE AND ADMINISTRATION--------------------

- Recommended Dose: Newly diagnosed Ph+ CML-CP: 300 mg orally twice daily. Resistant or intolerant Ph+ CML-CP and CML-AP: 400 mg orally twice daily. (2.1)
- Take each Tasigna dose approximately 12 hours apart. Tasigna must be taken on an empty stomach. Avoid food for at least 2 hours before the dose is taken and avoid food for at least 1 hour after the dose is taken. (2.1)
- Swallow the capsules whole with water. (2.1)
- Eligible newly diagnosed patients with Ph+ CML-CP who have received Tasigna for a minimum of 3years and have achieved a sustained molecular response (MR4.5) and patients with Ph+ CML-CP resistant or intolerant to imatinib who have received Tasigna for at least 3 years and have achieved a sustained molecular response (MR4.5) may be considered for treatment discontinuation. (2.2, 2.3)
- Dose adjustment may be required for hematologic and non-hematologic toxicities, and drug interactions. (2.4)
- A lower starting dose is recommended in patients with hepatic impairment (at baseline). (2.4)

---------------------------DOSAGE FORMS AND STRENGTHS----------------

150 mg and 200 mg hard capsules (3)

---------------------------------CONTRAINDICATIONS----------------------------

Tasigna is contraindicated in patients with hypokalemia, hypomagnesemia, or long QT syndrome. (4)

-------------------------WARNINGS AND PRECAUTIONS---------------------

- Myelosuppression: Associated with neutropenia, thrombocytopenia and anemia. Obtain CBC every 2 weeks for the first 2 months, then monthly. Manage by treatment interruption or dose-reduction. (5.1)
- Cardiac and Arterial Vascular Occlusive Events: Cardiovascular events including ischemic heart disease, peripheral arterial occlusive disease and ischemic cerebrovascular events have been reported in patients with newly diagnosed Ph+ CML receiving Tasigna. Evaluate cardiovascular status, monitor and manage cardiovascular risk factors during Tasigna therapy. (5.4)
- Pancreatitis and elevated serum lipase: Monitor serum lipase monthly or as clinically indicated. In case lipase elevations are accompanied by abdominal symptoms, interrupt doses and consider appropriate diagnostics to exclude pancreatitis. (5.5)
- Hepatotoxicity: Tasigna may result in elevations in bilirubin, AST/ALT, and alkaline phosphatase. Monitor hepatic function tests monthly or as clinically indicated. (5.6)
- Electrolyte abnormalities: Tasigna can cause hypophosphatemia, hypokalemia, hyperkalemia, hypocalcemia, and hyponatremia. Correct electrolyte abnormalities prior to initiating Tasigna and monitor periodically during therapy. (5.7, 5.15)
- Tumor lysis syndrome: Tumor lysis syndrome cases have been reported in Tasigna treated patients with resistant or intolerant CML. Maintain adequate hydration and correct uric acid levels prior to initiating therapy with Tasigna. (5.11)
- Hemorrhage: Hemorrhage from any site may occur. Advise patients to report signs and symptoms of bleeding and medically manage as needed. (5.12)
- Total gastrectomy: May reduce exposure. Consider dose-increase and monitor for effectiveness more frequently. (5.13)
- Fluid retention: Pericardial effusion, pleural effusion, and severe fluid retention have occurred in patients receiving Tasigna. Monitor patients for signs and symptoms such as unexpected rapid weight gain, swelling, and shortness of breath. (5.16)
- Embryo-Fetal toxicity: Tasigna can cause fetal harm. Advise patients of potential risk to a fetus and to use effective contraception. (5.17, 8.1, 8.3)
- Treatment Discontinuation: Patients must have typical BCR-ABL transcripts. An FDA-authorized test with a detection limit below MR4.5 must be used to determine eligibility for discontinuation. Patients must be frequently monitored by the FDA authorized test to detect possible loss of remission. (5.18)

----------------------------------ADVERSE REACTIONS--------------------------

The most commonly reported non-hematologic adverse reactions (greater than or equal to 20%) in patients with newly diagnosed Ph+ CML-CP, resistant or intolerant Ph+ CML-CP, or resistant or intolerant Ph+ CML-AP) were nausea, rash, headache, fatigue, pruritus, vomiting, diarrhea, cough, constipation, arthralgia, nasopharyngitis, pyrexia, and night sweats. Hematologic adverse drug reactions include myelosuppression: thrombocytopenia, neutropenia and anemia. (6.1)

To report SUSPECTED ADVERSE REACTIONS, contact Novartis Pharmaceuticals Corporation at 1-888-669-6682 or FDA at 1-800-FDA-1088 or www.fda.gov/medwatch.

----------------------------------DRUG INTERACTIONS--------------------------

- Tasigna is an inhibitor of CYP3A4, CYP2C8, CYP2C9, and CYP2D6. It may also induce CYP2B6, CYP2C8 and CYP2C9. Therefore, Tasigna may alter serum concentration of other drugs (7.1)

-----------------------------USE IN SPECIFIC POPULATIONS-----------------

- Lactation: Advise women not to breastfeed. (8.2)

See 17 for PATIENT COUNSELING INFORMATION and Medication Guide

Revised: 12/2017

FULL PRESCRIBING INFORMATION: CONTENTS*

WARNING: QT PROLONGATION AND SUDDEN DEATHS

1 INDICATIONS AND USAGE
- 1.1 Newly Diagnosed Ph+ CML-CP
- 1.2 Resistant or Intolerant Ph+ CML-CP and CML-AP

2 DOSAGE AND ADMINISTRATION
- 2.1 Recommended Dosing
- 2.2 Discontinuation of treatment after a sustained molecular response (MR 4.5) on Tasigna
- 2.3 Re-initiation of treatment in patients who lose molecular response after discontinuation of therapy with Tasigna.
- 2.4 Dose Adjustments or Modifications

3 DOSAGE FORMS AND STRENGTHS

4 CONTRAINDICATIONS

5 WARNINGS AND PRECAUTIONS
- 5.1 Myelosuppression
- 5.2 QT Prolongation
- 5.3 Sudden Deaths
- 5.4 Cardiac and Arterial Vascular Occlusive Events
- 5.5 Pancreatitis and Elevated Serum Lipase
- 5.6 Hepatotoxicity
- 5.7 Electrolyte Abnormalities
- 5.8 Drug Interactions
- 5.9 Food Effects
- 5.10 Hepatic Impairment
- 5.11 Tumor Lysis Syndrome
- 5.12 Hemorrhage
- 5.13 Total Gastrectomy
- 5.14 Lactose
- 5.15 Monitoring Laboratory Tests
- 5.16 Fluid Retention
- 5.17 Embryo-Fetal Toxicity
- 5.18 Monitoring of BCR-ABL Transcript Levels

6 ADVERSE REACTIONS
- 6.1 Clinical Trials Experience

7 DRUG INTERACTIONS
- 7.1 Effects of Nilotinib on Drug Metabolizing Enzymes and Drug Transport Systems
- 7.2 Drugs that Inhibit or Induce Cytochrome P450 3A4 Enzymes
- 7.3 Drugs that Affect Gastric pH
- 7.4 Drugs that Inhibit Drug Transport Systems
- 7.5 Drugs that May Prolong the QT Interval

8 USE IN SPECIFIC POPULATIONS
- 8.1 Pregnancy
- 8.2 Lactation
- 8.3 Females and Males of Reproductive Potential
- 8.4 Pediatric Use
- 8.5 Geriatric Use
- 8.6 Cardiac Disorders
- 8.7 Hepatic Impairment

10 OVERDOSAGE

11 DESCRIPTION

12 CLINICAL PHARMACOLOGY
- 12.1 Mechanism of Action
- 12.3 Pharmacokinetics
- 12.5 Pharmacogenomics
- 12.6 QT/QTc Prolongation

13 NONCLINICAL TOXICOLOGY
- 13.1 Carcinogenesis, Mutagenesis, Impairment of Fertility

14 CLINICAL STUDIES
- 14.1 Newly Diagnosed Ph+ CML-CP
- 14.2 Patients with Resistant or Intolerant Ph+ CML-CP and CML-AP
- 14.3 Treatment discontinuation in newly diagnosed Ph+ CML-CP patients who have achieved a sustained molecular response (MR4.5)
- 14.4 Treatment discontinuation in Ph+ CML-CP patients who have achieved a sustained molecular response (MR4.5) on Tasigna following prior imatinib therapy

16 HOW SUPPLIED/STORAGE AND HANDLING

17 PATIENT COUNSELING INFORMATION

* Sections or subsections omitted from the full prescribing information are not listed

FULL PRESCRIBING INFORMATION

WARNING: QT PROLONGATION AND SUDDEN DEATHS

- **Tasigna prolongs the QT interval. Prior to Tasigna administration and periodically, monitor for hypokalemia or hypomagnesemia and correct deficiencies (5.2). Obtain ECGs to monitor the QTc at baseline, seven days after initiation, and periodically thereafter, and following any dose adjustments (5.2, 5.3, 5.7, 5.15).**
- **Sudden deaths have been reported in patients receiving Tasigna (5.3). Do not administer Tasigna to patients with hypokalemia, hypomagnesemia, or long QT syndrome (4, 5.2).**
- **Avoid use of concomitant drugs known to prolong the QT interval and strong CYP3A4 inhibitors (5.8).**
- **Avoid food 2 hours before and 1 hour after taking the dose (5.9).**

1 INDICATIONS AND USAGE

1.1 Newly Diagnosed Ph+ CML-CP

Tasigna (nilotinib) is indicated for the treatment of adult patients with newly diagnosed Philadelphia chromosome positive chronic myeloid leukemia (Ph+ CML) in chronic phase. The effectiveness of Tasigna is based on major molecular response and cytogenetic response rates *[see Clinical Studies (14.1)]*.

1.2 Resistant or Intolerant Ph+ CML-CP and CML-AP

Tasigna is indicated for the treatment of chronic phase and accelerated phase Philadelphia chromosome positive chronic myelogenous leukemia (Ph+ CML) in adult patients resistant or intolerant to prior therapy that included imatinib. The effectiveness of Tasigna is based on hematologic and cytogenetic response rates *[see Clinical Studies (14.2)]*.

2 DOSAGE AND ADMINISTRATION

2.1 Recommended Dosing

Tasigna should be taken twice daily at approximately 12-hour intervals and must be taken on an empty stomach. No food should be consumed for at least 2 hours before the dose is taken and for at least 1 hour after the dose is taken. Advise patients to swallow the capsules whole with water *[see Boxed Warning, Warnings and Precautions (5.9), Clinical Pharmacology (12.3)]*.

For patients who are unable to swallow capsules, the contents of each capsule may be dispersed in 1 teaspoon of applesauce (puréed apple). The mixture should be taken immediately (within 15 minutes) and should not be stored for future use *[see Clinical Pharmacology (12.3)]*.

Tasigna may be given in combination with hematopoietic growth factors such as erythropoietin or G-CSF if clinically indicated. Tasigna may be given with hydroxyurea or anagrelide if clinically indicated.

Dosage in Newly Diagnosed Ph+ CML-CP

The recommended dose of Tasigna is 300 mg orally twice daily *[see Clinical Pharmacology (12.3)]*.

Dosage in Resistant or Intolerant Ph+ CML-CP and CML-AP

The recommended dose of Tasigna (nilotinib) is 400 mg orally twice daily *[see Clinical Pharmacology (12.3)]*.

2.2 Discontinuation of treatment after a sustained molecular response (MR 4.5) on Tasigna

Patient Selection

Eligibility for Discontinuation of Treatment

Ph+ CML-CP patients with typical BCR-ABL transcripts who have been taking Tasigna for a minimum of 3 years and have achieved a sustained molecular response (MR4.5, corresponding to = BCR-ABL/ABL ≤ 0.0032% IS) may be eligible for treatment discontinuation *[see Clinical Studies (14.3, 14.4)]*. Information on FDA authorized tests for the detection and quantitation of BCR-ABL transcripts to determine eligibility for treatment discontinuation is available at http://www.fda.gov/Companion Diagnostics.

Patients with typical BCR-ABL transcripts (i.e., 13a2/b2a2 or e14a2/b3a2) who achieve the sustained MR4.5 criteria are eligible for discontinuation of Tasigna treatment. Patients must continue to be monitored for possible loss of molecular remission after treatment discontinuation. Use the same FDA authorized test to consistently monitor molecular response levels while on and off treatment.

Consider discontinuation of treatment in patients with newly diagnosed Ph+ CML-CP who have:

- been treated with Tasigna for at least 3 years
- maintained a molecular response of at least MR4.0 (corresponding to = BCR-ABL/ABL ≤ 0.01% IS) for one year prior to discontinuation of therapy
- achieved an MR4.5 for the last assessment taken immediately prior to discontinuation of therapy
- been confirmed to express the typical BCR-ABL transcripts (e13a2/b2a2 or e14a2/b3a2)
- no history of accelerated phase or blast crisis
- no history of prior attempts of treatment-free remission discontinuation that resulted in relapse.

Consider discontinuation of treatment in patients with Ph+ CML-CP that are resistant or intolerant to treatment with imatinib who have achieved a sustained molecular response (MR4.5) on Tasigna who have:

- been treated with Tasigna for a minimum of 3 years
- been treated with imatinib only prior to treatment with Tasigna
- achieved a molecular response of MR.4.5 (corresponding to = BCR-ABL/ABL ≤ 0.0032% IS)
- sustained an MR4.5 for a minimum of one year immediately prior to discontinuation of therapy
- been confirmed to express the typical BCR-ABL transcripts (e13a2/b2a2 or e14a2/b3a2)
- no history of accelerated phase or blast crisis
- no history of prior attempts of treatment-free remission discontinuation that resulted in relapse.

Monitor BCR-ABL transcript levels and complete blood count with differential in patients who have discontinued Tasigna therapy monthly for one year, then every 6 weeks for the second year, and every 12 weeks thereafter *[see Warnings and Precautions (5.18)]*.

Upon the loss of MR4.0 (corresponding to = BCR-ABL/ABL ≤ 0.01%IS) during the treatment-free phase, monitor BCR-ABL transcript levels every 2 weeks until BCR-ABL levels remain lower than major molecular response (MMR, corresponding to MR3.0 or = BCR-ABL/ABL ≤ 0.1%IS) for 4 consecutive measurements. The patient can then proceed to the original monitoring schedule.

2.3 Re-initiation of treatment in patients who lose molecular response after discontinuation of therapy with Tasigna.

- Newly diagnosed patients who lose MMR must re-initiate treatment within 4 weeks at the dose level prior to discontinuation of therapy *[see Warnings and Precautions (5.18)]*. Patients who re-initiate Tasigna therapy should have their BCR-ABL transcript levels monitored monthly until major molecular response is re-established and every 12 weeks thereafter.
- Patients resistant or intolerant to prior treatment that included imatinib with confirmed loss of MR4.0 (2 consecutive measures separated by at least 4 weeks showing loss of MR4.0) or loss of MMR must re-initiate treatment within 4 weeks at the dose level prior to discontinuation of therapy *[see Warnings and Precautions (5.18)]*. Patients who re-initiate Tasigna therapy should have their BCR-ABL transcript levels monitored monthly until previous major molecular response or MR 4.0 is re-established and every 12 weeks thereafter.

2.4 Dose Adjustments or Modifications

QT Interval Prolongation:

Table 1: Dose Adjustments for QT Prolongation

ECGs with a QTc greater than 480 msec	1. Withhold Tasigna, and perform an analysis of serum potassium and magnesium, and if below lower limit of normal, correct with supplements to within normal limits. Concomitant medication usage must be reviewed.<br>2. Resume within 2 weeks at prior dose if QTcF returns to less than 450 msec and to within 20 msec of baseline.<br>3. If QTcF is between 450 msec and 480 msec after 2 weeks, reduce the dose to 400 mg once daily.<br>4. Discontinue Tasigna if, following dose-reduction to 400 mg once daily, QTcF returns to greater than 480 msec.<br>5. An ECG should be repeated approximately 7 days after any dose adjustment.

Myelosuppression

Withhold or dose-reduce Tasigna for hematological toxicities (neutropenia, thrombocytopenia) that are not related to underlying leukemia (Table 2).

Table 2: Dose Adjustments for Neutropenia and Thrombocytopenia

Newly diagnosed Ph+ CML in chronic phase at 300 mg twice daily<br><br>Resistant or intolerant Ph+ CML in chronic phase or accelerated phase at 400 mg twice daily	ANC* less than 1.0 x 10^9/L and/or platelet counts less than 50 x 10^9/L	1. Stop Tasigna, and monitor blood counts<br>2. Resume within 2 weeks at prior dose if ANC greater than 1.0 x 10^9/L and platelets greater than 50 x 10^9/L<br>3. If blood counts remain low for greater than 2 weeks, reduce the dose to 400 mg once daily

*ANC=absolute neutrophil count

See Table 3 for dose adjustments for elevations of lipase, amylase, bilirubin, and/or hepatic transaminases *[see Adverse Reactions (6.1)]*.

Table 3: Dose Adjustments for Selected Non-Hematologic Laboratory Abnormalities

Elevated serum lipase or amylase greater than or equal to Grade 3	1. Withhold Tasigna, and monitor serum lipase or amylase<br>2. Resume treatment at 400 mg once daily if serum lipase or amylase returns to less than or equal to Grade 1
Elevated bilirubin greater than or equal to Grade 3	1. Withhold Tasigna, and monitor bilirubin<br>2. Resume treatment at 400 mg once daily if bilirubin returns to less than or equal to Grade 1
Elevated hepatic transaminases greater than or equal to Grade 3	1. Withhold Tasigna, and monitor hepatic transaminases<br>2. Resume treatment at 400 mg once daily if hepatic transaminases returns to less than or equal to Grade 1

Other Non-Hematologic Toxicities

If other clinically significant moderate or severe non-hematologic toxicity develops, withhold dosing, and resume at 400 mg once daily when the toxicity has resolved. If clinically appropriate, escalation of the dose back to 300 mg (newly diagnosed Ph+ CML-CP) or 400 mg (resistant or intolerant Ph+ CML-CP and CML-AP) twice daily should be considered. For Grade 3 to 4 lipase elevations, dosing should be withheld, and may be resumed at 400 mg once daily. Test serum lipase levels monthly or as clinically indicated. For Grade 3 to 4 bilirubin or hepatic transaminase elevations, dosing should be withheld, and may be resumed at 400 mg once daily. Test bilirubin and hepatic transaminases levels monthly or as clinically indicated *[see Warnings and Precautions (5.5, 5.6), Use in Specific Populations (8.7)]*.

Hepatic Impairment

If possible, consider alternative therapies. If Tasigna must be administered to patients with hepatic impairment, consider the following dose reduction:

Table 4: Dose Adjustments for Hepatic Impairment (At Baseline)

Newly diagnosed Ph+ CML in chronic phase at 300 mg twice daily	Mild, Moderate, or Severe*	An initial dosing regimen of 200 mg twice daily followed by dose escalation to 300 mg twice daily based on tolerability
Resistant or intolerant Ph+ CML in chronic phase or accelerated phase at 400 mg twice daily	Mild or Moderate*	An initial dosing regimen of 300 mg twice daily followed by dose escalation to 400 mg twice daily based on tolerability
	Severe*	A starting dose of 200 mg twice daily followed by a sequential dose escalation to 300 mg twice daily and then to 400 mg twice daily based on tolerability

*Mild=mild hepatic impairment (Child-Pugh Class A); Moderate=moderate hepatic impairment (Child-Pugh Class B); Severe=severe hepatic impairment (Child-Pugh Class C) *[see Warnings and Precautions (5.10), Use in Specific Populations (8.7)]*.

Concomitant Strong CYP3A4 Inhibitors

Avoid the concomitant use of strong CYP3A4 inhibitors (e.g., ketoconazole, itraconazole, clarithromycin, atazanavir, indinavir, nefazodone, nelfinavir, ritonavir, saquinavir, telithromycin, voriconazole). Avoid grapefruit products since they may also increase serum concentrations of nilotinib. Should treatment with any of these agents be required, therapy with Tasigna should be interrupted. If patients must be coadministered a

strong CYP3A4 inhibitor, based on pharmacokinetic studies, consider a dose reduction to 300 mg once daily in patients with resistant or intolerant Ph+ CML or to 200 mg once daily in patients with newly diagnosed Ph+ CML-CP. However, there are no clinical data with this dose adjustment in patients receiving strong CYP3A4 inhibitors. If the strong inhibitor is discontinued, a washout period should be allowed before the Tasigna dose is adjusted upward to the indicated dose. For patients who cannot avoid use of strong CYP3A4 inhibitors, monitor closely for prolongation of the QT interval *[see Boxed Warning, Warnings and Precautions (5.2, 5.8), Drug Interactions (7.2)]*.

Concomitant Strong CYP3A4 Inducers

Avoid the concomitant use of strong CYP3A4 inducers (e.g., dexamethasone, phenytoin, carbamazepine, rifampin, rifabutin, rifapentine, phenobarbital). Also inform patients not to take St. John's Wort since these agents may reduce the concentration of Tasigna. Based on the nonlinear pharmacokinetic profile of nilotinib, increasing the dose of Tasigna when coadministered with such agents is unlikely to compensate for the loss of exposure *[see Drug Interactions (7.2)]*.

3 DOSAGE FORMS AND STRENGTHS

150 mg red opaque hard gelatin capsules with black axial imprint "NVR/BCR."

200 mg light-yellow opaque hard gelatin capsules with a red axial imprint "NVR/TKI."

4 CONTRAINDICATIONS

Tasigna is contraindicated in patients with hypokalemia, hypomagnesemia, or long QT syndrome *[see Boxed Warning]*.

5 WARNINGS AND PRECAUTIONS

5.1 Myelosuppression

Treatment with Tasigna can cause Grade 3/4 thrombocytopenia, neutropenia and anemia. Perform complete blood counts every 2 weeks for the first 2 months and then monthly thereafter, or as clinically indicated. Myelosuppression was generally reversible and usually managed by withholding Tasigna temporarily or dose reduction *[see Dosage and Administration (2.4)]*.

5.2 QT Prolongation

Tasigna has been shown to prolong cardiac ventricular repolarization as measured by the QT interval on the surface ECG in a concentration-dependent manner *[see Adverse Reactions (6.1), Clinical Pharmacology (12.6)]*. Prolongation of the QT interval can result in a type of ventricular tachycardia called torsade de pointes, which may result in syncope, seizure, and/or death. ECGs should be performed at baseline, 7 days after initiation of Tasigna, and periodically as clinically indicated and following dose adjustments *[see Warnings and Precautions (5.15)]*.

Tasigna should not be used in patients who have hypokalemia, hypomagnesemia or long QT syndrome. Before initiating Tasigna and periodically, test electrolyte, calcium and magnesium blood levels. Hypokalemia or hypomagnesemia must be corrected prior to initiating Tasigna and these electrolytes should be monitored periodically during therapy *[see Warnings and Precautions (5.15)]*.

Significant prolongation of the QT interval may occur when Tasigna is inappropriately taken with food and/or strong CYP3A4 inhibitors and/or medicinal products with a known potential to prolong QT. Therefore, coadministration with food must be avoided and concomitant use with strong CYP3A4 inhibitors and/or medicinal products with a known potential to prolong QT should be avoided *[see Warnings and Precautions (5.8, 5.9)]*. The presence of hypokalemia and hypomagnesemia may further prolong the QT interval *[see Warnings and Precautions (5.7, 5.15)]*.

5.3 Sudden Deaths

Sudden deaths have been reported in 0.3% of patients with CML treated with nilotinib in clinical studies of 5,661 patients. The relative early occurrence of some of these deaths relative to the initiation of nilotinib suggests the possibility that ventricular repolarization abnormalities may have contributed to their occurrence.

5.4 Cardiac and Arterial Vascular Occlusive Events

Cardiovascular events, including arterial vascular occlusive events, were reported in a randomized, clinical trial in newly diagnosed CML patients and observed in the postmarketing reports of patients receiving nilotinib therapy. With a median time on therapy of 60 months in the clinical trial, cardiovascular events, including arterial vascular occlusive events, occurred in 9.3% and 15.2% of patients in the Tasigna 300 and 400 mg twice daily arms, respectively, and in 3.2% in the imatinib arm. These included cases of cardiovascular events including ischemic heart disease-related cardiac events (5.0% and 9.4% in the Tasigna 300 mg and 400 mg twice daily arms respectively, and 2.5% in the imatinib arm), peripheral arterial occlusive disease (3.6% and 2.9% in the Tasigna 300 mg and 400 mg twice daily arms respectively, and 0% in the imatinib arm), and ischemic cerebrovascular events (1.4% and 3.2% in the Tasigna 300 mg and 400 mg twice daily arms respectively, and 0.7% in the imatinib arm). If acute signs or symptoms of cardiovascular events occur, advise patients to seek immediate medical attention. The cardiovascular status of patients should be evaluated and cardiovascular risk factors should be monitored and actively managed during Tasigna therapy according to standard guidelines *[see Dosage and Administration (2.4)]*.

5.5 Pancreatitis and Elevated Serum Lipase

Tasigna can cause increases in serum lipase. Patients with a previous history of pancreatitis may be at greater risk of elevated serum lipase. If lipase elevations are accompanied by abdominal symptoms, interrupt dosing and consider appropriate diagnostics to exclude pancreatitis. Test serum lipase levels monthly or as clinically indicated.

5.6 Hepatotoxicity

Tasigna may result in hepatotoxicity as measured by elevations in bilirubin, AST/ALT, and alkaline phosphatase. Monitor hepatic function tests monthly or as clinically indicated *[see Warnings and Precautions (5.15)]*.

5.7 Electrolyte Abnormalities

The use of Tasigna can cause hypophosphatemia, hypokalemia, hyperkalemia, hypocalcemia, and hyponatremia. Correct electrolyte abnormalities prior to initiating Tasigna and during therapy. Monitor these electrolytes periodically during therapy *[see Warnings and Precautions (5.15)]*.

5.8 Drug Interactions

Avoid administration of Tasigna with agents that may increase nilotinib exposure (e.g., strong CYP3A4 inhibitors) or anti-arrhythmic drugs (including, but not limited to amiodarone, disopyramide, procainamide, quinidine and sotalol) and other drugs that may prolong QT interval (including, but not limited to chloroquine, clarithromycin, haloperidol, methadone, moxifloxacin and pimozide). Should treatment with any of these agents be required, interrupt therapy with Tasigna. If interruption of treatment with Tasigna is not possible, patients who require treatment with a drug that prolongs QT or strongly inhibits CYP3A4 should be closely monitored for prolongation of the QT interval *[see Boxed Warning, Dosage and Administration (2.4), Drug Interactions (7.2)]*.

5.9 Food Effects

The bioavailability of nilotinib is increased with food, thus Tasigna must not be taken with food. No food should be consumed for at least 2 hours before and for at least 1 hour after the dose is taken. Also avoid grapefruit products and other foods that are known to inhibit CYP3A4 *[see Boxed Warning, Drug Interactions (7.2) and Clinical Pharmacology (12.3)]*.

5.10 Hepatic Impairment

Nilotinib exposure is increased in patients with impaired hepatic function. Use a lower starting dose for patients with mild to severe hepatic impairment (at baseline) and monitor the QT interval frequently *[see Dosage and Administration (2.4) and Use in Specific Populations (8.7)]*.

5.11 Tumor Lysis Syndrome

Tumor lysis syndrome cases have been reported in Tasigna treated patients with resistant or intolerant CML. Malignant disease progression, high WBC counts and/or dehydration were present in the majority of these cases. Due to potential for tumor lysis syndrome, maintain adequate hydration and correct uric acid levels prior to initiating therapy with Tasigna.

5.12 Hemorrhage

Serious hemorrhagic events, including fatal events, have occurred in patients with CML treated with Tasigna. In a randomized trial in patients with newly diagnosed Ph+ CML in chronic phase comparing Tasigna and imatinib, Grade 3 or 4 hemorrhage occurred in 1.1% of patients in the Tasigna 300 mg twice daily arm, in 1.8% patients in the Tasigna 400 mg twice daily arm, and 0.4% of patients in the imatinib arm. GI hemorrhage occurred in 2.9% and 5.1% of patients in the Tasigna 300 mg twice daily and 400 mg twice daily arms and in 1.4% of patients in the imatinib arm, respectively. Grade 3 or 4 events occurred in 0.7% and 1.4% of patients in the Tasigna 300 mg twice daily and 400 mg twice daily arms, respectively, and in no patients in the imatinib arm. Monitor for signs and symptoms of bleeding and medically manage as needed.

5.13 Total Gastrectomy

Since the exposure of nilotinib is reduced in patients with total gastrectomy, perform more frequent monitoring of these patients. Consider dose increase or alternative therapy in patients with total gastrectomy *[see Clinical Pharmacology (12.3)]*.

5.14 Lactose

Since the capsules contain lactose, Tasigna is not recommended for patients with rare hereditary problems of galactose intolerance, severe lactase deficiency with a severe degree of intolerance to lactose-containing products, or of glucose-galactose malabsorption.

5.15 Monitoring Laboratory Tests

Complete blood counts should be performed every 2 weeks for the first 2 months and then monthly thereafter. Perform chemistry panels, including electrolytes, calcium, magnesium, liver enzymes, lipid profile, and glucose prior to therapy and periodically. ECGs should be obtained at baseline, 7 days after initiation and periodically thereafter, as well as following dose adjustments *[see Warnings and Precautions (5.2)]*. Monitor lipid profiles and glucose periodically during the first year of Tasigna therapy and at least yearly during chronic therapy. Should treatment with any HMG-CoA reductase inhibitor (a lipid lowering agent) be needed to treat lipid elevations, evaluate the potential for a drug-drug interaction before initiating therapy as certain HMG-CoA reductase inhibitors are metabolized by the CYP3A4 pathway *[see Drug Interactions (7.1)]*. Assess glucose levels before initiating treatment with Tasigna and monitor during treatment as clinically indicated. If test results warrant therapy, physician should follow their local standards of practice and treatment guidelines.

5.16 Fluid Retention

In the randomized trial in patients with newly diagnosed Ph+ CML in chronic phase, severe (Grade 3 or 4) fluid retention occurred in 3.9% and 2.9% of patients receiving Tasigna 300 mg twice daily and 400 mg twice daily, respectively, and in 2.5% of patients receiving imatinib. Effusions (including pleural effusion, pericardial effusion, ascites) or pulmonary edema, were observed in 2.2% and 1.1% of patients receiving Tasigna 300 mg twice daily and 400 mg twice daily, respectively, and in 2.1% of patients receiving imatinib. Effusions were severe (Grade 3 or 4) in 0.7% and 0.4% of patients receiving Tasigna 300 mg twice daily and 400 mg twice daily, respectively, and in no patients receiving imatinib. Similar events were also observed in postmarketing reports. Monitor patients for signs of severe fluid retention (e.g., unexpected rapid weight gain or swelling) and for symptoms of respiratory or cardiac compromise (e.g., shortness of breath) during Tasigna treatment; evaluate etiology and treat patients accordingly.

5.17 Embryo-Fetal Toxicity

Based on findings from animal studies and its mechanism of action, Tasigna can cause fetal harm when administered to a pregnant woman. In animal reproduction studies, administration of nilotinib to pregnant rats and rabbits during organogenesis caused adverse developmental outcomes including embryo-fetal lethality/fetal effects (small renal papilla, fetal edema, and skeletal variations) in rats and increased resorptions of fetuses and fetal skeletal variations in rabbits at maternal AUCs approximately 2 and 0.5 times, respectively, the AUC in patients receiving the recommended dose. Advise pregnant women of the potential risk to a fetus. Advise females of reproductive potential to use effective contraception during treatment and for at least 14 days after the last dose *[see Use in Specific Populations (8.1, 8.3) and Clinical Pharmacology (12.1)]*.

5.18 Monitoring of BCR-ABL Transcript Levels

Monitoring of BCR-ABL Transcript Levels in Patients Who Discontinued Tasigna

Monitor BCR-ABL transcript levels in patients eligible for treatment discontinuation using an FDA authorized test validated to measure molecular response levels with a sensitivity of at least MR4.5 (BCR-ABL/ABL ≤ 0.0032% IS). In patients who discontinue Tasigna therapy, assess BCR-ABL transcript levels monthly for one year, then every 6 weeks for the second year, and every 12 weeks thereafter during treatment discontinuation *[see Clinical Studies (14.3,14.4) and Dosage and Administration (2.2)]*.

Newly diagnosed patients must reinitiate Tasigna therapy within 4 weeks of a loss of Major Molecular Response (MMR, corresponding to MR3.0 or = BCR-ABL/ABL ≤ 0.1%IS).

Patients resistant or intolerant to prior treatment which included imatinib must reinitiate Tasigna therapy within 4 weeks of a loss of MMR or confirmed loss of MR4.0 (two consecutive measures separated by at least 4 weeks showing loss of MR4.0, corresponding to = BCR-ABL/ABL ≤ 0.01%IS).

For patients who fail to achieve MMR after three months of treatment re initiation, BCR-ABL kinase domain mutation testing should be performed.

Monitoring of BCR-ABL Transcript Levels in Patients who have Reinitiated Therapy after Loss of Molecular Response

Monitor CBC and BCR-ABL transcripts in patients who re-initiate treatment with Tasigna due to loss of molecular response quantitation every 4 weeks until a major molecular response is re-established, then every 12 weeks.

6 ADVERSE REACTIONS

The following serious adverse reactions can occur with Tasigna and are discussed in greater detail in other sections of labeling:

- Myelosuppression *[see Warnings and Precautions (5.1)]*
- QT Prolongation *[see Boxed Warning, Warnings and Precautions (5.2)]*
- Sudden Deaths *[see Boxed Warning, Warnings and Precautions (5.3)]*
- Cardiac and Arterial Vascular Occlusive Events *[see Warnings and Precautions (5.4)]*
- Pancreatitis and Elevated Serum Lipase *[see Warnings and Precautions (5.5)]*
- Hepatotoxicity *[see Warnings and Precautions (5.6)]*
- Electrolyte Abnormalities *[see Boxed Warning, Warnings and Precautions (5.7)]*
- Hemorrhage *[see Warnings and Precautions (5.12)]*
- Fluid Retention *[see Warnings and Precautions (5.16)]*

6.1 Clinical Trials Experience

Because clinical trials are conducted under widely varying conditions, adverse reaction rates observed in the clinical trials of a drug cannot be directly compared to rates in the clinical trials of another drug and may not reflect the rates observed in practice.

In Patients with Newly Diagnosed Ph+ CML-CP

The data below reflect exposure to Tasigna from a randomized trial in patients with newly diagnosed Ph+ CML in chronic phase treated at the recommended dose of 300 mg twice daily (n=279). The median time on treatment in the nilotinib 300 mg twice daily group was 61 months (range 0.1 to 71 months). The median actual dose intensity was 593 mg/day in the nilotinib 300 mg twice daily group.

The most common (greater than 10%) non-hematologic adverse drug reactions were rash, pruritus, headache, nausea, fatigue, alopecia, myalgia, and upper abdominal pain. Constipation, diarrhea, dry skin, muscle spasms, arthralgia, abdominal pain, peripheral edema, vomiting, and asthenia were observed less commonly (less than or equal to 10% and greater than 5%) and have been of mild-to-moderate severity, manageable and generally did not require dose reduction.

Increase in QTcF greater than 60 msec from baseline was observed in 1 patient (0.4%) in the 300 mg twice daily treatment group. No patient had an absolute QTcF of greater than 500 msec while on study drug.

The most common hematologic adverse drug reactions (all grades) were myelosuppression including: thrombocytopenia (18%), neutropenia (15%) and anemia (8%). See Table 7 for Grade 3/4 laboratory abnormalities.

Discontinuation due to adverse reactions, regardless of relationship to study drug, was observed in 10% of patients.

In Patients with Resistant or Intolerant Ph+ CML-CP and CML-AP

In the single open-label multicenter clinical trial, a total of 458 patients with Ph+ CML-CP and CML-AP resistant to or intolerant to at least one prior therapy including imatinib were treated (CML-CP=321; CML-AP=137) at the recommended dose of 400 mg twice daily.

The median duration of exposure in days for CML-CP and CML-AP patients is 561 (range 1 to 1096) and 264 (range 2 to 1160), respectively. The median dose intensity for patients with CML-CP and CML-AP is 789 mg/day (range 151 to 1110) and 780 mg/day (range 150 to 1149), respectively and corresponded to the planned 400 mg twice daily dosing.

The median cumulative duration in days of dose interruptions for the CML-CP patients was 20 (range 1 to 345), and the median duration in days of dose interruptions for the CML-AP patients was 23 (range 1 to 234).

In patients with CML-CP, the most commonly reported non-hematologic adverse drug reactions (greater than or equal to 10%) were rash, pruritus, nausea, fatigue, headache, constipation, diarrhea, vomiting and myalgia. The common serious drug-related adverse reactions (greater than or equal to 1% and less than 10%) were thrombocytopenia, neutropenia and anemia.

In patients with CML-AP, the most commonly reported non-hematologic adverse drug reactions (greater than or equal to 10%) were rash, pruritus and fatigue. The common serious adverse drug reactions (greater than or equal to 1% and less than 10%) were thrombocytopenia, neutropenia, febrile neutropenia, pneumonia, leukopenia, intracranial hemorrhage, elevated lipase and pyrexia.

Sudden deaths and QT prolongation were reported. The maximum mean QTcF change from baseline at steady-state was 10 msec. Increase in QTcF greater than 60 msec from baseline was observed in 4.1% of the patients and QTcF of greater than 500 msec was observed in 4 patients (less than 1%) *[see Boxed Warning, Warnings and Precautions (5.2, 5.3), Clinical Pharmacology (12.6)]*.

Discontinuation due to adverse drug reactions was observed in 16% of CML-CP and 10% of CML-AP patients.

Most Frequently Reported Adverse Reactions

Tables 5 and 6 show the percentage of patients experiencing non-hematologic adverse reactions (excluding laboratory abnormalities) regardless of relationship to study drug. Adverse reactions reported in greater than 10% of patients who received at least 1 dose of Tasigna are listed.

Table 5: Most Frequently Reported Non-Hematologic Adverse Reactions (Regardless of Relationship to Study Drug) in Patients with Newly Diagnosed Ph+ CML-CP (Greater than or equal to 10% in Tasigna 300 mg Twice Daily or Imatinib 400 mg Once Daily Groups) 60-Month Analysis[a]

		Patients with Newly Diagnosed Ph+ CML-CP			
		**TASIGNA 300 mg twice daily**	**Imatinib 400 mg once daily**	**TASIGNA 300 mg twice daily**	**Imatinib 400 mg once daily**
		**N=279**	**N=280**	**N=279**	**N=280**
**Body System and Adverse Reaction**		**All Grades (%)**		**CTC Grades[b] 3/4 (%)**	
Skin and subcutaneous tissue disorders	Rash	38	19	<1	2
	Pruritus	21	7	<1	0
	Alopecia	13	7	0	0
	Dry skin	12	6	0	0
Gastrointestinal disorders	Nausea	22	41	2	2
	Constipation	20	8	<1	0
	Diarrhea	19	46	1	4
	Vomiting	15	27	<1	<1
	Abdominal pain upper	18	14	1	<1
	Abdominal pain	15	12	2	0
	Dyspepsia	10	12	0	0
Nervous system disorders	Headache	32	23	3	<1
	Dizziness	12	11	<1	<1
General disorders and administration site conditions	Fatigue	23	20	1	1
	Pyrexia	14	13	<1	0
	Asthenia	14	12	<1	0
	Peripheral edema	9	20	<1	0
	Face edema	<1	14	0	<1
Musculoskeletal and connective tissue disorders	Myalgia	19	19	<1	<1
	Arthralgia	22	17	<1	<1
	Muscle spasms	12	34	0	1
	Pain in extremity	15	16	<1	<1
	Back pain	19	17	1	1
Respiratory, thoracic and mediastinal disorders	Cough	17	13	0	0
	Oropharyngeal pain	12	6	0	0
	Dyspnea	11	6	2	<1
Infections and infestations	Nasopharyngitis	27	21	0	0
	Upper respiratory tract infection	17	14	<1	0
	Influenza	13	9	0	0
	Gastroenteritis	7	10	0	<1
Eye disorders	Eyelid edema	1	19	0	<1
	Periorbital edema	<1	15	0	0
Psychiatric disorders	Insomnia	11	9	0	0
Vascular disorder	Hypertension	10	4	1	<1

[a]Excluding laboratory abnormalities
[b]NCI Common Terminology Criteria for Adverse Events, Version 3.0

Table 6: Most Frequently Reported Non-Hematologic Adverse Reactions in Patients with Resistant or Intolerant Ph+ CML Receiving TASIGNA 400 mg Twice Daily (Regardless of Relationship to Study Drug) (Greater than or equal to 10% in any Group) 24-Month Analysis[a]

Body System and Adverse Reaction		CML-CP N=321		CML-AP N=137	
		All Grades (%)	CTC Grades[b] 3/4 (%)	All Grades (%)	CTC Grades[b] 3/4 (%)
Skin and subcutaneous tissue disorders	Rash	36	2	29	0
	Pruritus	32	<1	20	0
	Night sweat	12	<1	27	0
	Alopecia	11	0	12	0
Gastrointestinal disorders	Nausea	37	1	22	<1
	Constipation	26	<1	19	0
	Diarrhea	28	3	24	2
	Vomiting	29	<1	13	0
	Abdominal pain	15	2	16	3
	Abdominal pain upper	14	<1	12	<1
	Dyspepsia	10	<1	4	0
Nervous system disorders	Headache	35	2	20	1
General disorders and administration site conditions	Fatigue	32	3	23	<1
	Pyrexia	22	<1	28	2
	Asthenia	16	0	14	1
	Peripheral edema	15	<1	12	0
Musculoskeletal and connective tissue disorders	Myalgia	19	2	16	<1
	Arthralgia	26	2	16	0
	Muscle spasms	13	<1	15	0
	Bone pain	14	<1	15	2
	Pain in extremity	20	2	18	1
	Back pain	17	2	15	<1
	Musculoskeletal pain	11	<1	12	1
Respiratory, thoracic and mediastinal disorders	Cough	27	<1	18	0
	Dyspnea	15	2	9	2
	Oropharyngeal pain	11	0	7	0
Infections and infestations	Nasopharyngitis	24	<1	15	0
	Upper respiratory tract infection	12	0	10	0
Metabolism and nutrition disorders	Decreased appetite[c]	15	<1	17	<1
Psychiatric disorders	Insomnia	12	1	7	0
Vascular disorders	Hypertension	10	2	11	<1

[a]Excluding laboratory abnormalities [b]NCI Common Terminology Criteria for Adverse Events, Version 3.0 [c]Also includes preferred term anorexia

Laboratory Abnormalities

Table 7 shows the percentage of patients experiencing treatment-emergent Grade 3/4 laboratory abnormalities in patients who received at least one dose of Tasigna.

Table 7: Percent Incidence of Clinically Relevant Grade 3/4* Laboratory Abnormalities

	**Patient Population**			
	**Newly Diagnosed Ph+ CML-CP**		**Resistant or Intolerant Ph+**	
			**CML-CP**	**CML-AP**
	**TASIGNA 300 mg twice daily N=279 (%)**	**Imatinib 400 mg once daily N=280 (%)**	**TASIGNA 400 mg twice daily N=321 (%)**	**TASIGNA 400 mg twice daily N=137 (%)**
**Hematologic Parameters**				
Thrombocytopenia	10	9	30[1]	42[3]
Neutropenia	12	22	31[2]	42[4]
Anemia	4	6	11	27
**Biochemistry Parameters**				
Elevated lipase	9	4	18	18
Hyperglycemia	7	<1	12	6
Hypophosphatemia	8	10	17	15
Elevated bilirubin (total)	4	<1	7	9
Elevated SGPT (ALT)	4	3	4	4
Hyperkalemia	2	1	6	4
Hyponatremia	1	<1	7	7
Hypokalemia	<1	2	2	9
Elevated SGOT (AST)	1	1	3	2
Decreased albumin	0	<1	4	3
Hypocalcemia	<1	<1	2	5
Elevated alkaline phosphatase	0	<1	<1	1
Elevated creatinine	0	<1	<1	<1

*NCI Common Terminology Criteria for Adverse Events, version 3.0
[1]CML-CP: Thrombocytopenia: 12% were Grade 3, 18% were Grade 4
[2]CML-CP: Neutropenia: 16% were Grade 3, 15% were Grade 4
[3]CML-AP: Thrombocytopenia: 11% were Grade 3, 32% were Grade 4
[4]CML-AP: Neutropenia: 16% were Grade 3, 26% were Grade 4

Elevated total cholesterol (all grades) occurred in 28% (Tasigna 300 mg twice daily) and 4% (imatinib). Elevated triglycerides (all grades) occurred in 12% and 8% of patients in the Tasigna and imatinib arms, respectively. Hyperglycemia (all Grades) occurred in 50% and 31% of patients in the Tasigna and imatinib arms, respectively.

Most common biochemistry laboratory abnormalities (all grades) were alanine aminotransferase increased (72%), blood bilirubin increased (59%), aspartate aminotransferase increased (47%), lipase increased (28%), blood glucose increased (50%), blood cholesterol increased (28%), and blood triglyceride increased (12%).

Treatment discontinuation in Ph+ CML-CP patients who have achieved a sustained molecular response (MR 4.5)

In eligible patients who discontinued Tasigna therapy after attaining a sustained molecular response (MR4.5), musculoskeletal symptoms (e.g. myalgia, pain in extremity, arthralgia, bone pain, spinal pain, or musculoskeletal pain), were reported more frequently than before treatment discontinuation in the first year, as noted in Table 8. The rate of new musculoskeletal symptoms generally decreased in the second year after treatment discontinuation.

In the newly diagnosed population in whom musculoskeletal symptoms occurred at any time during the TFR phase, 23/53 (43.4%) had not resolved by the TFR end date or data cut-off date. In the population previously treated with imatinib in whom musculoskeletal events occurred at any time during the TFR phase, 32/57 (56.1%) had not resolved by the data cut-off date.

The rate of musculoskeletal symptoms decreased in patients who entered the nilotinib treatment re-initiation (NTRI) phase, at 11/88 (12.5%) in the newly diagnosed population and 14/56 (25%) in the population previously treated with imatinib. Other adverse reactions observed in the Tasigna re-treatment phase were similar to those observed Tasigna use in patients with newly diagnosed Ph+ CML-CP and resistant or intolerant Ph+ CML-CP and CML-AP.

Table 8: Musculoskeletal symptoms occurring upon treatment discontinuation in the context of treatment-free remission (TFR)

	Entire TFR period in all TFR patients				By time interval, in subset of patients in TFR greater than 48 weeks						
Ph+ CML-CP patients	N	Median follow-up in TFR	Patients with musculoskeletal symptoms		N	Year prior to TASIGNA discontinuation		1st year after TASIGNA discontinuation		2nd year after TASIGNA discontinuation	
			All Grades	Grade 3/4		All Grades	Grade 3/4	All Grades	Grade 3/4	All Grades	Grade 3/4
Newly Diagnosed	190	76 weeks	27.9%	1.1%	100	17.0%	0%	34.0%	2.0%	9.0%	0%
Previously treated with imatinib	126	99 weeks	45.2%	2.4%	73	13.7%	0%	47.9%	2.7%	15.1%	1.4%

Additional Data from Clinical Trials

The following adverse drug reactions were reported in patients in the Tasigna clinical studies at the recommended doses. These adverse drug reactions are ranked under a heading of frequency, the most frequent first using the following convention: common (greater than or equal to1% and less than 10%), uncommon (greater than or equal to 0.1% and less than 1%), and unknown frequency (single events). For laboratory abnormalities, very common events (greater than or equal to 10%), which were not included in Tables 5 and 6, are also reported. These adverse reactions are included based on clinical relevance and ranked in order of decreasing seriousness within each category, obtained from 2 clinical studies:

1. Newly diagnosed Ph+ CML-CP 60 month analysis and,
2. Resistant or intolerant Ph+ CML-CP and CMP-AP 24 months' analysis.

Infections and Infestations: Common: folliculitis. Uncommon: pneumonia, bronchitis, urinary tract infection, candidiasis (including oral candidiasis). Unknown frequency: hepatitis B reactivation, sepsis, subcutaneous abscess, anal abscess, furuncle, tinea pedis.

Neoplasms Benign, Malignant, and Unspecified: Common: skin papilloma. Unknown frequency: oral papilloma, paraproteinemia.

Blood and Lymphatic System Disorders: Common: leukopenia, eosinophilia, febrile neutropenia, pancytopenia, lymphopenia. Unknown frequency: thrombocythemia, leukocytosis.

Immune System Disorders: Unknown frequency: hypersensitivity.

Endocrine Disorders: Uncommon: hyperthyroidism, hypothyroidism. Unknown frequency: hyperparathyroidism secondary, thyroiditis.

Metabolism and Nutrition Disorders: Very Common: hypophosphatemia. Common: electrolyte imbalance (including hypomagnesemia, hyperkalemia, hypokalemia, hyponatremia, hypocalcemia, hypercalcemia, hyperphosphatemia), diabetes mellitus, hyperglycemia, hypercholesterolemia, hyperlipidemia,

hypertriglyceridemia. Uncommon: gout, dehydration, increased appetite. Unknown frequency: hyperuricemia, hypoglycemia.

Psychiatric Disorders: Common: depression, anxiety. Unknown frequency: disorientation, confusional state, amnesia, dysphoria.

Nervous System Disorders: Common: peripheral neuropathy, hypoesthesia, paresthesia. Uncommon: intracranial hemorrhage, ischemic stroke, transient ischemic attack, cerebral infarction, migraine, loss of consciousness (including syncope), tremor, disturbance in attention, hyperesthesia. Unknown frequency: basilar artery stenosis, brain edema, optic neuritis, lethargy, dysesthesia, restless legs syndrome.

Eye Disorders: Common: eye hemorrhage, eye pruritus, conjunctivitis, dry eye (including xerophthalmia). Uncommon: vision impairment, vision blurred, visual acuity reduced, photopsia, hyperemia (scleral, conjunctival, ocular), eye irritation, conjunctival hemorrhage. Unknown frequency: papilledema, diplopia, photophobia, eye swelling, blepharitis, eye pain, chorioretinopathy, conjunctivitis allergic, ocular surface disease.

Ear and Labyrinth Disorders: Common: vertigo. Unknown frequency: hearing impaired, ear pain, tinnitus.

Cardiac Disorders: Common: angina pectoris, arrhythmia (including atrioventricular block, cardiac flutter, extrasystoles, atrial fibrillation, tachycardia, bradycardia), palpitations, electrocardiogram QT prolonged. Uncommon: cardiac failure, myocardial infarction, coronary artery disease, cardiac murmur, coronary artery stenosis, myocardial ischemia, pericardial effusion, cyanosis. Unknown frequency: ventricular dysfunction, pericarditis, ejection fraction decrease.

Vascular Disorders: Common: flushing. Uncommon: hypertensive crisis, peripheral arterial occlusive disease, intermittent claudication, arterial stenosis limb, hematoma, arteriosclerosis. Unknown frequency: shock hemorrhagic, hypotension, thrombosis, peripheral artery stenosis.

Respiratory, Thoracic and Mediastinal Disorders: Common: dyspnea exertional, epistaxis, dysphonia. Uncommon: pulmonary edema, pleural effusion, interstitial lung disease, pleuritic pain, pleurisy, pharyngolaryngeal pain, throat irritation. Unknown frequency: pulmonary hypertension, wheezing.

Gastrointestinal Disorders: Common: pancreatitis, abdominal discomfort, abdominal distension, dysgeusia, flatulence. Uncommon: gastrointestinal hemorrhage, melena, mouth ulceration, gastroesophageal reflux, stomatitis, esophageal pain, dry mouth, gastritis, sensitivity of teeth. Unknown frequency: gastrointestinal ulcer perforation, retroperitoneal hemorrhage, hematemesis, gastric ulcer, esophagitis ulcerative, subileus, enterocolitis, hemorrhoids, hiatus hernia, rectal hemorrhage, gingivitis.

Hepatobiliary Disorders: Very Common: hyperbilirubinemia. Common: hepatic function abnormal. Uncommon: hepatotoxicity, toxic hepatitis, jaundice. Unknown frequency: cholestasis, hepatomegaly.

Skin and Subcutaneous Tissue Disorders: Common: eczema, urticaria, erythema, hyperhidrosis, contusion, acne, dermatitis (including allergic, exfoliative and acneiform). Uncommon: exfoliative rash, drug eruption, pain of skin, ecchymosis. Unknown frequency: psoriasis, erythema multiforme, erythema nodosum, skin ulcer, palmar-plantar erythrodysesthesia syndrome, petechiae, photosensitivity, blister, dermal cyst, sebaceous hyperplasia, skin atrophy, skin discoloration, skin exfoliation, skin hyperpigmentation, skin hypertrophy, hyperkeratosis.

Musculoskeletal and Connective Tissue Disorders: Common: bone pain, musculoskeletal chest pain, musculoskeletal pain, back pain, neck pain, flank pain, muscular weakness. Uncommon: musculoskeletal stiffness, joint swelling. Unknown frequency: arthritis.

Renal and Urinary Disorders: Common: pollakiuria. Uncommon: dysuria, micturition urgency, nocturia. Unknown frequency: renal failure, hematuria, urinary incontinence, chromaturia.

Reproductive System and Breast Disorders: Uncommon: breast pain, gynecomastia, erectile dysfunction. Unknown frequency: breast induration, menorrhagia, nipple swelling.

General Disorders and Administration Site Conditions: Common: pyrexia, chest pain (including non-cardiac chest pain), pain, chest discomfort, malaise. Uncommon: gravitational edema, influenza-like illness, chills, feeling body temperature change (including feeling hot, feeling cold). Unknown frequency: localized edema.

Investigations: Very Common: alanine aminotransferase increased, aspartate aminotransferase increased, lipase increased, lipoprotein cholesterol (including very low density and high density) increased, total cholesterol increased, blood triglycerides increased. Common: hemoglobin decreased, blood amylase increased, gamma-glutamyltransferase increased, blood creatinine phosphokinase increased, blood alkaline phosphatase increased, weight decreased, weight increased, globulins decreased. Uncommon: blood lactate dehydrogenase increased, blood urea increased. Unknown frequency: troponin increased, blood bilirubin unconjugated increased, insulin C-peptide decreased, blood parathyroid hormone increased.

7 DRUG INTERACTIONS

7.1 Effects of Nilotinib on Drug Metabolizing Enzymes and Drug Transport Systems

Nilotinib is a competitive inhibitor of CYP3A4, CYP2C8, CYP2C9, CYP2D6 and UGT1A1 in vitro, potentially increasing the concentrations of drugs eliminated by these enzymes. In vitro studies also suggest that nilotinib may induce CYP2B6, CYP2C8 and CYP2C9, and decrease the concentrations of drugs which are eliminated by these enzymes.

In patients with CML, multiple doses of Tasigna increased the systemic exposure of oral midazolam (a substrate of CYP3A4) 2.6-fold. Tasigna is a moderate CYP3A4 inhibitor. As a result, the systemic exposure of drugs metabolized by CYP3A4 (e.g., certain HMG-CoA reductase inhibitors) may be increased when coadministered with Tasigna. Dose adjustment may be necessary for drugs that are CYP3A4 substrates, especially those that have narrow therapeutic indices (e.g., alfentanil, cyclosporine, dihydroergotamine, ergotamine, fentanyl, sirolimus and tacrolimus) when coadministered with Tasigna.

Single-dose administration of Tasigna to healthy subjects did not change the pharmacokinetics and pharmacodynamics of warfarin (a CYP2C9 substrate). The ability of multiple doses of Tasigna to induce metabolism of drugs other than midazolam has not been determined in vivo. Monitor patients closely when coadministering Tasigna with drugs that have a narrow therapeutic index and are substrates for CYP2B6, CYP2C8, or CYP2C9 enzymes.

Nilotinib inhibits human P-glycoprotein (P-gp). If Tasigna is administered with drugs that are substrates of P-gp, increased concentrations of the substrate drug are likely, and caution should be exercised.

7.2 Drugs that Inhibit or Induce Cytochrome P450 3A4 Enzymes

Nilotinib undergoes metabolism by CYP3A4, and concomitant administration of strong inhibitors or inducers of CYP3A4 can increase or decrease nilotinib concentrations significantly. Avoid the administration of Tasigna with agents that are strong CYP3A4 inhibitors *[see Boxed Warning, Dosage and Administration (2.4), Warnings and Precautions (5.2, 5.8)]*. Concomitant use of Tasigna with medicinal products and herbal preparations that are potent inducers of CYP3A4 is likely to reduce exposure to nilotinib to a clinically relevant extent. Therefore, in patients receiving Tasigna, select alternative therapeutic agents with less potential for CYP3A4 induction for concomitant use.

<u>Ketoconazole</u>: In healthy subjects receiving ketoconazole, a CYP3A4 inhibitor, at 400 mg once daily for 6 days, systemic exposure (AUC) to nilotinib was increased approximately 3-fold.

<u>Rifampicin</u>: In healthy subjects receiving the CYP3A4 inducer, rifampicin, at 600 mg daily for 12 days, systemic exposure (AUC) to nilotinib was decreased approximately 80%.

7.3 Drugs that Affect Gastric pH

The concomitant use of proton pump inhibitors with Tasigna is not recommended. Nilotinib has pH-dependent solubility, with decreased solubility at higher pH. Drugs such as proton pump inhibitors that inhibit gastric acid secretion to elevate the gastric pH may decrease the solubility of nilotinib and reduce its bioavailability. In

healthy subjects, coadministration of a single 400 mg dose of Tasigna with multiple doses of esomeprazole (a proton-pump inhibitor) at 40 mg daily decreased the nilotinib AUC by 34%. Increasing the dose of Tasigna when coadministered with such agents is not likely to compensate for the loss of exposure. Since proton pump inhibitors affect pH of the upper GI tract for an extended period, separation of doses may not eliminate the interaction.

In healthy subjects, no significant change in nilotinib pharmacokinetics was observed when a single 400 mg dose of Tasigna was administered 10 hours after and 2 hours before famotidine (an H2 blocker). Therefore, when the concurrent use of a H2 blocker is necessary, it may be administered approximately 10 hours before and approximately 2 hours after the dose of Tasigna.

Administration of an antacid (aluminum hydroxide/magnesium hydroxide/simethicone) to healthy subjects, 2 hours before or 2 hours after a single 400 mg dose of Tasigna did not alter nilotinib pharmacokinetics. Therefore, if necessary, an antacid may be administered approximately 2 hours before or approximately 2 hours after the dose of Tasigna.

7.4 Drugs that Inhibit Drug Transport Systems

Nilotinib is a substrate of the efflux transporter P-glycoprotein (P-gp, ABCB1). If Tasigna is administered with drugs that inhibit P-gp, increased concentrations of nilotinib are likely, and caution should be exercised.

7.5 Drugs that May Prolong the QT Interval

The administration of Tasigna with agents that may prolong the QT interval such as anti-arrhythmic medicines should be avoided *[see Boxed Warning, Dosage and Administration (2.4), Warnings and Precautions (5.2, 5.8)]*.

8 USE IN SPECIFIC POPULATIONS

8.1 Pregnancy

Risk Summary

Based on findings from animal studies and the mechanism of action, Tasigna can cause fetal harm when administered to a pregnant woman *[see Clinical Pharmacology (12.1)]*.

There are no available data in pregnant women to inform the drug-associated risk. In animal reproduction studies, administration of nilotinib to pregnant rats and rabbits during organogenesis caused adverse developmental outcomes including embryo-fetal lethality, fetal effects, and fetal variations in rats and rabbits at maternal exposures (AUC) approximately 2 and 0.5 times, respectively, the exposures in patients at the recommended dose (*see Data*). Advise pregnant women of the potential risk to a fetus.

The background risk of major birth defects and miscarriage for the indicated population is unknown. All pregnancies have a background risk of birth defect, loss, or other adverse outcomes. In the U.S. general population, the estimated background risk of major birth defects and miscarriage in clinically recognized pregnancies are 2-4% and 15-20%, respectively.

Data

<u>Animal Data</u>

In embryo-fetal development studies in rats and rabbits, pregnant animals received oral doses of nilotinib up to 100 mg/kg/day and 300 mg/kg/day, respectively, during the period of organogenesis.

In rats, oral administration of nilotinib produced embryo-lethality/fetal effects at doses ≥ 30 mg/kg/day. At ≥ 30 mg/kg/day skeletal variations of incomplete ossification of the frontals and misshapen sternebra were noted, and there was an increased incidence of small renal papilla and fetal edema. At 100 mg/kg/day, nilotinib was associated with maternal toxicity (decreased gestation weight, gravid uterine weight, net weight gain, and food consumption) and resulted in a single incidence of cleft palate and two incidences of pale skin were noted in the fetuses. A single incidence of dilated ureters was noted in a fetus also displaying small renal papilla at 100

mg/kg/day. Additional variations of forepaw and hindpaw phalanx unossified, fused sternebra, bipartite sternebra ossification, and incomplete ossification of the cervical vertebra were noted at 100 mg/kg/day.

In rabbits, oral administration of nilotinib resulted in the early sacrifice of two females, maternal toxicity and increased resorption of fetuses at 300 mg/kg/day. Fetal skeletal variations (incomplete ossification of the hyoid, bent hyoid, supernumerary short detached ribs and the presence of additional ossification sites near the nasals, frontals and in the sternebral column) were also increased at this dose in the presence of maternal toxicity. Slight maternal toxicity was evident at 100 mg/kg/day but there were no reproductive or embryo-fetal effects at this dose.

At 30 mg/kg/day in rats and 300 mg/kg/day in rabbits, the maternal systemic exposure (AUC) were 72700 ng*hr/mL and 17100 ng*hr/mL respectively, representing approximately 2 and 0.5 times the exposure in humans at the highest recommended dose 400 mg twice daily.

When pregnant rats were dosed with nilotinib during organogenesis and through lactation, the adverse effects included a longer gestational period, lower pup body weights until weaning and decreased fertility indices in the pups when they reached maturity, all at a maternal dose of 60 mg/kg (i.e., 360 mg/m^2, approximately 0.7 times the clinical dose of 400 mg twice daily based on body surface area). At doses up to 20 mg/kg (i.e., 120 mg/m^2, approximately 0.25 times the clinical dose of 400 mg twice daily based on body surface area) no adverse effects were seen in the maternal animals or the pups.

8.2 Lactation

Risk Summary

No data are available regarding the presence of nilotinib or its metabolites in human milk or its effects on a breastfed child or on milk production. However, nilotinib is present in the milk of lactating rats. Because of the potential for serious adverse reactions in a nursing child, advise lactating women not to breastfeed during treatment with Tasigna and for at least 14 days after the last dose.

Animal Data

After a single 20 mg/kg of [^{14}C] nilotinib dose to lactating rats, the transfer of parent drug and its metabolites into milk was observed. The overall milk-to-plasma exposure ratio of total radioactivity was approximately 2, based on the $AUC_{0\text{-}24h}$ or $AUC_{0\text{-}\infty}$ values. No rat metabolites of nilotinib were detected that were unique to milk.

8.3 Females and Males of Reproductive Potential

Pregnancy

Based on animal studies, Tasigna can cause fetal harm when administered to a pregnant woman *[see Use in Specific Populations (8.1)]*. Females of reproductive potential should have a pregnancy test prior to starting treatment with Tasigna.

Contraception

Females

Based on animal studies, Tasigna can cause fetal harm when administered to a pregnant woman *[see Use in Specific Populations (8.1)]*. Advise females of reproductive potential to use effective contraception during treatment with Tasigna and for at least 14 days after the last dose.

Infertility

The risk of infertility in females or males of reproductive potential has not been studied in humans. In studies in rats and rabbits, the fertility in males and females was not affected *[see Nonclincal Toxicology (13.1)]*.

8.4 Pediatric Use

The safety and effectiveness of Tasigna in pediatric patients have not been established.

8.5 Geriatric Use

In the clinical trials of Tasigna (patients with newly diagnosed Ph+ CML-CP and resistant or intolerant Ph+ CML-CP and CML-AP), approximately 12% and 30% of patients were 65 years or over respectively.

- Patients with newly diagnosed Ph+ CML-CP: There was no difference in major molecular response between patients aged less than 65 years and those greater than or equal to 65 years.
- Patients with resistant or intolerant CML-CP: There was no difference in major cytogenetic response rate between patients aged less than 65 years and those greater than or equal to 65 years.
- Patients with resistant or intolerant CML-AP: The hematologic response rate was 44% in patients less than 65 years of age and 29% in patients greater than or equal to 65 years.

No major differences for safety were observed in patients greater than or equal to 65 years of age as compared to patients less than 65 years.

8.6 Cardiac Disorders

In the clinical trials, patients with a history of uncontrolled or significant cardiovascular disease, including recent myocardial infarction, congestive heart failure, unstable angina or clinically significant bradycardia, were excluded. Caution should be exercised in patients with relevant cardiac disorders *[see Boxed Warning, Warnings and Precautions (5.2)]*.

8.7 Hepatic Impairment

Nilotinib exposure is increased in patients with impaired hepatic function. In a study of subjects with mild to severe hepatic impairment following a single dose administration of 200 mg of Tasigna, the mean AUC values were increased on average of 35%, 35%, and 56% in subjects with mild (Child-Pugh class A, score 5 to 6), moderate (Child-Pugh class B, score 7 to 9) and severe hepatic impairment (Child-Pugh class C, score 10 to 15), respectively, compared to a control group of subjects with normal hepatic function. Table 9 summarizes the Child-Pugh Liver Function Classification applied in this study. A lower starting dose is recommended in patients with hepatic impairment and the QT interval should be monitored closely in these patients *[see Dosage and Administration (2.4), and Warnings and Precautions (5.10)]*.

Table 9: Child-Pugh Liver Function Classification

Assessment	Degree of Abnormality	Score
Encephalopathy Grade	None<br>1 or 2<br>3 or 4	1<br>2<br>3
Ascites	Absent<br>Slight<br>Moderate	1<br>2<br>3
Total Bilirubin (mg/dL)	<2<br>2–3<br>>3	1<br>2<br>3
Serum Albumin (g/dL)	>3.5<br>2.8–3.5<br><2.8	1<br>2<br>3
Prothrombin Time (seconds prolonged)	<4<br>4–6<br>>6	1<br>2<br>3

10 OVERDOSAGE

Overdose with nilotinib has been reported, where an unspecified number of Tasigna capsules were ingested in combination with alcohol and other drugs. Events included neutropenia, vomiting, and drowsiness. In the event of overdose, the patient should be observed and appropriate supportive treatment given.

11 DESCRIPTION

Tasigna (nilotinib) belongs to a pharmacologic class of drugs known as kinase inhibitors.

Nilotinib drug substance, a monohydrate monohydrochloride, is a white to slightly yellowish to slightly greenish yellow powder with the anhydrous molecular formula and weight, respectively, of $C_{28}H_{22}F_3N_7O$•HCl • H_2O and 584. The solubility of nilotinib in aqueous solutions decreases with increasing pH. Nilotinib is not optically active. The pK_a1 was determined to be 2.1; pK_a2 was estimated to be 5.4.

The chemical name of nilotinib is 4-methyl-N-[3-(4-methyl-1H-imidazol-1-yl)-5-(trifluoromethyl)phenyl]-3-[[4-(3-pyridinyl)-2-pyrimidinyl]amino]-benzamide, monohydrochloride, monohydrate. Its structure is shown below:

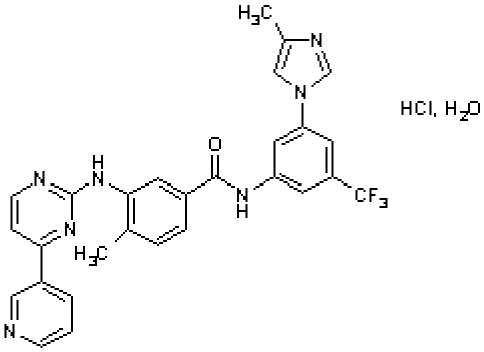

Tasigna (nilotinib) capsules, for oral use, contain 150 mg or 200 mg nilotinib base, anhydrous (as hydrochloride, monohydrate) with the following inactive ingredients: colloidal silicon dioxide, crospovidone, lactose monohydrate, magnesium stearate and poloxamer 188. The capsules contain gelatin, iron oxide (red), iron oxide (yellow), iron oxide (black), and titanium dioxide.

12 CLINICAL PHARMACOLOGY

12.1 Mechanism of Action

Nilotinib is an inhibitor of the BCR-ABL kinase. Nilotinib binds to and stabilizes the inactive conformation of the kinase domain of ABL protein. In vitro, nilotinib inhibited BCR-ABL mediated proliferation of murine leukemic cell lines and human cell lines derived from patients with Ph+ CML. Under the conditions of the assays, nilotinib was able to overcome imatinib resistance resulting from BCR-ABL kinase mutations, in 32 out of 33 mutations tested. In vivo, nilotinib reduced the tumor size in a murine BCR-ABL xenograft model. Nilotinib inhibited the autophosphorylation of the following kinases at IC50 values as indicated: BCR-ABL (20 to 60 nM), PDGFR (69 nM), c-KIT (210 nM), CSF-1R (125 to 250 nM), and DDR1 (3.7 nM).

12.3 Pharmacokinetics

Absorption and Distribution

The absolute bioavailability of nilotinib has not been determined. As compared to an oral drink solution (pH of 1.2 to 1.3), relative bioavailability of nilotinib capsule is approximately 50%. Peak concentrations of nilotinib are reached 3 hours after oral administration.

Steady-state nilotinib exposure was dose-dependent with less than dose-proportional increases in systemic exposure at dose levels higher than 400 mg given as once daily dosing. Daily serum exposure to nilotinib following 400 mg twice daily dosing at steady state was 35% higher than with 800 mg once daily dosing. Steady state exposure (AUC) of nilotinib with 400 mg twice daily dosing was 13% higher than with 300 mg twice daily dosing. The average steady state nilotinib trough and peak concentrations did not change over 12 months. There was no relevant increase in exposure to nilotinib when the dose was increased from 400 mg twice daily to 600 mg twice daily.

The bioavailability of nilotinib was increased when given with a meal. Compared to the fasted state, the systemic exposure (AUC) increased by 82% when the dose was given 30 minutes after a high fat meal.

Single dose administration of two 200 mg nilotinib capsules each dispersed in 1 teaspoon of applesauce and administered within 15 minutes was shown to be bioequivalent to a single dose administration of two 200 mg intact capsules. The blood-to-serum ratio of nilotinib is 0.68. Serum protein binding is approximately 98% on the basis of in vitro experiments.

Median steady-state trough concentration of nilotinib was decreased by 53% in patients with total gastrectomy compared to patients who had not undergone surgeries *[see Warnings and Precautions (5.13)]*.

Pharmacokinetics, Metabolism and Excretion

The apparent elimination half-life estimated from the multiple dose pharmacokinetic studies with daily dosing was approximately 17 hours. Inter-patient variability in nilotinib AUC was 32% to 64%. Steady state conditions were achieved by Day 8. An increase in serum exposure to nilotinib between the first dose and steady state was approximately 2-fold for daily dosing and 3.8-fold for twice daily dosing.

Main metabolic pathways identified in healthy subjects are oxidation and hydroxylation. Nilotinib is the main circulating component in the serum. None of the metabolites contribute significantly to the pharmacological activity of nilotinib.

After a single dose of radiolabeled nilotinib in healthy subjects, more than 90% of the administered dose was eliminated within 7 days: mainly in feces (93% of the dose). Parent drug accounted for 69% of the dose.

Age, body weight, gender, or ethnic origin did not significantly affect the pharmacokinetics of nilotinib.

Renal Impairment

Clinical studies have not been performed in patients with impaired renal function. Clinical studies have excluded patients with serum creatinine concentration greater than 1.5 times the upper limit of the normal range. Since nilotinib and its metabolites are not renally excreted, a decrease in total body clearance is not anticipated in patients with renal impairment.

Drug-Drug Interactions

In a Phase 1 trial of nilotinib 400 mg twice daily in combination with imatinib 400 mg daily or 400 mg twice daily, the AUC increased 30% to 50% for nilotinib and approximately 20% for imatinib.

12.5 Pharmacogenomics

Tasigna can increase bilirubin levels. A pharmacogenetic analysis of 97 patients evaluated the polymorphisms of UGT1A1 and its potential association with hyperbilirubinemia during Tasigna treatment. In this study, the (TA)7/(TA)7 genotype was associated with a statistically significant increase in the risk of hyperbilirubinemia relative to the (TA)6/(TA)6 and (TA)6/(TA)7 genotypes. However, the largest increases in bilirubin were observed in the (TA)7/(TA)7 genotype (UGT1A1*28) patients *[see Warnings and Precautions (5.6)]*.

12.6 QT/QTc Prolongation

In a placebo-controlled study in healthy volunteers designed to assess the effects of Tasigna on the QT interval, administration of Tasigna was associated with concentration-dependent QT prolongation; the maximum mean placebo-adjusted QTcF change from baseline was 18 msec (1-sided 95% Upper CI: 26 msec). A positive control was not included in the QT study of healthy volunteers. Peak plasma concentrations in the QT study were 26% lower than those observed in patients enrolled in the single-arm study *[see Boxed Warning, Warnings and Precautions (5.2), and Adverse Reactions (6.1)]*.

13 NONCLINICAL TOXICOLOGY

13.1 Carcinogenesis, Mutagenesis, Impairment of Fertility

A 2-year carcinogenicity study was conducted orally in rats at nilotinib doses of 5, 15, and 40 mg/kg/day. Exposures in animals at the highest dose tested were approximately 2- to 3-fold the human exposure (based on

AUC) at the nilotinib dose of 400 mg twice daily. The study was negative for carcinogenic findings. A 26-week carcinogenicity study was conducted orally in Tg.rasH2 mice, a model genetically modified to enhance susceptibility to neoplastic transformation, at nilotinib doses of 30, 100, and 300 mg/kg/day. Nilotinib induced in the skin and subcutis statistically significant increases in the incidence of papillomas in females and of papillomas and combined papillomas and carcinomas in males at 300 mg/kg/day. The no-observed-adverse-effect-level (NOAEL) for skin neoplastic lesions was 100 mg/kg/day.

Nilotinib was not mutagenic in a bacterial mutagenesis (Ames) assay, was not clastogenic in a chromosome aberration assay in human lymphocytes, did not induce DNA damage (comet assay) in L5178Y mouse lymphoma cells, nor was it clastogenic in an in vivo rat bone marrow micronucleus assay with two oral treatments at doses up to 2000 mg/kg/dose.

There were no effects on male or female rat and female rabbit mating or fertility at doses up to 180 mg/kg in rats (approximately 4- to 7-fold for males and females, respectively, the AUC in patients at the dose of 400 mg twice daily) or 300 mg/kg in rabbits (approximately one-half the AUC in patients at the dose of 400 mg twice daily). The effect of Tasigna on human fertility is unknown. In a study where male and female rats were treated with nilotinib at oral doses of 20 to 180 mg/kg/day (approximately 1- to 6.6-fold the AUC in patients at the dose of 400 mg twice daily) during the pre-mating and mating periods and then mated, and dosing of pregnant rats continued through gestation Day 6, nilotinib increased post-implantation loss and early resorption, and decreased the number of viable fetuses and litter size at all doses tested.

14 CLINICAL STUDIES

14.1 Newly Diagnosed Ph+ CML-CP

The ENESTnd (Evaluating Nilotinib Efficacy and Safety in clinical Trials-Newly Diagnosed patients) study (NCT00471497) was an open-label, multicenter, randomized trial conducted to determine the efficacy of Tasigna versus imatinib tablets in adult patients with cytogenetically confirmed newly diagnosed Ph+ CML-CP. Patients were within 6 months of diagnosis and were previously untreated for CML-CP, except for hydroxyurea and/or anagrelide. Efficacy was based on a total of 846 patients: 283 patients in the imatinib 400 mg once daily group, 282 patients in the nilotinib 300 mg twice daily group, 281 patients in the nilotinib 400 mg twice daily group.

Median age was 46 years in the imatinib group and 47 years in both nilotinib groups, with 12%, 13%, and 10% of patients greater than or equal to 65 years of age in imatinib 400 mg once daily, nilotinib 300 mg twice daily and nilotinib 400 mg twice daily treatment groups, respectively. There were slightly more male than female patients in all groups (56%, 56%, and 62% in imatinib 400 mg once daily, nilotinib 300 mg twice daily and nilotinib 400 mg twice daily treatment groups, respectively). More than 60% of all patients were Caucasian, and 25% were Asian.

The primary data analysis was performed when all 846 patients completed 12 months of treatment (or discontinued earlier). Subsequent analyses were done when patients completed 24, 36, 48, and 60 months of treatment (or discontinued earlier). The median time on treatment was approximately 61 months in all three treatment groups.

The primary efficacy endpoint was major molecular response (MMR) at 12 months after the start of study medication. MMR was defined as less than or equal to 0.1% BCR-ABL/ABL % by international scale measured by RQ-PCR, which corresponds to a greater than or equal to 3 log reduction of BCR-ABL transcript from standardized baseline. Efficacy endpoints are summarized in Table 10.

Two patients in the nilotinib arm progressed to either accelerated phase or blast crisis (both within the first 6 months of treatment) while 12 patients on the imatinib arm progressed to either accelerated phase or blast crisis (7 patients within first 6 months, 2 patients within 6 to 12 months, 2 patients within 12 to 18 months and 1 patient within 18 to 24 months).

Table 10: Efficacy (MMR and CCyR) of TASIGNA Compared to Imatinib in Newly Diagnosed Ph+ CML-CP (ENESTnd)

	TASIGNA 300 mg twice daily	Imatinib 400 mg once daily
	N=282	N=283
MMR at 12 months (95% CI)	44% (38.4, 50.3)	22% (17.6, 27.6)
P-Value[a]	<0.0001	
CCyR[b] by 12 months (95% CI)	80% (75.0, 84.6)	65% (59.2, 70.6)
MMR at 24 months (95% CI)	62% (55.8, 67.4)	38% (31.8, 43.4)
CCyR[b] by 24 months (95% CI)	87% (82.4, 90.6)	77% (71.7, 81.8)

[a]CMH test stratified by Sokal risk group
[b]CCyR: 0% Ph+ metaphases. Cytogenetic responses were based on the percentage of Ph+ metaphases among greater than or equal to 20 metaphase cells in each bone marrow sample.

By the 60 months, MMR was achieved by 77% of patients on Tasigna and 60% of patients on imatinib; MR4.5 was achieved by 53.5% of patients on Tasigna and 31.4% on imatinib. Median overall survival was not reached in either arm. At the time of the 60-month final analysis, the estimated survival rate was 93.7% for patients on Tasigna and 91.7% for patients on imatinib.

14.2 Patients with Resistant or Intolerant Ph+ CML-CP and CML-AP

Study CAMN107A2101 (referred to as Study A2101) (NCT00109707) was a single-arm, open-label, multicenter study conducted to evaluate the efficacy and safety of Tasigna (400 mg twice daily) in patients with imatinib-resistant or -intolerant CML with separate cohorts for chronic and accelerated phase disease. The definition of imatinib resistance included failure to achieve a complete hematologic response (by 3 months), cytogenetic response (by 6 months) or major cytogenetic response (by 12 months) or progression of disease after a previous cytogenetic or hematologic response. Imatinib intolerance was defined as discontinuation of treatment due to toxicity and lack of a major cytogenetic response at time of study entry. At the time of data cut-off, 321 patients with CML-CP and 137 patients with CML-AP with a minimum follow-up of 24 months were enrolled. In this study, about 50% of CML-CP and CML-AP patients were males, over 90% (CML-CP) and 80% (CML-AP) were Caucasian, and approximately 30% were age 65 years or older.

Overall, 73% of patients were imatinib resistant while 27% were imatinib intolerant. The median time of prior imatinib treatment was approximately 32 (CML-CP) and 28 (CML-AP) months. Prior therapy included hydroxyurea in 85% of patients, interferon in 56% and stem cell or bone marrow transplant in 8%. The median highest prior imatinib dose was 600 mg per day for patients with CML-CP and CML-AP, and the highest prior imatinib dose was greater than or equal to 600 mg/day in 74% of all patients with 40% of patients receiving imatinib doses greater than or equal to 800 mg/day.

Median duration of nilotinib treatment was 18.4 months in patients with CML-CP and 8.7 months in patients with CML-AP.

The efficacy endpoint in CML-CP was unconfirmed major cytogenetic response (MCyR) which included complete and partial cytogenetic responses.

The efficacy endpoint in CML-AP was confirmed hematologic response (HR), defined as either a complete hematologic response (CHR) or no evidence of leukemia (NEL). The rates of response for CML-CP and CML-AP patients are reported in Table 11.

Median durations of response had not been reached at the time of data analysis.

Table 11: Efficacy of TASIGNA in Resistant or Intolerant Ph+ CML-CP and CML-AP (Study A2101)

**Cytogenetic Response Rate (Unconfirmed) (%)[a]**	
	**Chronic Phase (n=321)**
Major (95% CI)	51% (46%–57%)
Complete (95% CI)	37% (32%–42%)
Partial (95% CI)	15% (11%–19%)
	**Accelerated Phase (n=137)**
**Hematologic Response Rate (Confirmed) (95% CI)[b]**	39% (31%–48%)
Complete Hematologic Response Rate (95% CI)	30% (22%–38%)
No Evidence of Leukemia (95% CI)	9% (5%–16%)

[a]Cytogenetic response criteria: Complete (0% Ph + metaphases) or partial (1% to 35%). Cytogenetic responses were based on the percentage of Ph-positive metaphases among greater than or equal to 20 metaphase cells in each bone marrow sample.
[b]Hematologic response=CHR + NEL (all responses confirmed after 4 weeks).

CHR (CML-CP): WBC less than 10 x 10^9/L, platelets less than 450,000/mm^3, no blasts or promyelocytes in peripheral blood, less than 5% myelocytes + metamyelocytes in bone marrow, less than 20% basophils in peripheral blood, and no extramedullary involvement.

CHR (CML-AP): neutrophils greater than or equal to 1.5 x 10^9/L, platelets greater than or equal to 100 x 10^9/L, no myeloblasts in peripheral blood, myeloblasts less than 5% in bone marrow, and no extramedullary involvement.

NEL: same criteria as for CHR but neutrophils greater than or equal to 1.0 x 10^9/L and platelets greater than or equal to 20 x 10^9/L without transfusions or bleeding.

Patients with Chronic Phase

The MCyR rate in 321 CML-CP patients was 51%. The median time to MCyR among responders was 2.8 months (range 1 to 28 months). The median duration of MCyR cannot be estimated. The median duration of exposure on this single arm-trial was 18.4 months. Among the CML-CP patients who achieved MCyR, 62% of them had MCyR lasting more than 18 months. The CCyR rate was 37%.

Patients with Accelerated Phase

The overall confirmed hematologic response rate in 137 patients with CML-AP was 39%. The median time to first hematologic response among responders was 1 month (range 1 to 14 months). Among the CML-AP patients who achieved HR, 44% of them had a response lasting for more than 18 months.

After imatinib failure, 24 different BCR-ABL mutations were noted in 42% of chronic phase and 54% of accelerated phase CML patients who were evaluated for mutations.

14.3 Treatment discontinuation in newly diagnosed Ph+ CML-CP patients who have achieved a sustained molecular response (MR4.5)

The ENESTfreedom (Evaluating Nilotinib Efficacy and Safety in clinical Trials-freedom) study (NCT01784068) is an open-label, multicenter, single-arm study, where 215 adult patients with Ph+ CML-CP treated with Tasigna in first-line for ≥ 2 years who achieved MR4.5 as measured with the MolecularMD MRDx™ BCR-ABL Test were enrolled to continue Tasigna treatment for an additional 52 weeks (Tasigna consolidation phase).

Of the 215 patients, 190 patients (88.4%) entered the “Treatment-free Remission” (TFR) phase after achieving a sustained molecular response (MR4.5) during the consolidation phase, defined by the following criteria:

- The 4 last quarterly assessments (taken every 12 weeks) were at least MR4 (BCR-ABL/ABL ≤ 0.01% IS), and maintained for 1 year
- The last assessment being MR4.5 (BCR-ABL/ABL ≤ 0.0032% IS)
- No more than two assessments falling between MR4 and MR4.5 (0.0032% IS < BCR-ABL/ABL ≤ 0.01% IS).

The median age of patients who entered the TFR phase was 55 years, 49.5% were females, and 21.1% of the patients were ≥ 65 years of age. BCR-ABL levels were monitored every 4 weeks during the first 48 weeks of the TFR phase. Monitoring frequency was intensified to every 2 weeks upon the loss of MR4.0. Biweekly monitoring ended at one of the following time points:

- Loss of MMR requiring patient to re-initiate Tasigna treatment
- When the BCR-ABL levels returned to a range between MR4.0 and MR4.5
- When the BCR-ABL levels remained lower than MMR for 4 consecutive measurements (8 weeks from initial loss of MR4.0).

Any patient with loss of MMR during the TFR phase re-initiated Tasigna treatment at 300 mg twice daily or at a reduced dose level of 400 mg once daily if required from the perspective of tolerance, within 5 weeks after the collection date of the blood sample demonstrating loss of MMR. Patients who required re-initiation of Tasigna treatment were monitored for BCR-ABL levels every 4 weeks for the first 24 weeks and then every 12 weeks thereafter in patients who regained MMR.

Efficacy was based on the 96-week analysis data cut-off date, by which time, 91 patients (47.9%) discontinued from the TFR phase due to loss of MMR, and 1 (0.5%), 1 (0.5%), 1 (0.5%) and 3 patients (1.6%) due to death from unknown cause, physician decision, lost to follow-up and subject decision, respectively. Among the 91 patients who discontinued the TFR phase due to loss of MMR, 88 patients restarted Tasigna treatment and 3 patients permanently discontinued from the study.

By the 96-week data cut-off, of the 88 patients who restarted treatment due to loss of MMR in the TFR phase, 87 patients (98.9%) patients regained MMR (one patient discontinued study permanently due to subject decision after 7.1 weeks of retreatment without regaining MMR) and 81 patients (92.0%) regained MR4.5 by the time of the cut-off date. The cumulative rate of MMR and MR4.5 regained at 24 weeks since treatment re initiation was 97.7% (86/88 patients) and 86.4% (76/88 patients), respectively.

Table 12: Efficacy Results for ENESTfreedom

Patients Who Entered the Treatment Free Remission (TFR) Phase (Full Analysis Set, N=190)			
	Patients in TFR phase[1] at the specified time point		Loss of MMR[2] by the specified time point
	%	95% CI	%
24 weeks	62.1	(54.8, 69.0)	35.8
48 weeks	51.6	(44.2, 58.9)	45.8
96 weeks	48.9	(41.6, 56.3)	47.9

[1]Patients in MMR at the specified time point in the TFR phase

[2]Based on the time to event (loss of MMR) data during the TFR phase

Among the 190 patients in the TFR phase, 98 patients had a treatment-free survival (TFS) event (defined as discontinuation from TFR phase due to any reason, loss of MMR, death due to any cause, progression to AP/BC up to the end of TFR phase, or re-initiation of treatment due to any cause in the study) by the 96-week cut-off date.

Figure 14-1 Kaplan-Meier estimate of treatment-free survival after start of TFR (Full Analysis Set ENESTfreedom)

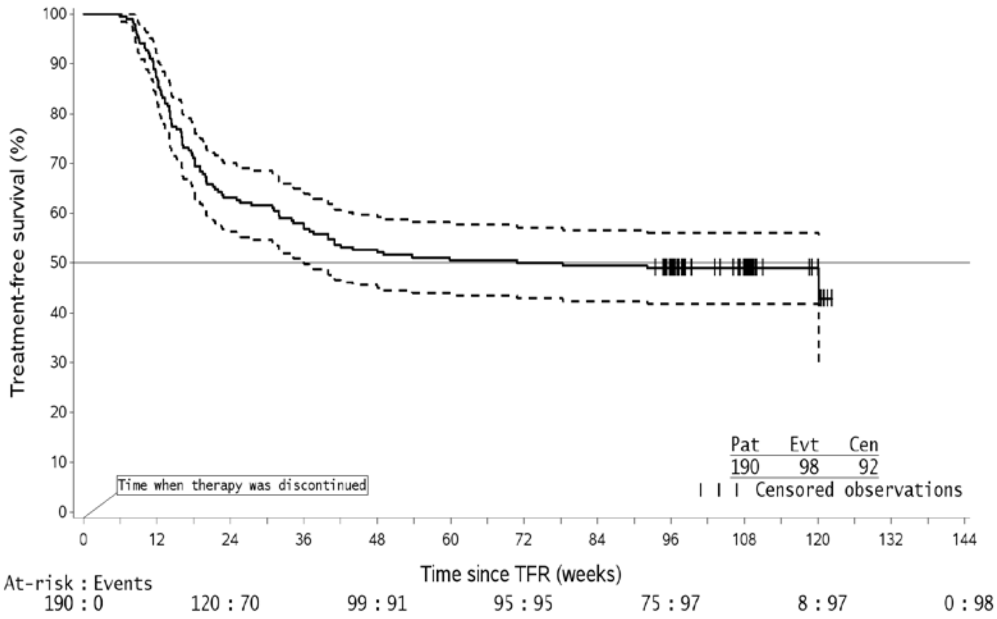

1. For a given time point, the points on the dashed curves represent the 95% confidence limits for the associated KM estimate on the solid curve.
2. By the time of the 96-week data cut-off date, one single patient lost MMR at week 120, at the time when only 8 patients were considered at risk. This explains the artificial drop at the end of the curve.

14.4 Treatment discontinuation in Ph+ CML-CP patients who have achieved a sustained molecular response (MR4.5) on Tasigna following prior imatinib therapy

The ENESTop (Evaluating Nilotinib Efficacy and Safety in clinical Trials-STop) study (NCT01698905) is an open-label, multicenter, single-arm study, where 163 adult patients with Ph+ CML-CP taking tyrosine kinase inhibitors (TKIs) for ≥ 3 years (imatinib as initial TKI therapy for more than 4 weeks without documented MR4.5 on imatinib at the time of switch to Tasigna, then switched to Tasigna for at least 2 years), and who achieved MR4.5 on Tasigna treatment as measured with the MolecularMD MRDx™ BCR-ABL Test were enrolled to continue Tasigna treatment for an additional 52 weeks (Tasigna consolidation phase). Of the 163 patients, 126 patients (77.3%) entered the TFR phase after achieving a sustained molecular response (MR4.5) during the consolidation phase, defined by the following criterion:

- The 4 last quarterly assessments (taken every 12 weeks) showed no confirmed loss of MR4.5 (BCR-ABL/ABL ≤ 0.0032% IS) during 1 year.

The median age of patients who entered the TFR phase was 56 years, 55.6% were females, and 27.8% of the patients were ≥ 65 years of age. The median actual dose intensity during the 52-week Tasigna consolidation phase was 771.8 mg/day with 52.4%, 29.4%, 0.8%, 16.7% and 0.8% of patients receiving a daily Tasigna dose of 800 mg, 600 mg, 450mg, 400mg and 300mg just before entry into the TFR phase, respectively.

Patients who entered the TFR phase but experienced two consecutive measurements of BCR-ABL/ABL > 0.01% IS were considered having a confirmed loss of MR4.0, triggering re-initiation of Tasigna treatment. Patients with loss of MMR in the TFR phase immediately restarted Tasigna treatment without confirmation. All patients who restarted Tasigna therapy had BCR-ABL transcript levels monitored every 4 weeks for the first 24 weeks, then once every 12 weeks.

Efficacy was based on the 96-week analysis data cut-off date, by which time, 61 patients (48.4%) had discontinued from the TFR phase: 58 patients (46.0%) due to loss of MMR or confirm loss of MR4.0, 2 patients (1.6%) due to subject/guardian decision and one patient (0.8%) due to pregnancy. Among the 58 patients who

discontinued from the TFR phase due to confirmed loss of MR4.0 or loss of MMR, 56 patients restarted Tasigna therapy and 2 patients permanently discontinued from the study.

By the 96-week data cut-off, of the 56 patients who restarted Tasigna treatment due to confirmed loss of MR4.0 or loss of MMR in the TFR phase, 52 patients (92.9%) regained MR4.0 and MR4.5; 4 patients (7.1%) did not regain MR4.0 by the time of the cut-off date. The cumulative rate of MR4 and MR4.5 regained by 48-weeks since treatment re-initiation, was 92.9% (52/56 patients) and 91.1% (51/56 patients), respectively.

Table 13: Efficacy Results for ENESTop

Patients Who Entered the Treatment Free Remission (TFR) Phase (Full Analysis Set, N=126)			
	Patients in TFR phase[1] at the specified time point		Loss of MMR or confirmed loss of MR4[2] by the specified time point
	%	95% CI	%
24 weeks	60.3	(51.2, 68.9)	38.9
48 weeks	57.9	(48.8, 66.7)	41.3
96 weeks	53.2	(44.1, 62.1)	43.7

[1]Patients without loss of MMR or confirmed loss of MR4 by specified time point of TFR phase

[2]Based on the time to event (loss of MMR or confirmed loss of MR4) data during the TFR phase

Among the 126 patients in the TFR phase, 61 patients (48.4%) had a treatment-free survival (TFS) event (defined as discontinuation from TFR phase due to any reason, loss of MMR, confirmed loss of MR4, death due to any cause, progression to AP/BC up to the end of TFR phase, or re-initiation of treatment due to any cause in the study) on or before the 96-month cut-off date.

Figure 14-2 Kaplan-Meier estimate of treatment-free survival after start of TFR (Full Analysis Set ENESTop)

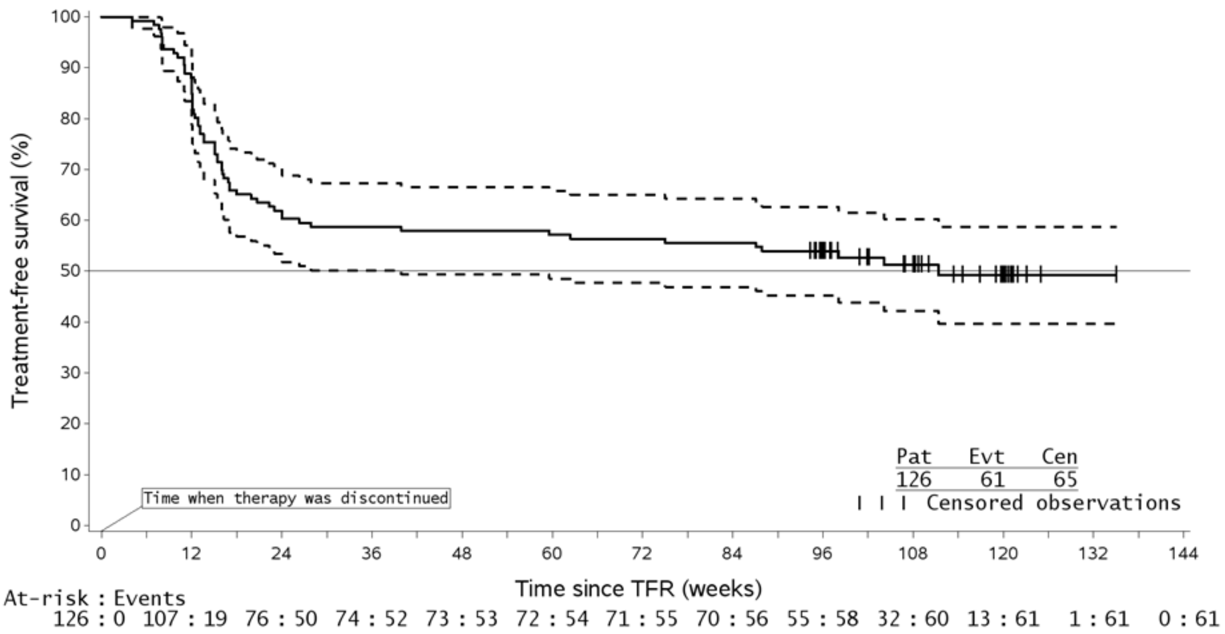

1. For a given time point, the points on the dashed curves represent the 95% confidence limits for the associated KM estimate on the solid curve.

16 HOW SUPPLIED/STORAGE AND HANDLING

Tasigna (nilotinib) 150 mg capsules are red opaque hard gelatin capsules, size 1 with black axial imprint "NVR/BCR." Tasigna (nilotinib) 200 mg capsules are light yellow opaque hard gelatin capsules, size 0 with the red axial imprint "NVR/TKI." Tasigna capsules are supplied in blister packs.

150 mg
Carton of 4 blister packs of (4x28) ....NDC 0078-0592-87
Blisters of 28 capsules ....NDC 0078-0592-51

200 mg
Carton of 4 blister packs of (4x28) ....NDC 0078-0526-87
Blisters of 28 capsules ....NDC 0078-0526-51

Tasigna (nilotinib) capsules should be stored at 25°C (77°F); excursions permitted between 15°C to 30°C (59°F to 86°F) [see USP Controlled Room Temperature].

17 PATIENT COUNSELING INFORMATION

Advise the patient to read the FDA-approved patient labeling (Medication Guide).

A Medication Guide is required for distribution with Tasigna. The complete text of the Medication Guide is reprinted at the end of this document.

Myelosuppression

Advise patients that treatment with Tasigna can cause serious thrombocytopenia, neutropenia, and anemia. Advise patients to seek immediate medical attention if symptoms suggestive of low blood counts occur, such as fever, chills or other signs of infection, unexplained bleeding or bruising, or unexplained weakness or shortness of breath *[see Warnings and Precautions (5.1)]*.

QT Prolongation

Advise patients that Tasigna can cause possibly life-threatening, abnormal heart beat. Advise patients to seek immediate medical attention if symptoms of abnormal heart beat occur, such as feeling light-headed, faint or experiencing an irregular heartbeat *[see Warnings and Precautions (5.2)]*.

Cardiac and Arterial Vascular Occlusive Events

Advise patients that cardiovascular events (including ischemic heart disease, peripheral arterial occlusive disease, and ischemic cerebrovascular events) have been reported. Advise patients to seek immediate medical attention if any symptoms suggestive of a cardiovascular event occur, such as chest or leg pain, numbness or weakness, or problems walking or speaking occur suddenly *[see Warnings and Precautions (5.4)]*.

Pancreatitis and Elevated Serum Lipase

Advise patients that Tasigna can increase the risk of pancreatitis and that patients with a previous history of pancreatitis may be at greater risk. Advise patients to seek immediate medical attention if symptoms suggestive of pancreatitis occur, such as sudden stomach area pain with accompanying nausea and vomiting *[see Warnings and Precautions (5.5)]*.

Taking Tasigna

Advise patients to take Tasigna doses twice daily approximately 12 hours apart. The capsules should be swallowed whole with water.

Advise patients to take Tasigna on an empty stomach. No food should be consumed for at least 2 hours before the dose is taken and for at least 1 hour after the dose is taken. Patients should not consume grapefruit products and other foods that are known to inhibit CYP3A4 at any time during Tasigna treatment *[see Dosage and Administration (2.4), Warnings and Precautions (5.8, 5.9) and Medication Guide]*.

If the patient missed a dose of Tasigna, the patient should take the next scheduled dose at its regular time. The patient should not take two doses at the same time.

Should patients be unable to swallow capsules, the contents of each capsule may be dispersed in one teaspoon of applesauce and the mixture swallowed immediately (within 15 minutes).

Tumor Lysis Syndrome

Advise patients that Tasigna can cause tumor lysis syndrome and to seek immediate medical attention if any symptoms suggestive of tumor lysis syndrome occur such as an abnormal heartbeat or less urine production *[see Warnings and Precautions (5.11)]*.

Hemorrhage

Advise patients that serious hemorrhagic events, including fatal events, have occurred in patients with CML treated with Tasigna. Advise patients to seek immediate medical attention if symptoms suggestive of hemorrhage occur, such as uncontrolled bleeding, changes in eyesight, unconsciousness, or sudden headache or sudden confusion in surroundings *[see Warnings and Precautions (5.12)]*.

Fluid Retention

Advise patients that Tasigna can cause fluid retention and to seek immediate medical attention if any symptoms suggestive of fluid retention such as shortness of breath, rapid weight gain, or swelling occur *[see Warnings and Precautions (5.16)]*.

Treatment Free Remission (TFR)

Advise patients that frequent monitoring is required to detect possible loss of remission if TFR is attempted. Advise patients that musculoskeletal symptoms such as muscle pain, pain in extremity, joint pain, bone pain, or spinal pain, may occur more frequently than before treatment discontinuation *[see Warnings and Precautions (5.18) and Medication Guide]*.

Drug Interactions

Advise patients that Tasigna and certain other medicines, including over the counter medications or herbal supplements (such as St. John's Wort), can interact with each other *[see Warnings and Precautions (5.8) and Drug Interactions (7)]*.

Embryo-Fetal Toxicity

Advise females to inform their healthcare provider if they are pregnant or become pregnant. Inform female patients of the risk to a fetus and potential loss of the pregnancy *[see Use in Specific Populations (8.1)]*.

Advise females of reproductive potential to use effective contraception during treatment and for at least 14 days after receiving the last dose of Tasigna *[see Warnings and Precautions (5.17) and Use in Specific Populations (8.1, 8.3)]*.

Lactation

Advise lactating women not to breastfeed during treatment with Tasigna and for at least 14 days after the last dose *[see Use in Specific Populations (8.2)]*.

Compliance

Advise patients of the following:

- Continue taking Tasigna every day for as long as their doctor tells them.
- This is a long-term treatment.
- Do not change dose or stop taking Tasigna without first consulting their doctor.
- If a dose is missed, take the next dose as scheduled. Do not take a double dose to make up for the missed capsules.

Medication Guide
TASIGNA® (ta-sig-na)
(nilotinib)
capsules

What is the most important information I should know about Tasigna?

Tasigna can cause a possible life-threatening heart problem called QTc prolongation. QTc prolongation causes an irregular heartbeat, which may lead to sudden death.

Your healthcare provider should check the electrical activity of your heart with a test called an electrocardiogram (ECG):

- before starting Tasigna
- 7 days after starting Tasigna
- with any dose changes
- regularly during Tasigna treatment

You may lower your chances for having QTc prolongation with Tasigna if you:

- **Take Tasigna on an empty stomach:**
  - Avoid eating food for at least 2 hours before the dose is taken, and
  - Avoid eating food for at least 1 hour after the dose is taken.
- Avoid grapefruit, grapefruit juice, and any supplement containing grapefruit extract while taking Tasigna. Food and grapefruit products increase the amount of Tasigna in your body.
- Avoid taking other medicines or supplements with Tasigna that can also cause QTc prolongation.
- Tasigna can interact with many medicines and supplements and increase your chance for serious and life-threatening side effects.
- Do not take any other medicine while taking Tasigna unless your healthcare provider tells you it is okay to do so.
- If you cannot swallow Tasigna capsules whole, you may open the Tasigna capsule and sprinkle the contents of each capsule in 1 teaspoon of applesauce (puréed apple). Swallow the mixture right away (within 15 minutes). For more information, see "How should I take Tasigna?"

Call your healthcare provider right away if you feel lightheaded, faint, or have an irregular heartbeat while taking Tasigna. These can be symptoms of QTc prolongation.

What is Tasigna?

Tasigna is a prescription medicine used to treat a type of leukemia called Philadelphia chromosome positive chronic myeloid leukemia (Ph+ CML) in adults who:

- are newly diagnosed, **or**
- are no longer benefiting from previous other treatments, including treatment with imatinib (Gleevec), **or**
- have taken other treatments, including imatinib (Gleevec), and cannot tolerate them

It is not known if Tasigna is safe and effective in children.

Who should not take Tasigna?

Do not take if you have:

- low levels of potassium or magnesium in your blood
- long QTc syndrome

Before taking Tasigna, tell your healthcare provider about all of your medical conditions, including if you :

- have heart problems
- have had a stroke or other problems due to decreased blood flow to the brain
- have problems with decreased blood flow to your legs
- have irregular heartbeat
- have QTc prolongation or a family history of it
- have liver problems
- have had pancreatitis
- have low blood levels of potassium or magnesium in your blood
- have a severe problem with lactose (milk sugar) or other sugars. Tasigna capsules contain lactose. Most people who have mild or moderate lactose intolerance can take Tasigna.
- have bleeding problems
- had a surgical procedure involving the removal of the entire stomach (total gastrectomy)
- are pregnant or plan to become pregnant. Tasigna can harm your unborn baby. Tell your healthcare provider right away if you are pregnant, or if you become pregnant during treatment with Tasigna.

<u>In females who are able to become pregnant:</u>

- o Your healthcare provider should do a pregnancy test before you start treatment with Tasigna.
- o Use effective birth control (contraception) during treatment with Tasigna and for at least 14 days after the last dose.

- are breastfeeding or plan to breastfeed. It is not known if Tasigna passes into your breast milk. Do not breastfeed during treatment and for at least 14 days after your last dose of Tasigna**.**

Tell your healthcare provider about all the medicines you take, including prescription and over-the-counter medicines, vitamins and herbal supplements.

If you need to take antacids (medicines to treat heartburn) do not take them at the same time that you take Tasigna. If you take:

- **a medicine to block the amount of acid produced in the stomach (H2 blocker):** Take these medicines **about 10 hours before** you take Tasigna, **or about 2 hours after** you take Tasigna.
- **an antacid that contains aluminum hydroxide, magnesium hydroxide, and simethicone to reduce the amount of acid in the stomach:** Take these medicines **about 2 hours before or about 2 hours after** you take Tasigna.

Tasigna can interact with many medicines and supplements and increase your chance for serious and life-threatening side effects. **See "What is the most important information I should know about Tasigna?"**

How should I take Tasigna?

- Take Tasigna exactly as your healthcare provider tells you to take it. Do not change your dose or stop taking Tasigna unless your healthcare provider tells you.
- Tasigna is a long-term treatment.
- Your healthcare provider will tell you how many Tasigna capsules to take and when to take them.
- **Tasigna must be taken on an empty stomach.**
  - o **Avoid eating food for at least 2 hours before the dose is taken, and**
  - o **Avoid eating food for at least 1 hour after the dose is taken.**
- Swallow Tasigna capsules whole with water. If you cannot swallow Tasigna capsules whole, tell your healthcare provider.
- **If you cannot swallow Tasigna capsules whole:**
  - o **Open the Tasigna capsules and sprinkle the contents in 1 teaspoon of applesauce (puréed apple).**
    - ▪ **Do not use more than 1 teaspoon of applesauce.**
    - ▪ **Only use applesauce. Do not sprinkle Tasigna onto other foods.**
  - o **Swallow the mixture right away (within 15 minutes).**
- Do not drink grapefruit juice, eat grapefruit, or take supplements containing grapefruit extract at any time during treatment. **See "What is the most important information I should know about Tasigna?"**
- If you miss a dose, just take your next dose at your regular time. Do not take 2 doses at the same time to make up for a missed dose.
- If you take too much Tasigna, call your healthcare provider or poison control center right away. Symptoms may include vomiting and drowsiness.
- During treatment with Tasigna your healthcare provider will do tests to check for side effects and to see how well Tasigna is working for you. The tests will check your:
  - o heart
  - o blood cells (white blood cells, red blood cells, and platelets). Your blood cells should be checked every 2 weeks for the first 2 months and then monthly.
  - o electrolytes (potassium, magnesium)
  - o pancreas and liver function
  - o bone marrow samples

Your healthcare provider may change your dose. Your healthcare provider may have you stop Tasigna for some time or lower your dose if you have side effects with it.

- Your healthcare provider will monitor your CML during treatment with Tasigna to see if you are in a remission. After at least 3 years of treatment with Tasigna, your healthcare provider may do certain tests to determine if you continue to be in remission. Based on your test results, your healthcare provider may decide if you may be eligible to try stopping treatment with Tasigna. This is called Treatment Free Remission (TFR).
- Your healthcare provider will carefully monitor your CML during and after you stop taking Tasigna. Based you your test results, your healthcare provider may need to re-start your Tasigna if your CML is no longer in remission.
- It is important that you are followed by your healthcare provider and undergo frequent monitoring to find out if you

need to re-start your Tasigna treatment because you are no longer in TFR. Follow your healthcare provider's instructions about re-starting Tasigna if you are no longer in TFR.

What are the possible side effects of Tasigna?

Tasigna may cause serious side effects, including:

- **See "What is the most important information I should know about Tasigna?"**
- **Low blood cell counts.** Low blood cell counts (red blood cells, white blood cells, and platelets) are common with Tasigna, but can also be severe. Your healthcare provider will check your blood counts regularly during treatment with Tasigna. Call your healthcare provide or get medical help right away if you develop any signs or symptoms of low blood counts including:
    - fever
    - chills or other signs of infection
    - unexplained bleeding or bruising
    - unexplained weakness
    - shortness of breath
- **Decreased blood flow to the leg, heart, or brain.** People who have recently been diagnosed with Ph+ CML and take Tasigna may develop decreased blood flow to the leg, the heart, or brain.

  Get medical help right away if you suddenly develop any of the following symptoms:
    - chest pain or discomfort
    - numbness or weakness
    - problems walking or speaking
    - leg pain
    - your leg feels cold
    - change in the skin color of your leg
- **Pancreas inflammation (pancreatitis).** Tell your healthcare provider right away if you develop any symptoms of pancreatitis including sudden stomach area pain with nausea and vomiting.
- **Liver problems.** Symptoms include yellow skin and eyes.
- **Tumor Lysis Syndrome (TLS).** TLS is caused by a fast breakdown of cancer cells. TLS can cause you to have:
    - **kidney failure and the need for dialysis treatment**
    - **an abnormal heart beat**

  Your healthcare provider may do blood tests to check you for TLS.
- **Bleeding problems.** Serious bleeding problems and death have happened during treatment with Tasigna. Tell your healthcare provider right away if you develop any signs and symptoms of bleeding during treatment with Tasigna.
- **Fluid retention.** Your body may hold too much fluid (fluid retention). Symptoms of fluid retention include shortness of breath, rapid weight gain, and swelling.

The most common side effects of Tasigna include:

- nausea
- rash
- headache
- tiredness
- itching
- vomiting
- diarrhea
- cough
- constipation
- muscle and joint pain
- runny or stuffy nose, sneezing, sore throat
- fever
- night sweats

If you and your healthcare provider decide that you can stop taking Tasigna and try treatment free remission (TFR), you may have more muscle and bone (musculoskeletal) symptoms than before you stopped treatment. Symptoms may include:

- muscle pain
- arm and leg pain
- joint pain
- bone pain
- spine pain

These are not all of the possible side effects of Tasigna.

Call your doctor for medical advice about side effects. You may report side effects to FDA at 1-800-FDA-1088.

How should I store Tasigna?

- Store Tasigna at room temperature between 68°F to 77°F (20°C to 25°C).
- Safely throw away medicine that is out of date or no longer needed.

Keep Tasigna and all medicines out of the reach of children.

General information about the safe and effective use of Tasigna.

Medicines are sometimes prescribed for purposes other than those listed in a Medication Guide. Do not use Tasigna for a condition for which it was not prescribed. Do not give Tasigna to other people, even if they have the same symptoms that you have. It may harm them. You can ask your pharmacist or healthcare provider for information about Tasigna that is written for health professionals.

What are the ingredients in Tasigna?

Active ingredient: nilotinib

Inactive ingredients: colloidal silicon dioxide, crospovidone, lactose monohydrate, magnesium stearate and poloxamer 188.

The capsule shell contains gelatin, iron oxide (red), iron oxide (yellow), iron oxide (black), and titanium dioxide.

Distributed by: Novartis Pharmaceuticals Corporation, East Hanover, New Jersey 07936

© Novartis

For more information, go to www.Tasigna.com or call 1-866-411-8274.

This Medication Guide has been approved by the U.S. Food and Drug Administration. Revised: December 2017

T2017-126

APPENDIX 2: EXCLUSIONARY AND PRECAUTIONARY MEDICATIONS

NOTE: This list is not exhaustive but is designed to give examples of more commonly used drug classes.

Table 1. Exclusionary Medications: Known risk to prolong the QT interval

**Antimicrobials**	**Antipsychotics (all have some risk)**
Erythromycin	Fluphenazine
Azithromycin (systemic)	Haloperidol
Levofloxacin (systemic)	Pimozide
Sparfloxacin	Chlorpromazine
Ciprofloxacin (systemic)	Droperidol
Clarithromycin	Aripiprazole
Telithromycin	Thioridazine
Moxifloxacin	Sulpiride
Fluconazole (systemic)	Sertindole
**Antiprotozoal, Antimalarials, Antituberculosis**	
Pentamidine	**Antidepressant**
Mefloquine	Citalopram/escitalopram*
Chloroquine	Trimipramine
Quinine	Clomipramine
Delamanid	Dosulepin
Bedaquiline	Mirtazapine
Mesoridazine	Trazodone
**Antiarrhythmics**	Venlafaxine
Dronedarone	Lofepramine
Sotalol	**Antineoplastics**
Dofetilide	Arsenic trioxide
Ibutilide	Ceritinib
Quinidine	Dabrafenib
Amiodarone	Dasatinib
Disopyramide	Pazopanib
**Antiplatelet**	Bosutinib
Cilostazol	Panobinostat
**Antiemetics**	Lapatinib
Domperidone	Crizotinib
Cisapride	Bortezomib
Granisetron	Oxaliplatin
Droperidol	Ribociclib
Ondansetron//Granisetron	Vandetanib
Propafenone	Osimertinib
Procainamide	Pazopanib
Flecainide	Romidepsin
**Others**	Sunitinib
Methadone	Tamoxifen

Sevoflurane	Toremifene
Anagrelide	Vemurafenib
Levomethadyl	
Vilanterol	
Propofol	**Hepatitis C Antivirals**
Probucol	Telaprevir
Halofantrine	Boceprevir
Tacrolimus	**Anticholinergic**
Nicardipine	Solifenacin

***stable regimen of** Citalopram (≤20mg) or escitalopram (≤10mg) will be allowed provided that the baseline EKG did not demonstrate QT prolongation

Table 2. Exclusionary Medications: Strong CYP3A4 Inhibitors

**Generic**	**Brand**
Ketoconazole (systemic use)	--
Itraconazole	Onmel; Sporanox; Sporanox Pulsepak
Posaconazole	Noxafil
Clarithromycin	Biaxin; Biaxin XL; Biaxin XL Pac; Prevpac; Omeclamox-Pak
Aprepitant	Emend
Domperidone	--
Atazanavir	Reyataz; Evotaz
Indinavir	Crixivan
Nefazodone	--
Nelfinavir	Viracept
Ritonavir	Norvir; Kaletra; Technivie; Viekira Pak; Viekira XR
Saquinavir	Invirase
Telithromycin	Ketek
Voriconazole	Vfend

Table 3. Exclusionary Medications: Proton Pump Inhibitors

**Generic**	**Brand**
Dexlansoprazole	Dexilant
Esomeprazole	Nexium; Vimovo
Lansoprazole	First-Lansoprazole; GoodSense Lansoprazole [OTC]; Heartburn Relief 24 Hour [OTC]; Heartburn Treatment 24 Hour [OTC]; Prevacid 24HR [OTC]; Prevacid SoluTab; Prevpac
Omeprazole	First-Omeprazole; Omeclamox-Pak; Omeprazole+Syrspend SF Alka; Prilosec [OTC]; Yosprala; Zegrid
Pantoprazole	Protonix
Rabeprazole	Aciphex; AcipHex Sprinkle

Table 4. Exclusionary Medications: Strong CYP3A4 Inducers

**Generic**	**Brand**
Carbamazepine	Carbatrol; Epitol; Equetro; TEGretol; TEGretol-XR
Dexamethasone (systemic use)	Dexamethasone Intensol; DexPak; DoubleDex
Phenytoin	Dilantin; Dilantin Infatabs; Phenytek; Phenytoin Infatabs
Phenobarbital	Phenobarb; Donnatal; Donnatal Extentabs; Phenohytro
Rifampin	Rifadin; Rifamate; Rifater
Rifabutin	Mycobutin
St. John's Wort	--

Table 5. Exclusionary Medications: P-glycoprotein Inhibitors

Generic	Brand
Amiodarone	Cordarone; Nexterone; Pacerone
Clarithromycin	Biaxin; Biaxin XL; Biaxin XL Pac; Prevpac; Omeclamox-Pak
Erythromycin (systemic use)	EES; Ery-Tab; EryPed; Erythrocin; PCE
Ketoconazole (systemic use)	--
Quinidine	BioQuin Durules; Quinate; Nudexta
Saquinavir	Invirase
Verapamil	Calan; Calan SR; Isoptin SR; Verelan; Tarka

Table 6. Exclusionary Medications: P-glycoprotein Inducers

Generic	Brand
Carbamazepine	Carbatrol; Epitol; Equetro; TEGretol; TEGretol-XR
Rifampin	Rifadin; Rifamate; Rifater
St. John's wort	--
Tipranavir	Aptivus

Table 7. Exclusionary Medications: Anticoagulants

Generic Name	Brand Name
Warfarin	Coumadin
Dabigatran	Pradaxa
Edoxaban	Savaysa
Rivaroxaban	Xarelto
Apixaban	Eliquis

Table 8. Precautionary Medications: Drugs with possible or conditional risk of Torsades de Pointes

**Anticholinergic**	**Antibiotics**	**CNS Stimulants**
Tolterodine	Garenoxacin	Amphetamine
**Anticonvulsants**	Gemifloxacin	Dexmethylphenidate
Ezogabine	Norfloxacin	Dextroamphetamine
Felbamate	Ofloxacin	Lisdexamfetamine
Fosphenytoin	Metronidazole	Methamphetamine
**Antidepressants**	Sulfamethoxazole and Trimethoprim	Methylphenidate
Amitriptyline	Telavancin	Phentermine
Desipramine	**Antineoplastics**	**Contrast Media**
Doxepin	Capecitabine	Gadobenate
Fluoxetine	Degarelix	Iodinated ionic contrast media
Fluvoxamine	Eribulin	Perflutren lipid microspheres
Imipramine	Lenvatinib	**Diuretics**
Maprotiline	Leuprolide	Bendroflumethiazide
Nortriptyline	Sorafenib	Furosemide
Paroxetine	Vorinostat	Hydrochlorothiazide
Sertraline	**Antipsychotics**	Indapamide
**Antiemetics**	Amisulpiride	Torsemide
Dolasetron	Asenapine	**GI Agents**
Promethazine	Clozapine	Famotidine
Tropisetron	Cyamemazine	Metoclpramide
**Antihistamines**	Flupentixol	**Opioids**
Diphenhydramine	Iloperidone	Buprenorphine
Hydroxyzine	Olanzapine	Hydrocodone
**Antifungals**	Paliperidone	**Miscellaneous**
Amphotericin B	Perphenazine	Alfuzosin
	Pimavanserin**	
Itraconazole	Pipamperone	Apomorphine
	Quetiapine**	
Ketoconazole	Risperidone	Atomoxetine
Posaconazole	Ziprasidone	Chloral hydrate
Voriconazole	Zotepine	Fingolimod
**Antimalarials**	**Beta-agonists**	Galantamine
Artenimol + piperaquine	Albuterol	Ivabradine
Hydroxychloroquine	Arformoterol	Lithium

**Antiretrovirals**	Formoterol	Loperamide
Atazanavir	Isoproterenol	Mifeprisfepristone
Efavirenz	Levalbuterol	Mirabegron
Nelfinavir	Metaproterenol	Moxepril
Rilpivirine	Olodaterol	Octreotide
Ritonavir	Salmetrol	Oxytocin
Saquinavir	Terbutaline	Pasireotide
		Ranolazine
**Antiviral**	**Calcium Channel Blockers**	Tetrabenazine
Foscarnet	Isradipine	Tiapride
		Vardenafil
		Tizanidine

**Pimavanserin and Quetiapine are also metabolized by CYP3A4. Therefore, low doses of quetiapine should be used when given with nilotinib (moderate inhibitor of CYP3A4). For pimavanserin, a dose of 17 mg once daily is recommended when given with a strong CYP3A4 inhibitor. For quetiapine, it is recommended to decrease the dose by one-sixth when given with a strong CYP3A4 inhibitor.

APPENDIX 3: STEERING COMMITTEE

Brian Fiske, PhD
Senior Vice President, Research Programs, MJFF, New York, NY

Kalpana Merchant, PhD
President and CSO TransThera Consulting Co, Portland, OR (MJFF Consultant, Therapeutic Development)

Professor Christopher S. Coffey, PhD
Chief Biostatistics, University of Iowa, Iowa City IA,

Helen Matthews
Chief Operating Officer, The Cure Parkinson's Trust, UK

Richard Wyse, MD
Director Research and Development, The Cure Parkinson's Trust, UK

Professor Patrik Brundin
Van Andel Research Institute, Grand Rapids, MI

Professor David Simon
Harvard Medical School Beth Israel Deaconess Medical Center, Boston, MA

Professor Michael Schwarzschild
Massachusetts General Hospital, Boston, MA

David Weiner, MD,
Chief Medical Officer Lumos Pharma, Austin, TX (MJFF Consultant, Therapeutic Development)

Charles Venuto, Pharm D
CHET CTCC, University of Rochester, Rochester, NY

Gary Rafaloff, MS
Parkinson's Disease Patient Advocate
