# Supplementary material for: Efficacy of Nilotinib in Patients With Moderately Advanced Parkinson Disease: A Randomized Clinical Trial
Source: JAMA Neurol. 2020 Dec 14;78(3):1–9. doi: 10.1001/jamaneurol.2020.4725 (PMC7737147; doi:10.1001/jamaneurol.2020.4725)
Supplement: Supplement 2. — Statistical analysis plan [file jamaneurol-e204725-s002.pdf]

NILO-PD

Statistical Analysis Plan
(Cohort 1)

A Randomized, Double-Blind, Placebo-Controlled, Phase IIa, Parallel Group, Two Cohort Study to Define the Safety, Tolerability, Clinical, and Exploratory Biological Activity of the Chronic Administration of Nilotinib in Participants with Parkinson's Disease (PD)

Tanya Simuni, MD
Professor of Neurology
Director, Parkinson's Disease and Movement Disorders Center
Northwestern University Feinberg School of Medicine
Principal Investigator

Christopher S. Coffey, PhD
Professor of Biostatistics
University of Iowa
Principal Investigator of Biostatistics Coordinating Center

VERSION 1.0
September 24, 2019

STATISTICAL ANALYSIS PLAN SIGNATURE PAGE

NILO-PD: A Randomized, Double-Blind, Placebo-Controlled, Phase IIa, Parallel Group, Two-Cohort Study to Define the Safety, Tolerability, Clinical, and Exploratory Biological Activity of the Chronic Administration of Nilotinib in Participants with Parkinson's Disease (PD)

Principal Investigator Approval

Signature: Tanya Simuni **Date:** 9.24.2019
Name: Tanya Simuni, MD
Affiliation: Northwestern University Feinberg School of Medicine

Biostatistics Coordinating Center Approval

Signature: Christopher S. Coffey **Date:** 09/25/19
Name: Christopher S. Coffey, PhD
Affiliation: University of Iowa

Clinical Coordinating Center Approval

Signature: Cynthia Casaceli

Digitally signed by Cynthia Casaceli
DN: cn=Cynthia Casaceli, o=University of Rochester/CHET, ou=Clinical Trials Coordination Center, email=cindy.casaceli@chet.rochester.edu, c=US
Date: 2019.09.25 08:25:14 -04'00'

Date:
Name: Cynthia Casaceli, MBA
Affiliation: University of Rochester

PREFACE

This Statistical Analysis Plan (SAP) describes the planned analyses for cohort 1 of the NILO-PD study, funded by the Michael J. Fox Foundation for Parkinson's Research (MJFF). The planned analyses identified in this SAP are intended to support the completion of a final report for cohort 1 that will be prepared and presented to the study sponsor. Should the study continue to enroll cohort 2, a separate SAP will be prepared for that cohort. All final, planned analyses identified in this SAP will be performed only after the last randomized study participant in cohort 1 has completed the study, and all data have been cleaned and verified in accord with applicable standard operating procedures at the NILO-PD Data Coordinating Center (DCC). Once all cohort 1 data have been cleaned and verified, a "locked" version of the data will be used for reporting the final study results. Key statistics and study results will be presented verbally to the PI, Clinical Coordinating Center (CCC), DCC, Data and Safety Monitoring Board (DSMB), and Steering Committee (SC) for review and discussion following database lock and prior to completion of the final report for cohort 1.

Due to the exploratory nature of this study, we will not attempt to make any type of adjustment for multiple comparisons. Correspondingly, unless specified differently, all hypotheses will be assessed at the 0.05 significance level.

1. STUDY DESIGN

Nilotinib is approved by the U.S. Food and Drug Administration (FDA) for certain types of leukemia, but not for Parkinson's disease (PD). A small number of cell and animal models suggest that nilotinib may positively affect the alpha (α)-synuclein pathology observed in PD (Hebron et al, 2013; Hebron et al, 2014; Karuppagounder et al, 2014; Lonskaya et al, 2013; Lonskaya et al, 2014a; Lonskaya et al, 2014b; Mahul-Mellier et al, 2014; Wenqiang et al, 2014). A small open-label clinical study that lacked a placebo control group tested the safety and tolerability of nilotinib in PD patients for the first time, and explored its efficacy (Pagan et al, 2016). Although preliminary data appear promising, the study design precludes firm conclusions about safety, tolerability, optimal dose, or efficacy (Wyse et al, 2016). Hence, an opportunity exists to rigorously establish the safety, tolerability, and optimal dose of nilotinib to enable and inform the conduct of future definitive efficacy studies in PD patients. All participants will be treated for 6 months, and followed for two months post drug evaluation. Hence, the duration for each participant in cohort 1 is expected to be approximately 8.5 months from screening to post drug evaluation.

The primary objective of cohort 1 is to determine whether either of the doses are sufficiently safe and tolerated to justify consideration of moving forward with cohort 2 (early/de novo PD patients). This objective will be accomplished by comparing the percentage of participants who complete the study on their assigned dose across the three arms. Although the decision will be primarily based on tolerability, we will also compare the safety profiles and CSF PK analysis across the three groups and utilize all this information in making a final decision. At the end of cohort 1, we will make one of three decisions:

- If the 300 mg dose is tolerable, and has an acceptable safety profile, this dose will be chosen for consideration in the second cohort.
- If the 300 mg dose is not tolerable, or if the 300 mg dose is tolerable but has an unacceptable safety profile, then we will consider the 150 mg dose. If the 150 mg dose is tolerable, and has an acceptable safety profile, this dose will be chosen for consideration in the second cohort.
- If neither dose is tolerable, or if all tolerable doses have unacceptable safety profiles, we will conclude that no dose appears suitably safe and tolerable for inclusion in the second cohort. Under this scenario, the trial will not proceed to a second cohort.

A key secondary objective of cohort 1 is to conduct a single group futility hypothesis in each PD group comparing the observed change in MDS-UPDRS Part III between baseline and month 6 to determine whether the study can rule out the large change previously reported in the prior clinical study (Pagan et al, 2016). Other secondary objectives will involve examining the degree of symptomatic effect of nilotinib as measured by the change in MDS-UPDRS Part III shortly after initiation of study drug (between baseline and 1 month), and after discontinuation of study drug [between month 6 (end of treatment) and month 7 (1 month after treatment)]. For all participants, assessment of the potential symptomatic effect will be done using the MDS-UPDRS Part III collected in the ON (at least one-hour post dose) state. An additional secondary objective will assess the impact of nilotinib on the progression of PD disability over the course of the 6-month treatment period. Sparse

PK sampling will be conducted to assess serum and CSF concentrations of nilotinib throughout the duration of the study.

Dosage reductions for tolerability or safety can occur at any time during the study. If study drug is temporarily suspended for occurrences other than study drug related safety or tolerability issues, study drug may be resumed by the site investigator. Drug suspensions lasting more than 7 days require re-titration. If drug suspension is less than 7 days, the participant can restart study drug at prior dosing level. Participants will not be allowed to remain in the study if study drug is permanently discontinued. Once the decision to permanently discontinue study drug is made, a premature withdrawal visit should be conducted. The participant will be required to return to the study site for the 30 and 60-day safety follow-up visits (V05 & V06).

The site investigator may also withdraw study drug from a participant in the event of intercurrent illness, adverse events, other reasons concerning the health or well-being of the participant, non-compliance, protocol violation, or other administrative reasons. Premature withdrawal will also be implemented in the case of emergency disclosure of study drug treatment. Reasons for withdrawal of the participant prior to completion of the study must be stated in the eCRF and in the site source documentation for all study participants who were enrolled in the study. The participant will be encouraged to remain in the study, and return for the 1 month and 2-month post-drug evaluations (V05 & V06) as a safety follow-up.

Since the data on the safety profile of the drug, and prior clinical experience with nilotinib, were limited to an advanced PD population with cognitive deficits, PDD, and/or DLB, it was felt prudent to validate and expand the data on nilotinib safety and tolerability in the moderate/advanced PD population. It is expected that tolerability of nilotinib in this moderate/advanced cohort will be lower than that observed in an early/de novo PD population. Such data are important to establish, provided that nilotinib, if shown to have disease modifying benefit, will be administered long-term to PD patients across all disease stages. Another important objective of the study is to establish the magnitude of potential symptomatic effect of nilotinib and dopaminergic therapy interaction. These data will be essential for the design of the future disease modifying trials.

If shown to be safe and well tolerable, future development of nilotinib will target disease modification objectives. Disease modification has historically been tested in cohorts of de novo PD population. To pursue future disease modification trials, safety and tolerability of nilotinib must be established in the de novo population as well. While we do not expect that the de novo population will have a lower tolerability and safety threshold, this hypothesis must be tested in a subsequent study. If at least one dose is shown to be safe and tolerable in cohort 1, and nilotinib CSF PK samples demonstrate expected CNS penetration, the study steering committee will consider the possibility of selecting a dose for consideration in cohort 2. If the study proceeds, participants in cohort 2 will be treated with the selected dose determined in cohort 1 or matching placebo.

1.1 Primary Objective

The primary objective is to assess the safety and tolerability of the daily oral administration of nilotinib (150 mg or 300 mg once daily) in moderate/advanced PD participants.

1.2 Key Secondary Objective

To conduct a futility analysis within each treated group in Cohort 1 by comparing the observed change in MDS-UPDRS Part III “ON” score between baseline (BL) and Month 6 (V04).

1.3 Additional Secondary Objectives

(1) *To establish the degree of symptomatic effect of nilotinib as measured by the change in MDS-UPDRS Part III score in the defined medications ON state between:*

- ***(a)*** *Baseline (BL) & 1 Month (V02)*
- ***(b)*** *Final Visit on Study Drug (V04 or PW) & 30 Days Off Study Drug (V05)*

(2) *To explore the impact of nilotinib on progression of PD disability as measured by the change in the MDS-UPDRS Part III score in the defined medications OFF state between baseline and 6 months.*

2. PRIMARY ENDPOINTS

2.1. Tolerability

The primary endpoint for tolerability is defined as the proportion (percentage) of study participants who completed the 6-month study treatment period while active on their originally assigned dose. Study completion can occur despite temporary drug interruptions, protocol deviations, etc. Conversely, any participant who must be removed from study drug (either participant-initiated or researcher initiated), completes the study on a dose below their assigned dose, or fails to complete the study (for any reason) will be deemed not to have tolerated their assigned medication. Tolerability will be assessed by comparing the percentage of participants enrolled in each dosage group who are able to complete the study on their originally assigned dosage group across all groups.

Dosage reductions can occur at any time during the study. In case of intolerability at two capsules, daily dosage reduction to one capsule daily should be attempted prior to discontinuing study drug. Participants will reduce their dosage in a blinded fashion by 1 capsule (equivalent to 150 mg of nilotinib or placebo). Participants unable to tolerate one capsule will be taken off the study drug and will not be allowed to continue in the study. Following dosage reductions for tolerability reasons, participants may be re-challenged once during the study titration phase at the discretion of the site investigator. Participants may resume titration to two capsules daily dosage at the discretion of the site investigator, provided the intolerability issues have resolved. Participants that are unable to tolerate the dose increase will be reduced to one capsule daily and enter the maintenance phase of the study. Participants unable to tolerate one capsule daily will be taken off the study drug and will follow procedures outlined for premature withdrawal. No rechallenges will be allowed after safety visit 02 (SV02 – Day 60). Visit 03 (Day 90) will be the formal beginning of the maintenance phase of the study.

Dose adjustments may also be needed for safety reasons, including dose adjustment for QT prolongation (ECG with QTc > 480 msec), for myelosuppression, for selected non-hematologic laboratory abnormalities, or for new cardiac and vascular occlusive events. If a participant discontinues study drug for safety reasons, the participant should complete a premature withdrawal visit either while the participant is still taking the study drug or as soon as possible after the drug discontinuation. Adverse events will be followed for 30 days after a participant's last dose of study drug. Participants will be encouraged to return for the 30 and 60 days post drug evaluation (V05 & V06). Dosage suspension for safety reasons can occur at any time during the study. Re-challenge will be allowed when abnormalities have resolved fully.

2.2 Safety

The primary endpoint for safety is defined as the proportion of study participants who experience any treatment-related serious adverse events (SAEs). Safety will be primarily assessed by examining the frequency of treatment-related SAEs across all treatment groups. Additional safety assessments will involve further comparisons of adverse events (AEs) and overall SAEs across the three groups.

For the purposes of this study, an AE is defined as any untoward medical occurrence associated with the use of a drug in humans, whether or not considered drug related. FDA and Office of Human Research Protection (OHR) requirements for reporting AEs will be followed. AEs are generally detected in two ways:

- Clinical → Symptoms reported by the study participant or signs detected on examination
- Ancillary Tests → Abnormalities of vital signs, laboratory tests, and other diagnostic procedures

An AE can be any unfavorable and unintended sign (e.g., an abnormal laboratory finding), symptom, or disease temporally associated with the use of a drug, and does not imply any judgment about causality. An AE can arise with any use of the drug (e.g., off-label use, use in combination with another drug), and with any route of administration, formulation, or dose (including an overdose). Some examples of potential AEs are:

- A change, excluding minor fluctuations, in the nature, severity, frequency, or duration of a pre-existing condition
- A deterioration in the participant's condition due to the participant's primary disease or a pre-existing condition
- Development of an intercurrent illness during the study
- Development of symptoms which may or may not be related to the use of a concomitant medication or study drug

- Appearance of abnormal laboratory results or significant shifts from baseline, but still within the reference ranges, which the investigator considers clinically important.

At each visit, the site study staff will assess AEs by recording all voluntary complaints of the participant, and by assessment of clinical and laboratory features. At each study visit, the occurrence of AEs should be sought by non-leading questioning of the participant and caregiver during the study. AEs may also be identified when the participant and/or caregiver spontaneously volunteered them. Collection of AEs will begin immediately following signing of the ICF through the final study visit. When possible, AEs should be reported by diagnosis (if known) rather than individual signs and symptoms. The site investigator is responsible for monitoring each participant closely, and recording all observed or volunteered AEs. All AEs identified should be recorded into the AE eCRF in the EDC within 5 business days of the site learning of a new AE. The AE description must include:

- Type of Event
- Start and Stop Dates (when available)
- Intensity / Severity
- Seriousness (does the AE meet the definition of an SAE)
- Causality (relation to investigational product and disease)
- Outcome
- Action taken regarding investigational product

Study participants who sign consent and receive investigational treatment will be monitored for AEs from the time they sign consent. All AEs will be followed until resolution, stabilization, or 30 days after a participant's last dose of study drug (whichever occurs first). All AEs will be recoded into MedDRA terms by the DCC.

The Site Investigator will assess the severity (intensity) for each AE reported during the study. The assessment will be based on the Site Investigator's clinical judgment as follows:

- Mild: An event that causes no limitations of usual activities
- Moderate: An event that causes some limitations of usual activities
- Severe: An event that prevents carrying out usual activities

The Site Investigator is initially responsible for classifying AEs as serious or non-serious. A serious adverse event (SAE) is an AE or suspected adverse reaction occurring at any dose of the investigational product (including placebo) that is considered serious if it meets one or more of the following criteria:

- Results in death
- Is life-threatening (i.e., a study participant is at substantial risk of death at the time the AE occurs, on continued use of the medical product might have resulted in death; not an AE where occurrence in a more serious form might have caused death)
- Requires inpatient hospitalization or prolongation of existing hospitalization
  - o Emergency room visits that do not result in admission to the hospital should be evaluated for one of the other serious outcomes (e.g., life-threatening; required intervention to prevent permanent impairment or damage; other serious medically important event)
  - o Does not include hospitalization for:
    - ▪ Elective or pre-planned treatment for a pre-existing condition which has not worsened since signing the informed consent
    - ▪ Social reasons (study participant admitted for reasons other than medical, e.g., lives far from the hospital or has no place to sleep) and/or respite care in the absence of any deterioration of the participant's general condition
  - o Complications that occur during hospitalization are AEs. If a complication prolongs hospitalization of fulfills any other serious criteria, the event is an SAE
- Results in persistent or significant disability or permanent damage
  - o This serious criterion applies if the "disability" caused by the reported AE results in a substantial disruption of the study participant's ability to carry out normal life function (i.e., if the AE resulted

in a significant, persistent, or permanent change, impairment, damage, or disruption in the patient's body function/structure, physical activities, and/or quality of life)

- Results in a congenital anomaly / birth defect
  - Applicable if there is suspicion that exposure to the medical product prior to conception or during pregnancy may have resulted in an adverse outcome in the child
- Other Serious (Important Medical Events)
  - Applicable when the AE does not fit the other outcomes, but the event may jeopardize the participant and may require medical or surgical intervention (treatment) to prevent one of the other outcomes.
  - Category also includes any event the site investigator or Independent Medical Monitor (IMM) judges to be serious or which would suggest a significant hazard, contraindication, side effect, or precaution. Examples include allergic bronchospasm (a serious problem with breathing) requiring treatment in an emergency room, serious blood dyscrasias (blood disorders) or seizures/convulsions that do not result in hospitalization, or the development of drug dependency or drug abuse.
  - Can also involve the withdrawal of a participant from a study due to abnormal lab values (excluding screening labs)

For the purposes of this study, a treatment-related AE (also referred to as an Adverse Drug Reaction) is defined as any noxious or unintended response to a medicinal product related to any dose. The phrase "responses to a medicinal product" means that a causal relationship between a medicinal product and an AE is at least a reasonable possibility, i.e., the relationship cannot be ruled out. Therefore, a subset of AEs can be classified as treatment related if there is thought to be a causal relationship to study drug. For each reported AE, the causality (i.e., relationship to study treatment) must be assessed by the site investigator and recorded on the AE Log according to the classifications below. Ambiguous cases should be considered as having a reasonable possibility of a causal relationship unless further evidence becomes available to refute this.

- <u>Definite (Causal relationship is certain)</u>: The temporal relationship between drug exposure and AE onset/course is reasonable, there is a clinically compatible response to dechallenge, other causes have been eliminated, and the event must be definitive pharmacologically or phenomenologically, using a satisfactory rechallenge procedure if necessary
- <u>Probable (High degree of certainty for causal relationship)</u>: The temporal relationship between drug exposure and AE onset/course is reasonable. There is a clinically compatible response to dechallenge (rechallenge is not required), and other causes have been eliminated or are unlikely
- <u>Possible (Causal relationship is uncertain)</u>: The temporal relationship between drug exposure and AE onset/course is reasonable or unknown, dechallenge or rechallenge information is either unknown or unequivocal, and while other potential causes may not exist, a causal relationship to the study drug does not appear probable
- <u>Unlikely (Not reasonable related, although a causal relationship cannot be rule out)</u>: While the temporal relationship between drug exposure and AE onset/course does not preclude causality, there is a clear alternate cause that is more likely to have caused the AE than the study drug
- <u>Unrelated (No possible relationship)</u>: The temporal relationship between drug exposure and AE onset/course is unreasonable or incompatible, or a causal relationship to study drug is implausible

As this is a double-blind study, the causality assessment should be made under the assumption that the study participant is receiving active study medication. If considering unblinding, this assessment should be made prior to unblinding to avoid bias. For the purposes of this study, an SAE is considered to be treatment-related if the attribution is possible, probable, or definite. The site investigator determines causality of the AE for the purposes of initial SAE reporting. The IMM will make the final adjudication on causality.

Expectedness will also be determined for each SAE reported during the study. The assessment of "Expected" vs. "Unexpected" will be based on the IMM clinical judgement. The judgement will be based on the known spectrum of AEs reported with the investigational drug nilotinib, as reported in the package insert (Tasigna, revised 2018), and limited data from a previous study in a small PD population (Pagan et al, 2016).

3. SECONDARY ENDPOINTS

A key secondary efficacy outcome will involve a comparison of the Movement Disorder Society Unified Parkinson's Disease Rating Scale (MDS-UPDRS) over time. The other secondary objectives will also utilize the MDS-UPDRS, which has four parts:

- Part I (Non-motor experiences of daily living), compromising:
  - Part IA concerning behaviors that are assessed by the site investigator with all pertinent information from patients and caregivers
  - Part IB that is completed by the patient with or without the aid of the caregiver, but independently of the site investigator
- Part II (Motor experiences of daily living), designed to be a self-administered questionnaire like Part 1B, but similarly can be reviewed by the site investigator to ensure completeness and clarity
- Part III (Motor examination) has instructions for the rater to give or demonstrate to the patient; it is completed by the clinician rater
- Part IV (Motor complications) to be completed by the clinician rater

All participants will have an assessment of the motor exam (Part III) in both the practically defined medications OFF (12 hours post dose) and ON (based on the participant / site investigator defined best ON and/or approximately 1-hour post dose) states for designated visits per the schedule of activities. Participants will self-administer Parts IB and II, but will review responses for accuracy and clarity with the site investigator or coordinator. Parts IA, III, and IV must be conducted by the site investigator. All parts of the MDS-UPDRS will be conducted at study visits, as indicated on the schedule of activities. The use of the MDS-UPDRS is responsive to core instrument recommendations for the Quality of Life subdomain of the National Institute of Neurological Disorders and Stroke (NINDS) Common Data Elements (CDEs) for PD, and to FDA guidance encouraging the use of patient-reported outcomes (PROs) as a substantial portion of the responses are patient-reported. Ideally, the same site investigator should assess all participants in parts IA and III of the MDS-UPDRS at all study visits.

While the study is not powered to test efficacy of nilotinib as a putative disease modifying intervention, the data on the magnitude of the effect of nilotinib versus placebo on the rate of progression in PD disability will be collected. These data will guide the decision to pursue further clinical evaluation for that indication as well as provide essential data for the power calculations and sample size estimation for future disease modifying trials.

4. ENROLLMENT & RANDOMIZATION

A total of approximately 75 study participants will be randomized into the study. Participants will be assigned a Participant ID number at the time they sign/date the informed consent form. An Enrollment ID number will be assigned at the randomization visit to confirm enrollment and proper receipt of the randomized study drug assignment. Following the screening phase, study participants who continue to meet entry criteria will be enrolled and randomly assigned in a 1:1:1 manner to one of three treatment groups: nilotinib 150 mg, nilotinib 300 mg, or matching placebo. The BCC will generate the randomization codes that identify the Enrollment ID/randomization numbers that will be included on the labels of the bottles/kits that will be supplied to sites.

The treatment assignment for each participant will be assigned by a randomized code using randomizations tables generated by the NILO-PD DCC. The process will proceed as follows:

- Once the participant qualifies for the randomized phase of the study, the Site Investigator or Study Coordinator will enter data into the eClinical system that will assign a unique, 4-digit Enrollment ID (randomization kit) number that will match a study drug kit. These numbers are assigned in a randomized order, rather than sequentially.
- The randomization algorithm and participant enrollment process will be implemented through the Internet accessible Electronic Data Capture (EDC) system using authenticated, password-protected accounts for each study site. The EDC system will verify participant eligibility based on inclusion/exclusion criteria.
- Once the online enrollment process is completed, the site will print an Enrollment Verification Report that verifies the participant has been randomized. The report will note the Enrollment ID number that was assigned that corresponds to the drug kit number and the upcoming study visit windows.

- Once a participant has been allocated an Enrollment ID number, this number cannot be assigned to another participant.

5. TABULATIONS

All study participants who provide informed consent will be accounted for in this study. The number of randomized participants and their study disposition will be reported overall, and by treatment group. A CONSORT diagram summarizing the final status of all study participants will be provided. The proportion of randomized study participants completing each visit will be summarized as in Table 1 below.

Table 5.1: Completeness of Study Data by Visit

Visit	Placebo (N = XX)	150 mg (N = XX)	300 mg (N = XX)
BL	XX (XX%)	XX (XX%)	XX (XX%)
V01	XX (XX%)	XX (XX%)	XX (XX%)
V02	XX (XX%)	XX (XX%)	XX (XX%)
V03	XX (XX%)	XX (XX%)	XX (XX%)
V04	XX (XX%)	XX (XX%)	XX (XX%)
V05	XX (XX%)	XX (XX%)	XX (XX%)
V06	XX (XX%)	XX (XX%)	XX (XX%)

Additional summary reports will describe:

- Number of study participants consented, eligible, and randomized by site
- Reasons for exclusion
- Completeness of study visits, case report forms, and collection of LPs
- Protocol deviations
- Study Drug Dose Reductions
- Study Drug Suspensions
- Early Study Terminations
- Reportable Events
- Study Drug Compliance: The number of expected doses of nilotinib or placebo received will be summarized as a continuous variable. Study participants will be deemed as “compliant” if the percentage of pills taken falls between 80% and 125% (inclusive) of expected pills.

Baseline participant data will also be summarized by treatment group (nilotinib 150 mg / nilotinib 300 mg / placebo) with respect to important demographic characteristics. Distribution of numeric and categorical variables will be tabulated by treatment group and overall. Numeric variables will be summarized by the mean, standard deviation, minimum, and maximum. Categorical variables will be tabulated by proportions of percentages. Variables that will be summarized include:

- Demographic Characteristics:
    - Age
    - Gender
    - Race
    - Ethnicity
    - PD Duration (Years)
    - Age at Diagnosis
    - Initial Symptoms
        - Resting Tremor
        - Rigidity
        - Bradykinesia

    - Postural Instability
    - Other
- Baseline Motor Disability PD Characteristics
  - MDS-UPDRS Total “OFF” Score
  - MDS-UPDRS Total “ON” Score
  - Hoehn & Yahr “ON” Score
  - Levodopa Equivalent Dose
  - Class of Dopaminergic Therapy
    - Dopamine Replacement
    - COMT Inhibitors
    - Dopamine Agonists
    - MAOB Inhibitors
    - Other
- Other Baseline PD Characteristics
  - Education-Adjusted MOCA Score
  - Beck Depression Inventory (BDI) II Score
  - Mattis Dementia Rating Scale
  - Parkinson’s Disease Sleep Scale (PDSS)
  - Modified Schwab & England (S&E)
  - Parkinson’s Disease Quality of Life Questionnaire 39 (PDQ-39)
  - European Quality of Life Scale (EQ-5D)

6. ANALYSIS POPULATIONS

6.1. Primary and Secondary Efficacy Analyses (6 Month Treatment Period)

6.1.1. Intent-to-Treat (ITT) Population

For the primary safety and tolerability outcomes, as well as all secondary outcomes during the 6 month treatment period, all analyses will be performed consistent with the intention-to-treat (ITT) principle – per the recent JAMA Guide to Statistics and Medicine (Detry, 2014). All participants will be analyzed as-randomized, which means they will be analyzed based on the treatment to which they were randomized, whether or not they remained compliant with respect to taking study medication.

6.1.2. Per Protocol (PP) Population

To assess the sensitivity of key results, and to obtain knowledge regarding the potential effects when the protocol was strictly adhered to, we will also replicate all primary and key secondary efficacy objectives using a per protocol population. The per-protocol population includes the subset of all ITT participants who satisfy both of the following conditions:

- Completed full 6-month treatment period with no dosing interruptions, and with 80% to 125% compliance (both limit values inclusive) with assigned study medication as randomized in the double-blind phase
- No major protocol deviations (defined as any alteration/modification to the protocol that has the potential to negatively impact participant safety, integrity of the study, or ability to draw conclusions from study data)

6.2. Post Treatment Analyses

Assessment of the post-treatment secondary objective will include the subset of all randomized participants who satisfy both of the following conditions:

- Had a final visit on treatment at 3 months or later past randomization
- Had a 30-day safety assessment after stopping treatment

7. PRIMARY ANALYSES

Primary Tolerability Hypothesis: To assess whether either 150 mg or 300 mg once daily dosing of nilotinib is sufficiently tolerable, compared to placebo

The first primary objective of this study is to evaluate the tolerability of nilotinib versus placebo. This primary objective will be met by conducting a comparison of the proportion of study participants who complete the study while active on their assigned dose among the placebo group and each of the treatment groups. Separate tests will be conducted for each of the treatment arms (i.e., a separate test for placebo vs. nilotinib 150 mg & placebo vs. nilotinib 300 mg) with a significance level of 0.05 for each test.

Below, we introduce some key notation that we will use to describe the analysis plan for the proposed trial:

- Let p_{150} represent the true (unknown) percentage of participants treated with nilotinib 150 mg who will tolerate the assigned medication and dosage
- Let p_{300} represent the true (unknown) percentage of participants treated with nilotinib 300 mg who will tolerate the assigned medication and dosage
- Let p_p represent the true (unknown) percentage of participants treated with placebo who will tolerate the assigned medication

The primary tolerability null hypotheses being tested in this trial are that participants treated with either dose of nilotinib will have similar tolerability to participants treated with placebo. The primary hypotheses will compare the overall tolerability rates for each nilotinib arm vs. placebo participants. Using the notation above, the two one-sided hypothesis tests below will be assessed:

$$H_{01}: p_{150} = p_p \quad \text{vs.} \quad H_{A1}: p_{150} < p_p$$

AND

$$H_{02}: p_{300} = p_p \quad \text{vs.} \; H_{A2}: p_{300} < pp$$

Therefore, rejecting the null hypothesis suggests that the particular dose group of nilotinib shows significantly less tolerability relevant to the placebo group. If we do not reject the null hypothesis, this would imply that there are not significant enough concerns with tolerability to preclude moving forward with further study of one or both doses of nilotinib. Specifically, this would provide justification for proceeding with further study of the drug (pending the results of the safety assessment). Each hypothesis will be assessed via a Pearson chi-square test. Due to randomization, it is unlikely that important covariates will be imbalanced in this study. However, given the small sample size, this cannot be dismissed. We will assess for important baseline imbalances, and if any imbalances exist, we will fit a logistic regression model that will allow adjustment for the relevant covariates with important imbalances present.

The results of the tolerability analysis will be summarized graphically, with a bar plot showing the proportion difference and one-sided CI for each group comparison. The results will also be depicted as in Table 2 below.

Table 7.1. Proportion of Participants Meeting Study Definition for Tolerability

Group	Placebo (N = XX)	150 mg (N = XX)	300 mg (N = XX)
Number (%)	XX (XX%)	XX (XX%)	XX (XX%)
P-Value for comparison versus placebo	N/A	p = X.XX	p = X.XX
Decision	N/A	Tolerable (Yes/No?)	Tolerable (Yes/No?)

The primary tolerability analysis will follow the intent-to-treat (ITT) paradigm. All randomized participants must be included in the primary ITT analysis, and will be analyzed in the treatment group to which they were initially randomized. As such, it will be critically important to minimize the occurrence of missing data. Obviously, the optimal strategy for dealing with missing data is to make every effort to obtain complete data during the conduct of the study. The DCC data managers and CCC protocol coordinators will work diligently and use a variety of methods to minimize the percentage of missing data in this trial. Nevertheless, there is likely to be a small percentage of missing data. For the primary analysis, we will take a conservative approach and assume that any participant not completing the study for any reason did not tolerate study therapy. To further assess the potential dependence of the results of the primary analysis to these missing values, a series of sensitivity

analyses will be conducted. We will attempt to collect as much detailed information as possible for participants who are lost to follow-up or prematurely withdraw from the study due to any reason. Any participants who terminate treatment or the study early due to tolerability concerns (AEs) will always be counted as not tolerating their assigned treatment dose. For participants that do not provide complete data, and for which there is no clear tolerability concern, we will analyze the data using the following strategies:

- Using Only Observed Data (No Imputation)
- Worst-Case Scenario: Assume all missing participants in the treatment group did not tolerate the dose, and all missing participants in the placebo group did tolerate the dose
- Best-Case Scenario: Assume all missing participants in the treatment group did tolerate the dose, and all missing participants in the placebo group did not tolerate the dose

The results of these analyses will provide important information regarding the sensitivity of the findings to the missing data, and will be critical to the steering committee in assessing the full value of the study results.

Primary Objective #2: *The second primary objective is to assess the safety of the daily oral administration of nilotinib (150 mg or 300 mg once daily)*

The safety objective will be assessed by comparing the proportion of study participants with any SAE among the placebo group and each of the treatment groups (separate tests for placebo vs. nilotinib 150 mg & placebo vs. nilotinib 300 mg). The safety analysis will be conducted in a similar manner as the tolerability analysis. Each study participant will be classified according to whether they experienced a treatment-emergent SAE ("yes" or "no"). The specific safety primary hypotheses will be assessed in two ways. First, the percentage of participants who experience any treatment-related SAE, overall and by body system, will be compared across each of the treatment groups versus placebo using standard chi-square tests. Then, the rates of treatment-related SAEs across the treatment groups will be compared using a Poisson regression model. If there are specific differences within any specific SOC, then additional assessments will compare differences across groups for specific MedDRA terms within that SOC to further explore the cause of the observed differences. To provide an overall summary of the safety of each nilotinib dose, additional safety assessments will involve further comparisons of AEs and SAEs across the three groups. In addition to detailed safety tables that will be provided as part of a final version of standard DSMB tables, high level safety results will be summarized as shown in Table 7.2 below.

Table 7.2. Summary of Safety Analyses

	Placebo (N = XX)	150 mg (N = XX)	300 mg (N = XX)	p-value (150 vs. pbo)	p-value (300 vs. pbo
Any AE	XX (XX%)	XX (XX%)	XX (XX%)	X.XX	X.XX
Any Treatment-Related AE	XX (XX%)	XX (XX%)	XX (XX%)	X.XX	X.XX
Any Serious AE	XX (XX%)	XX (XX%)	XX (XX%)	X.XX	X.XX
Early Drug Reductions (Any)	XX (XX%)	XX (XX%)	XX (XX%)	X.XX	X.XX
Early Drug Reductions (Due to AE)	XX (XX%)	XX (XX%)	XX (XX%)	X.XX	X.XX
Early Drug Discontinuations (Any)	XX (XX%)	XX (XX%)	XX (XX%)	X.XX	X.XX
Early Drug Discontinuations (Due to AE)	XX (XX%)	XX (XX%)	XX (XX%)	X.XX	X.XX
Premature Withdrawals (Any)	XX (XX%)	XX (XX%)	XX (XX%)	X.XX	X.XX
Premature Withdrawals (Due to AE)	XX (XX%)	XX (XX%)	XX (XX%)	X.XX	X.XX
Deaths (Any)	XX (XX%)	XX (XX%)	XX (XX%)	X.XX	X.XX
Deaths (Due to AE)	XX (XX%)	XX (XX%)	XX (XX%)	X.XX	X.XX

Decision Algorithm Based on Primary Tolerability & Safety Hypotheses

All this information will be synthesized and reviewed by the Sponsor and steering committee at the end of cohort 1. This summary information will be critical for making the dosing decision for consideration in cohort 2 (or deciding not to proceed with cohort 2). At the end of cohort 1, a decision rule will be invoked to decide regarding the "best" dose for consideration of moving forward with in a potential second cohort of early/de novo PD participants. This decision will be based on a combination of the results from the tolerability and safety hypotheses. In general, the decision algorithm will proceed as follows:

- Did the 300 mg nilotinib group show suitable tolerability?
    - If 'Yes', then did the 300 mg nilotinib group show suitable safety?
        - If 'Yes', then **recommend the 300 mg nilotinib dose for consideration in a second cohort**
        - If 'No', then did the 150 mg nilotinib group show suitable tolerability?
            - If 'Yes', then **recommend the 150 mg nilotinib dose for consideration in a second cohort**
            - If 'No; then **conclude that neither dose is sufficiently safe and tolerable to justify further exploration**
    - If 'No', then did the 150 mg nilotinib group show suitable tolerability?
        - If 'Yes', then did the 150 mg nilotinib group show suitable safety?
            - If 'Yes', then **recommend the 150 mg nilotinib dose for consideration in a second cohort**
            - If 'No', then **conclude that neither dose is sufficiently safe and tolerable to justify further exploration**
        - If 'No', then **conclude that neither dose is sufficiently safe and tolerable to justify further exploration**

If either of the doses are considered sufficiently safe and tolerable, the study steering committee will also review the results of the CSF PK analysis. If at least one dose is shown to be safe and tolerable, and nilotinib CSF PK samples demonstrate expected CNS penetration, the steering committee will consider moving that dose forward into a second cohort of early/de novo PD participants.

8. KEY SECONDARY ANALYSES

Key Secondary Objective: *To conduct a futility analysis within each treated group.*

A key secondary objective of this study is to conduct a single group hypothesis within each PD group to assess "futility" for replicating the large difference observed in a previously published study (Pagano et al, 2016). This key secondary objective will be assessed by conducting a futility analysis to examine the change of the MDS-UPDRS Part III between baseline and 6 months. Within each of the two dose groups, this futility analysis will compare the observed change in MDS-UPDRS Part III to the observed change from the Pagan et al (2016) study to determine whether we can rule out the large change previously reported – i.e., declare "futility". This will be based on a single group hypothesis test within each dose group comparing the observed change in the MDS-UPDRS Part III scores collected ON (at least one-hour post dose) between baseline and 6 months to the observed change reported from the Pagan et al (2016) study to determine whether we can rule out the large change previously reported. Based on the results from Table 2 in the Pagan et al (2016) publication, which used the UPDRS:

- Mean change over 6 months in the 150 mg nilotinib group = 7.0 reduction (std dev = 12.9)
- Mean change over 6 months in the 300 mg nilotinib group = 10.8 reduction (std dev = 7.8)

For consistency, we will test a similar hypothesis within each group using the smallest observed reduction. However, since we will be using the MDS-UPDRS, we include a correction factor of 1.4 as specified in Goetz et al (2012) and use the smallest reduction for both futility tests. In other words, the futility test within each nilotinib dose group will be based on the following set of hypotheses:

$$H_0: \delta \leq -9.8 \ (7 \times 1.4) \quad \text{vs.} \quad H_A: \delta > -9.8$$

Where δ represents the change from baseline to 6 months in the corresponding group being tested. Since this is an early phase exploratory study, and we want to ensure adequate power for ruling out the large effects previously observed, we assume an alpha level of 0.10 for each one-sided hypothesis of interest.

The raw data for all three treatment groups will be summarized graphically, as in the following table:

Table 8.1: Descriptive Statistics of MDS-UPDRS Part III "ON" Scores Over Time

Visit	Nilotinib 150 mg (N = XX)	Nilotinib 300 mg (N = XX)	Placebo (N = XX)
Baseline<br>Mean (SD)<br>(Min, Max)<br>Missing	 XX.X (XX)<br>(XX.X , XX.X)<br>XX	 XX.X (XX)<br>(XX.X , XX.X)<br>XX	 XX.X (XX)<br>(XX.X , XX.X)<br>XX
Day 14 (Month = 0.5)<br>Mean (SD)<br>(Min, Max)<br>Missing	 XX.X (XX)<br>(XX.X , XX.X)<br>XX	 XX.X (XX)<br>(XX.X , XX.X)<br>XX	 XX.X (XX)<br>(XX.X , XX.X)<br>XX
Day 30 (Month = 1)<br>Mean (SD)<br>(Min, Max)<br>Missing	 XX.X (XX)<br>(XX.X , XX.X)<br>XX	 XX.X (XX)<br>(XX.X , XX.X)<br>XX	 XX.X (XX)<br>(XX.X , XX.X)<br>XX
Month 3<br>Mean (SD)<br>(Min, Max)<br>Missing	 XX.X (XX)<br>(XX.X , XX.X)<br>XX	 XX.X (XX)<br>(XX.X , XX.X)<br>XX	 XX.X (XX)<br>(XX.X , XX.X)<br>XX
Month 6<br>Mean (SD)<br>(Min, Max)<br>Missing	 XX.X (XX)<br>(XX.X , XX.X)<br>XX	 XX.X (XX)<br>(XX.X , XX.X)<br>XX	 XX.X (XX)<br>(XX.X , XX.X)<br>XX

The null hypotheses of interest can be evaluated based on an assessment of parameter estimates from a linear mixed effects model (LMM: Laird & Waire, 1982). LMMs are advantageous for longitudinal clinical trials because they can account for dependency due to repeated measures with relatively few parameters, which potentially enhances statistical efficiency. Furthermore, LMMs can accommodate incomplete cases (i.e., missing data), which is expected in this study due to dropout. LMMs are typically estimated using maximum likelihood methods (Verbeke & Molenberghs, 2000) that yield valid inferences with incomplete cases under the widely applicable assumption that the missing data are ignorable (Little & Rubin, 2002).

For this analysis, assuming linear change over time, the LMM can be written in the following manner:

$$Y_{ij} = \alpha + \beta_1 X_{1ij} + \beta_2 t_{ij} X_{2i} + \beta_3 t_{ij} X_{3i} + \beta_4 t_{ij} X_{4i} + a_i + b_i + \varepsilon_{ij}$$

where:

- Y_{ij} is the MDS-UPDRS Part III "ON" score for the ith participant at the jth time point (day 14, day 30, month 3, month 6, or time of PW visit)
- α is the common intercept parameter
- t_{ij} denotes time (in months)
- X_{1i} = LEDD for the ith participant at the jth time point
- X_{2i} = 1 if i^{th} participant is in the nilotinib 150 mg group; = 0 otherwise
- X_{3i} = 1 if i^{th} participant is in the nilotinib 300 mg group; = 0 otherwise
- X_{4i} = 1 if i^{th} participant is in the placebo group; = 0 otherwise
- a_i and b_i are random effects (random intercepts and slopes)
- ε_{ij} is a random error term

Assuming a common intercept constrains the baseline means to be equal, which controls for any initial imbalance that might occur due to empirical randomization (Senn, 2013). We make the typical assumptions:

$$\begin{bmatrix} a_i \\ b_i \end{bmatrix} \sim N(\mathbf{0}, \mathbf{G}), \quad \varepsilon_{ij} \sim N(0, \sigma^2 \boldsymbol{I}_i), \quad \text{and} \quad \begin{bmatrix} a_i \\ b_i \end{bmatrix} \perp \varepsilon_{ij}$$

The main parameters of interest are β_1, β_2, and β_3, which correspond to the estimated slopes in the two treatment and placebo groups, respectively. The two desired tests of interest for this key secondary objective can be obtained by testing the null hypothesis of the following contrasts:

- For the nilotinib 150 mg group comparison:

$$H_0: 6\beta_2 \leq -9.8 \quad \text{vs.} \quad H_A: 6\beta_2 > -9.8$$

- For the nilotinib 300 mg group comparison:

$$H_0: 6\beta_3 \leq -9.8 \quad \text{vs.} \quad H_A: 6\beta_3 > -9.8$$

Each null hypothesis can be evaluated with the corresponding likelihood ratio test. Rejecting the null hypothesis implies that a future study is unlikely to observe the large effects reported in the Pagan et al (2016) study. However, that would not rule out potentially meaningful effects on a smaller scale. Furthermore, note that failure to reject the null hypothesis does not imply that a significant difference has been observed. If the null hypothesis is not rejected, or if the hypothesis is rejected but the observed differences seem potentially meaningful (just on a smaller scale of magnitude than previously reported), further study should be warranted.

Since the longitudinal measurements will span up to five time points per participant (baseline, 14 days, 30 days, 3 months, and 6 months), there is a possibility that the MDS-UPDRS Part III "ON" trajectories will not be linear. To assess the sensitivity of the results to the assumption of linearity, non-linear trends will be modeled by inserting time as a categorical variable, modeling change from baseline, and including baseline values as a covariate. For this modeling approach, visits that were performed outside of an expected scheduled visit will be categorized into the next expected scheduled visit. We will compare model fit between the non-linear and linear models using the Akaike Information Criterion (AIC), and will report results from the non-linear model if the AIC suggests that the nonlinear model provides a better fit. In this case, the primary comparisons will involve a comparison of the estimated change from baseline means within each of the two groups at the 6-month visit.

The residuals of the fitted statistical models will be examined for evidence of departure from assumptions, such as normality. If assumptions appear to be grossly violated, then transformations of response variables might be considered. Finally, the proposed study involves multiple sites, which is a potential source of additional variation. Patients within a site tend to be correlated due to similarity of environment, e.g. because of testing by the same set of clinicians (Localio et al, 2001). Sensitivity analyses may also be conducted to account for site variation by augmenting the models described above with additional random effects and associated variance components.

9. ADDITIONAL SECONDARY ANALYSES

The study will also assess additional secondary objectives comparing the change in MDS-UPDRS scores over time between groups to establish both the potential degree of symptomatic effect of nilotinib and to assess the impact of nilotinib on the progression of PD disability.

Additional Secondary Objective #1: *To establish the degree of symptomatic effect of nilotinib as measured by the change in MDS-UPDRS Part III ON score between baseline and visit 2 (1 month).*

To assess the degree of symptomatic effect of nilotinib, we will compare the change in MDS-UPDRS Part III ON score from baseline to 1 month. To assess this hypothesis, the same LMM described in section 8 will be used. Assuming the linear model provides the best fit, an initial two degree of freedom test will be utilized to test for any differences among the slopes for the three groups:

$$H_0: \begin{bmatrix} \beta_2 - \beta_4 \\ \beta_3 - \beta_4 \end{bmatrix} = \begin{bmatrix} 0 \\ 0 \end{bmatrix} \quad vs. \quad H_A: \begin{bmatrix} \beta_2 - \beta_4 \\ \beta_3 - \beta_4 \end{bmatrix} \neq \begin{bmatrix} 0 \\ 0 \end{bmatrix}$$

If this hypothesis is not rejected, then we will conclude that there is not sufficient evidence to conclude that either nilotinib treatment group shows a symptomatic effect on MDS-UPDRS Part III "ON" scores during the first month of the treatment period. If the null hypothesis is rejected, pairwise comparisons will be utilized to assess which pairs of treatment groups show significant differences over time with respect to the MDS-UPDRS Part III "ON" scores.

If a non-linear model is chosen to provide a better fit in the analysis described in section 8 above, the primary comparisons for this additional secondary objective will involve a two degree of freedom test for differences in the change from baseline means between each treatment group versus placebo at the month 1 visit.

Additional Secondary Objective #2: *To establish the degree of symptomatic effect of nilotinib as measured by the change in MDS-UPDRS Part III ON score between the final visit on study drug & 30 days off study drug.*

To further assess the degree of symptomatic effect, we will also examine the change in MDS-UPDRS Part III ON after the end of treatment or final study visit. For this analysis, the following LMM will be used:

$$Y_{ij} = \alpha + \beta_1 X_{1i} + \beta_2 X_{2i} + \beta_3 t_{ij} X_{3i} + \beta_4 t_{ij} X_{4i} + \beta_5 t_{ij} X_{5i} + a_i + b_i + \varepsilon_{ij}$$

where:

- Y_{ij} is the change from final visit on study drug to the jth time point (day 30 post, day 60 post) for the ith participant
- α is the common intercept parameter
- t_{ij} denotes time (in months; day 30 = 1 & day 60 = 2)
- X_{1i} represents the MDS-UPDRS Part III "ON" score at the final visit on study drug
- X_{2i} = LEDD for the ith participant at the jth time point
- X_{3i} = 1 if i[th] participant is in the nilotinib 150 mg group; = 0 otherwise
- X_{4i} = 1 if i[th] participant is in the nilotinib 300 mg group; = 0 otherwise
- X_{5i} = 1 if i[th] participant is in the placebo group; = 0 otherwise
- a_i and b_i are random effects (random intercepts and slopes)
- ε_{ij} is a random error term

Assuming the linear model provides the best fit, and initial two degree of freedom test will be utilized to test for any differences among the slopes for the three groups:

$$H_0: \begin{bmatrix} \beta_3 - \beta_5 \\ \beta_4 - \beta_5 \end{bmatrix} = \begin{bmatrix} 0 \\ 0 \end{bmatrix} \quad vs. \quad H_A: \begin{bmatrix} \beta_3 - \beta_5 \\ \beta_4 - \beta_5 \end{bmatrix} \neq \begin{bmatrix} 0 \\ 0 \end{bmatrix}$$

If this hypothesis is not rejected, then we will conclude that there is not sufficient evidence to conclude that either nilotinib treatment group shows a symptomatic effect on MDS-UPDRS Part III "ON" scores during the first month after the end of treatment. If the null hypothesis is rejected, pairwise comparisons will be utilized to assess which pairs of treatment groups show significant differences over time with respect to the MDS-UPDRS Part III "ON" scores.

As above, to assess the sensitivity of the results to the assumption of linearity, non-linear trends will be modeled by inserting time as a categorical variable. We will compare model fit between the non-linear and linear models using the AIC, and will report results from the non-linear model if the AIC suggests that the non-linear model provides a better fit. If the assumption of linearity is supported, any significant findings would suggest symptomatic effects at both 30 and 60 days. If a non-linear model is chosen to provide a better fit, the primary comparisons for this additional secondary objective will involve a two degree of freedom test for differences in the change from baseline means between each treatment group versus placebo at 30 days following the end of treatment. If a significant symptomatic effect is observed for either treatment arm versus placebo at 30 days post dose in the non-linear model, we will also examine the effect at 60 days post dose to explore the longevity of the effect.

Additional Secondary Objective #3: *To explore the impact of nilotinib in progression of PD disability as measured by the change in the MDS-UPDRS Part III in the defined medications OFF state between baseline and 6 months.*

To assess the impact of nilotinib on the progression of PD disability, we will examine the change in MDS-UPDRS from baseline to 6 months (evaluated for both the "OFF" and "ON" states).

To assess this hypothesis for the MDS-UPDRS Part III "ON" scores, the same LMM described in section 8 will be used. Assuming the linear model provides the best fit, an initial two degree of freedom test will be utilized to test for any differences among the slopes for the three groups:

$$H_0: \begin{bmatrix} \beta_2 - \beta_4 \\ \beta_3 - \beta_4 \end{bmatrix} = \begin{bmatrix} 0 \\ 0 \end{bmatrix} \quad vs. \quad H_A: \begin{bmatrix} \beta_2 - \beta_4 \\ \beta_3 - \beta_4 \end{bmatrix} \neq \begin{bmatrix} 0 \\ 0 \end{bmatrix}$$

If this hypothesis is not rejected, then we will conclude that there is not sufficient evidence to conclude that either nilotinib treatment group shows differences in the progression of MDS-UPDRS Part III "ON" scores over the 6-month treatment period. If the null hypothesis is rejected, pairwise comparisons will be utilized to assess which pairs of treatment groups show significant differences over time with respect to the MDS-UPDRS Part III "ON" scores.

If a non-linear model is chosen to provide a better fit in the analysis described in section 8 above, the primary comparisons for this additional secondary objective will involve a two degree of freedom test for differences in the change from baseline means between each treatment group versus placebo at the month 6 visit.

A similar approach will be utilized to assess the MDS-UPDRS Part III “OFF” scores over time, except the model specified in section 8 will be simplified to include only baseline, month 3, and month 6 values (since an “OFF” assessment was not performed at days 14 and 30 per the SOA).

10. OTHER EXPLORATORY ANALYSES

Several additional exploratory analyses are also planned, but will not be included as part of the final report. These exploratory analyses will be assessed to further examine the impact of nilotinib on disability, quality of life, and functional status. These additional analyses will include, but are not limited to:

- Change in Motor Disability
  - The change in MDS-UPDRS Total score (ON and OFF scores)
  - The change in the ambulatory capacity (Sum of 5 MDS-UPDRS questions: Falling, Freezing, Walking, Gait, Postural Stability)
  - Analysis of ST utilization as measured by levodopa equivalent dose
  - Severity of motor complications as measured by MDS-UPDRS Part IV subscale (complications of therapy)
- Change in Cognitive Disability – Changes in cognitive function, as measured by the change in Mattis Dementia Rating Scale (DRS-2)
- Changes in Sleep Function – Changes in sleep quality, as measured by the Parkinson’s Disease Sleep Scale (PDSS)
- Measures of Global Disability – Clinician and Participant Global Impression of Change Score (CGI)
- Measures of Functional Status and Quality of Life
  - The change in the MDS-UPDRS Motor and Non-Motor Experiences and Daily Living subscores
  - The change in the Modified Schwab and England scale (S&E)
  - The change in Parkinson Disease Quality of Life Questionnaire 39 (PDQ-39)
  - The change in European Quality of Life Scale (EQ-5D)
- Blindedness and treatment expectations assessments, as measured by questionnaires completed by the site investigator and participant
- Pharmacokinetics and Pharmacodynamics
  - To determine serum PK of nilotinib in patients with PD
  - To investigate ability of nilotinib to cross the blood brain barrier in patients at a dose(s) that is tolerable and safe, as measured by dose-dependent increases in unbound nilotinib concentrations in the cerebrospinal fluid (CSF)
  - To investigate factors that could affect the PK of nilotinib, such as age, sex, PD severity, race/ethnicity
  - To investigate the ability of nilotinib to engage its known molecular target(s) at a dose that is tolerable and safe, as measured by biomarkers of pathway inhibition in the serum and CSF
  - To investigate the relationship between nilotinib exposures in serum and CSF with its ability to engage known molecular target(s), as measured by biomarkers of pathway inhibition in the serum and CSF
- To explore impact of nilotinib on:
  - Dopaminergic system as measured by an increase in dopamine and its metabolites in CSF [e.g., homovanillic acid (HVA) level]
  - Other biomarkers linked to potential mechanism of neuroprotection (including, but not limited to, CSF α-synuclein, total tau, phosphorylated tau, etc.)

Additional blood/serum/plasma/DNA/CSF samples will be collected and stored for future research.

11. SAMPLE SIZE JUSTIFICATION

11.1. Primary Objective

For the purposes of computing the required sample size, we assumed that at least 90% of participants on placebo will meet the study definition of "tolerability" (completing the study on the assigned dose). Furthermore, it was determined that an absolute decrease of 30% or greater with respect to tolerability for active treatment arms versus placebo would provide sufficient tolerability concerns that would not warrant further study of that dose. Hence, the sample size was chosen to provide sufficient power to compare an expected 90% tolerability rate in the placebo group versus a 60% or lower tolerability rate in each of the treatment groups. Under these assumptions, and considering a one-sided test with an alpha = 0.05, a total of 25 participants per group provides 80% power for the moderate/advanced cohort. With three groups, this leads to a total of approximately 75 required participants for cohort 1.

11.2 Major Secondary Objective

For the major secondary objective, we utilized preliminary estimates from the Pagan et al (2016) publication, as described in section 8. Converting from the UPDRS used in the Pagan et al (2016) study to the MDS-UPDRS utilized in this study, the test is set up in the manner described in section 15.3.3. Furthermore, we believe that since the conversion factor maintains the relationships between the mean and standard deviation, although the expected values might differ the power calculations shown below give a valid estimate of the power for the proposed study. Using these estimates, we computed the power of the study to detect "futility" based on comparisons of the change within the two nilotinib dosage groups. Specifically, we assume an alpha level of 0.10 for the one-sided futility hypothesis, and assume a standard deviation of 12.9. The table below shows the power for rejecting the null hypothesis of interest (i.e., declaring "futility"), computed across a range of assumed values for the true change from baseline in the nilotinib treated patients. This table demonstrates the benefits of using the futility hypothesis (with differences shown in both UPDRS and converted MDS-UPDRS units).

Table 11.1. Power for Futility Analysis for Key Secondary Objective

True Change in UPDRS	-7.0	-3.5	0	+3.5
True Change in MDS-UPDRS	-9.8	-4.9	0	+4.9
Pr(Futility)	10%	52%	92%	99%

When the true change in MDS-UPDRS part III ON score over the 6-month treatment period involves an increase in scores (implying progression of the disease), the test will declare "futility" with high probability. Likewise, when the true change in MDS-UPDRS part III ON score is equal to or greater than the observed change in the Pagan et al (2016) study, the trial has a very low chance of incorrectly declaring futility. If the observed change in the MDS-UPDRS Part III ON score is half that observed in the prior study (a reduction of 3.5), then the study has marginal power (52%) to declare futility. If there is no observed reduction associated with treatment, the study has 92% power to declare futility. Given the sample size limitations of the study, this provides a reasonable chance of addressing the main futility question of interest regarding whether the observed large observed effect in the prior study can be refuted. The test of futility will have adequate power mainly if the direction of effect is in the opposite direction from that observed in the previous study (i.e., if there is no reduction, only a potentially smaller increase in the MDS-UPDRS over time). These sample size calculations were based on the observed change for the nilotinib 150 mg group in the Pagan et al (2016) study. Since bigger effects were observed over 6 months for the nilotinib 300 mg group, the trial has adequate power for addressing futility in the nilotinib 300 mg group in a similar manner.

12. SAFETY MONITORING

All aspects of the study will be monitored by authorized individuals in compliance with Good Clinical Practice (GCP) and applicable regulations. Refer to the Safety Management Plan for additional details.

12.1 Clinical Monitor

The Clinical Monitor (CM) is responsible for frequent review of blinded safety data, including AEs, SAEs, laboratory and EKG data. Additionally, the CM is responsible for assisting sites in responding appropriately to safety concerns. If the CM encounters significant safety concerns, they will discuss the severity, importance,

and relevance of the safety concern with the PI, the Site Investigator, and the Project Manager, and will work with all parties to determine appropriate action.

12.2 Independent Medical Monitor

The study sponsor will appoint an Independent Medical Monitor (IMM). The IMM will review (in real-time) all events that meet the definition of an SAE. The IMM will review SAE reports, and classify each with respect to causality, severity, and expectedness as outlined in the Safety Management Plan. The IMM may determine that the SAE is serious, related, and unexpected and requires expedited reporting to the FDA. When this occurs, the IMM may communicate with the CM and/or PI for clarification and to provide further information on the event – especially if the event has not resolved or stabilized by the time of completing of the initial report. This may involve contacting other clinicians responsible for the participant's care, with participant's authorization for release of information, to obtain information on diagnoses, investigations performed, and treatment given. However, the IMM should code all events independent from the CM and/or Site Investigator. Consensus between the Site Investigator and IMM is not required. In case of disagreement between the Site Investigator and IMM, the IMM adjudication prevails for reporting purposes. The IMM will also alert the CTCC PM and PI of any concerns related to SAEs that should be forwarded to the entire Data and Safety Monitoring Board (DSMB) for review via email.

12.2 Data and Safety Monitoring Board

A Data and Safety Monitoring Board (DSMB) will be appointed by MJFF, and will be responsible for periodic review of trial conduct, progress, and safety data throughout the trial. The DSMB will meet at approximately three-month intervals (or as determined by MJFF) to review partially unblinded study data provided by the study statistician. The DSMB will periodically review and evaluate the accumulated data for participant safety, adverse events, study conduct, and study progress. The DSMB may suggest changes to the protocol or consent form to the PI because of AEs. The DSMB may also make recommendations to MJFF concerning continuation, modification, or termination of the study. The frequency and format of DSMB meetings and reports will be established and documented prior to study participant enrollment.

REFERENCES

Detry MA and Lewis RJ (2014). "The Intention-to-Treat Principle: How to Assess the True Effect of Choosing a Medical Treatment," *Journal of the American Medical Association*, 312(1): 85-86.

Goetz CG, Stebbins GT, and Tilley BC (2012). "Calibration of Unified Parkinson's Disease Rating Scale Scores to Movement Disorders Society – Unified Parkinson's Disease Rating Scale Scores," *Movement Disorders*, 27(10): 1239-1242.

Hebron ML, Lonskaya I, and Moussa CE (2013). "Nilotinib Reverses Loss of Dopamine Neurons and Improves Motor Behavior via Autophagic Degradation of Alpha-Synuclein in Parkinson's Disease Models," *Human Molecular Genetics*, 22(16): 3315-3328.

Hebron ML, Lonskaya I, Olopade P, Selby ST, Pagan F, and Moussa CE (2014). "Tyrosine Kinase Inhibition Regulates Early Systemic Immune Changes and Modulates the Neuroimmune Response in Alpha-Synucleinopathy," *Journal of Clinical & Cellular Immunology*, 5: 259.

Karuppagounder SS, Brahmachari S, Lee Y, Dawson VL, Dawson TM, and Ko HS (2014). "The c-Abl Inhibitor, Nilotinib, Protects Dopaminergic Neurons in a Preclinical Animal Model of Parkinson's Disease," *Scientific Reports*, 4: 4874.

Laird NM and Ware JH (1982). "Random-Effects Models for Longitudinal Data," *Biometrics*, 38(4): 963-974.

Little RJA and Rubin DB (2002). *Statistical Analysis with Missing Data, Second Edition*. New York: Wiley.

Localio AR, Berlin JA, Ten Have TR, and Kimmel SE (2001). "Adjustments for Center in Multicenter Studies: An Overview," *Archives of Internal Medicine*, 135(2): 112-123.

Lonskaya I, Hebron M, Chen W, Schachter J, and Moussa C (2014a). "Tau Deletion Impairs Intracellular Beta-Amyloid-42 Clearance and Leads to More Extracellular Plaque Deposition in Gene Transfer Models," *Molecular Neurodegeneration*, 9: 46.

Lonskaya I, Hebron ML, Desforges NM, Franjie A, and Moussa CE (2013). “Tyronise Kinase Inhibition Increases Functional Parkin-Beclin-1 Interaction and Enhances Amyloid Clearance and Cognitive Performance,” *EMBO Molecular Medicine*, 5(8): 1247-1262.

Lonskaya I, Hebron ML, Desforges NM, Schachter JB, and Moussa CE (2014b). “Nilotinib-Induced Autophagic Changes Increase Endogenous Parkin Level and Ubiquitation, Leading to Amyloid Clearance,” *Journal of Molecular Medicine*, 92(4): 373-386.

Mahul-Mellier AL, Fauvet B, Gysbers A, Dikiy I, Oueslati A, Georgeon S, Lamontanara AJ, Bisquett A, Eliezer D, Masliah E, Halliday G, Hantschel O, and Lashuel HA (2014). “c-Abl Phosphorylates Alpha-Synuclein and Regulates Its Degradation: Implication for Alpha-Synuclein Clearance and Contribution to the Pathogenesis of Parkinson’s Disease,” *Human Molecular Genetics*, 23(11): 2858-2879.

Pagan F, Hebron M, Valadez EH, Torres-Yaghi Y, Huang X, Mills RR, Wilmarth BM, Howard H, Dunn C, Carlson A, Lawler A, Rogers SL, Falconer RA, Ahn J, Li Z, and Moussa C (2016). “Nilotinib Effects in Parkinson’s Disease and Dementia with Lewy Bodies,” *Journal of Parkinson’s Disease,* 6(3): 503-517.

Senn S (2013). “Seven Myths of Randomization in Clinical Trials,” *Statistics in Medicine*, 32: 1439-1450.

Tasigna, AMN107, Novartis, Switzerland, revised 2018: https://www.pharma.us.novartis.com/sites/www.pharma.us.novartis.com/files/tasigna.pdf)

Verbeke G and Molenbergs G (2000). *Linear Mixed Models for Longitudinal Data*. New York: Springer.

Wenqiang C, Lonskaya I, Hebron ML, Ibrahim Z, Olszewski RT, Neale JH, and Moussa CE (2014). “Parkin-Mediated Reduction of Nuclear and Soluble TDP-43 Reverses Behavioral Decline in Symptomatic Mice,” *Human Molecular Genetics*, 23(18): 4960-4969.

Wyse RK, Brundin P, and Sherer TB (2016). “Nilotinib – Differentiating the Hope from the Hype,” *Journal of Parkinson’s Disease,* 6(3): 519-522.
