# Supplementary material for: Efficacy of Nilotinib in Patients With Moderately Advanced Parkinson Disease: A Randomized Clinical Trial
Source: JAMA Neurol. 2020 Dec 14;78(3):1–9. doi: 10.1001/jamaneurol.2020.4725 (PMC7737147; doi:10.1001/jamaneurol.2020.4725)
Supplement: Supplement 3. — eMethods. Analytical methods for assessment of monoamines and their metabolites in the CSF and to determine nilotinib concentrations in the serum and CSF eTable 1. Changes in the exploratory outcomes over time eTable 2. Steady state nilotinib serum and csf concentrations at 3 mon around tmax (2 +/-0.5 hr) eTable 3. CSF levels of dopamine, its metabolites by treatment group excluding participants on MAO-B inhibitors eAppendix. Serum and CSF pharmacokinetic study in the dog [file jamaneurol-e204725-s003.pdf]

Supplemental Online Content

Simuni T, Fiske B, Merchant K, et al; the Parkinson Study Group NILO-PD Investigators and Collaborators. Efficacy of nilotinib in patients with moderately advanced Parkinson disease: a randomized clinical trial. *JAMA Neurol*. Published online December 14, 2020. doi:10.1001/jamaneurol.2020.4725

eMethods. Analytical methods for assessment of monoamines and their metabolites in the CSF and to determine nilotinib concentrations in the serum and CSF

eTable 1. Changes in the exploratory outcomes over time

eTable 2. Steady state nilotinib serum and csf concentrations at 3 mon around tmax (2 +/-0.5 hr)

eTable 3. CSF levels of dopamine, its metabolites by treatment group excluding participants on MAO-B inhibitors

eAppendix. Serum and CSF pharmacokinetic study in the dog

This supplemental material has been provided by the authors to give readers additional information about their work.

© 2020 American Medical Association. All rights reserved.

eMethods. **Analytical method for assessment of monoamines and their metabolites in the CSF.**

The CSF biospecimens were analyzed by Biocrates Life Sciences AG (Innsbruck, Austria) using a validated multiplexed, mass spectrometry-based analytical platform. The table below shows the 17 distinct analytes measured and their respective Lower Limit of Detection (LOD) and Upper Limit of Quantification (ULLQ) values. Absolute concentrations of monoamines and related metabolites were determined using procedures adapted from Yamada et al[1]. In brief, samples were subjected to ultracentrifugation and derivatization prior to online solid-phase extraction and LC-MS/MS analysis (Symbiosis Pharma, Spark, Emmen, Netherlands) coupled to an Applied Biosystems API4000 MS/MS-System). Heavy isotope-labeled catecholamines used as internal standards were added to the standard calibration curve as well as to each sample before extraction to correct for random and systematic errors. Each analyte was normalized to its appropriate internal standard, resulting in relative areas.

© 2020 American Medical Association. All rights reserved.

	Metabolite Name	LOD [ng/mL]	ULOQ [ng/mL]
Catecholamine catabolites	3,4-Dihydroxymandelic acid	0.3	25
	3,4-Dihydroxphenylacetic acid (DOPAC)	0.05	100
	3,4-Dihydroxyphenylglycol (DOPEG)	0.01	40
	3-Methoxytyrosine	0.4	300
	3-O-Methyldopamine	0.02	7.5
	4-Hydroxy-3-methoxymandelic acid	0.3	400
	4-Hydroxy-3-methoxyphenylglycol (HMPG)	0.03	150
	Homovanillic Acid (HVA)	0.5	500
	Metanephrine	0.015	7.5
	Normetanephrine	0.02	35
Catecholamine pre-cursor	3,4-Dihydroxyphenylalanine (DOPA)	0.05	19.3
Catecholamines	Adrenaline (Epinephrine)	0.01	2.5
	Dopamine	0.005	1.8
	Noradrenaline (Norepinephrine)	0.04	10
Signal Substance, Serotonin & Metabolite	Histamine	0.05	200
	5-Hydroxy-3-indoleacetic acid (5-HIAA)	0.5	128
	Serotonin (5-HT)	0.1	87.5

© 2020 American Medical Association. All rights reserved.

Analytical methods to determine nilotinib concentrations in the serum and CSF.

Bioanalytics for nilotinib concentrations in serum and CSF was performed at WuXi AppTec (Shanghai, China) under Good Laboratory Practice (GLP) procedures. Frozen biospecimens aliquots were shipped to WuXi on dry ice and stored at -80^0C until analysis of nilotinib concentrations by previously validated LC-MS/MS assays for each matrix. Concentrations of nilotinib were determined using the slope and intercept of calibration curves for each matrix as described below. In addition, quality control samples were included in each batch analyses and data were accepted only if calculated concentrations of the quality control samples were within 15% of their nominal values. The lack of carryover was demonstrated by injecting blank samples after nilotinib injected at Upper Limit of Quantification for each matrix. Incurred sample reproducibility was conducted for each matrix using 10% of study samples to demonstrate the reproducibility of nilotinib concentration in the incurred samples under study storage conditions.

Serum Assay: The reference material was nilotinib and internal standard was $[^{13}C_3{}^{15}N_2]$ AMN107, supplied by Novartis. Human serum was used as a blank matrix for preparation of 8-point calibration curve (2.5 to 5000 ng/mL nilotinib). The Lower Limit of Quantification (LLOQ) and Upper Limit of Quantification (ULOQ) were 2.5 ng/mL and 5000 ng/mL, respectively. Freshly prepared quality control samples (6, 150, 1400 and 4000 ng/mL nilotinib) were tested in each batch.

CSF assay: The reference material was nilotinib and internal standard was nilotinib-d6. Triton X-100 (20 mg/mL) was added to artificial CSF (Harvard Apparatus, Cambridge, MA, USA) to reduce non-specific binding to tubes and used as blank matrix. An 8-point calibration curve (0.200 to 100 ng/mL) was prepared in the blank matrix. The LLOQ and ULOQ for CSF were 0.200 ng/mL and 100 ng/mL, respectively. The quality control samples (0.6, 4, 40 and 75 ng/mL nilotinib) were

© 2020 American Medical Association. All rights reserved.

prepared fresh in human CSF with Triton X-100 (20 mg/mL) and were tested for each batch of CSF analyzed.

Pharmacokinetic Sampling Study Design

On day 14 and months 1 and 2 of the study, pre-dose trough serum samples were collected before administration of the daily dose of study drug . Serum samples were also collected at 2 hours post dose (reported as T_{max}) at month 3 to approximate a maximum concentration (C_{max}), and serum samples at random times relative to dose at months 2, 4, and 6. Serum was also collected in month 7 after a month off the study drug. CSF samples were collected at screening (i.e., pre-drug) 2 hours post dose of study drug at month 3 and a month off the study drug (month 7); the latter CSF collection was optional.

© 2020 American Medical Association. All rights reserved.

eTable 1S: Changes in the Exploratory Outcomes Over Time

	Placebo		Nilotinib 150		Nilotinib 300		
**Measure**	**N**	**Adjusted Change LSM (95% CI)**	**N**	**Adjusted Change LSM (95% CI)**	**N**	**Adjusted Change LSM (95% CI)**	**p value[a]**
**Exploratory Outcomes**							
MDS-UPDRS Total ON, Change from Baseline to 1 Month	25	-3.78 (-7.70,0.14)	25	-0.00 (-3.91,3.91)	26	0.62 (-3.22,4.47)	0.24
MDS-UPDRS Total ON, Change from Baseline to 6 Months	25	-4.35 (-8.33,-0.38)	25	1.55 (-2.48,5.58)	26	-0.02 (-4.04,4.00)	0.11
MDS-UPDRS Total OFF, Change from Baseline to 6 Months	25	-5.48 (-10.48,-0.49)	25	0.07 (-5.12,5.27)	26	-0.31 (-5.38,4.77)	0.23
MDS-UPDRS Total ON, Change from Month 6 to 30 Days Off Drug	25	0.47 (-3.14,4.08)	24	1.51 (-2.65,5.67)	24	1.39 (-2.55,5.33)	0.92
PDQ-39, Change from Baseline to Month 6	25	-0.76 (-4.00,2.47)	25	0.87 (-2.57,4.30)	26	-3.38 (-6.68,-0.07)	0.21
		Mean (SD)		Mean (SD)		Mean (SD)	p value[b]
LEDD at Month 6	24	1050.17 (498.15)	23	954.85 (226.17)	23	1024.38 (408.97)	0.70
CGII-P	24	N (%)	23	N (%)	22	N (%)	p value[c]
Improved<br>No Change<br>Worsened		9 (38%)<br>10 (42%)<br>5 (21%)		8 (35%)<br>12 (52%)<br>3 (13%)		10 (45%)<br>5 (23%)<br>7 (32%)	0.31
CGII-I	24	N (%)	23	N (%)	23	N (%)	p value[c]
Improved<br>No Change<br>Worsened		9 (38%)<br>12 (50%)<br>3 (13%)		5 (22%)<br>13 (57%)<br>5 (22%)		6 (26%)<br>15 (65%)<br>2 (9%)	0.61

[a]p values are from a two degrees of freedom test for any difference in adjusted slopes between the treatment groups
[b]p values are from a two degrees of freedom test for any difference in means at 6 months
[c]p values are from Fisher's Exact Test
[d]Worsening defined as a participant having an increase of ≥ 50% or ≥ 25%, respectively, in MDS-UPDRS Part III “OFF score versus baseline at any time point during the study

© 2020 American Medical Association. All rights reserved.

eTable 2S: Steady State Nilotinib Serum and CSF Concentrations at 3 Mon around Tmax (2 +/-0.5 hr)

	Nilotinib 150	Nilotinib 300
**C_{max} Serum Nilotinib (ng/mL)**		
N	20	21
Geometric Mean (95% CI)	424·29 (343·31, 524·37)	549·33 (485·77, 621·20)
**CSF Nilotinib (ng/mL)**		
N	21	21
Geometric Mean (95% CI)	0·84 (0·61, 1·10)	1·44 (1·10, 1·90)
N < LOD	2	0
**CSF-to-Serum Nilotinib Concentration (%) at C_{max}**		
N	17	20
Geometric Mean (95% CI)	0·19% (0·16%, 0·23%)	0·26% (0·20%, 0·32%)

Values of 0 were imputed for observations below the limit of detection (LOD).
Samples were collected around Tmax defined as 2 +/-0.5 hr post last dose of Nilotinib.
Participants who had samples collected outside of the pre-defined window were excluded from this analysis.

© 2020 American Medical Association. All rights reserved.

eTable 3S. CSF levels of Dopamine, its Metabolites by Treatment Group Excluding Participants on MAO-B Inhibitors

Variable	Placebo (N = 13)	Active 150 (N = 14)	Active 300 (N = 10)	Total (N = 37)	p-value
**Dopamine (DA)**					0.95
Mean (SD)<br>Median<br>(Min, Max)	0.07 (0.06)<br>0.06<br>(0.01, 0.25)	0.05 (0.03)<br>0.04<br>(0.01, 0.10)	0.07 (0.04)<br>0.06<br>(0.02, 0.13)	0.06 (0.04)<br>0.05<br>(0.01, 0.25)	
**3,4-Dihydroxyphenylacetic acid (DOPAC)**					0.35
Mean (SD)<br>Median<br>(Min, Max)	3.08 (2.03)<br>2.70<br>(0.27, 7.37)	2.29 (2.03)<br>1.42<br>(0.39, 6.63)	3.96 (3.75)<br>2.61<br>(0.84, 11.21)	3.02 (2.61)<br>2.39<br>(0.27, 11.21)	
**Homovanillic acid (HVA)**					0.96
Mean (SD)<br>Median<br>(Min, Max)	142.30 (86.21)<br>127.40<br>(34.21, 348.40)	89.98 (63.55)<br>71.88<br>(30.89, 255.80)	111.10 (46.90)<br>119.05<br>(21.65, 169.00)	114.07 (70.72)<br>104.40<br>(21.65, 348.40)	
**DOPAC : DA**					0.89
Mean (SD)<br>Median<br>(Min, Max)	54.98 (47.76)<br>49.53<br>(15.98, 196.27)	45.92 (24.94)<br>39.61<br>(16.64, 111.96)	62.21 (51.47)<br>39.48<br>(18.80, 156.06)	53.51 (41.12)<br>42.56<br>(15.98, 196.27)	
**HVA : DA**					0.27
Mean (SD)<br>Median<br>(Min, Max)	2431.67 (1128.77)<br>2427.34<br>(860.75, 5066.60)	2165.58 (1050.84)<br>2506.25<br>(631.42, 4089.97)	1812.71 (813.00)<br>1680.37<br>(922.85, 3001.78)	2163.70 (1024.14)<br>2387.99<br>(631.42, 5066.60)	

© 2020 American Medical Association. All rights reserved.

Supplement 6~~5~~. **Serum and CSF Pharmacokinetics (PK) Study in the Dog**

In parallel to the human study, we conducted a study in chronically cannulated beagle dogs to assess whether at steady state nilotinib crosses the blood-brain barrier sufficiently and inhibits c-Abl in the brain .

The objective of this study was to establish steady-state PK of nilotinib in the serum and CSF as well as brain exposure at ~Tmax to evaluate its penetration into the CNS.

Study summary

The absolute levels of nilotinib in the CSF at the two doses tested (20 mg/kg and 50 mg/kg per day for 2 weeks) were ~8-10 times higher than those observed in our clinical study. Consistent with the human data, the dog study demonstrated CSF exposures that were <1% of those seen in the serum. The brain levels of unbound nilotinib were 2-4% of serum levels in the dog and fell below the cellular IC_{50} for c-Abl inhibition. In accordance with the apparent poor brain penetration, we failed to detect a decrease in p-Abl levels in the brain as measured by immunoblots.

Methods

<u>Animals and Surgery</u>: The study was conducted at MPI Research (Mattawan, MI, USA) under a protocol approved by the Institutional Animal Care and Use Committee (IACUC). Animal welfare was in compliance with the U.S. Department of Agriculture's (USDA) Animal Welfare Act (9 Code of Federal Regulations (CFR) Parts 1, 2 and 3), the Guide for the Care and Use of Laboratory Animals, Institute of Laboratory Animal Resources (National Academy Press, Washington, D.C.). Ten adult male beagle dogs weighing 9-12 Kg were surgically instrumented with a jugular vein catheter for blood collection as well as an intrathecal catheter between L3 and L4 with an access port for collection of cerebral spinal fluid (CSF) under general anesthesia.

<u>Nilotinib treatment</u>: Dogs were randomly assigned to 2 treatment arms of 5 animals each and

© 2020 American Medical Association. All rights reserved.

received either 20 mg/kg or 50 mg/kg nilotinib in a formulation of 1.5% Avicel/0.3% HPMC in water *via* oral gavage daily for 14 days (dose volume = 5 mL/kg). The lower dose was selected on the basis of dog PK data (Novartis Investigators' Brochure) to target serum PK in the range of human PK at 300 mg of nilotinib. The animals were not fasted prior to dosing and food/fluids were provided *ad libitum*.

Biospecimen Collection and Bioanalytics: Blood and CSF samples were collected from 4 animals of each dose group on Day 14. Blood collection from the jugular vein occurred at pre-dose, and 0.25, 0.5, 1, 2, 4, 8, 12, 24 and 48 hours post-dose and was processed for serum collection. CSF collection occurred at pre-dose, and 1, 2, 4 and 12 hours post-dose. Dosing of the animals was continued on day 15 and 16 as per their originally assigned dosage. On day 16, animals were euthanized 2 hours post-dose (~Tmax) for collection of brain tissue. An additional 2 treatment-naïve dogs without catheterization were necropsied as controls for the brain exposures. All biospecimens were frozen and stored at -60 to -90^{0}C until analyses. Nilotinib concentrations in serum, CSF and brain homogenate were assessed by liquid chromatography-tandem mass spectrometry (LC/MS-MS) using validated analytical methods developed by MPI Research. Bioanalytics was conducted on samples from 4 animals per group (2 extra animals went through surgery and treatment to ensure that at least 4 per group would remain patent and without major tolerability issues).

Results

Table 1: Summary of nilotinib pharmacokinetics in the serum and CSF

Pharmacokinetic Parameter	20 mg/kg (n=4)		50 mg/kg (n=4)	
	Geometric Mean [Interquartile Range]		Geometric Mean [Interquartile Range]	
	Serum	CSF	Serum	CSF

© 2020 American Medical Association. All rights reserved.

C_{max} (ng/mL)	692 [182 - 3118]	6.4 [2.5, 24.2]	2,271 [1808 - 2605]	13.7 [11.8, 16.7]
T_{max} (h)	-- [1.5 - 2.0]	-- [1.5, 2.0]	-- [1.75 - 2.5]	3.4[3.5, 4.0]
C_0/C_{min} (ng/mL)	22.8 [10.3 - 60.9]	0.5 [0.4, 0.6]	342 [256 - 588]	4.1 [3.3, 4.6]
C_{24} (ng/mL)	4.8 [10.9 - 17.7]	--	59.7 [24.2 - 216]	--
$AUC_{0\text{-}tau}$, ng*h/mL	3,482 [679 - 17849]	38.4 [10.9, 140]	17,606 [10169 - 27560]	116 [93, 142]
([CSF]/[Serum] at 2 h) x 100	0.92%		0.60%	

C_{max}: maximum concentration; T_{max}: time to maximum concentration; C_0: concentration at pre-dose sample; C_{min}: minimum concentration observed; C_{24}: concentration at 24-hour post-dose sample; $AUC_{0\text{-}tau}$: area under the time-concentration curve over the dosing interval**.**

High variability was seen in nilotinib concentrations in the 20 mg/kg group for both matrices for reasons unknown. The C_{max} concentration of nilotinib in the serum at 20 mg/kg was in the range of that observed in the 300 mg arm of the clinical study. As seen in the clinical data, the CSF levels of nilotinib were <1% of those observed in the serum.

Brain Tissue Concentrations of Nilotinib in the Dog

On day 16, paired samples of serum and brain tissue were collected at 2 h post-dose to target the T_{max} concentrations. The average (standard deviations) serum concentrations in the 20 mg/kg and 50 mg/kg dose groups were 1359 (1318) ng/mL and 1214 (597) ng/mL, respectively. The average (standard deviations) brain tissue concentrations in the 20 mg/kg and 50 mg/kg dose groups were 4132 (4158) ng/g and 3355 (1392) ng/g, respectively. When corrected for protein binding (reported dog plasma protein binding is 98.3%; Xia et al., 2012), the mean (standard deviation)

© 2020 American Medical Association. All rights reserved.

unbound concentration of nilotinib in the brain tissues were 70 (71) and 57 (24) ng/g tissue, which reflects 4% and 2.3% of serum concentration for 20 mg and 50 mg/kg arms, respectively.

Assessment of c-Abl inhibition in the dog brain.

Methods

The dog brain tissues (cerebral cortex and cerebellum) were homogenized in RIPA buffer (50 mM Tris, pH 8.0, 150mM NaCl, 1% NonidetTM P-40, 1% SDS, 0.5% sodium deoxycholate) supplemented with phosphatase inhibitor cocktail II and III (Sigma-Aldrich), and complete protease inhibitor mixture. The homogenate was centrifuged (20 min at 4° C, 15000 rpm) and the resulting supernatant was collected. The protein concentrations of the samples were measured by BCA assay. Samples were electrophoresed on SDS-PAGE gels and transformed to nitrocellulose membranes. Membranes were blocked with 5% non-fat dry milk (wt/vol) in Tris-buffered saline with Tween-20 (TBS-T) and incubated with primary antibodies (mouse anti-c-Abl (# 554148, BD Biosciences); Rabbit anti-pY245 c-Abl (# 2861, Cell Signaling)). After an incubation with horseradish peroxidase-conjugated secondary antibody (anti-mouse IgG (# 7076S, Cell Signaling) or Anti-rabbit (# 7074S Cell Signaling)), the immunoblot signal was detected using chemiluminescent substrates (Thermo Scientific). The integrated band densities were measured using ImageJ software and the relative densities of pY245 c-Abl and c-Abl were calculated with respect to total c-Abl and beta-actin respectively.

Results

Nilotinib treatment did not affect total c-Abl or pY245 c-Abl levels normalized to total c-Abl in the cortex or the cerebellum. The mean and (standard deviation) for the ratio of optical density of pY245 c-Abl to total c-Abl bands in the cerebral cortex for the vehicle, 20 mg/kg nilotinib and 50

© 2020 American Medical Association. All rights reserved.

mg/kg nilotinib groups, respectively, were: 2.133±0.82, 2.551±1.153 and 4.060±1.393. The corresponding values for the cerebellum were: 0.290±0.276, 0.766±0.544 and 0.792±0.444. Statistical analyses using Sidak's multiple comparison test showed $p>0.15$ for the cortex and $p>0.46$ for the cerebellum.

Taken together, this carefully designed dog PK study with pharmacodynamic assessment indicates that nilotinib has poor brain penetration resulting in concentrations in the CNS that are not sufficient to inhibit c-Abl activity in the brain tissue.

1. Yamada H, Yamahara A, Yasuda S, et al. Dansyl chloride derivatization of methamphetamine: a method with advantages for screening and analysis of methamphetamine in urine. *J Anal Toxicol.* 2002;26(1):17-22.

© 2020 American Medical Association. All rights reserved.
