# Supplementary material for: Efficacy of Nilotinib in Patients With Moderately Advanced Parkinson Disease: A Randomized Clinical Trial
Source: JAMA Neurol. 2020 Dec 14;78(3):1–9. doi: 10.1001/jamaneurol.2020.4725 (PMC7737147; doi:10.1001/jamaneurol.2020.4725)
Supplement: Supplement 4. — Nonauthor Collaborators. Parkinson Study Group NILO-PD Investigators [file jamaneurol-e204725-s004.pdf]

Supplemental Online Content: Nonauthor Collaborators

*Indicates required information. Only first name, last name, and suffix will appear in PubMed.

Group Name: The Parkinson Study Group NILO-PD Investigators and Collaborators

First Name, Middle Initial*	Last Name*	Suffix*	Academic Degrees	Institution	Location (city, state/province, country)	Role or Contribution, eg, chair, principal investigator	Subgroup, eg, Steering Committee
Jamie	Adams		MD	University of Rochester	Rochester, NY, USA	Medical Monitor	Steering Committee
Erika	Augustine		MD	University of Rochester	Rochester, NY, USA	Medical Monitor	
Deborah	Baker		AAS	University of Rochester	Rochester, NY, USA	Administrative Ast.	
Alicia	Brocht		MS	University of Rochester	Rochester, NY, USA	Unblinded Programmer	
Cindy	Casaceli		MBA	University of Rochester	Rochester, NY, USA	Clinical Trials Coordination Center Administrative Principal Investigator	
Ken	Eaton		MS	University of Rochester	Rochester, NY, USA	Programmer	
Sue	Henderson		AAS	University of Rochester	Rochester, NY, USA	Project Manager	
Nichole	McMullen		LPN	University of Rochester	Rochester, NY, USA	Clinical Database Manager	
Phounsavath	Muneath		BS	University of Rochester	Rochester, NY, USA	Clinical Database Manager	
Laura	Trusso		MS	University of Rochester	Rochester, NY, USA	Project Manager	
Carlinda	Field		CCRP	University of Rochester	Rochester, NY, USA	Monitor	
Saurav	Brahmachari		PhD	Johns Hopkins University	Baltimore, MD, USA		
Liana	Rosenthal		MD	Johns Hopkins University	Baltimore, MD, USA	Site Investigator	
Emily	Carman		RN	Johns Hopkins University	Baltimore, MD, USA	Site Coordinator	
Cornelia	Kamp		MBA	Clinical Materials Services Unit	Rochester, NY, USA	Director of Drug Packaging Unit	
Patrick	Bolger		R.Ph., MBA	Clinical Materials Services Unit	Rochester, NY, USA	Director of Drug Packaging Unit	
Claire	Wegel		MPH	Indiana University - Biologics Laboratory	Bloomington, IN, USA	Safety Lab Vendor	
Holly	Reynolds		BA	Indiana University - Biologics Laboratory	Bloomington, IN, USA	Safety Lab Vendor	
Oren	Levy		MD	Columbia University Medical Center	New York, NY, USA	Site Investigator	

Supplemental Online Content: Nonauthor Collaborators

*Indicates required information. Only first name, last name, and suffix will appear in PubMed.

First Name, Middle Initial*	Last Name*	Suffix*	Academic Degrees	Institution	Location (city, state/province, country)	Role or Contribution, eg, chair, principal investigator	Subgroup, eg, Steering Committee
Amber	Servi		BA	Columbia University Medical Center	New York, NY, USA	Site Coordinator	
Kelvin	Chou		MD	University of Michigan	Ann Arbor, MI, USA	Site Investigator	
Angela S	Stovall		BS	University of Michigan	Ann Arbor, MI, USA	Site Coordinator	
Gian	Pal		MD	Rush University Medical Center	Chicago, IL, USA	Site Investigator	
Kellie	Keith		BA	Oregon Health & Science University	Portland, OR, USA	Site Coordinator	
Kathryn	Chung		MD	Oregon Health & Science University	Portland, OR, USA	Site Investigator	
Joohi	Shahed		MD	Baylor College of Medicine	Houston, TX, USA	Site Investigator	
Christine	Hunter		BSN	Baylor College of Medicine	Houston, TX, USA	Site Coordinator	
Binit	Shah		MD	University of Virginia	Charlottesville, VA, USA	Site Investigator	
Katie	Sullivan		MSW, CCRC	University of Virginia	Charlottesville, VA, USA	Site Coordinator	
Albert Y.	Hung		MD	Massachusetts General Hospital	Boston, MA, USA	Site Investigator	
Grace	Bwala		MBBS	Massachusetts General Hospital	Boston, MA, USA	Site Coordinator	
Meredith	Spindler		MD	University of Pennsylvania	Philadelphia, PA, USA	Site Investigator	
Alexandria	Oliver		BS	University of Pennsylvania	Philadelphia, PA, USA	Site Coordinator	
Robert A.	Hauser		MD	University of South Florida	Tampa, FL, USA	Site Investigator	
Claudia	Rocha		BS	University of South Florida	Tampa, FL, USA	Site Coordinator	
Eric	Molho		MD	Albany Medical Center	Albany, NY, USA	Site Investigator	
Sharon	Evans		LPN	Albany Medical Center	Albany, NY, USA	Site Coordinator	
Holly A.	Shill		MD	Barrow Neurological Institute	Phoenix, AZ, USA	Site Investigator	
Farah	Ismail		M.B.Ch.B	Barrow Neurological Institute	Phoenix, AZ, USA	Site Coordinator	
Natividad	Stover		MD	University of Alabama at Birmingham	Birmingham, AL, USA	Site Investigator	
Candace	Cromer		BS	University of Alabama at Birmingham	Birmingham, AL, USA	Site Coordinator	
Courtney	Blair		MA	University of Alabama at Birmingham	Birmingham, AL, USA	Site Coordinator	
Lin	Zhang		MD	UC Davis	Sacramento, CA	Site Investigator	
Olga	Kishchenko		BS	UC Davis	Sacramento, CA	Site Coordinator	

Supplemental Online Content: Nonauthor Collaborators

*Indicates required information. Only first name, last name, and suffix will appear in PubMed.

First Name, Middle Initial*	Last Name*	Suffix*	Academic Degrees	Institution	Location (city, state/province, country)	Role or Contribution, eg, chair, principal investigator	Subgroup, eg, Steering Committee
Matthew	Swan		MD	Icahn School of Medicine at Mount Sinai	New York, NY, USA	Site Investigator	
Laura	Ramirez		BSN	Icahn School of Medicine at Mount Sinai	New York, NY, USA	Site Coordinator	
Samuel	Frank		MD	Harvard Medical School	Boston, MA, USA	Site Investigator	
Stephanie	Burrows		BS	Harvard Medical School	Boston, MA, USA	Site Coordinator	
Andrew	Duker		MD	University of Cincinnati	Cincinnati, OH, USA	Site Investigator	
Christina	Gruenwald		BS	University of Cincinnati	Cincinnati, OH, USA	Site Coordinator	
Karen	Blindauer		MD	Medical College of Wisconsin	Milwaukee, WI, USA	Site Investigator	
Lynn	Wheeler		MS	Medical College of Wisconsin	Milwaukee, WI, USA	Site Coordinator	
Lauren	Seeberger		MD	University of Colorado	Boulder, CO, USA	Site Investigator	
Abigail	Simpson		BS	University of Colorado	Boulder, CO, USA	Site Coordinator	
Burton L.	Scott		MD	Duke University	Durham, NC, USA	Site Investigator	
Lisa	Gauger		BA	Duke University	Durham, NC, USA	Site Coordinator	
Anwar	Ahmed		MD	Cleveland Clinic	Cleveland, OH, USA	Site Investigator	
Yvette	Pitchford		MS	Cleveland Clinic	Cleveland, OH, USA	Site Coordinator	
Jennifer	Mule		BS	Cleveland Clinic	Cleveland, OH, USA	Site Coordinator	
Adolfo	Ramirez-Zamora		MD	University of Florida College of Medicine	Gainsville, FL, USA	Site Investigator	
Derek B.	Ridgeway		BS	University of Florida College of Medicine	Gainsville, FL, USA	Site Coordinator	
John Slevin	Slevin		MD	University of Kentucky	Lexington, KY, USA	Site Investigator	
Renee Wagner	Wagner		BA	University of Kentucky	Lexington, KY, USA	Site Coordinator	
Vanessa	Hinson		MD	Medical University of South Carolina	Charleston, SC	Site Investigator	
Shonna	Jenkins		BS	Medical University of South Carolina	Charleston, SC	Site Coordinator	
John L.	Goudreau		DO	Michigan State University, College of Osteopathic Medicine	East Lansing, MI, USA	Site Investigator	

Supplemental Online Content: Nonauthor Collaborators

*Indicates required information. Only first name, last name, and suffix will appear in PubMed.

First Name, Middle Initial*	Last Name*	Suffix*	Academic Degrees	Institution	Location (city, state/province, country)	Role or Contribution, eg, chair, principal investigator	Subgroup, eg, Steering Committee
Doozie	Russell		BS	Michigan State University, College of Osteopathic Medicine	East Lansing, MI, USA	Site Coordinator	
Zoltan	Mari		MD	Cleveland Clinic Lou Ruvo Center for Brain Health	Las Vegas, NV	Site Investigator	
Lilliana	Dumitrescu		MA	Cleveland Clinic Lou Ruvo Center for Brain Health	Las Vegas, NV	Site Coordinator	
Jason	Aldred		MD	Inland Northwest Research	Spokane, WA, USA	Site Investigator	
Melissa	Bixby		MS	Inland Northwest Research	Spokane, WA, USA	Site Coordinator	
Mark	LeDoux		MD	Veracity Neuroscience and University of Memphis	Memphis, TN, USA	Site Investigator	
