# Supplementary material for: Efficacy of Nilotinib in Patients With Moderately Advanced Parkinson Disease: A Randomized Clinical Trial
Source: JAMA Neurol. 2020 Dec 14;78(3):1–9. doi: 10.1001/jamaneurol.2020.4725 (PMC7737147; doi:10.1001/jamaneurol.2020.4725)
Supplement: Supplement 5. — Data sharing statement [file jamaneurol-e204725-s005.pdf]

Data Sharing Statement

Data
Data available: Yes
Data types: Deidentified participant data
How to access data: https://clinicaltrials.gov/ct2/show/NCT03205488
When available: With publication

Supporting Documents
Document types: Statistical/analytic code
How to access documents: https://clinicaltrials.gov/ct2/show/NCT03205488
When available: With publication

Additional Information
Who can access the data: anyone requesting the data
Types of analyses: for any purpose
Mechanisms of data availability: with investigator support
